# Supplementary material for: Changes in daily mental health service use and mortality at the commencement and lifting of COVID-19 ‘lockdown’ policy in 10 UK sites: a regression discontinuity in time design
Source: BMJ Open. 2021 May 26;11(5):e049721. doi: 10.1136/bmjopen-2021-049721 (PMC8159668; doi:10.1136/bmjopen-2021-049721)
Supplement: Supplementary data [file bmjopen-2021-049721supp001.pdf]

TECHNICAL APPENDIX

Rationale for statistical analysis

Regression discontinuity is a quasi-experimental design that has been applied previously for the evaluation of nationwide policies,[1] though this approach has found little application in epidemiology to date.[2,3] Regression discontinuity in time (RDiT),[4] applied here is being employed for the analysis of time series data. In our RDiT framework, we know the date *c* of a policy change (here 23rd March 2020 and 10th May 2020). Thus, we assume that for all dates $t > c$, the unit is 'treated', and for all dates $t < c$, the unit is not. This RDiT set-up uses time-series data: in our case, daily counts across 10 UK sites. Two comparable time intervals (cohorts) are chosen: in our case, a period in 2019 and one in 2020. In one interval, but not the other, an event (discontinuity) occurs: in this study, the start or end of 'lockdown'. Changes at the discontinuity are measured, with the other interval acting as the control. The key feature of RDiT is the focus on comparing outcomes in a 'short' time interval before the policy implementation with a 'short' time interval after the policy implementation relative to changes observed in the previous year. We therefore assume that no 2020-specific unobserved factors confound the relationship between the exposure and the outcome by using these short time windows (and in this analysis, considered changes in national infection rates as a particular confounder).

The conditions for a valid RDiT analysis[3] were met in our study: i) the decision rule (exposed or not exposed to the intervention) and cut-off value (23rd March 2020 and 10th May 2020) were known; ii) the assignment variable (distance from the lockdown announcement, measured in days) was continuous near the cut-off value and was not affected by the policy (see Supplementary Figures 1-4); iii) there was visual confirmation of an intervention effect, in that a graphical analysis (Supplementary Figures 1-4) confirmed the discontinuity, i.e. a visible jump at the cut-off value, indicating a policy effect. We also assumed parallel trends in the time window around the lockdown and lift of lockdown announcement (a form of common/parallel-trend assumption). We further fitted different models to our time series data, with our final model being visualised in Supplementary Figure 5.

Bakolis I, *et al. BMJ Open* 2021; 11:e049721. doi: 10.1136/bmjopen-2021-049721

Further details of statistical analyses

We adopted a parametric specification for the conditional expectation functions which account for heterogeneity of temporal effects (by weekday and month). We included functions to adjust for seasonal trends before and after the cut-off points

$$Y_{i,c} = \beta_0 + \beta_1 T_{i,c} + \beta_2 Year_{i,c} + \beta_3 T_{i,c} * Year_{i,c} + \beta_4 f(D_{i,c}) + \beta_5 f(D_{i,c}) * T_{i,c} + \beta_6 f(D_{i,c}) * Year_{i,c} + \beta_7 f(D_{i,c}) * T_{i,c} * Year_{i,c} + \beta_8 W_{i,c} + \beta_9 M_{i,c} + logE_c + \varepsilon_{i,c}$$

Within each day i and site c we employed negative binomial regression to model daily counts of mortality and mental health service use activity levels defined as $Y_{i,c}$. We allowed for overdispersion and accounted for a proportional relationship between activity counts and the total daily caseload of the site by mean of an offset $\log E_c$. $T_{i,c}$ is a categorical variable whose levels were: before lockdown, during lockdown, or after lockdown (or equivalent periods in 2019). The year of the lockdown is binary variable $Year_{i,c}$ and corresponds to 2020. $D_{i,c}$ is defined as the absolute distance in days from the lockdown order announcement -$f(D_{i,c})$ is a linear function of the distance in days from the lockdown announcement interacted with the lockdown variable $T_{i,c}$ and the $Year_{i,c}$ to allow for different effects on either side of the cut-off. The model also includes $W_{i,c}$ and $M_{i,c}$ defined as set of dummy variables of weekdays and months respectively. The parameter of interest, which identifies the effect of the lockdown, is the vector β_3 which is the coefficients associated with the interaction term between our pre-post policy dummy variable $T_{i,c}$and cohort variable $Year_{i,c}$. $\beta_1, \beta_2, \beta_3, \ldots\ldots, \beta_9$ are fixed effects coefficients. Differences in daily measures before versus after the cut-off dates are reported as relative risks (RR) with their corresponding 95% confidence intervals (CI). Negative binomial regression results were pooled across sites using random effects meta-analysis and heterogeneity was summarised using the I^2 statistic.

We performed the same negative binomial modelling with minor modifications for Site I weekly data. The time resolution was changed to week and only $M_{i,c}$ was included as an additional categorical covariate. Variables T_i and D_i were defined with respect to the week numbers of the announcement (week 13) and lift (week 20) of lockdown.

References

1. Bakolis I, Kelly R, Fecht D, et al. Protective effects of smoke-free legislation on birth outcomes in England: a regression discontinuity design. *Epidemiology* 2016; **27**: 810-8.

2. O'Keeffe AG, Geneletti S, Baio G, Sharples LD, Nazareth I, Petersen I. Regression discontinuity designs: an approach to the evaluation of treatment efficacy in primary care using observational data. *BMJ* 2014; **349**: g5293.

3. Moscoe E, Bor J, Bärnighausen T. Regression discontinuity designs are underutilized in medicine, epidemiology, and public health: a review of current and best practice. *Journal of Clinical Epidemiology* 2015; **68**: 132-43.

4. Imbens G, Lemieux T. Regression Discontinuity Designs: A Guide to Practice. *Journal of Econometrics* 2008; **142**: 615-35.

5. DerSimonian R, Laird N. Meta-analysis in clinical trials. *Controlled Clinical Trials* 1986; **7**: 177-88.

Supplementary Table 1: Site-specific data for lockdown initiation changes in daily mortality and service activity

	IRR (95%CI) for lockdown vs. pre-lockdown comparisons								
**Measures**	**Site A**	**Site B**	**Site C**	**Site D**	**Site E**	**Site F**	**Site G**	**Site H**	**Site J**
Number of deaths	3.47(2.92-4.14)	1.93(1.74-2.13)	4.00(1.25-12.85)	2.18(1.76-2.70)	5.30(2.87-9.79)	1.55(1.23-1.94)	2.41(1.69-3.44)	1.56(1.10-2.22)	2.23(1.76-2.84)
Trust-wide activity									
Number of new referrals accepted	0.64(0.46-0.90)	0.65(0.45-0.93)	N/A	0.62(0.46-0.83)	N/A	0.62(0.52-0.75)	N/A	0.62(0.43-0.89)	0.60(0.42-0.87)
Number of discharges	0.96(0.78-1.19)	1.08(0.75-1.56)	N/A	1.00(0.71-1.41)	N/A	0.89(0.73-1.09)	N/A	0.74(0.46-1.18)	1.05(0.78-1.41)
Inpatient care									
New admissions	0.81(0.67-0.99)	0.77(0.57-1.03)	0.59(0.45-0.77)	0.79(0.60-1.04)	0.72(0.47-1.11)	0.76(0.53-1.09)	N/A	0.73(0.53-0.99)	0.77(0.55-1.06)
Discharges	1.25(0.92-1.69)	1.06(0.68-1.63)	1.02(0.66-1.59)	1.24(0.75-2.06)	0.99(0.57-1.72)	1.10(0.72-1.69)	N/A	1.48(0.91-2.40)	0.92(0.62-1.39)
Inpatient caseload	0.77(0.75-0.79)	0.82(0.81-0.84)	0.91(0.90-0.92)	0.97(0.96-0.98)	0.79(0.77-0.80)	0.67(0.65-0.69)	N/A	0.89(0.87-0.90)	1.00(0.99-1.01)
Inpatient caseload (MHA)	0.84(0.82-0.86)	0.93(0.92-0.94)	N/A	1.00(0.99-1.02)	N/A	0.78(0.76-0.80)	N/A	0.99(0.97-1.00)	1.05(1.04-1.06)
AMH (community)									
F2f contacts	0.54(0.41-0.70)	0.44(0.27-0.71)	N/A	1.20(0.70-2.06)	0.72(0.42-1.23)	0.44(0.27-0.73)	0.37(0.14-0.97)	0.57(0.40-0.82)	0.41(0.29-0.59)
Non-f2f contacts	2.13(1.71-2.66)	3.16(2.11-4.74)	N/A	3.14(1.80-5.47)	3.04(1.75-5.31)	8.38(4.97-14.13)	8.22(3.65-18.56)	2.24(1.41-3.54)	5.32(3.74-7.55)
F2f and Non-f2f contacts	1.03(0.85-1.24)	1.21(0.77-1.89)	N/A	1.38(0.81-2.36)	1.11(0.65-1.89)	0.86(0.52-1.43)	0.51(0.20-1.30)	1.19(0.80-1.77)	1.15(0.83-1.61)
Cancelled/DNA	0.52(0.41-0.66)	1.24(0.76-2.02)	N/A	1.28(0.75-2.19)	0.45(0.25-0.81)	1.17(0.63-2.18)	0.54(0.22-1.38)	1.13(0.69-1.85)	0.93(0.62-1.41)
Caseload	1.02(1.00-1.04)	1.03(1.03-1.04)	N/A	1.02(1.00-1.04)	1.01(1.00-1.02)	1.02(1.01-1.03)	N/A	1.02(1.02-1.03)	1.06(1.05-1.07)
CAMHS									
F2f contacts	0.12(0.08-0.17)	N/A	N/A	1.01(0.62-1.63)	0.12(0.04-0.36)	0.99(0.59-1.65)	0.82(0.39-1.71)	0.32(0.20-0.51)	0.15(0.10-0.23)
Non-f2f contacts	2.53(2.03-3.15)	N/A	N/A	5.14(3.12-8.48)	30.64(3.9-67.51)	2.73(1.65-4.52)	8.87(4.10-19.15)	2.42(1.46-4.00)	3.02(2.05-4.47)
F2f and Non-f2f contacts	0.89(0.71-1.12)	N/A	N/A	1.41(0.87-2.29)	1.43(0.71-2.91)	1.15(0.69-1.93)	1.14(0.58-2.24)	1.25(0.77-2.04)	1.28(0.87-1.86)
Cancelled/DNA	0.83(0.66-1.04)	N/A	N/A	1.36(0.84-2.21)	0.62(0.13-2.98)	0.56(0.35-0.89)	2.91(0.95-8.96)	0.78(0.46-1.31)	0.80(0.54-1.20)
Caseload	1.02(1.00-1.04)	N/A	N/A	1.02(1.00-1.04)	0.83(0.82-0.85)	0.97(0.96-0.99)	N/A	0.94(0.93-0.95)	0.99(0.98-0.99)
EIP									
F2f contacts	0.19(0.15-0.26)	0.20(0.12-0.31)	N/A	1.06(0.61-1.87)	0.38(0.21-0.68)	0.16(0.09-0.30)	N/A	0.34(0.22-0.53)	0.16(0.12-0.23)
Non-f2f contacts	2.35(1.78-3.10)	3.71(2.49-5.51)	N/A	2.61(1.46-4.68)	7.95(4.33-14.60)	18.15(4.96-66.42)	N/A	2.17(1.50-3.14)	6.48(4.23-9.94)
F2f and Non-f2f contacts	0.97(0.76-1.23)	1.11(0.74-1.64)	N/A	1.34(0.77-2.34)	1.60(0.92-2.79)	0.47(0.26-0.84)	N/A	1.17(0.81-1.71)	1.24(0.97-1.58)
Cancelled/DNA	0.52(0.40-0.67)	1.30(0.80-2.13)	N/A	1.16(0.66-2.03)	0.38(0.19-0.78)	0.49(0.27-0.89)	N/A	1.10(0.72-1.70)	0.84(0.54-1.32)
Caseload	1.04(1.02-1.05)	1.01(1.00-1.02)	N/A	1.02(1.01-1.03)	1.01(1.00-1.02)	1.06(1.05-1.07)	N/A	1.04(1.03-1.05)	1.09(1.08-1.10)
HTT									
F2f contacts	0.47(0.44-0.51)	0.30(0.26-0.35)	N/A	0.75(0.69-0.83)	0.67(0.58-0.78)	0.48(0.41-0.57)	N/A	0.46(0.39-0.54)	0.15(0.11-0.20)
Non-f2f contacts	1.95(1.63-2.34)	2.06(1.80-2.35)	N/A	1.42(1.07-1.89)	2.79(1.73-4.50)	1.43(1.06-1.94)	N/A	1.64(1.45-1.86)	1.67(1.16-2.42)
F2f and Non-f2f contacts	0.76(0.71-0.81)	0.94(0.86-1.04)	N/A	0.83(0.77-0.90)	0.83(0.73-0.95)	0.65(0.56-0.76)	N/A	1.08(0.94-1.23)	0.38(0.28-0.52)
Cancelled/DNA	1.00(0.81-1.24)	0.97(0.78-1.22)	N/A	0.77(0.59-1.00)	0.48(0.33-0.70)	0.48(0.33-0.70)	N/A	0.94(0.76-1.16)	0.29(0.18-0.46)
Caseload	0.74(0.72-0.76)	0.56(0.53-0.59)	N/A	0.58(0.55-0.61)	0.42(0.39-0.45)	0.70(0.65-0.75)	N/A	0.98(0.97-0.99)	0.49(0.45-0.53)
Liaison									
F2f contacts	0.51(0.46-0.57)	0.34(0.24-0.49)	N/A	0.60(0.49-0.74)	0.36(0.27-0.47)	0.49(0.34-0.70)	0.38(0.28-0.51)	0.48(0.42-0.55)	0.29(0.23-0.37)
Non-f2f contacts	1.47(1.13-1.93)	1.69(0.95-2.99)	N/A	0.85(0.65-1.11)	1.22(0.51-2.89)	1.83(0.91-3.69)	3.37(1.77-6.42)	1.47(1.08-1.99)	1.63(1.16-2.29)
F2f and Non-f2f contacts	0.61(0.54-0.68)	0.53(0.37-0.75)	N/A	0.65(0.53-0.80)	0.39(0.29-0.51)	0.66(0.46-0.94)	0.74(0.56-0.98)	0.61(0.54-0.69)	0.59(0.49-0.70)
Cancelled/DNA	0.31(0.21-0.47)	0.41(0.13-1.33)	N/A	1.23(0.76-1.99)	0.15(0.02-1.35)	0.85(0.49-1.50)	2.05(0.80-5.24)	0.21(0.11-0.39)	1.04(0.67-1.63)
Caseload	0.91(0.90-0.92)	0.35(0.31-0.39)	N/A	0.92(0.91-0.93)	0.45(0.42-0.50)	0.82(0.80-0.84)	N/A	1.03(1.00-1.06)	0.92(0.90-0.93)
OA									
F2f contacts	0.28(0.20-0.38)	0.18(0.10-0.31)	N/A	1.21(0.70-2.11)	0.58(0.33-1.03)	0.19(0.11-0.33)	0.22(0.11-0.45)	0.40(0.24-0.65)	0.18(0.12-0.28)
Non-f2f contacts	1.84(1.44-2.36)	4.45(2.54-7.79)	N/A	3.22(1.86-5.57)	9.68(4.70-19.93)	3.78(2.27-6.31)	7.98(3.03-20.99)	2.40(1.41-4.09)	3.43(2.51-4.69)

Bakolis I, *et al. BMJ Open* 2021; 11:e049721. doi: 10.1136/bmjopen-2021-049721

F2f and Non-f2f contacts	0.74(0.59-0.94)	1.34(0.79-2.29)	N/A	1.77(1.02-3.08)	1.51(0.85-2.66)	0.63(0.38-1.03)	0.80(0.41-1.55)	1.17(0.71-1.92)	1.20(0.85-1.70)
Cancelled/DNA	0.60(0.37-0.99)	1.56(0.90-2.71)	N/A	1.52(0.87-2.65)	0.65(0.24-1.77)	1.85(1.17-2.93)	0.41(0.15-1.16)	0.73(0.47-1.14)	1.77(1.38-2.27)
Caseload	0.91(0.91-0.92)	1.00(1.00-1.00)	N/A	1.00(0.99-1.02)	0.92(0.91-0.93)	0.96(0.94-0.98)	N/A	0.94(0.93-0.95)	0.98(0.97-0.98)

AMH: adult mental health service; CAMHS: child and adolescent mental health service; EIP: early intervention for psychosis service; HTT: home treatment team; OA: older adult service. N/A: data not available for these type of services

Bakolis I, *et al. BMJ Open* 2021; 11:e049721. doi: 10.1136/bmjopen-2021-049721

Supplementary Table 2: Site-specific data for lift-of-lockdown changes in daily mortality and service activity

	IRR (95%CI) for lift of lockdown vs. lockdown comparisons								
**Measures**	**Site A**	**Site B**	**Site C**	**Site D**	**Site E**	**Site F**	**Site G**	**Site H**	**Site J**
Number of deaths	0.42(0.32-0.55)	0.69(0.59-0.81)	0.12(0.05-3.56)	0.14(0.10-0.22)	0.08(0.02-0.40)	1.06(0.79-1.43)	0.21(0.06-0.68)	0.56(0.32-0.98)	0.63(0.46-0.87)
Trust-wide activity									
Number of new referrals accepted	1.32(0.92-1.89)	1.55(0.90-2.65)	N/A	1.39(0.88-2.18)	N/A	1.33(1.02-1.74)	N/A	1.49(0.85-2.61)	1.21(0.70-2.08)
Number of discharges	0.90(0.71-1.15)	1.10(0.62-1.95)	N/A	0.97(0.58-1.60)	N/A	0.97(0.71-1.32)	N/A	1.40(0.66-2.96)	0.83(0.50-1.40)
Inpatient care									
New admissions	1.08(0.79-1.47)	1.47(0.95-2.27)	1.32(0.87-2.00)	1.27(0.84-1.91)	1.17(0.63-2.15)	0.87(0.48-1.58)	N/A	1.34(0.88-2.04)	1.18(0.66-2.12)
Discharges	0.51(0.34-0.76)	1.04(0.55-1.97)	0.90(0.48-1.72)	0.69(0.32-1.51)	0.44(0.16-1.20)	0.44(0.20-0.99)	N/A	0.60(0.29-1.23)	0.96(0.50-1.85)
Inpatient caseload	1.01(0.99-1.04)	1.14(1.13-1.16)	1.04(1.03-1.06)	0.94(0.93-0.95)	1.11(1.08-1.14)	1.04(1.00-1.07)	N/A	1.14(1.12-1.16)	1.08(1.07-1.09)
Inpatient caseload (MHA)	1.04(1.02-1.07)	1.08(1.07-1.10)	N/A	0.92(0.92-0.93)	N/A	1.07(1.03-1.11)	N/A	0.87(0.84-0.89)	1.09(1.08-1.10)
AMH (community)									
F2f contacts	0.82(0.59-1.15)	1.31(0.62-2.76)	N/A	1.23(0.52-2.91)	1.02(0.43-2.43)	0.91(0.35-2.40)	1.17(0.12-11.37)	1.16(0.67-2.00)	1.13(0.65-1.96)
Non-f2f contacts	1.35(1.07-1.70)	1.33(0.72-2.46)	N/A	1.30(0.53-3.22)	0.72(0.30-1.71)	0.62(0.25-1.49)	2.29(0.20-26.05)	1.15(0.56-2.38)	1.46(0.87-2.45)
F2f and Non-f2f contacts	1.10(0.88-1.38)	1.29(0.64-2.59)	N/A	1.23(0.52-2.91)	0.92(0.40-2.16)	0.78(0.31-1.97)	2.61(0.23-29.41)	1.14(0.61-2.14)	1.32(0.78-2.25)
Cancelled/DNA	1.24(0.95-1.62)	1.02(0.47-2.21)	N/A	0.96(0.41-2.26)	0.44(0.16-1.22)	0.49(0.21-1.16)	1.91(0.20-17.82)	1.17(0.56-2.46)	0.95(0.49-1.86)
Caseload	1.01(1.01-1.02)	1.01(1.00-1.01)	N/A	1.00(1.00-1.01)	1.01(1.01-1.01)	1.03(1.02-1.03)	N/A	1.01(1.00-1.01)	1.01(1.01-1.01)
CAMHS									
F2f contacts	1.29(0.60-2.80)	N/A	N/A	1.16(0.54-2.48)	2.25(0.56-9.08)	1.59(0.66-3.81)	0.30(0.04-2.12)	1.41(0.71-2.80)	0.58(0.28-1.19)
Non-f2f contacts	1.32(1.03-1.70)	N/A	N/A	1.04(0.46-2.36)	0.52(0.04-6.70)	0.80(0.40-1.60)	2.58(0.43-15.31)	1.38(0.63-3.03)	1.09(0.59-2.01)
F2f and Non-f2f contacts	1.25(0.93-1.67)	N/A	N/A	1.14(0.53-2.46)	1.33(0.48-3.68)	1.43(0.61-3.32)	0.47(0.07-2.98)	1.41(0.68-2.92)	0.93(0.53-1.66)
Cancelled/DNA	1.12(0.87-1.46)	N/A	N/A	0.73(0.33-1.63)	N/A	1.42(0.65-3.13)	0.07(0.01-0.64)	0.99(0.45-2.15)	0.85(0.44-1.65)
Caseload	0.99(0.99-0.99)	N/A	N/A	1.00(0.99-1.00)	1.11(1.09-1.12)	0.96(0.96-0.96)	N/A	0.98(0.97-0.98)	0.99(0.99-0.99)
EIP									
F2f contacts	1.16(0.85-1.60)	2.56(1.35-4.84)	N/A	1.11(0.47-2.65)	1.36(0.56-3.30)	3.92(1.77-8.72)	N/A	1.37(0.69-2.72)	1.70(1.05-2.76)
Non-f2f contacts	1.67(1.23-2.27)	1.30(0.68-2.48)	N/A	1.08(0.45-2.61)	0.54(0.22-1.34)	1.72(0.17-16.98)	N/A	1.41(0.80-2.49)	3.05(1.80-5.16)
F2f and Non-f2f contacts	1.37(1.04-1.81)	1.61(0.87-2.95)	N/A	1.09(0.46-2.57)	0.79(0.34-1.87)	2.02(0.92-4.41)	N/A	1.29(0.71-2.37)	1.64(1.22-2.22)
Cancelled/DNA	1.16(0.88-1.54)	0.74(0.36-1.55)	N/A	1.03(0.44-2.45)	0.24(0.09-0.65)	1.34(0.53-3.36)	N/A	1.27(0.67-2.39)	1.20(0.72-2.01)
Caseload	0.99(0.99-1.00)	1.06(1.04-1.07)	N/A	1.01(1.00-1.01)	1.00(1.00-1.00)	1.04(1.03-1.05)	N/A	1.10(1.10-1.11)	1.05(1.04-1.06)
HTT									
F2f contacts	1.55(1.37-1.75)	1.90(1.56-2.31)	N/A	1.40(1.21-1.61)	1.38(1.16-1.65)	1.38(1.13-1.67)	N/A	1.47(1.18-1.83)	1.15(0.73-1.81)
Non-f2f contacts	1.21(0.93-1.57)	1.11(0.93-1.34)	N/A	1.57(1.10-2.23)	0.61(0.33-1.13)	0.82(0.57-1.20)	N/A	0.86(0.72-1.02)	1.85(1.03-3.31)
F2f and Non-f2f contacts	1.37(1.24-1.52)	1.36(1.18-1.56)	N/A	1.39(1.22-1.59)	1.21(1.03-1.43)	1.19(0.97-1.46)	N/A	0.98(0.81-1.18)	1.42(0.92-2.17)
Cancelled/DNA	1.09(0.78-1.52)	1.34(0.97-1.85)	N/A	1.88(1.30-2.72)	1.49(0.76-2.92)	3.00(1.72-5.21)	N/A	0.93(0.67-1.27)	1.91(0.87-4.23)
Caseload	1.22(1.18-1.27)	1.26(1.17-1.36)	N/A	1.23(1.16-1.31)	1.35(1.24-1.46)	1.25(1.14-1.36)	N/A	0.93(0.91-0.94)	0.35(0.33-0.38)
Liaison									
F2f contacts	1.81(1.52-2.14)	2.11(1.22-3.64)	N/A	1.48(1.06-2.05)	1.58(1.04-2.42)	1.50(0.88-2.56)	0.98(0.53-1.82)	1.40(1.17-1.67)	2.67(1.93-3.67)
Non-f2f contacts	1.31(0.91-1.89)	0.59(0.25-1.36)	N/A	1.12(0.74-1.69)	2.62(0.79-8.69)	6.11(1.58-23.63)	1.09(0.35-3.40)	0.74(0.48-1.14)	0.93(0.57-1.52)
F2f and Non-f2f contacts	1.68(1.43-1.97)	1.51(0.88-2.60)	N/A	1.37(0.98-1.92)	1.57(1.02-2.43)	1.80(1.02-3.18)	0.87(0.45-1.67)	1.24(1.04-1.47)	1.55(1.21-1.99)
Cancelled/DNA	1.39(0.70-2.76)	1.04(0.10-10.5)	N/A	0.92(0.46-1.82)	6.97(0.41-117.19)	0.44(0.19-1.02)	0.13(0.01-1.21)	0.12(0.02-0.89)	0.83(0.51-1.35)
Caseload	1.10(1.08-1.12)	1.19(1.02-1.39)	N/A	0.99(0.98-1.01)	1.90(1.71-2.11)	1.16(1.12-1.20)	N/A	1.04(1.01-1.06)	1.00(0.98-1.01)
OA									
F2f contacts	1.98(1.37-2.86)	1.09(0.46-2.61)	N/A	1.65(0.69-3.96)	1.18(0.45-3.12)	0.76(0.34-1.72)	0.68(0.12-3.67)	1.20(0.53-2.71)	1.25(0.65-2.40)
Non-f2f contacts	1.20(0.87-1.66)	1.34(0.58-3.10)	N/A	0.91(0.38-2.21)	1.23(0.39-3.92)	1.34(0.65-2.78)	0.45(0.05-3.91)	1.37(0.61-3.08)	1.65(0.99-2.75)
F2f and Non-f2f contacts	1.46(1.07-1.99)	1.20(0.52-2.77)	N/A	1.28(0.53-3.06)	1.15(0.46-2.89)	0.88(0.41-1.87)	0.65(0.12-3.46)	1.16(0.53-2.56)	1.47(0.85-2.53)

Bakolis I, *et al. BMJ Open* 2021; 11:e049721. doi: 10.1136/bmjopen-2021-049721

Cancelled/DNA	2.15(1.10-4.17)	0.70(0.29-1.70)	N/A	0.82(0.34-2.01)	3.36(0.49-23.00)	0.15(0.07-0.34)	0.41(0.04-4.20)	0.64(0.29-1.43)	0.58(0.37-0.91)
Caseload	0.91(0.90-0.92)	0.99(0.98-0.99)	N/A	0.95(0.95-0.96)	1.11(1.09-1.13)	0.99(0.97-1.01)	N/A	0.99(0.98-1.00)	0.98(0.98-0.99)

AMH: adult mental health service; CAMHS: child and adolescent mental health service; EIP: early intervention for psychosis service; HTT: home treatment team; OA: older adult service. N/A: data not available for these type of services

Supplementary Table 3: Site-specific data for lift-of-lockdown vs. pre-lockdown changes in daily mortality and service activity

	IRR (95%CI) for lift of lockdown vs. pre-lockdown comparisons								
**Measures**	**Site A**	**Site B**	**Site C**	**Site D**	**Site E**	**Site F**	**Site G**	**Site H**	**Site J**
Number of deaths	1.46(1.15-1.85)	1.32(1.15-1.53)	0.49(0.02-12.44)	0.31(0.22-0.46)	0.43(0.09-2.00)	1.64(1.29-2.09)	0.50(0.16-1.58)	0.87(0.52-1.45)	1.42(1.10-1.82)
Trust-wide activity									
Number of new referrals accepted	0.85(0.72-0.99)	1.00(0.63-1.58)	N/A	0.85(0.58-1.26)	N/A	0.83(0.67-1.04)	N/A	0.92(0.57-1.49)	0.73(0.46-1.15)
Number of discharges	0.87(0.75-1.01)	1.19(0.73-1.96)	N/A	0.96(0.63-1.47)	N/A	0.86(0.67-1.11)	N/A	1.03(0.54-1.98)	0.88(0.54-1.41)
Inpatient care									
New admissions	0.88(0.66-1.15)	1.13(0.78-1.63)	0.76(0.53-1.09)	1.00(0.71-1.42)	0.84(0.52-1.36)	0.66(0.39-1.12)	N/A	0.97(0.68-1.40)	0.90(0.53-1.53)
Discharges	0.64(0.47-0.86)	1.10(0.63-1.89)	0.83(0.51-1.35)	0.86(0.43-1.69)	0.44(0.18-1.08)	0.49(0.23-1.03)	N/A	0.89(0.48-1.63)	0.89(0.50-1.56)
Inpatient caseload	0.78(0.77-0.79)	0.94(0.93-0.95)	0.95(0.94-0.96)	0.91(0.90-0.92)	0.87(0.85-0.89)	0.69(0.67-0.71)	N/A	1.01(1.00-1.02)	1.08(1.07-1.09)
Inpatient caseload (MHA)	0.88(0.87-0.89)	1.01(1.00-1.02)	N/A	0.93(0.92-0.93)	N/A	0.83(0.81-0.85)	N/A	0.86(0.84-0.88)	1.14(1.13-1.15)
AMH (community)									
F2f contacts	0.44(0.34-0.56)	0.57(0.30-1.09)	N/A	1.48(0.70-3.14)	0.73(0.34-1.55)	0.40(0.17-0.97)	0.43(0.05-3.58)	0.67(0.41-1.08)	0.46(0.29-0.76)
Non-f2f contacts	2.88(2.43-3.41)	4.20(2.46-7.17)	N/A	4.08(1.84-9.05)	2.18(1.02-4.64)	5.17(2.29-11.67)	18.87(1.87-90.10)	2.57(1.37-4.84)	7.79(5.04-12.04)
F2f and Non-f2f contacts	1.13(0.96-1.33)	1.56(0.85-2.85)	N/A	1.70(0.80-3.61)	1.02(0.49-2.14)	0.67(0.29-1.55)	1.33(0.14-12.90)	1.36(0.78-2.35)	1.53(0.95-2.44)
Cancelled/DNA	0.65(0.56-0.75)	1.26(0.65-2.46)	N/A	1.23(0.59-2.60)	0.20(0.08-0.50)	0.58(0.29-1.14)	1.04(0.13-8.21)	1.32(0.70-2.50)	0.89(0.50-1.59)
Caseload	1.04(1.03-1.04)	1.04(1.04-1.04)	N/A	1.02(1.02-1.02)	1.01(1.01-1.01)	1.04(1.04-1.05)	N/A	1.03(1.03-1.03)	1.07(1.07-1.07)
CAMHS									
F2f contacts	0.15(0.07-0.32)	N/A	N/A	1.16(0.60-2.26)	0.27(0.09-0.77)	1.57(0.72-3.40)	0.24(0.04-1.55)	0.45(0.25-0.82)	0.09(0.05-0.16)
Non-f2f contacts	3.35(2.76-4.06)	N/A	N/A	5.36(2.62-10.99)	15.89(2.94-85.84)	2.19(1.21-3.96)	22.85(4.40-81.80)	3.33(1.69-6.56)	3.30(1.93-5.65)
F2f and Non-f2f contacts	1.11(0.89-1.38)	N/A	N/A	1.61(0.82-3.15)	1.91(0.79-4.59)	1.64(0.79-3.43)	0.53(0.09-3.06)	1.77(0.95-3.27)	1.19(0.72-1.96)
Cancelled/DNA	0.93(0.78-1.11)	N/A	N/A	1.00(0.49-2.02)	N/A	0.79(0.39-1.60)	0.22(0.03-1.40)	0.77(0.40-1.48)	0.69(0.38-1.23)
Caseload	1.01(1.01-1.01)	N/A	N/A	1.02(1.01-1.02)	0.92(0.91-0.94)	0.93(0.92-0.94)	N/A	0.91(0.90-0.92)	0.97(0.97-0.97)
EIP									
F2f contacts	0.23(0.19-0.28)	0.50(0.30-0.84)	N/A	1.19(0.57-2.49)	0.52(0.24-1.09)	0.63(0.34-1.15)	N/A	0.47(0.26-0.84)	0.28(0.18-0.42)
Non-f2f contacts	3.91(3.18-4.82)	4.83(2.69-8.67)	N/A	2.82(1.29-6.16)	4.33(2.00-9.38)	31.28(3.75-60.97)	N/A	3.07(1.88-5.00)	19.75(12.4-31.2)
F2f and Non-f2f contacts	1.33(1.12-1.58)	1.78(1.04-3.02)	N/A	1.46(0.70-3.06)	1.27(0.61-2.64)	0.95(0.51-1.76)	N/A	1.52(0.90-2.58)	2.03(1.64-2.53)
Cancelled/DNA	0.60(0.51-0.71)	0.97(0.52-1.81)	N/A	1.20(0.57-2.51)	0.09(0.04-0.21)	0.66(0.30-1.41)	N/A	1.40(0.82-2.40)	1.02(0.71-1.44)
Caseload	1.04(1.03-1.04)	1.07(1.06-1.08)	N/A	1.03(1.02-1.03)	1.01(1.00-1.01)	1.10(1.09-1.11)	N/A	1.15(1.14-1.15)	1.15(1.14-1.15)
HTT									
F2f contacts	0.73(0.66-0.82)	0.57(0.49-0.66)	N/A	1.05(0.93-1.19)	0.93(0.82-1.06)	0.67(0.58-0.77)	N/A	0.68(0.56-0.82)	0.17(0.11-0.25)
Non-f2f contacts	2.36(1.87-2.97)	2.29(1.95-2.70)	N/A	2.23(1.64-3.02)	1.69(1.03-2.80)	1.18(0.88-1.59)	N/A	1.41(1.22-1.63)	3.09(1.81-5.28)
F2f and Non-f2f contacts	1.04(0.96-1.14)	1.28(1.14-1.44)	N/A	1.16(1.03-1.30)	1.01(0.88-1.15)	0.78(0.67-0.91)	N/A	1.06(0.91-1.23)	0.55(0.39-0.77)
Cancelled/DNA	1.09(0.81-1.46)	1.31(1.00-1.71)	N/A	1.44(1.07-1.94)	0.72(0.39-1.31)	1.44(0.90-2.32)	N/A	0.87(0.65-1.15)	0.55(0.28-1.10)
Caseload	0.91(0.88-0.94)	0.71(0.67-0.75)	N/A	0.71(0.68-0.75)	0.56(0.52-0.60)	0.87(0.80-0.94)	N/A	0.91(0.89-0.92)	0.17(0.16-0.18)
Liaison									
F2f contacts	0.93(0.80-1.08)	0.72(0.45-1.15)	N/A	0.89(0.67-1.18)	0.56(0.39-0.81)	0.73(0.47-1.14)	0.37(0.21-0.64)	0.67(0.59-0.77)	0.78(0.60-1.01)
Non-f2f contacts	1.93(1.41-2.65)	0.99(0.47-2.06)	N/A	0.95(0.67-1.35)	3.18(1.11-9.11)	11.20(3.05-41.19)	3.67(1.36-9.94)	1.09(0.76-1.56)	1.51(0.97-2.37)
F2f and Non-f2f contacts	1.02(0.89-1.16)	0.80(0.50-1.28)	N/A	0.89(0.67-1.19)	0.61(0.42-0.88)	1.18(0.72-1.93)	0.64(0.35-1.18)	0.75(0.65-0.86)	0.91(0.74-1.14)
Cancelled/DNA	0.44(0.24-0.81)	0.42(0.05-3.45)	N/A	1.13(0.65-1.98)	1.06(0.15-7.26)	0.38(0.19-0.76)	0.26(0.03-2.06)	0.02(0.00-0.19)	0.87(0.56-1.33)
Caseload	1.00(0.98-1.02)	0.41(0.36-0.47)	N/A	0.91(0.90-0.92)	0.86(0.79-0.94)	0.95(0.92-0.99)	N/A	1.06(1.03-1.10)	0.91(0.90-0.92)
OA									
F2f contacts	0.55(0.44-0.70)	0.20(0.09-0.42)	N/A	2.00(0.94-4.26)	0.69(0.29-1.63)	0.15(0.07-0.29)	0.15(0.03-0.72)	0.48(0.23-0.98)	0.23(0.13-0.40)
Non-f2f contacts	2.21(1.73-2.83)	5.98(2.9-12.2)	N/A	2.94(1.36-6.38)	11.92(4.31-32.98)	5.08(2.75-9.39)	3.56(0.49-26.01)	3.28(1.64-6.58)	5.66(3.58-8.95)
F2f and Non-f2f contacts	1.09(0.87-1.36)	1.61(0.78-3.33)	N/A	2.26(1.06-4.84)	1.73(0.77-3.90)	0.55(0.29-1.05)	0.52(0.11-2.47)	1.36(0.68-2.70)	1.76(1.10-2.82)

Cancelled/DNA	1.30(0.76-2.20)	1.09(0.50-2.37)	N/A	1.25(0.57-2.73)	2.19(0.39-12.32)	0.28(0.13-0.58)	0.17(0.02-1.41)	0.47(0.23-0.97)	1.03(0.67-1.57)
Caseload	0.83(0.83-0.84)	0.99(0.99-0.99)	N/A	0.95(0.95-0.95)	1.02(1.01-1.03)	0.95(0.94-0.96)	N/A	0.93(0.92-0.94)	0.96(0.96-0.96)

AMH: adult mental health service; CAMHS: child and adolescent mental health service; EIP: early intervention for psychosis service; HTT: home treatment team; OA: older adult service. N/A: data not available for these type of services

Supplementary Table 4. Meta-analysed effects of lockdown announcements on mortality and mental health service use activity omitting one week around the two transition dates (23rd March and 10th May).

	Lockdown Announcement vs Pre-lockdown		Lift of Lockdown Announcement vs Lockdown		Lift of Lockdown vs. Pre-lockdown	
**Measures**	IRR (95%CI)	I^2	IRR (95%CI)	I^2	IRR (95%CI)	I^2
Number of deaths	2.40*(2.02-2.84)	67.5	0.33*(0.18-0.60)	72.7	0.79 (0.46-1.33)	63.8
Trust-wide activity						
Number of new referrals accepted	0.69*(0.60-0.79)	0.0	1.68*(1.21-2.35)	0.0	1.09 (0.81-1.47)	0.0
Number of discharges	0.86*(0.77-0.97)	0.0	1.34 (0.91-1.98)	0.0	1.23 (0.86-1.76)	0.0
Inpatient care						
New admissions	0.82^(0.68-0.98)	49.8	1.62*(1.21-2.17)	0.0	1.14 (0.80-1.63)	39.3
Discharges	0.96 (0.80-1.16)	0.0	1.23 (0.69-2.18)	26.5	1.25 (0.78-1.99)	13.4
Inpatient caseload	0.84*(0.76-0.92)	99.8	1.13*(1.06-1.21)	98.3	0.95 (0.89-1.01)	99.2
Inpatient caseload on MHA section	0.96 (0.89-1.03)	99.5	0.99 (0.90-1.10)	98.8	0.95 (0.85-1.07)	99.3
AMH (community)						
F2f contacts	0.55*(0.44-0.69)	14.8	1.97*(1.15-3.37)	0.0	1.19 (0.73-1.95)	0.0
Non-f2f contacts	4.54*(2.84-7.25)	85.7	1.52 (0.94-2.45)	0.0	5.35*(3.43-8.35)	0.0
F2f and Non-f2f contacts	1.23*(1.06-1.44)	0.0	1.90*(1.16-3.10)	0.0	2.37*(1.50-3.75)	0.0
Cancelled appointments or DNAs	0.74 (0.54-1.02)	59.3	1.85*(1.05-3.26)	0.0	1.57 (0.92-2.66)	0.0
Caseload	1.05*(1.03-1.07)	98.6	1.01 (1.00-1.02)	89.4	1.04^(1.02-1.06)	98.3
CAMHS						
F2f contacts	0.38*(0.16-0.91)	90.9	1.94 (0.95-3.97)	0.0	0.86 (0.45-1.64)	0.0
Non-f2f contacts	4.67*(3.34-6.53)	57.5	0.98 (0.48-1.99)	21.1	4.87*(1.99-11.89)	52.5
F2f and Non-f2f contacts	1.46*(1.22-1.75)	0.0	1.71 (0.95-3.07)	0.0	2.55*(1.48-4.41)	0.0
Cancelled appointments or DNAs	0.80 (0.64-1.01)	17.8	1.29 (0.67-2.46)	0.0	1.03 (0.57-1.88)	0.0
Caseload	0.92*(0.89-0.96)	98.8	0.98 (0.96-1.00)	98.3	0.94^(0.89-0.99)	99.8
EIP						
F2f contacts	0.31*(0.21-0.45)	71.9	1.49 (0.85-2.61)	3.5	0.47 (0.21-1.04)	57.5
Non-f2f contacts	5.61*(3.08-10.22)	88.7	7.44 (0.63-48.53)	95.8	4.03*(3.58-14.05)	96.5
F2f and Non-f2f contacts	1.35*(1.17-1.56)	0.0	1.24 (0.78-1.98)	0.0	1.69*(1.10-2.61)	0.0
Cancelled appointments or DNAs	0.83 (0.58-1.20)	66.0	1.44 (0.73-2.85)	21.0	1.18 (0.48-2.92)	60.1
Caseload	1.06^(1.01-1.11)	99.8	1.03 (1.00-1.06)	99.3	1.09*(1.04-1.13)	99.6
HTT						
F2f contacts	0.45*(0.31-0.65)	97.1	1.49*(1.24-1.80)	39.0	0.76 (0.57-1.01)	76.3
Non-f2f contacts	1.90*(1.62-2.23)	58.8	1.05 (0.74-1.49)	63.1	2.02*(1.52-2.68)	54.9
F2f and Non-f2f contacts	0.88*(0.80-0.96)	66.1	1.27*(1.11-1.46)	26.8	1.14 (0.96-1.34)	52.8
Cancelled appointments or DNAs	0.71*(0.52-0.97)	82.2	1.27 (0.91-1.79)	29.5	1.05 (0.67-1.63)	64.2
Caseload	0.53*(0.36-0.79)	99.9	1.18(0.94-1.49)	98.9	0.63*(0.50-0.79)	99.4
Liaison						
F2f contacts	0.46*(0.36-0.60)	84.9	1.70*(1.34-2.17)	20.5	0.80*(0.67-0.94)	0.0
Non-f2f contacts	1.67*(1.15-2.41)	75.7	1.32 (0.90-1.93)	2.7	1.90*(1.19-3.04)	37.2
F2f and Non-f2f contacts	0.63*(0.52-0.76)	75.6	1.49*(1.10-2.01)	46.4	0.88 (0.74-1.05)	0.0
Cancelled appointments or DNAs	0.74 (0.45-1.22)	67.6	1.26 (0.51-3.12)	0.0	1.11 (0.42-2.88)	13.6
Caseload	0.77*(0.72-0.82)	98.7	1.19*(1.09-1.30)	95.9	0.87 (0.78-0.97)	97.8
OA						
F2f contacts	0.33*(0.21-0.53)	77.4	2.03*(1.09-3.78)	0.0	0.93 (0.45-1.89)	31.0
Non-f2f contacts	4.14*(2.69-6.37)	75.5	1.50 (0.83-2.71)	0.0	6.29*(2.95-13.41)	41.4
F2f and Non-f2f contacts	1.19 (0.86-1.64)	58.6	1.64 (0.92-2.95)	0.0	2.38*(1.23-4.60)	28.5
Cancelled appointments or DNAs	0.99 (0.69-1.42)	58.7	1.58 (0.74-3.39)	0.0	1.72 (0.85-3.49)	0.0
Caseload	0.91^(0.85-0.97)	99.8	1.02 (0.99-1.06)	99.3	0.94 (0.89-1.00)	99.7

^p<0.05; *p<0.001

AMH: adult mental health service; CAMHS: child and adolescent mental health service; EIP: early intervention for psychosis service; HTT: home treatment team; OA: older adult service.

Supplementary Table 5. Meta-analysed effects of lockdown announcements on mortality and mental health service use activity further adjusted for national covid-19 related deaths.

	Lockdown Announcement vs Pre-lockdown		Lift of Lockdown Announcement Vs lockdown		Lift of Lockdown vs. Pre-lockdown	
**Measures**	IRR (95%CI)	I^2	IRR (95%CI)	I^2	IRR (95%CI)	I^2
Number of deaths	1.74*(1.23-2.46)	88.1	0.44*(0.24-0.78)	92.1	0.71 (0.42-1.21)	90.9
Trust-wide activity						
Number of new referrals accepted	0.69*(0.59-0.81)	0.0	1.28*(1.07-1.53)	0.0	0.90 (0.78-1.04)	0.0
Number of discharges	1.10 (0.95-1.29)	0.0	0.88 (0.75-1.04)	0.0	0.96 (0.84-1.11)	0.0
Inpatient care						
New admissions	0.71*(0.60-0.85)	22.9	1.18 (1.00-1.41)	0.0	0.86 (0.73-1.01)	7.1
Discharges	1.60*(1.32-1.94)	1.3	0.52*(0.41-0.66)	0.0	0.86 (0.69-1.08)	0.0
Inpatient caseload	0.89*(0.84-0.95)	99.4	1.03 (0.97-1.09)	99.2	0.91^(0.84-0.99)	99.8
Inpatient caseload on MHA section	0.95 (0.89-1.01)	98.3	0.99 (0.90-1.08)	99.4	0.93 (0.82-1.05)	99.8
AMH (community)						
F2f contacts	0.59*(0.45-0.77)	41.4	0.94 (0.76-1.16)	0.0	0.61*(0.43-0.86)	50.0
Non-f2f contacts	4.09*(2.55-6.55)	81.1	1.27*(1.05-1.52)	0.0	4.07*(2.62-6.32)	69.6
F2f and Non-f2f contacts	1.04 (0.90-1.20)	0.0	1.12 (0.94-1.32)	0.0	1.18 (0.99-1.40)	0.0
Cancelled appointments or DNAs	0.99 (0.66-1.47)	66.4	0.87 (0.62-1.23)	35.9	0.86 (0.60-1.23)	50.1
Caseload	1.01 (1.00-1.02)	85.1	1.01 (1.00-1.02)	95.1	1.04^(1.02-1.07)	98.8
CAMHS						
F2f contacts	0.36*(0.16-0.84)	92.1	1.11 (0.77-1.60)	16.7	0.38*(0.16-0.91)	86.3
Non-f2f contacts	4.05*(2.63-6.24)	74.2	1.23^(1.02-1.49)	0.0	3.97*(2.78-5.66)	47.7
F2f and Non-f2f contacts	1.12 (0.88-1.41)	26.2	1.22 (0.99-1.51)	0.0	1.24 (1.00-1.52)	2.1
Cancelled appointments or DNAs	1.09 (0.81-1.47)	45.7	0.94 (0.73-1.22)	15.8	0.98 (0.81-1.18)	0.0
Caseload	0.95 (0.92-0.99)	99.5	1.03 (0.90-1.19)	99.7	0.95 (0.91-1.00)	99.6
EIP						
F2f contacts	0.33*(0.21-0.51)	74.6	1.40^(1.02-1.91)	38.3	0.48*(0.30-0.75)	76.9
Non-f2f contacts	4.56*(2.57-8.11)	85.4	1.45 (0.97-2.17)	57.5	6.07*(2.92-12.60)	88.6
F2f and Non-f2f contacts	1.15 (0.89-1.48)	50.3	1.41*(1.20-1.66)	0.0	1.53*(1.20-1.94)	40.4
Cancelled appointments or DNAs	0.77 (0.48-1.26)	75.8	1.01 (0.74-1.37)	36.4	0.70 (0.38-1.29)	87.0
Caseload	1.04^(1.02-1.05)	93.6	1.03(0.99-1.07)	99.3	1.08^(1.03-1.13)	99.7
HTT						
F2f contacts	0.44*(0.34-0.58)	92.5	1.50*(1.39-1.61)	0.0	0.65*(0.51-0.83)	92.3
Non-f2f contacts	1.70*(1.48-1.95)	20.7	1.08 (0.83-1.41)	70.6	1.80*(1.34-2.40)	80.6
F2f and Non-f2f contacts	0.67*(0.53-0.84)	92.5	1.30*(1.14-1.47)	67.5	0.89 (0.75-1.06)	86.6
Cancelled appointments or DNAs	0.56*(0.37-0.83)	83.5	1.63*(1.08-2.44)	77.9	0.97 (0.78-1.21)	37.5
Caseload	0.65*(0.52-0.83)	99.5	0.95 (0.67-1.35)	99.8	0.62*(0.45-0.86)	99.7
Liaison						
F2f contacts	0.48*(0.41-0.55)	57.6	1.63*(1.38-1.93)	51.1	0.77*(0.66-0.91)	57.3
Non-f2f contacts	1.52*(1.07-2.15)	72.0	1.20 (0.83-1.75)	62.8	1.73*(1.18-2.54)	68.8
F2f and Non-f2f contacts	0.62*(0.55-0.70)	46.8	1.43*(1.22-1.68)	51.4	0.88 (0.77-1.01)	42.4
Cancelled appointments or DNAs	0.81 (0.39-1.71)	88.7	0.68 (0.39-1.16)	53.0	0.72 (0.43-1.21)	60.9
Caseload	0.86*(0.81-0.91)	97.8	1.13*(1.06-1.21)	98.2	0.96 (0.92-1.00)	95.7
OA						
F2f contacts	0.39*(0.23-0.66)	81.9	1.31 (0.95-1.80)	24.6	0.46*(0.26-0.85)	80.4
Non-f2f contacts	3.84*(2.43-6.08)	82.3	1.24 (1.00-1.52)	0.0	4.20*(2.61-6.77)	71.1
F2f and Non-f2f contacts	1.09 (0.80-1.48)	61.2	1.30*(1.06-1.60)	0.0	1.31 (0.93-1.85)	48.1
Cancelled appointments or DNAs	1.34 (0.90-1.99)	63.8	0.63 (0.33-1.23)	74.9	0.88 (0.49-1.57)	70.0
Caseload	0.97 (0.95-1.00)	98.8	0.98 (0.92-1.04)	99.7	0.95 (0.89-1.02)	99.8

^p<0.05; *p<0.001

AMH: adult mental health service; CAMHS: child and adolescent mental health service; EIP: early intervention for psychosis service; HTT: home treatment team; OA: older adult service.

Bakolis I, *et al. BMJ Open* 2021; 11:e049721. doi: 10.1136/bmjopen-2021-049721

Supplementary Table 6. Meta-analysed effects of the lockdown announcement on mortality and mental health service use activity with the use of equally spaced windows around the cut-off date of 23rd March

	Lockdown Announcement Vs Pre-lockdown		Lift of the Lockdown vs. Pre-lockdown	
**Measures**	IRR (95%CI)	I^2	IRR (95%CI)	I^2
Number of deaths	2.13 (1.96-2.31)	4.1	0.82 (0.55-1.21)	86.0
Trust-wide activity				
Number of new referrals accepted	0.65 (0.58-0.74)	0.0	0.88 (0.74-1.03)	0.0
Number of discharges	0.86 (0.77-0.96)	0.0	0.88 (0.74-1.04)	3.4
Inpatient care				
New admissions	0.79 (0.69-0.90)	24.2	0.97 (0.83-1.12)	0.0
Discharges	1.04 (0.88-1.23)	0.0	0.82 (0.65-1.03)	0.0
Inpatient caseload	0.85 (0.80-0.91)	99.4	0.93 (0.86-0.99)	99.8
Inpatient caseload on MHA section	0.95 (0.90-1.01)	98.8	0.94 (0.84-1.06)	99.8
AMH (community)				
F2f contacts	0.56 (0.45-0.70)	40.3	0.68 (0.53-0.88)	0.0
Non-f2f contacts	3.70 (2.63-5.20)	77.6	3.32 (2.50-4.42)	23.7
F2f and Non-f2f contacts	1.16 (1.02-1.33)	0.0	1.34 (1.07-1.69)	0.0
Cancelled appointments or DNAs	0.77 (0.59-1.02)	59.3	0.87 (0.60-1.26)	39.6
Caseload	1.05 (1.03-1.06)	93.8	1.04 (1.03-1.06)	97.7
CAMHS				
F2f contacts	0.37 (0.18-0.77)	90.5	0.40 (0.19-0.83)	71.3
Non-f2f contacts	3.89 (2.83-5.34)	60.3	3.63 (2.23-5.91)	56.5
F2f and Non-f2f contacts	1.32 (1.11-1.55)	0.0	1.37 (1.03-1.81)	0.0
Cancelled appointments or DNAs	0.83 (0.66-1.04)	26.0	0.79 (0.59-1.06)	0.0
Caseload	0.96 (0.93-0.99)	99.0	0.96 (0.92-1.00)	99.4
EIP				
F2f contacts	0.30 (0.20-0.46)	81.5	0.45 (0.31-0.66)	52.8
Non-f2f contacts	3.65 (2.39-5.59)	80.8	4.30 (2.73-6.78)	64.7
F2f and Non-f2f contacts	1.22 (1.05-1.40)	11.9	1.48 (1.19-1.84)	0.0
Cancelled appointments or DNAs	0.69 (0.47-1.01)	75.2	0.58 (0.29-1.16)	85.6
Caseload	1.06 (1.04-1.09)	98.3	1.10 (1.04-1.16)	99.7
HTT				
F2f contacts	0.44 (0.33-0.60)	96.3	0.70 (0.57-0.87)	88.3
Non-f2f contacts	1.69 (1.47-1.94)	53.6	1.62 (1.19-2.22)	88.1
F2f and Non-f2f contacts	0.82 (0.75-0.91)	74.7	0.97 (0.84-1.13)	82.8
Cancelled appointments or DNAs	0.74 (0.61-0.90)	68.5	1.10 (0.87-1.38)	54.9
Caseload	0.59 (0.46-0.77)	99.5	0.62 (0.46-0.85)	99.7
Liaison				
F2f contacts	0.50 (0.42-0.59)	77.2	0.75 (0.64-0.88)	47.7
Non-f2f contacts	1.56 (1.20-2.03)	64.6	1.50 (1.10-2.06)	52.3
F2f and Non-f2f contacts	0.66 (0.57-0.76)	70.3	0.84 (0.75-0.94)	10.8
Cancelled appointments or DNAs	0.62 (0.39-0.99)	69.4	1.08 (0.76-1.53)	0.0
Caseload	0.77 (0.70-0.84)	99.2	0.90 (0.82-1.00)	99.1
OA				
F2f contacts	0.34 (0.21-0.53)	82.7	0.48 (0.29-0.80)	64.7
Non-f2f contacts	3.39 (2.38-4.82)	72.2	3.60 (2.38-5.43)	49.3
F2f and Non-f2f contacts	1.10 (0.87-1.40)	46.8	1.32 (0.92-1.87)	38.2
Cancelled appointments or DNAs	0.90 (0.65-1.26)	64.5	0.66 (0.43-1.03)	39.8
Caseload	0.93 (0.91-0.96)	98.7	0.93 (0.87-1.00)	99.8

AMH: adult mental health service; CAMHS: child and adolescent mental health service; EIP: early intervention for psychosis service; HTT: home treatment team; OA: older adult service.

Supplementary Table 7. Meta-analysed effects of lockdown announcements on mortality and mental health service use activity further adjusted for covid-19 google trends in United Kingdom

	Lockdown Announcement Vs Pre-lockdown		Lift of Lockdown Announcement Vs Lockdown		Lift of Lockdown vs. Pre-lockdown	
**Measures**	IRR (95%CI)	I^2	IRR (95%CI)	I^2	IRR (95%CI)	I^2
Number of deaths	2.30(1.92-2.75)	55.1	0.39(0.23-0.67)	92.5	0.90(0.59-1.38)	87.7
Trust-wide activity						
Number of new referrals accepted	0.68(0.58-0.79)	0.0	1.32(1.10-1.57)	0.0	0.95(0.84-1.07)	0.0
Number of discharges	0.85(0.73-0.99)	0.0	1.01(0.85-1.19)	0.0	0.87(0.77-0.99)	0.0
Inpatient care						
New admissions	0.78(0.67-0.91)	15.1	1.21(1.02-1.43)	0.0	0.92(0.79-1.06)	0.0
Discharges	0.73(0.59-0.91)	0.0	0.78(0.60-1.02)	8.6	0.54(0.44-0.67)	0.0
Inpatient caseload	0.84(0.77-0.90)	99.1	1.05(0.98-1.13)	99.1	0.88(0.82-0.94)	99.4
Inpatient caseload on MHA section	0.91(0.84-0.99)	99.3	0.99(0.90-1.09)	99.6	0.90(0.85-0.96)	98.8
AMH (community)						
F2f contacts	0.64(0.51-0.80)	0.0	0.92(0.71-1.17)	0.0	0.59(0.47-0.72)	4.3
Non-f2f contacts	1.69(1.38-2.08)	0.0	1.49(1.21-1.83)	0.0	2.50(2.12-2.94)	0.0
F2f and Non-f2f contacts	1.03(0.85-1.25)	0.0	1.11(0.90-1.37)	0.0	1.19(1.03-1.38)	0.0
Cancelled appointments or DNAs	0.63(0.50-0.79)	0.0	1.08(0.82-1.42)	9.0	0.70(0.50-0.97)	42.1
Caseload	1.02(1.01-1.03)	92.8	1.01(1.00-1.02)	95.0	1.03(1.03-1.04)	87.1
CAMHS						
F2f contacts	0.50(0.28-0.90)	73.3	1.20(0.83-1.73)	0.0	0.56(0.28-1.11)	72.4
Non-f2f contacts	2.19(1.51-3.18)	40.0	1.50(1.19-1.90)	0.0	3.11(2.07-4.67)	47.8
F2f and Non-f2f contacts	1.08(0.87-1.35)	0.0	1.22(0.95-1.56)	0.0	1.29(1.06-1.57)	0.0
Cancelled appointments or DNAs	0.65(0.51-0.83)	0.0	1.15(0.82-1.61)	20.4	0.77(0.61-0.97)	8.5
Caseload	0.98(0.96-1.00)	96.6	0.99(0.96-1.01)	98.7	0.97(0.92-1.02)	99.1
EIP						
F2f contacts	0.37(0.23-0.58)	65.3	1.50(1.01-2.23)	50.4	0.55(0.33-0.92)	78.4
Non-f2f contacts	1.69(1.18-2.43)	45.8	1.88(1.47-2.41)	0.0	2.78(2.30-3.36)	0.0
F2f and Non-f2f contacts	0.90(0.73-1.12)	0.0	1.43(1.15-1.80)	0.0	1.28(1.09-1.50)	0.0
Cancelled appointments or DNAs	0.66(0.52-0.85)	0.0	0.97(0.65-1.46)	50.1	0.60(0.35-1.04)	80.4
Caseload	1.04(1.02-1.05)	96.8	1.04(0.99-1.08)	99.6	1.07(1.02-1.12)	99.8
HTT						
F2f contacts	0.60(0.48-0.75)	90.3	1.39(1.29-1.49)	8.2	0.84(0.71-0.99)	86.4
Non-f2f contacts	1.43(1.21-1.69)	51.0	1.14(0.95-1.36)	55.7	1.57(1.19-2.07)	85.8
F2f and Non-f2f contacts	0.88(0.81-0.96)	57.5	1.25(1.16-1.34)	31.4	1.08(0.95-1.21)	80.2
Cancelled appointments or DNAs	0.78(0.64-0.94)	49.4	1.44(1.08-1.93)	66.6	1.12(0.89-1.40)	57.1
Caseload	0.68(0.55-0.84)	99.0	1.16(1.01-1.35)	98.1	0.80(0.73-0.87)	96.5
Liaison						
F2f contacts	0.61(0.53-0.70)	39.0	1.40(1.24-1.57)	7.4	0.81(0.65-1.01)	76.3
Non-f2f contacts	1.06(0.89-1.28)	0.0	1.34(0.89-2.03)	62.1	1.46(0.97-2.19)	69.3
F2f and Non-f2f contacts	0.71(0.63-0.80)	23.7	1.33(1.17-1.51)	15.4	0.91(0.75-1.11)	68.5
Cancelled appointments or DNAs	0.56(0.37-0.87)	42.0	0.73(0.39-1.34)	41.6	0.57(0.35-0.95)	33.8
Caseload	0.88(0.82-0.94)	97.5	1.08(1.02-1.14)	95.4	0.94(0.86-1.03)	98.4
OA						
F2f contacts	0.46(0.29-0.71)	61.8	1.25(0.93-1.68)	4.1	0.48(0.26-0.87)	79.1
Non-f2f contacts	1.63(1.27-2.10)	0.0	1.51(1.16-1.97)	0.0	2.65(1.91-3.66)	30.1
F2f and Non-f2f contacts	0.91(0.72-1.15)	0.0	1.28(0.99-1.65)	0.0	1.14(0.89-1.46)	12.5
Cancelled appointments or DNAs	0.67(0.51-0.88)	0.0	0.88(0.41-1.90)	75.0	0.60(0.33-1.10)	67.1
Caseload	0.95(0.93-0.97)	97.8	0.99(0.93-1.04)	99.4	0.94(0.88-1.01)	99.8

^p<0.05; *p<0.001

AMH: adult mental health service; CAMHS: child and adolescent mental health service; EIP: early intervention for psychosis service; HTT: home treatment team; OA: older adult service.

Bakolis I, *et al. BMJ Open* 2021; 11:e049721. doi: 10.1136/bmjopen-2021-049721

Supplementary Figure 1. Number of deaths per day before and after lockdown announcement for sites A, F, G and J during the period of 1st January 2020 to 31st July 2020. The vertical axis shows the daily number of deaths in the days before (orange dots) and after (red dots) the lockdown order was announced in 23rd March 2020 and after the lift (purple dots) of the lockdown order announced in 10th May. The green dots represent the number of daily deaths during the period of 1st January 2019 to 31st July 2019.

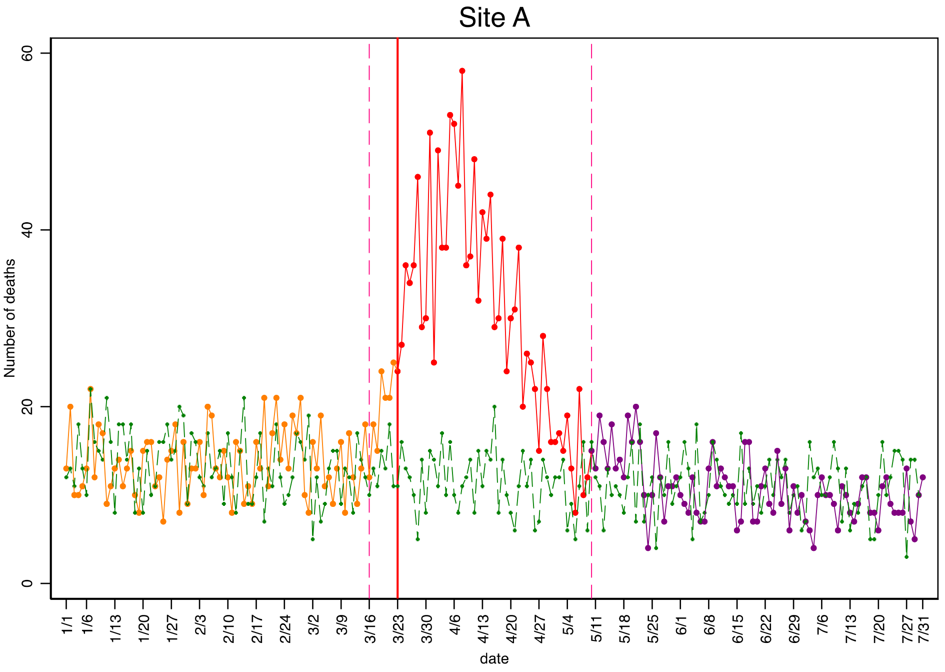

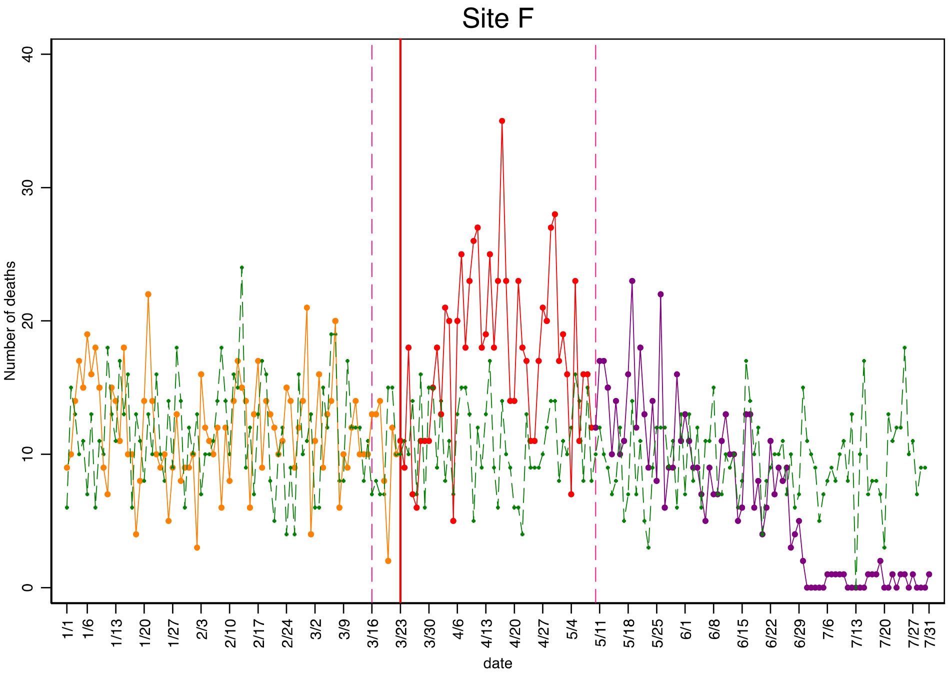

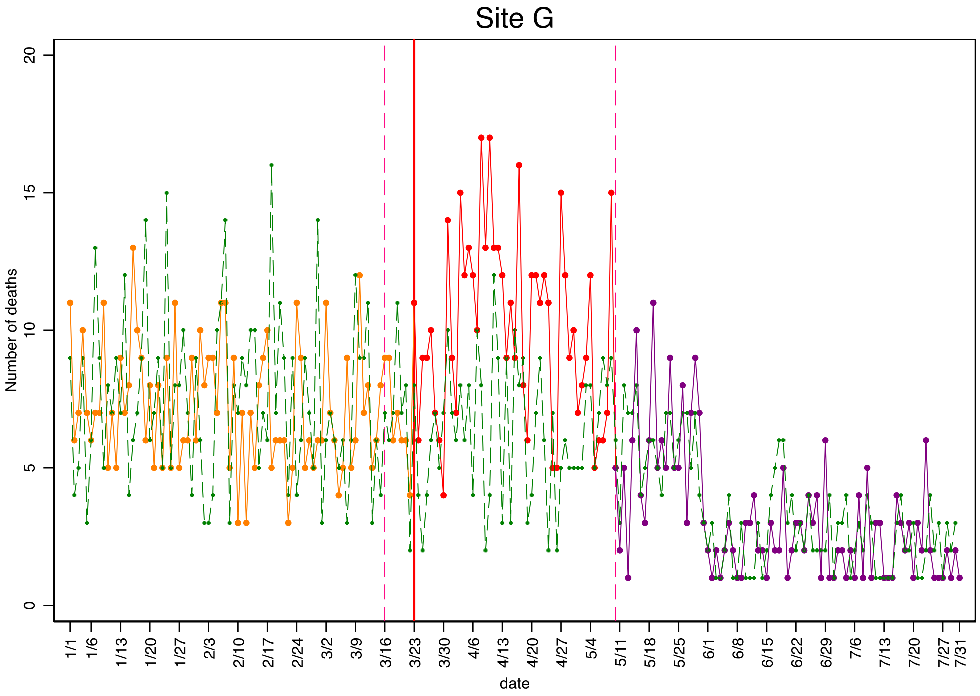
Site G
Number of deaths
date
0
5
10
15
20
1/1
1/6
1/13
1/20
1/27
2/3
2/10
2/17
2/24
3/2
3/9
3/16
3/23
3/30
4/6
4/13
4/20
4/27
5/4
5/11
5/18
5/25
6/1
6/8
6/15
6/22
6/29
7/6
7/13
7/20
7/27
7/31

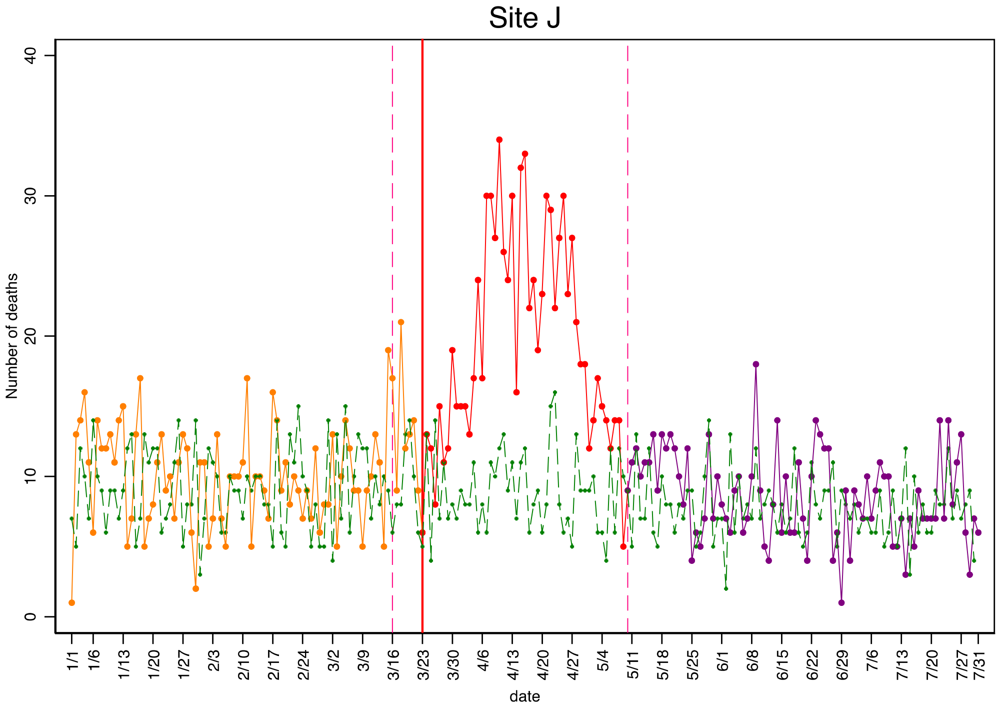
Site J
Number of deaths
date
0
10
20
30
40
1/1
1/6
1/13
1/20
1/27
2/3
2/10
2/17
2/24
3/2
3/9
3/16
3/23
3/30
4/6
4/13
4/20
4/27
5/4
5/11
5/18
5/25
6/1
6/8
6/15
6/22
6/29
7/6
7/13
7/20
7/27
7/31

Supplementary Figure 2. Number of deaths (A) and inpatient admissions (B) per week before and after lockdown announcement for site I during the period of 1st January 2020 to 5th July 2020. The vertical axis shows the daily number of deaths in the days before (orange dots) and after (red dots) the lockdown order was announced on 23rd March 2020 and after the lift (purple dots) of the lockdown order announced in 10th May. The green dots represent the number of daily deaths during the period of 1st January 2019 to 5th July 2019.

A

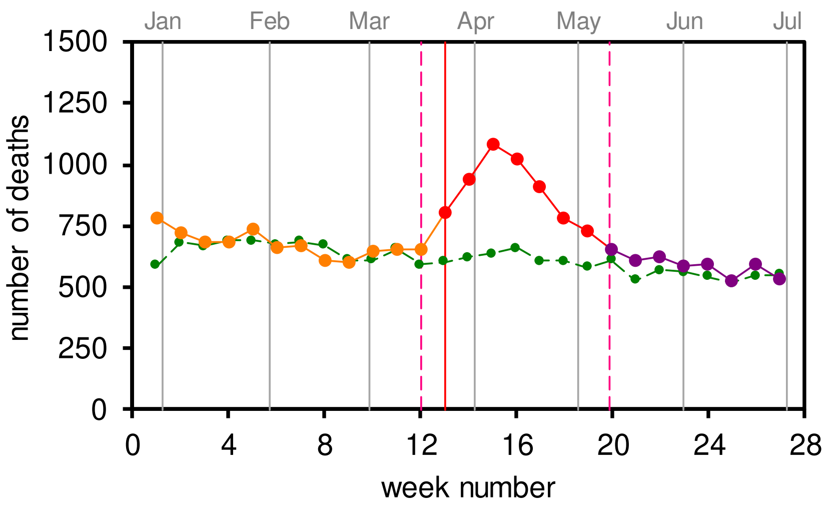

B

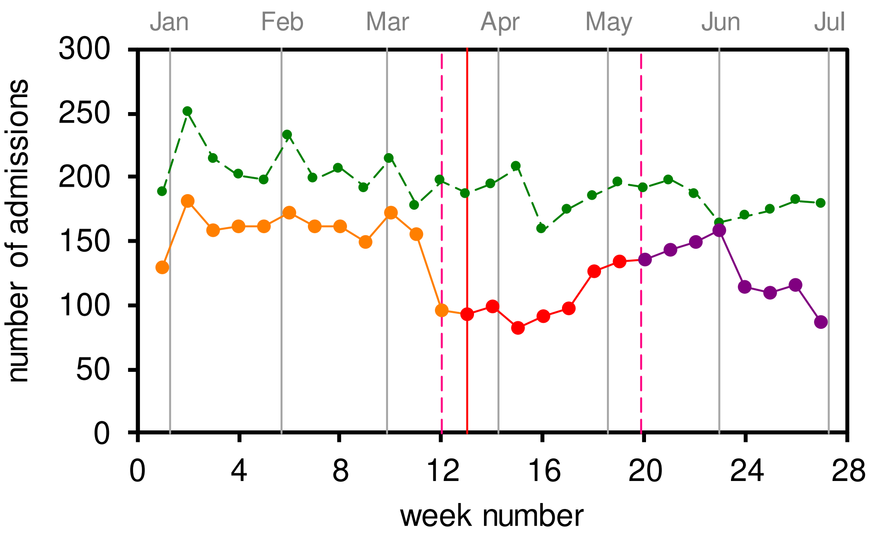

Bakolis I, *et al. BMJ Open* 2021; 11:e049721. doi: 10.1136/bmjopen-2021-049721

Supplementary Figure 3. Number of deaths per day before and after lockdown announcement for sites B, C, D, E, H and I during the period of 1st January 2020 to 31st May 2020. The vertical axis shows the daily number of deaths in the days before (orange dots) and after (red dots) the lockdown order was announced in 23rd March 2020 and after the lift (purple dots) of the lockdown order announced in 10th May. The green dots represent the number of daily deaths during the period of 1st January 2019 to 31st May 2019.

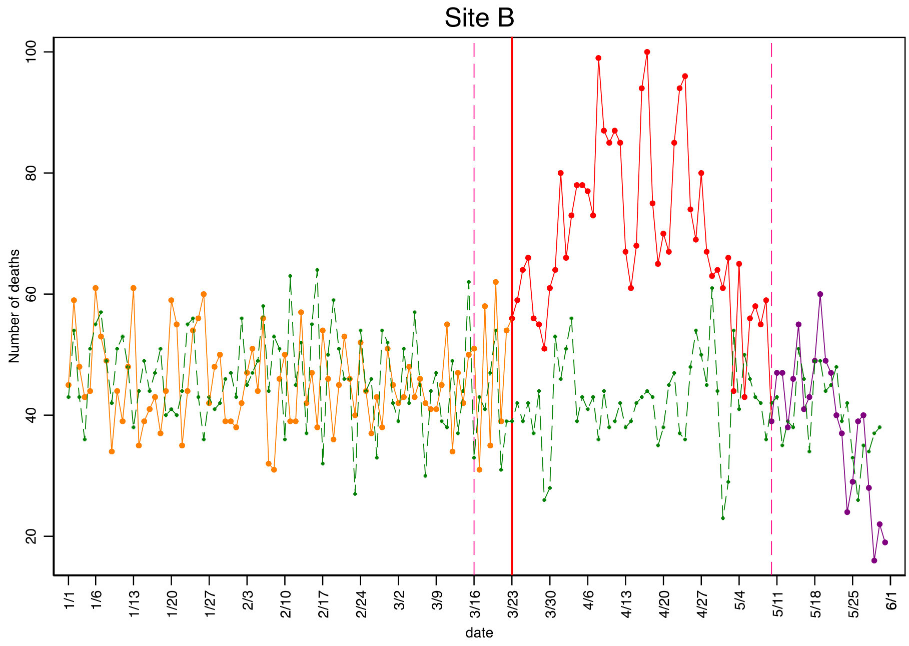

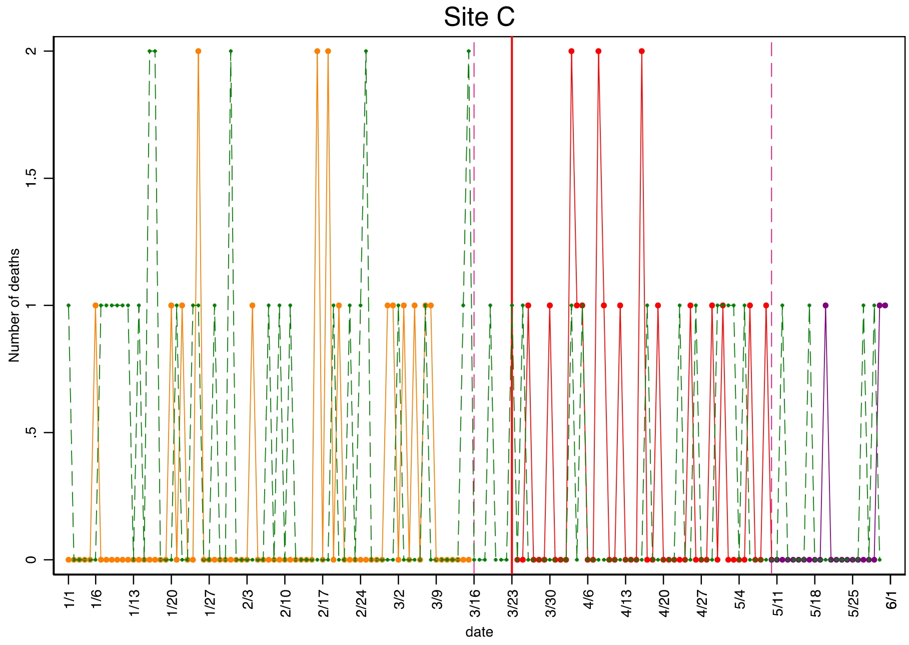

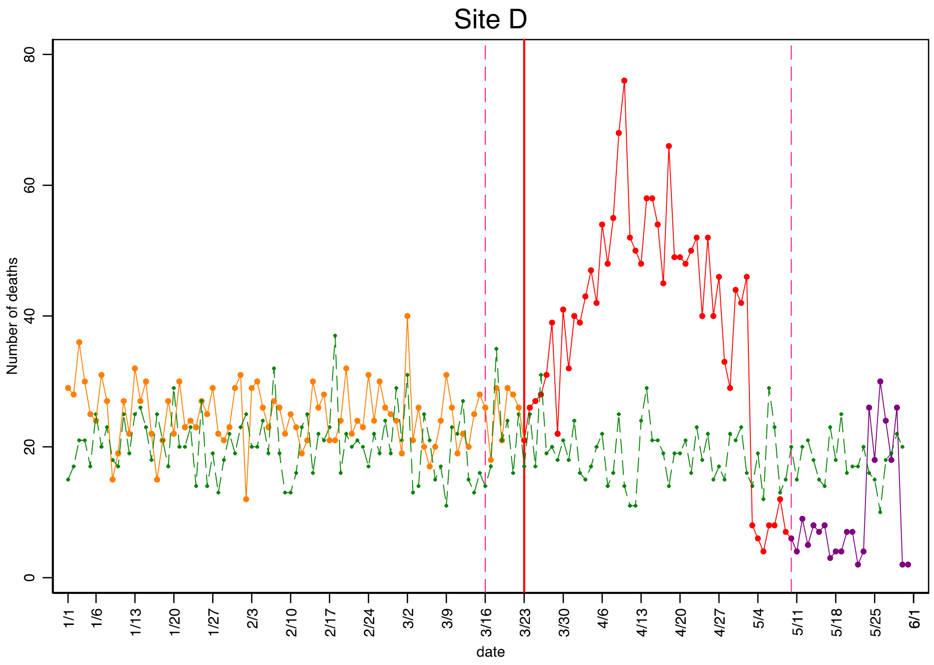
Site D
Number of deaths
0
20
40
60
80
1/1
1/6
1/13
1/20
1/27
2/3
2/10
2/17
2/24
3/2
3/9
3/16
3/23
3/30
4/6
4/13
4/20
4/27
5/4
5/11
5/18
5/25
6/1
date

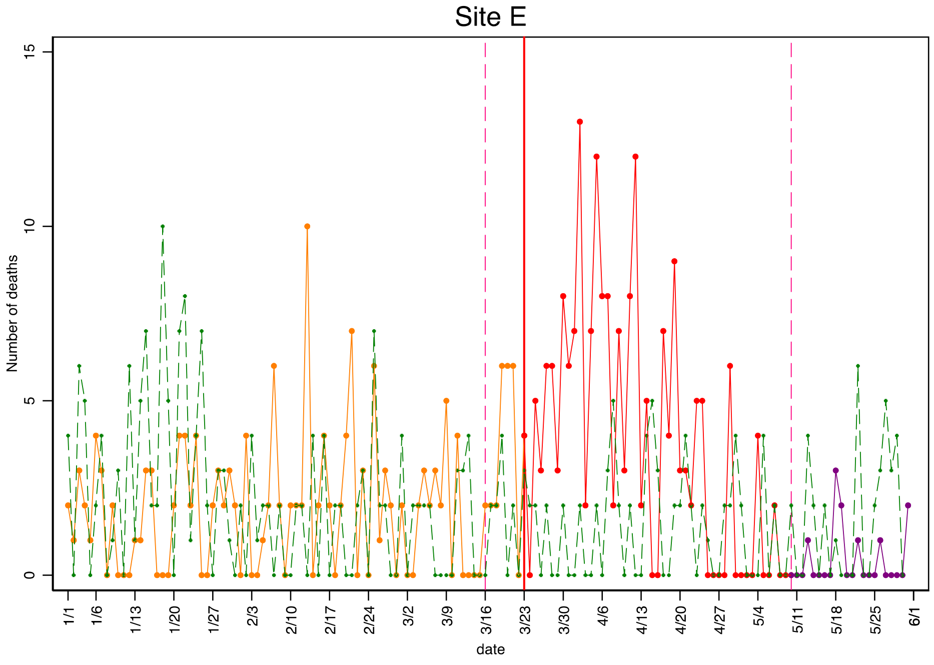
Site E
Number of deaths
0
5
10
15
1/1
1/6
1/13
1/20
1/27
2/3
2/10
2/17
2/24
3/2
3/9
3/16
3/23
3/30
4/6
4/13
4/20
4/27
5/4
5/11
5/18
5/25
6/1
date

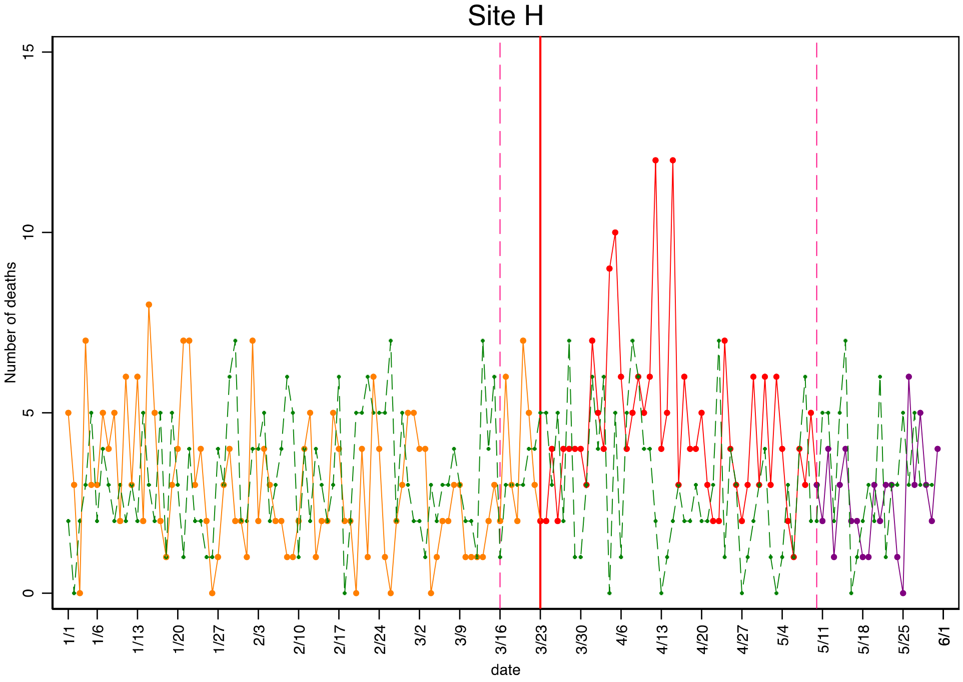

Bakolis I, *et al. BMJ Open* 2021; 11:e049721. doi: 10.1136/bmjopen-2021-049721

Supplementary Figure 4. Number of inpatient admissions per day before and after lockdown announcement for sites A, F and J during the period of 1st January 2020 to 31st July 2020. The vertical axis shows the daily number of deaths in the days before (orange dots) and after (red dots) the lockdown order was announced on 23rd March and after the lift (purple dots) of the lockdown order announced in 10th May. The green dots represent the number of daily deaths during the period of 1st January 2019 to 31st July 2019.

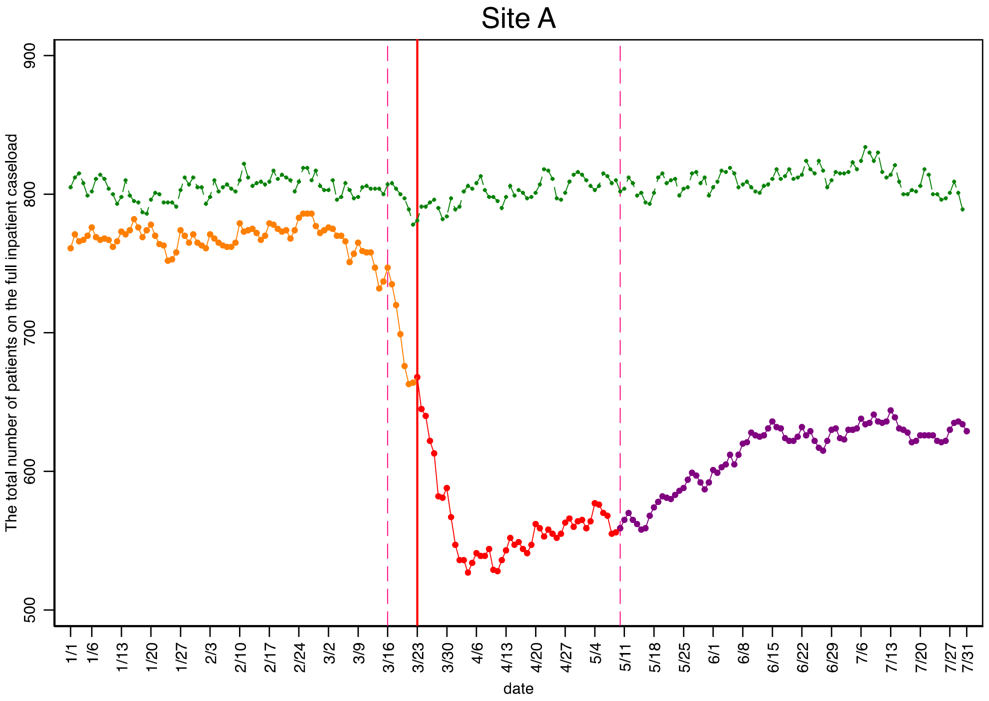

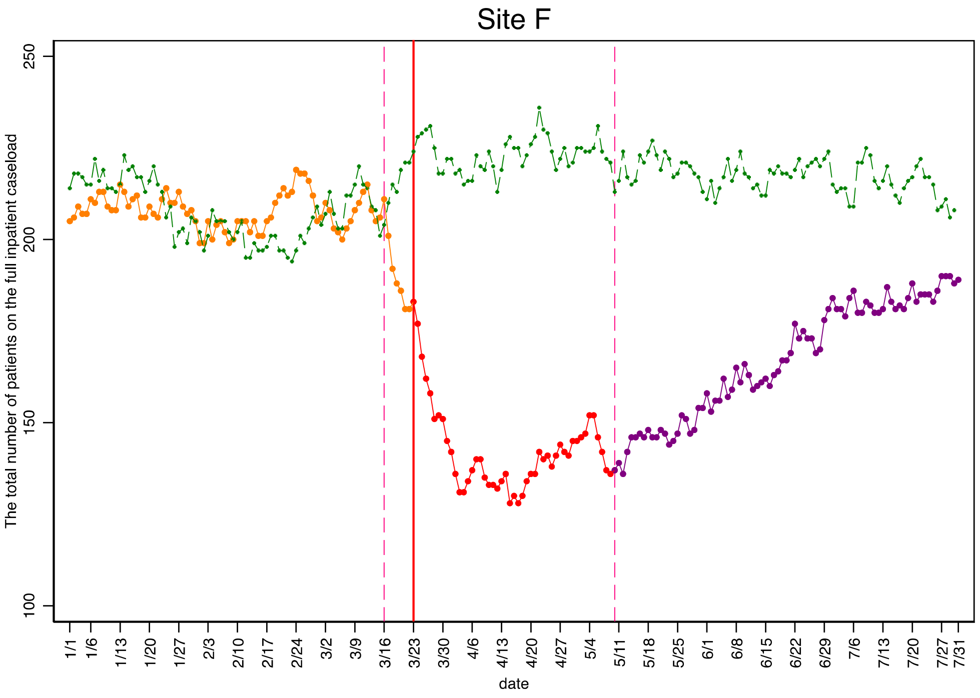

Bakolis I, *et al. BMJ Open* 2021; 11:e049721. doi: 10.1136/bmjopen-2021-049721

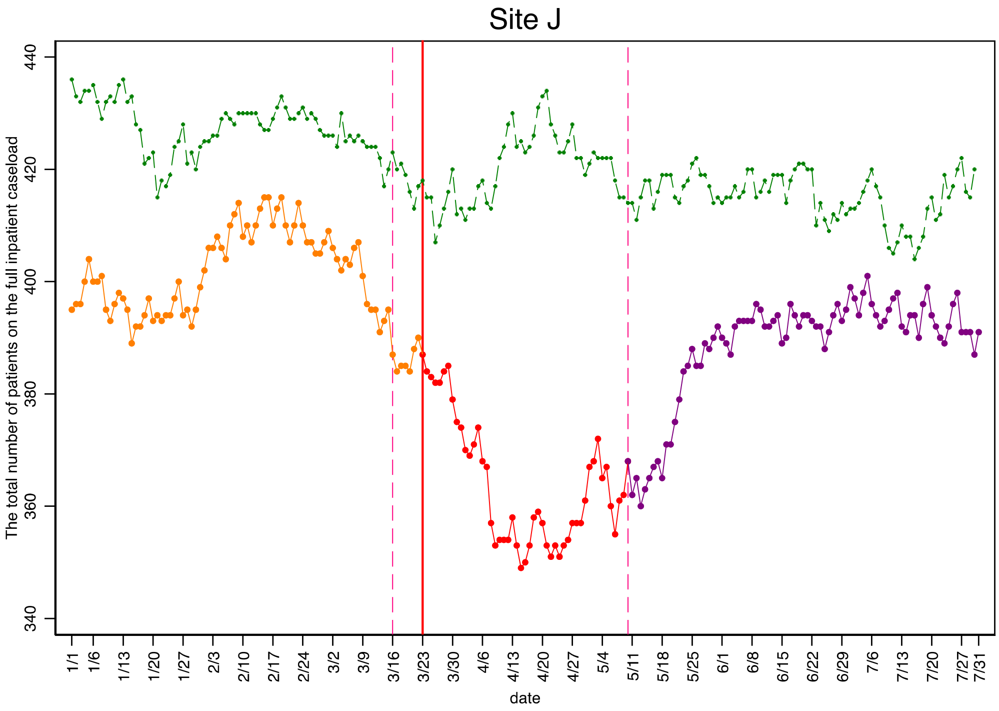
Site J
The total number of patients on the full inpatient caseload
440
420
400
380
360
340
1/1
1/6
1/13
1/20
1/27
2/3
2/10
2/17
2/24
3/2
3/9
3/16
3/23
3/30
4/6
4/13
4/20
4/27
5/4
5/11
5/18
5/25
6/1
6/8
6/15
6/22
6/29
7/6
7/13
7/20
7/27
7/31
date

Supplementary Figure 5. Number of deaths per day before and after lockdown announcement for sites A, B, D, F, and J during the period of 1st January 2020 to 31st May 2020. The vertical axis shows the daily number of deaths in the days before (orange dots) and after (red dots) the lockdown order was announced on 23rd March and after the lift (purple dots) of the lockdown order announced on 10th May. The green dots represent the number of daily deaths during the period of 1st January 2019 to 31st May 2019. The lines represent the predicted values of our fitted model.

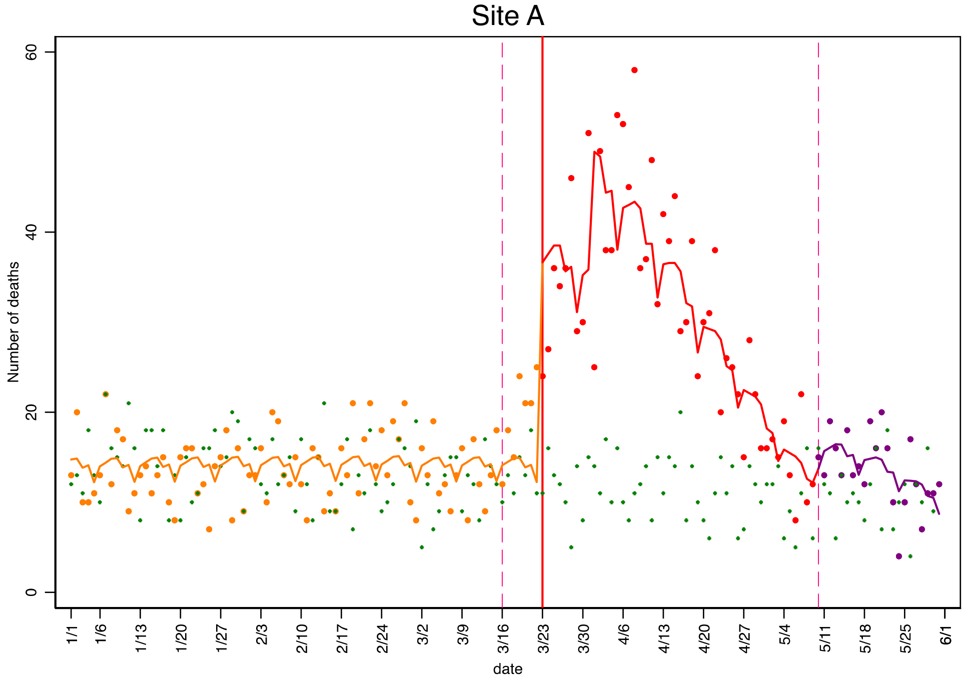

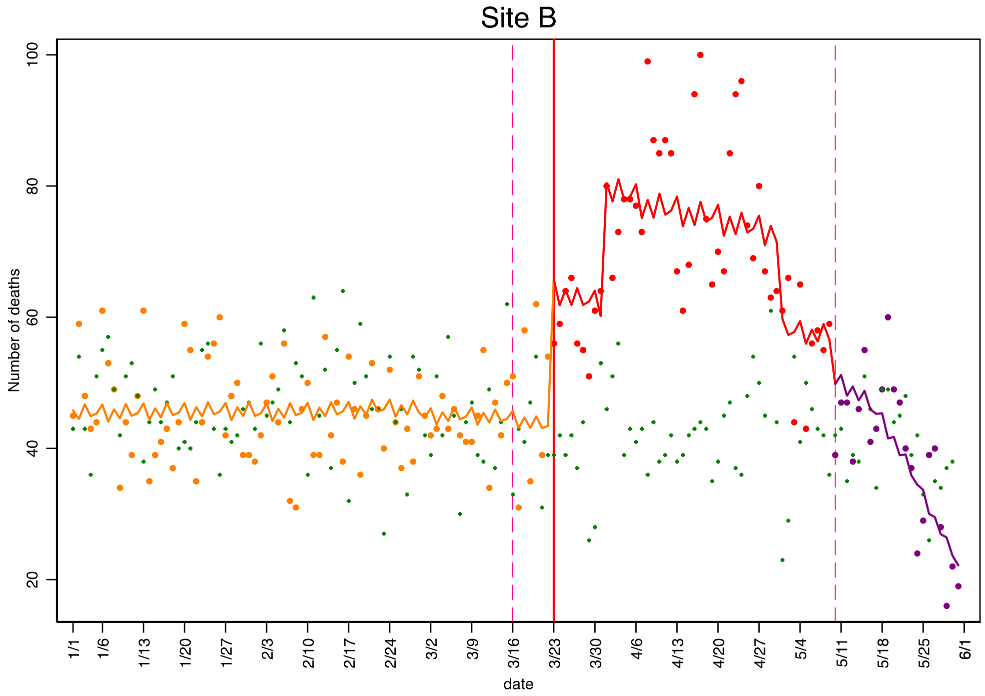

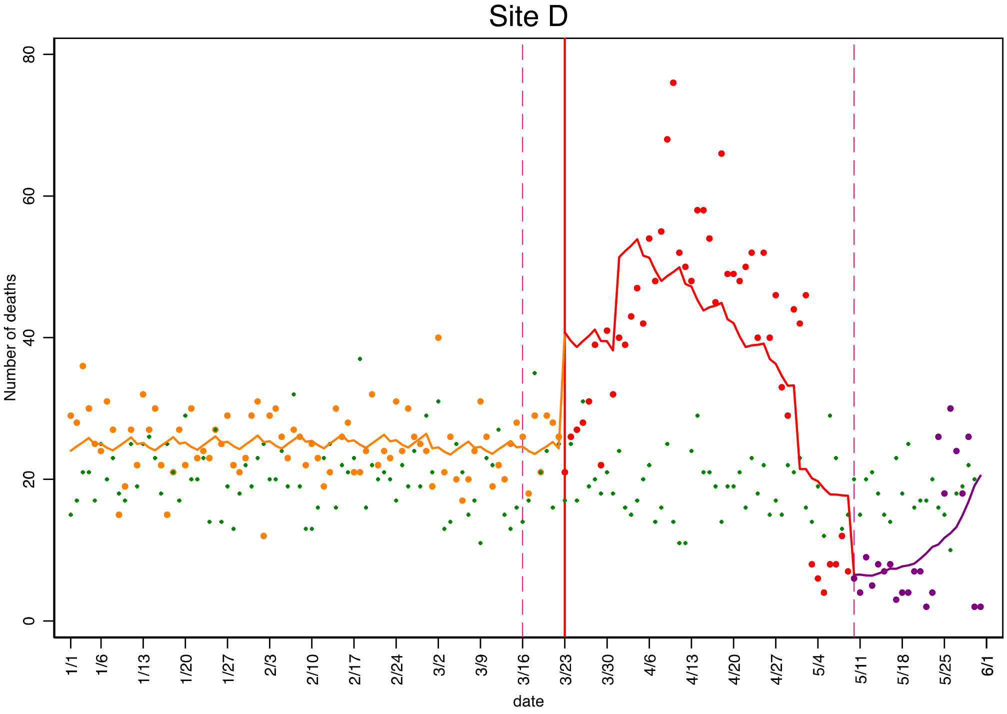

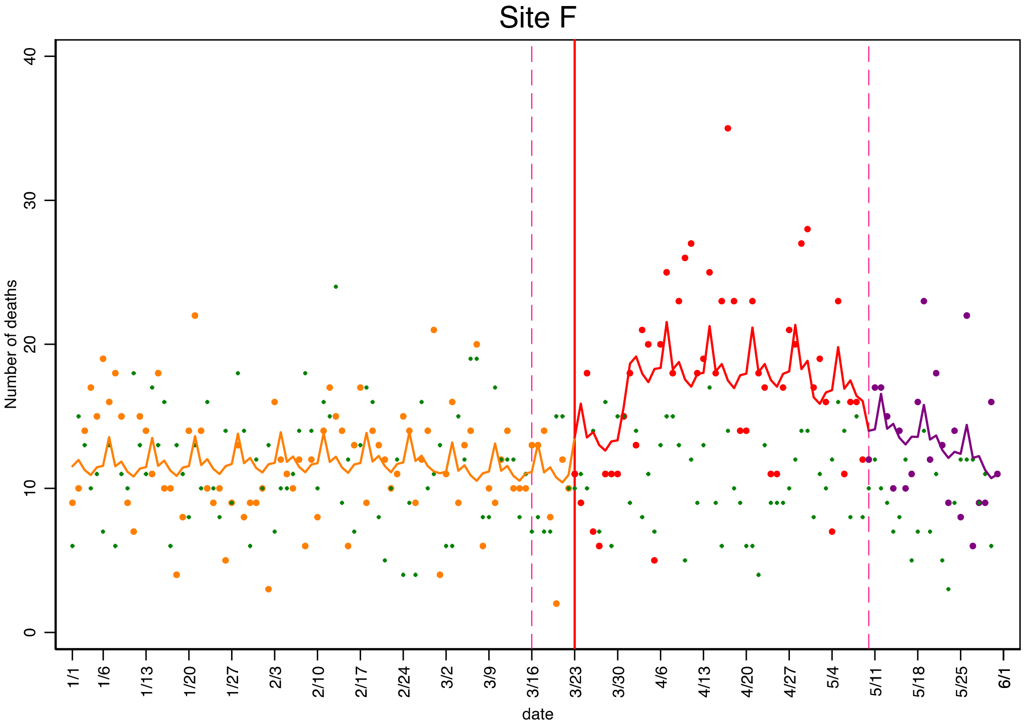

Bakolis I, *et al. BMJ Open* 2021; 11:e049721. doi: 10.1136/bmjopen-2021-049721

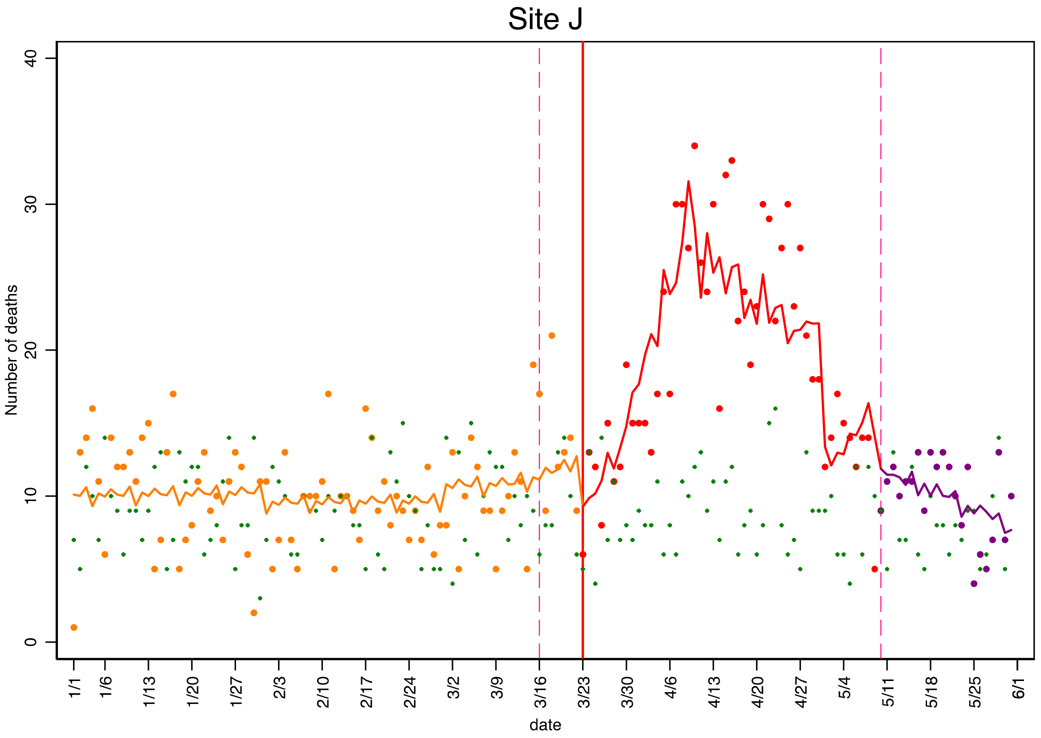

Bakolis I, *et al. BMJ Open* 2021; 11:e049721. doi: 10.1136/bmjopen-2021-049721
