# Supplementary material for: Changes in daily mental health service use and mortality at the commencement and lifting of COVID-19 ‘lockdown’ policy in 10 UK sites: a regression discontinuity in time design
Source: BMJ Open. 2021 May 26;11(5):e049721. doi: 10.1136/bmjopen-2021-049721 (PMC8159668; doi:10.1136/bmjopen-2021-049721)
Supplement: Author's manuscript [file bmjopen-2021-049721.draft_revisions.pdf]

BMJ Open is committed to open peer review. As part of this commitment we make the peer review history of every article we publish publicly available.

When an article is published we post the peer reviewers' comments and the authors' responses online. We also post the versions of the paper that were used during peer review. These are the versions that the peer review comments apply to.

The versions of the paper that follow are the versions that were submitted during the peer review process. They are not the versions of record or the final published versions. They should not be cited or distributed as the published version of this manuscript.

BMJ Open is an open access journal and the full, final, typeset and author-corrected version of record of the manuscript is available on our site with no access controls, subscription charges or pay-per-view fees (http://bmjopen.bmj.com).

If you have any questions on BMJ Open's open peer review process please email info.bmjopen@bmj.com

BMJ Open

Changes in daily mental health service use and mortality at the commencement and lifting of COVID-19 'lockdown' policy in ten UK sites

Journal:	*BMJ Open*
Manuscript ID	bmjopen-2021-049721
Article Type:	Original research
Date Submitted by the Author:	03-Feb-2021
Complete List of Authors:	Bakolis, Ioannis; King's College London Institute of Psychiatry Psychology and Neuroscience<br>Stewart, Robert; King's College London Institute of Psychiatry Psychology and Neuroscience; South London and Maudsley NHS Foundation Trust<br>Baldwin, David ; Southern Health NHS Foundation Trust; University of Southampton Faculty of Medicine<br>Beenstock, Jane; Lancashire and South Cumbria NHS Foundation Trust<br>Bibby, Paul; Lancashire and South Cumbria NHS Foundation Trust<br>Broadbent, Matthew; South London and Maudsley NHS Foundation Trust<br>Cardinal, Rudolf; University of Cambridge School of Clinical Medicine, Department of Psychiatry; Cambridgeshire and Peterborough NHS Foundation Trust, Liaison Psychiatry Service<br>Chen, Shanquan; University of Cambridge School of Clinical Medicine, Department of Psychiatry<br>Chinnasamy, Karthik; Camden and Islington NHS Foundation Trust<br>Cipriani, Andrea; University of Oxford Medical Sciences Division, Department of Psychiatry; Oxford Health NHS Foundation Trust<br>Douglas, Simon; Cumbria Northumberland Tyne and Wear NHS Foundation Trust<br>Horner, Philip; Lancashire and South Cumbria NHS Foundation Trust<br>Jackson, Caroline A.; University of Edinburgh Division of Medical and Radiological Sciences, Usher Institute of Population Health Sciences & Informatics<br>John, Ann; Swansea University Medical School, Population Data Science<br>Joyce, Dan W.; University of Oxford, Department of Psychiatry<br>Lee, Sze; Swansea University Medical School, Population Data Science<br>Lewis, Jonathan; Cambridgeshire and Peterborough NHS Foundation Trust<br>McIntosh, Andrew; University of Edinburgh Division of Medical and Radiological Sciences, Division of Psychiatry<br>Nixon, Neil; University of Nottingham Faculty of Medicine and Health Sciences, Division of Psychiatry and Applied Psychology; Nottinghamshire Healthcare NHS Foundation Trust<br>Osborn, David; University College London Faculty of Medical Sciences, Division of Psychiatry<br>Phiri, Peter; Southern Health NHS Foundation Trust<br>Rathod, Shanaya; Southern Health NHS Foundation Trust<br>Smith, Tanya; Oxford Health NHS Foundation Trust

Sokal, Rachel; Nottinghamshire Healthcare NHS Foundation Trust
Waller, Rob; Lothian Primary Care NHS Trust
Landau, Sabine; King's College London Institute of Psychiatry Psychology and Neuroscience

Keywords: COVID-19, MENTAL HEALTH, Organisation of health services < HEALTH SERVICES ADMINISTRATION & MANAGEMENT, Adult psychiatry < PSYCHIATRY, Old age psychiatry < PSYCHIATRY

SCHOLARONE™
Manuscripts

I, the Submitting Author has the right to grant and does grant on behalf of all authors of the Work (as defined in the below author licence), an exclusive licence and/or a non-exclusive licence for contributions from authors who are: i) UK Crown employees; ii) where BMJ has agreed a CC-BY licence shall apply, and/or iii) in accordance with the terms applicable for US Federal Government officers or employees acting as part of their official duties; on a worldwide, perpetual, irrevocable, royalty-free basis to BMJ Publishing Group Ltd ("BMJ") its licensees and where the relevant Journal is co-owned by BMJ to the co-owners of the Journal, to publish the Work in this journal and any other BMJ products and to exploit all rights, as set out in our licence.

The Submitting Author accepts and understands that any supply made under these terms is made by BMJ to the Submitting Author unless you are acting as an employee on behalf of your employer or a postgraduate student of an affiliated institution which is paying any applicable article publishing charge ("APC") for Open Access articles. Where the Submitting Author wishes to make the Work available on an Open Access basis (and intends to pay the relevant APC), the terms of reuse of such Open Access shall be governed by a Creative Commons licence – details of these licences and which Creative Commons licence will apply to this Work are set out in our licence referred to above.

Other than as permitted in any relevant BMJ Author's Self Archiving Policies, I confirm this Work has not been accepted for publication elsewhere, is not being considered for publication elsewhere and does not duplicate material already published. I confirm all authors consent to publication of this Work and authorise the granting of this licence.

For peer review only

Changes in daily mental health service use and mortality at the commencement and lifting of COVID-19 'lockdown' policy in ten UK sites

Ioannis Bakolis[1]*, Robert Stewart[2,3]*, David S. Baldwin[4], Jane Beenstock[5], Paul Bibby[5], Matthew Broadbent[3], Rudolf Cardinal[6,7], Shanquan Chen[6], Karthik Chinnasamy[8], Andrea Cipriani[9,10], Simon Douglas[11], Philip Horner[5], Caroline Jackson[12], Ann John[13], Dan W. Joyce[9], Sze Chim Lee[13], Jonathan Lewis[7], Andrew McIntosh[14], Neil Nixon[15,16], David P.J. Osborn[17], Peter Phiri[18], Shanaya Rathod[18], Tanya Smith[10], Rachel Sokal[16], Rob Waller[19], Sabine Landau[20].

*Equal contributions to authorship

[1]Departments of Biostatistics and Health Informatics & Health Services and Population Research, Institute of Psychiatry, Psychology and Neuroscience, King's College London, London, UK.

[2]Department of Psychological Medicine, Institute of Psychiatry, Psychology and Neuroscience, King's College London, London, UK.

[3]South London and Maudsley NHS Foundation Trust, London, UK.

[4]Clinical and Experimental Sciences (CNS and Psychiatry), Faculty of Medicine, University of Southampton, Southampton, UK.

[5]Lancashire & South Cumbria NHS Foundation Trust, Preston, UK.

[6]Department of Psychiatry, University of Cambridge, Cambridge, UK.

[7]Cambridgeshire & Peterborough NHS Foundation Trust, Cambridge, UK.

[8]Camden and Islington NHS Foundation Trust, London, UK.

[9]Department of Psychiatry, University of Oxford, Oxford, UK.

[10]Oxford Health NHS Foundation Trust, Oxford, UK.

[11]Cumbria, Northumberland, Tyne & Wear NHS Foundation Trust, Newcastle upon Tyne, UK.

[12]Usher Institute of Population Health Sciences & Informatics, University of Edinburgh, Edinburgh, UK.

[13]Population Data Science, Swansea University Medical School, Swansea, UK.

[14]University of Edinburgh, Centre for Clinical Brain Sciences, Edinburgh, UK.

For peer review only - http://bmjopen.bmj.com/site/about/guidelines.xhtml

For peer review only

[15]Division of Psychiatry and Applied Psychology, School of Medicine, University of Nottingham, Nottingham, UK.

[16]Nottinghamshire Healthcare NHS Foundation Trust, Nottingham, UK.

[17]Division of Psychiatry, University College London, London, UK.

[18]Southern Health NHS Foundation Trust, Southampton, UK.

[19]Lothian Primary Care NHS Trust, Edinburgh, UK.

[20]Department of Biostatistics and Health Informatics, Institute of Psychiatry, Psychology and Neuroscience, King's College London, London, UK.

Corresponding author: Prof. Robert Stewart, Department of Psychological Medicine, Institute of Psychiatry, Psychology and Neuroscience, King's College London, De Crespigny Park, London SE5 8AF, UK. Email: r.stewart@iop.kcl.ac.uk

For peer review only

Funding statement

No specific funding was received for this study. Regarding relevant background infrastructure funding, RS is part-funded by: i) the NIHR Biomedical Research Centre at the South London and Maudsley NHS Foundation Trust and King's College London; ii) a Medical Research Council (MRC) Mental Health Data Pathfinder Award to King's College London; iii) an NIHR Senior Investigator Award; iv) the NIHR Applied Research Collaboration South London (NIHR ARC South London) at King's College Hospital NHS Foundation Trust. IB and SL are supported by the NIHR Biomedical Research Centre at South London and Maudsley NHS Foundation Trust and King's College London, and by the NIHR Applied Research Collaboration South London (NIHR ARC South London) at King's College Hospital NHS Foundation Trust. RNC's research is funded by the MRC (grant MC_PC_17213), and the NIHR Cambridge Biomedical Research Centre. AC is supported by the NIHR Oxford Cognitive Health Clinical Research Facility, by an NIHR Research Professorship (grant RP-2017-08-ST2-006), by the NIHR Oxford and Thames Valley Applied Research Collaboration, and by the NIHR Oxford Health Biomedical Research Centre (grant BRC-1215-20005). AJ is part funded by MQ ADP, and a Medical Research Council (MRC) Mental Health Data Pathfinder Award to Swansea University. AJ and SCL are part funded by Health and Care Research Wales National Centre for Mental Health. DO is supported by the NIHR Biomedical Research Centre (BRC) at University College London Hospitals (UCLH), and by the National Institute for Health Research ARC North Thames. Additional infrastructure funding was provided by the Medical Research Council (MRC)

For peer review only - http://bmjopen.bmj.com/site/about/guidelines.xhtml

Mental Health Data Pathfinder Award to University of Edinburgh (MC_PC_17209). The collaboration providing Wales data was led by the Swansea University Health Data Research UK team under the direction of the Welsh Government Technical Advisory Cell (TAC) and includes the following groups and organisations: the Secure Anonymised Information Linkage (SAIL) Databank, Administrative Data Research (ADR) Wales, NHS Wales Informatics Service (NWIS), Public Health Wales, NHS Shared Services and the Welsh Ambulance Service Trust (WAST), MRC grant MR/V028367. This study was additionally supported by the NIHR Mental Health Translational Research Collaboration (MH-TRC). The views expressed are those of the authors and not necessarily those of the UK National Health Service, the NIHR, or the UK Department of Health.

Declaration of interests of interests statement

RNC consults for Campden Instruments Ltd. and receives royalties from Cambridge University Press, Cambridge Enterprise, and Routledge. AC reports research funding from Angelini Pharma and personal fees from INCiPiT, CARIPLO Foundation and Angelini Pharma. AM reports research funding from The Sackler Trust and personal fees from Janssen and Illumina. PP reports research funding from NovoNordisk, and royalties from John Wiley & Sons. SR reports education support from Janssen, Otsuka and Lundbeck. RS reports research support from Janssen, GSK and Takeda, and royalties from Oxford University Press.

Acknowledgements

This work uses data provided by patients and collected by the NHS as part of their care and support. We would also like to acknowledge all data providers who make anonymised data available for research. We wish to acknowledge the collaborative partnership that enabled acquisition and access to the de-identified data, which led to this output, and which was facilitated by MQ and the National Institute for Health Research (NIHR) Mental Health Translational Research Collaborative (MH-TRC).

Author contributions

The study was initially formulated by RS, IB and SL, and further formulation and design were developed with the input of all authors. All authors were involved in the coordination and/or implementation of data extractions for at least one of the constituent sites. Data analyses were carried out by IB, SL, AJ and SCL. The manuscript was prepared by IB and

For peer review only

RS with input from all authors, the final version of which has been seen and approved by all authors.

Patient and Public Involvement statement

The research data resources and infrastructures supporting this study receive extensive patient and public input. The specific analyses reported here did not include any specific involvement in the design, conduct or reporting.

Manuscript word count: 3795

For peer review only

ABSTRACT

Objectives: To investigate the impact of the COVID-19 pandemic on mental health services.

Design: A time series analysis of daily service-level activity

Setting and participants: Mental healthcare data were extracted from ten UK providers

Outcome measures: Daily (weekly for one site) deaths, referrals and discharges, inpatient care (admissions, discharges, caseloads), and community services (face-to-face/non-face-to-face contacts, caseloads): Adult, Older Adult, and Child/Adolescent Mental Health; Early Intervention in Psychosis; Home Treatment Teams; Liaison/A&E. Data were extracted for 2019 and 2020 to 31st May for all sites, supplemented to 31st July for four sites. Changes around the commencement and lifting of the UK national 'lockdown' (March 23rd and May 10th respectively) were estimated using a regression discontinuity design with a difference-in-difference approach generating incidence rate ratios (IRRs), meta-analysed across sites.

Results: Pooled estimates for the lockdown transition showed increased daily deaths (IRR 2.31, 95% CI 1.86-2.87), reduced referrals (0.62, 0.55-0.70), and reduced inpatient admissions (0.75, 0.67-0.83) and caseloads (0.85, 0.79-0.91). All community services saw shifts from face-to-face to non-face-to-face contacts, but varied in caseload changes. Lift-of-lockdown was associated with reduced deaths (0.42, 0.27-0.66), increased referrals (1.36, 1.15-1.60), and increased inpatient admissions (1.21, 1.04-1.42) and caseloads (1.06, 1.00-1.12). Trust-wide activity, inpatient care and community services did not return to pre-lockdown levels after lift-of-lockdown, while number of deaths did. Between-site heterogeneity most often indicated variation in size rather than direction of effect.

Conclusions: Mental health service delivery underwent sizeable changes during the first national lockdown, with as-yet unknown and unevaluated consequences.

Keywords: COVID-19; mental healthcare; electronic health records; inpatient mental health; home treatment team; community mental health team; child and adolescent mental health; older adult mental health; liaison psychiatry.

For peer review only - http://bmjopen.bmj.com/site/about/guidelines.xhtml

Strengths and limitations of this study

- The data were obtained from a large number of mental health providers covering an extensive and varied geography within the UK: we believe, the most extensive multi-site evaluation to date
- Despite this, data are catchment-specific and would require further replication to clarify national and international generalisability
- Data are combined across a number of potentially diverse services for each site, with no attempt to investigate within-site heterogeneity.
- The changes described here evaluated the first wave of the COVID-19 pandemic and accompanying 'lockdown' policy in the UK and cannot necessarily be extrapolated to further pandemic waves or lockdown periods

For peer review only

INTRODUCTION

The 'first wave' of the COVID-19 pandemic affected healthcare sectors, not only through the virus' direct effects on communities and healthcare staff, but also from the national public health policies enacted to reduce spread (1). Mental health (MH) care faced a range of challenges in many countries, including the heightened vulnerability of its patient populations (e.g. through cardiovascular and respiratory disorders), already-reduced life expectancy (2), and problems in accessing healthcare (3, 4). Services had to be radically reconfigured to manage suspected or confirmed COVID-19 infections in inpatient and outpatient settings, staff sickness or self-isolation, the need to minimise face-to-face contacts, and the imperative to accommodate increasing pressures on acute medical care from cases of viral pneumonia (5). In turn, these changes were accompanied by the (as yet unknown) impacts of 'social distancing' on already isolated or otherwise vulnerable populations, and of economic constraints on already impoverished and disadvantaged communities (6, 7). This indicates a pressing need for research assessing the impact of the pandemic on mental health service demand and uptake (8).

Published data are emerging on MH outcomes in community samples (9) and specific groups such as healthcare workers (10), as well as on infection rates in MH inpatients (11), and staff concerns about MH provision (12). However, there have been few studies to date quantifying changes in mental healthcare activity (5). Single-site reports from UK services have highlighted falls in activity following the March 23rd national lockdown, followed by increased demand for some services; with shifts from face-to-face to virtual consultations, and a rise in mortality (13-16). Drawing on a network of providers, we sought to determine the level and heterogeneity of such mental healthcare changes across multiple national sites using a quasi-experimental, regression discontinuity design in time.

For peer review only

METHODS

Data sources

Early in the first UK lockdown, we enquired of several MH care providers as to the feasibility of a relatively short-notice extraction of service-level data according to a standardised protocol. Sites were sourced initially from the NIHR Mental Health Translational Research Collaboration, a network of MH services with NIHR or equivalent funding. MH Trusts in England provide all NHS specialist MH care to defined geographic catchment areas. Information from these was supplemented by further extractions of available mental healthcare data from Scotland and Wales service providers.

The following English Trusts were able to participate: i) Cambridgeshire and Peterborough Foundation Trust, serving the Combined Authority of Cambridgeshire and Peterborough, around 860,000 people; ii) Camden & Islington Foundation Trust, serving the Camden and Islington boroughs of north London, around 500,000 people; iii) Cumbria, Northumberland, Tyne & Wear Foundation Trust, based in Newcastle and covering more than 70 sites across North Cumbria, Northumberland and Tyne and Wear, a population of around 2 million; iv) Lancashire & South Cumbria Foundation Trust, serving Lancashire and South Cumbria, population 1.8 million; v) Oxford Health Foundation Trust, serving Oxfordshire, Buckinghamshire, Wiltshire, Milton Keynes, Swindon, Bath and North East Somerset, a population of around 3 million; vi) Nottinghamshire Healthcare Trust, serving City and County of Nottinghamshire, around 1.2 million people; vii) South London and Maudsley Foundation Trust, serving Croydon, Lambeth, Lewisham, and Southwark boroughs of south London, a population of around 1.3 million; viii) Southern Health Foundation Trust, serving Hampshire (excluding Portsmouth City), a population of around 1.3 million. Additional data were made available from NHS Lothian, a provider of all health services to a population of 850,000 in and around Edinburgh, including all physical and MH services; and from the Secure Anonymised Information Linkage (SAIL) databank which contains anonymised data on all health services provided to the population of Wales, around 3 million.

All data were extracted at service level, with no individual patient-level extractions. The authors assert that all procedures contributing to this work comply with the ethical standards of the relevant national and institutional committees on human experimentation and with the Helsinki Declaration of 1975, as revised in 2008. The extractions were carried out as service

For peer review only - http://bmjopen.bmj.com/site/about/guidelines.xhtml

evaluations (http://www.hra-decisiontools.org.uk/research/) with appropriate local information governance approval. Those for Cambridge and Peterborough, and South London and Maudsley sites used CRATE and CRIS data platforms respectively which have been pre-approved for both service evaluation and research purposes (REC references 17/EE/0442 and 18/SC/0372 respectively). Those for Wales sites received Information Governance Research Panel approval (SAIL 0911). Sites were de-identified prior to analysis and are referred to subsequently as Sites A-J.

Nine of the ten sites extracted daily activity data from 1st January 2020 to 31st May 2020 and 2019; extractions took place in June-July 2020. Four of the ten sites were subsequently able to provide extensions of daily data up to 31st July 2020, extracted in August-September 2020. One site extracted (in September 2020) all activity data on a weekly level from 1st January 2020 to 5th July 2020 in addition to 2019.

Mental health service activity and mortality variables

Where possible, sites extracted the following data for each day:

1. Site activity: number of new referrals accepted; number of discharges from services.
2. MH inpatient services: number of new admissions; number of discharges; daily inpatient caseload (total and caseload detained under the Mental Health Act [MHA]) section for all age groups).
3. Community services. For each service, the number of face-to-face (f2f) contacts, non-face-to-face (non-f2f) contacts, total contacts (sum of f2f and non-f2f), cancelled appointments or non-attendance (DNA), and daily caseload (patients with an active referral to that service). These were extracted for each of the following community service types:
    a. Adult Mental Health (AMH; community MH teams)
    b. Child and Adolescent Mental Health Services (CAMHS)
    c. Early Intervention in Psychosis (EIP)
    d. Home Treatment Team (HTT) – also known as crisis resolution teams which provide the option for at least daily reviews as an alternative to inpatient care.
    e. Liaison psychiatry services, including services provided to emergency departments and settings created as alternatives to emergency departments during the pandemic.
    f. Older Adults (OA) MH services.

For peer review only - http://bmjopen.bmj.com/site/about/guidelines.xhtml

Daily mortality was extracted as recorded within clinical systems from routine data supplied to sites from national sources. For sites A, B, E, F, H and J, deaths recorded on the clinical system represent those for all past service contacts regardless of whether they are a current patient at the time of death (for 90% of the catchment served by site H). Sites D and G ascertain deaths for all current patients at the time of death along with selected discharged patients (up to 6 months post-discharge for Site G). For site C, deaths were restricted to those on MH inpatient wards at the time of death. For Site I, weekly deaths and weekly inpatient admissions/discharges were extracted where mental health was recorded as the primary diagnosis.

Statistical analysis

To describe changes in the above measures we used two time points coinciding with the commencement and lifting of the national lockdown: 23rd March 2020 and 10th May 2020 respectively. To estimate these, we deployed a regression discontinuity design in time (RDiT) (17) combined with a difference-in difference (DiD) approach to estimation, comparing measures before and after the lockdown announcement in 2020 to those before and after the same date in 2019, thus adjusting for potential seasonal changes. As similar analysis was conducted for the lift-of-lockdown announcement.

For analytical purposes, we divided the sample into two cohort periods (2019, 2020). The parameters of interest were the effects of (a) lockdown and (b) lift of the lockdown policy announcements upon extracted measures in different time windows before and after 23rd March and 10th May 2020, relative to that observed before and after 23rd March and 10th May 2019. This approach was strengthened by borrowing elements from a DiD approach and is similar to a difference in discontinuities design because it rests on the intuition of combining an RDiT with a DiD strategy (RDiT-DiD) (18). Further rationale and details of the analytic approach are provided in the Technical Appendix.

Differences in measures before versus after the cut-off dates were reported as incidence rate ratios (IRRs) and their corresponding 95% confidence intervals (CI). Negative binomial regression models were used to assess the effect of March 23rd national lockdown on mortality and mental health service activity adjusted for temporal trends and taking into

For peer review only - http://bmjopen.bmj.com/site/about/guidelines.xhtml

account overdispersion. Results from each site extracting daily data were pooled across sites using random effects meta-analysis and heterogeneity was summarised using the I^2 statistic (19).

A series of sensitivity analyses were also performed.

1. We re-ran a subgroup analysis of our main RDiT-DiD analysis for four sites with daily data that could update their data extractions to 31 July 2020. We increased the time window after the lift-of the lockdown announcement in order to capture any potential effects of the lift-of the lockdown announcement on mortality and mental health service activity with greater statistical certainty due to larger numbers.
2. To account for anticipatory effects of the lockdown and lift-of-lockdown announcements, we omitted one week either side of the cut-off date of 23rd March 2020 (i.e. 16th March 2020 to 30th March 2020) and one week either side of 10th May 2020 (i.e. 3rd May 2020 to 17th May 2020). Anticipatory effects, such as the announcement of social distancing on 16th March 2020, could have resulted in altered MH service activity in the study population just before the implementation of lockdown. Similarly, non-adherence to the lockdown rules was documented during the month of April 2020 and before lockdown was lifted (20).
3. To account for changes in the infection rate, we further adjusted our models for national COVID-19 related deaths occurring one week prior to the date of our observed daily counts.
4. To check robustness of our findings, that these were not an artefact of the pre-lockdown time period from 1st January 2020, we reduced the time window before the lockdown announcement from 4th February 2020. Thus, we used the same number of daily observations before and after the lockdown announcement (23rd March to 10th May 2020 vs. 4th February to 23rd March 2020) and the lift-of-lockdown announcement (10th to 31st May 2020 vs. 19th February to 23rd March 2020).

Analyses were performed using STATA 15.1 (Stata Corporation, College Station, TX USA).

For peer review only - http://bmjopen.bmj.com/site/about/guidelines.xhtml

RESULTS

The ten participating sites are described and compared in Table 1 and pooled IRRs from the random-effect meta-analysis are presented in Table 2 for the three period comparisons of interest in the sites extracting daily data.

Transition to lockdown

For this transition, pooled estimates showed a significant and greater than two-fold increase in daily deaths (IRR 2.31 (95% CI: 1.86-2.87); Figure 1). New accepted referrals decreased (IRR 0.62 (0.55-0.70)). Pooled estimates from inpatient care showed a decrease in new admissions (IRR 0.75 (0.67-0.83)), total daily caseload (IRR 0.85 (0.79-0.91)) and numbers of patients detained under an MHA section (IRR 0.93 (0.87-0.99)). Of community services evaluated, all showed sizeable decreases in daily numbers of f2f contacts and increases in non-f2f contacts (both strongest for EIP). Total contacts reduced in Liaison and HTT services and increased (but not significantly so) in AMH, CAMHS, EIP, and OA services. Daily caseloads significantly reduced in CAMHS, HTT and Liaison, and significantly increased in AMH. No significant changes were observed in the numbers of cancelled or non-attended appointments, apart from a reduction within HTT.

Transition from lockdown

Numbers of deaths reduced considerably (IRR 0.42 (0.27-0.66)), and IRR estimates for pre- vs. post-lockdown differences were not significantly different (0.97 (0.68-1.38)). Numbers of new site referrals increased after the lifting of lockdown (IRR 1.36 (1.15-1.60)) compared to the lockdown period but remained lower than pre-lockdown (IRR 0.85 (0.76-0.95)). Inpatient services saw increased admissions (IRR 1.21 (1.04-1.42)) and reduced discharges (IRR 0.66 (0.53-0.84)) after the lifting of lockdown. Discharges remained lower than before lockdown, as did caseloads. F2f contacts increased after lockdown in all services apart from AMH and CAMHS, increases in non-f2f contacts were seen in AMH, EIP and OA services, and total contacts increased in EIP, HTT, Liaison and OA services. Compared to pre-lockdown, pooled daily f2f contacts reduced and non-f2f contacts increased in all services after lifting of lockdown apart from HTT in which f2f contacts increased and non-f2f contacts were unchanged; total contacts increased in AMH, CAMHS, and EIP services and decreased in Liaison. Daily caseloads decreased in HTT and Liaison services, and cancelled or non-attended appointments reduced in Liaison services.

For peer review only - http://bmjopen.bmj.com/site/about/guidelines.xhtml

There was moderate to substantial heterogeneity in the majority of our IRRs estimates across the three periods (i.e., before, during, and after lockdown) in number of deaths, inpatient caseloads, f2f and non-f2f contacts and daily caseloads for all community services. For example, IRRs for number of deaths on transition into lockdown ranged from 1.55 (1.23-1.94) to 5.30 (2.87-9.79) (Figure 1). All IRRs are provided individually by site in Supplementary Tables 1-3. Inpatient caseload reductions on the transition into lockdown varied in IRRs from 0.67 (0.65-0.69) to 1.00 (0.99-1.01), reductions in HTT caseloads from 0.42 (0.39-0.45) to 0.98 (0.97-0.99), Liaison caseload reductions from 0.35 (0.31-0.39) to 1.03 (1.00-1.06). IRRs for the transition from before lockdown to lifting of lockdown (Supplementary Table 3) also varied, with daily deaths ranging from 0.31 (0.22-0.46) to 1.64 (1.29-2.09), those for inpatient caseloads from 0.87 (0.85-0.89) to 1.08 (1.07-1.09), HTT caseloads from 0.17 (0.16-0.18) to 0.91 (0.89-0.92), and Liaison caseloads from 0.41 (0.36-0.47) to 1.06 (1.03-1.10).

For Site I, where extractions of numbers of deaths and service activity data were weekly rather than daily and could not therefore be combined with other sites, there was a significant increase in weekly deaths (IRR 1.47 (1.35-1.60)) at lockdown, and a decrease (IRR 0.72 (0.66-0.80)) after the lifting of lockdown. Slightly, but non-significantly, increased numbers of deaths were observed after the lifting of lockdown compared to before lockdown. There was a reduction in admissions (IRR 0.64 (0.58-0.71)) and discharges (IRR 0.83 (0.73-0.94)) at lockdown, but no differences after the lifting of lockdown compared to before lockdown.

Sensitivity analyses

1. Further data for the post- vs. pre-lockdown comparisons are presented in Table 3 for the four sites able to extract daily data to 31st July. Considering the findings that were most consistently significant, all showed reduced total inpatient caseload, reduced OA f2f contacts, increased non-f2f AMH and EIP contacts, and reduced CAMHS, Liaison and OA caseloads.

2. When excluding data for one week around the lockdown and lift-of-lockdown announcements, minimal changes were observed, and IRRs remained largely similar (Supplementary Table 4).

For peer review only - http://bmjopen.bmj.com/site/about/guidelines.xhtml

3. Further adjustment for levels of national COVID-19 mortality levels (Supplementary Table 5) resulted in some dilution in the strength of IRRs for deaths, new admissions and daily caseloads at lockdown initiation, but minimal changes to those IRRs that were statistically significant. A few IRRs for the lift-of-lockdown transition fell below statistical significance (increases in inpatient admissions, in OA f2f contacts), as did some 'after vs. before' lockdown differences (reductions in Trust new referrals and inpatient discharges; increases in CAMHS total contacts).

4. Additionally, no substantial changes were observed in our IRRs when we employed different equally spaced exposure time window intervals around the lockdown and lift-of-lockdown announcements (Supplementary Table 6).

For peer review only

For peer review only - http://bmjopen.bmj.com/site/about/guidelines.xhtml

DISCUSSION

Summary of findings

This is the first multi-site study to present data and evaluate the impact of the COVID-19 pandemic and lockdown policy in the UK on mortality and MH service activity provision. In summary, the initiation of lockdown was associated with increases in the death rate, decreases in new accepted referrals, and decreases in inpatient admissions and daily caseload. In community services, sizeable increases were observed in non-f2f contacts, with decreases in f2f contacts. Caseloads decreased in CAMHS, HTT, Liaison and OA services, but increased slightly in AMH and EIP services. The lift-of-lockdown transition was associated with decreased deaths, increased referrals and inpatient admissions and decreased inpatient discharges. In community settings, most services saw increases in non-f2f contacts and total contacts. There was moderate to high heterogeneity of these estimates across sites; only site-wide new referrals/discharges, and inpatient admissions/discharges were consistent.

Impact of the COVID-19 first wave

The potential impact of the COVID-19 pandemic has been widely discussed in a general sense, focusing on the initial priorities of infection control, treatment options for severe complications, and the preparedness of critical care services (21). For mental healthcare, there is a need to understand the population-level impact of both the viral infection and the social distancing being imposed by many national governments (8, 22, 23). For people with pre-existing mental disorders, there is also a concern that vulnerability to COVID-19 infection may be higher than expected, because of infection susceptibility due to physical comorbidities and because of barriers to health service access (24, 25). A higher risk of relapse of mental disorders is a concern due to the stress of the pandemic itself, the stress of consequent quarantine (26), reduced access to routine outpatient visits for evaluations and prescriptions, and possibly also an avoidance of health services because of the perceived risk of infection or a wish to 'protect the NHS'. Finally, a higher risk of suicide might result from rapid social, economic, and health changes (27, 28). As far as we are aware, the scale of mental healthcare changes has not yet been fully quantified, although recommendations made in China for tighter admission criteria and reduced hospital outpatient visits, amongst others (29), may reflect similar changes in service provision to those we report.

For peer review only - http://bmjopen.bmj.com/site/about/guidelines.xhtml

While it was not our intention here to investigate factors underlying the observed service changes, many are likely to be unsurprising. Clearly, a reduction in f2f clinical contacts was a likely outcome of social distancing, plus rising concern about the infection risks to patients and staff of such contacts. These were balanced by an increase in non-f2f contacts, and the only reductions after 23rd March 2020 in total contacts were seen in Liaison and HTT services. These also saw strongest reductions in daily caseload, which for Liaison services may reflect high numbers of discharges from acute hospitals to prepare for a surge in pandemic-related demand, and possibly patient reluctance to attend emergency care. While HTT caseload reductions might reflect reduced demand, the levels and consequences of unmet need during the lockdown require further evaluation.

Between-site variation

An important feature of the findings was their heterogeneity between sites. Mainly this reflected magnitude rather than direction of effect: for example, all sites saw increases in deaths around lockdown initiation (Figure 1, Supplementary Table 1), although IRRs varied in size. This heterogeneity may reflect site differences in patient demographics and pandemic timing across the country. IRRs for inpatient caseload changes at the same transition point may reflect differences in facilities – for example, strongest caseload reductions were seen at Site A which had highest pre-lockdown levels (Table 1). On the other hand, reductions in HTT caseloads were weakest at Site H where pre-lockdown caseloads were highest, and Liaison caseload reductions differed substantially between sites (B and H) with similar pre-lockdown caseloads; this might reflect local differences in acute hospital practice/demand and indicates scope for further investigation.

Causality considerations

It is challenging to disentangle the effect of the ongoing COVID-19 pandemic itself from those of social distancing/lockdown policies (or other psychosocial consequences) on MH service activity and mortality. Our estimates retrieved from RDiT are of a compound effect: the causal effect of the 23rd March lockdown announcement on mental health service activity and any unobserved sorting/anticipation/adaptation/avoidance effects that may exist but cannot be tested for. The extent to which the results should be interpreted solely as the causal treatment effect of interest depend on the likelihood of other influences. For example, observed increases in mortality are likely to represent effects of the ongoing pandemic rather than lockdown policy; whereas for the service activity measures, adjustment for national

For peer review only

mortality (sensitivity analysis 3) is less of an issue and we consider those results to be robust. However, we did not attempt to capture the variation in the timing or magnitude of infection and mortality rates across the UK.

Strengths and limitations

Study strengths include the use of relatively 'real-time' mental healthcare data from ten large providers or routinely collected datasets, allowing investigation of changes in service activity following dramatic and rapid transitions at a national scale. The size of the data permitted precise estimates and the relative consistency in the direction of findings supports generalisability. Limitations include that data were drawn from specific services of interest and do not reflect the full activity of the ten sites; they were also combined by service type and we did not seek to investigate within-service (team-level) variation. Daily contact numbers were quantified from structured fields in electronic health records and might reflect recording behaviour rather than activity levels (e.g. if multiple contacts were subsumed within one entry); also, the dichotomy between f2f and non-f2f contact is a relatively crude one and does not reflect the quality or depth of assessments being recorded, nor the platform that was used (e.g. telephone vs. video). While we sought to harmonise data extractions as much as was feasible, there were inevitable differences in data availability between sites and likely variation in some measurements (e.g. in the completeness of mortality data in clinical systems), although we believe there should be robust within-site consistency across the time periods examined. Some IRR heterogeneity may reflect differences in service provision (e.g. acute hospital beds covered by a given Liaison service). Finally, here we report on what might be termed a 'natural experiment'; while individuals were not randomly assigned to experience lockdown or not, assignment is assumed to be quasi-random for observations close to the cut-off, allowing valid causal effects to be identified (30). Clearly the observation period was limited in duration and there will be a continuing need to collect data to evaluate potential longer-term consequences of both the firs and subsequent pandemic waves.

Implications

The alterations in MH service activity observed here have been profound and reflect the very limited time period over which to implement responses to the COVID-19 'first wave'. The sharp downturn in inpatient admissions and f2f community contacts are particularly concerning, as is the apparently much longer-term shift in balance from f2f to virtual contacts which remains a profound but unevaluated change in care. Predicting the continuing impact

For peer review only - http://bmjopen.bmj.com/site/about/guidelines.xhtml

of COVID-19 and related infection-control policies on MH service provision and demand remains challenging. For example, while national 'shared' emergencies may be experienced as less isolating for people with mental disorders, and some consequences may be less problematic than feared (27), treatment pathways are likely to have been disrupted significantly, particularly since social distancing/lockdown policies were imposed (continuously or intermittently) over many months. It is therefore imperative that national and international collaborations are established to monitor MH service needs and experiences of care as closely as possible, to identify adverse outcomes early enough for rapid intervention – in particular, to understand how people with new and existing severe mental health conditions can best be supported in a pandemic, given the need for socially distanced care input.

For peer review only

REFERENCES

1. Kontis V, Bennett JE, Rashid T, Parks RM, Pearson-Stuttard J, Guillot M, et al. Magnitude, demographics and dynamics of the effect of the first wave of the COVID-19 pandemic on all-cause mortality in 21 industrialized countries. Nature Medicine. 2020; https://doi.org/10.1038/s41591-020-1112-0.

2. Chang CK, Hayes RD, Perera G, Broadbent MTM, Fernandes AC, Lee WE, et al. Life expectancy at birth for people with serious mental illness, substance use disorders, and depressive disorders from a secondary mental health care case register in London, UK. PLoS One. 2011; 6: e19590.

3. Woodhead C, Ashworth M, Broadbent M, Callard F, Hotopf M, Schofield P, et al. Cardiovascular disease treatment among severe mental illness patients: a data linkage study between primary and secondary care. British Journal of General Practice. 2016; 66: e374-e81.

4. Woodhead C, Cunningham R, Ashworth M, Barley E, Stewart R, Henderson M. Cervical and breast cancer screening uptake among women with serious mental illness: a data linkage study. BMC Cancer. 2016; 16: 819.

5. WHO. The impact of COVID-19 on mental, neurological and substance use services: results of a rapid assessment. World Health Organization. 2020; https://www.who.int/publications/i/item/978924012455.

6. Leigh-Hunt N, Bagguley D, Bash K, Turner V, Turnbull S, Valtorta N, et al. An overview of systematic reviews on the public health consequences of social isolation and loneliness. Public Health. 2017; 152: 157-71.

7. Gayer-Anderson C, Latham R, El Zerbi C, Strang L, Moxham Hall V, Knowles G, et al. Impacts of social isolation among disadvantaged and vulnerable groups during public health crises. ESRC. 2020; https://esrc.ukri.org/files/news-events-and-publications/evidence-briefings/impacts-of-social-isolation-among-disadvantaged-and-vulnerable-groups-during-public-health-crises/.

8. Holmes EA, O'Connor RC, Perry VH, Tracey I, Wessely S, Arseneault L, et al. Multidisciplinary research priorities for the COVID-19 pandemic: a call for action for mental

For peer review only - http://bmjopen.bmj.com/site/about/guidelines.xhtml

health science. Lancet Psychiatry. 2020; Apr 15: https://doi.org/10.1016/S2215-0366(20)30168-1.

9. Torales J, O'Higgins M, Castaldelli-Maia JM, Ventriglio A. The outbreak of COVID-19 coronavirus and its impact on global mental health. International Journal of Social Psychiatry. 2020; 66(4): 317-20.

10. Lai J, Ma S, Wang Y, Cai Z, Hu J, Wei N, et al. Factors Associated With Mental Health Outcomes Among Health Care Workers Exposed to Coronavirus Disease 2019. JAMA Network Open. 2020; 3(3): e203976.

11. Livingston G, Rostamipour H, Gallagher P, Kalafatis C, Shastri A, Huzzey L, et al. Prevalence, management, and outcomes of SARS-CoV-2 infections in older people and those with dementia in mental health wards in London, UK: a retrospective observational study. Lancet Psychiatry. 2020; doi: https://doi.org/10.1016/S2215-0366(20)30434-X.

12. Johnson S, Dalton-Locke C, Vera San Juan N, Foye U, Oram S, Papamichail A, et al. Impact on mental health care and on mental health service users of the COVID-19 pandemic: a mixed methods survey of UK mental health care staff. Social Psychiatry and Psychiatric Epidemiology. 2020.

13. Chen S, Jones PB, Underwood BR, Moore A, Bullmore ET, Banerjee S, et al. The early impact of COVID-19 on mental health and community physical health services and their patients' mortality in Cambridgeshire and Peterborough, UK. Journal of Psychiatric Research. 2020; 22 Sep 2020: https://doi.org/10.1016/j.jpsychires.2020.09.020.

14. Stewart R, Martin E, Bakolis I, Broadbent M, Byrne N, Landau S. Comparison of mental health service activity before and shortly after UK social distancing responses to the COVID-19 pandemic: February-March 2020. medRxiv. 2020; 28 Sep 2020: https://www.medrxiv.org/content/10.1101/2020.09.26.20202150v1.

15. Stewart R, Broadbent M, Das-Munshi J. Excess mortality in mental health service users during the COVID-19 pandemic described by ethnic group: South London and Maudsley data. medRxiv. 2020; 14 Jul 2020: https://medrxiv.org/cgi/content/short/2020.07.13.20152710v1.

For peer review only

16. Stewart R, Martin E, Broadbent M. Mental health service activity during COVID-19 lockdown: South London and Maudsley data on working age community and home treatment team services and mortality from February to mid-May 2020. medRxiv. 2020; 16 Jun 2020: https://www.medrxiv.org/content/10.1101/2020.06.13.20130419v1

17. Hausman C, Rapson DS. Regression discontinuity in time: considerations for empirical applications. Davis Energy Economics Program. 2018; April: http://deep.ucdavis.edu/uploads/5/6/8/7/56877229/deep_wp019.pdf.

18. Grembi V, Nannicini T, Troiano U. Policy Responses to Fiscal Restraints: A Difference-inDiscontinuities Design. 2012: https://www.princeton.edu/rppe/speaker-series/speakerseries-2012-13/Troiano.pdf.

19. DerSimonian R, Laird N. Meta-analysis in clinical trials. Controlled Clinical Trials. 1986; 7: 177-88.

20. Jeffrey B, Walters CE, Ainslie KEC, Eales O, Ciavarella C, Bhatia S, et al. Anonymised and aggregated crowd level mobility data from mobile phones suggests that initial compliance with COVID-19 social distancing interventions was high and geographically consistent across the UK. Imperial College London. 2020; 29 May 2020: https://doi.org/10.25561/79387.

21. Adalja AA, Toner E, Inglesby TV. Priorities for the US health community responding to COVID-19. Journal of the American Medical Association. 2020; 323: 1343-4.

22. WHO. Mental health and psychosocial considerations during the COVID-19 outbreak. World Health Organization. 2020; https://www.who.int/docs/default-source/coronaviruse/mental-health-considerations.pdf?sfvrsn=6d3578af_2.

23. Auerbach J, Miller BF. COVID-19 exposes the cracks in our already fragile mental health system. American Journal of Public Health. 2020; Apr 9: doi:10.2105/AJPH.020.305699.

24. Yao H, Chen JH, Xu YF. Patients with mental health disorders in the COVID-19 epidemic. Lancet Psychiatry. 2020; 7: e21.

For peer review only

For peer review only - http://bmjopen.bmj.com/site/about/guidelines.xhtml

25. Wang Q, Xu R, Volkow ND. Increased risk of COVID-19 infection and mortality in people with mental disorders: analysis from electronic health records in the United States. . World Psychiatry. 2020; 7 October: DOI: 10.1002/wps.20806.

26. Brooks SK, Webster RK, Smith LE, Woodland L, Wessely S, Greenberg N, et al. The psychological impact of quarantine and how to reduce it: rapid review of the evidence. Lancet. 2020; 395: 912-20.

27. Reger MA, Stanley IH, Joiner TE. Suicide mortality and coronavirus disease 2019 - a perfect storm? JAMA Psychiatry. 2020; April 10: doi:10.1001/jamapsychiatry.2020.1060.

28. Gunnell D, Appleby L, Arensman E, Hawton K, John A, Kapur N, et al. Suicide risk and prevention during the COVID-19 pandemic. Lancet Psychiatry. 2020; 7: 468-71.

29. Xiang YT, Zhao YJ, Liu ZH, Li XH, Zhao N, Cheung T, et al. The COVID-19 outbreak and psychiatric hospitals in China: managing challenges through mental health service reform. International Journal of Biological Sciences. 2020; 16: 1741-4.

30. Bor J, Moscoe E, Mutevedzi P, Newell ML, Barnighausen T. Regression discontinuity designs in epidemiology: causal inference without randomized trials. Epidemiology. 2014; 25(5): 729-37.

For peer review only

Table 1. Descriptive characteristics of daily mortality and mental health service caseloads by site

Measures	Site	Before lockdown* Median (IQR)	During lockdown* Median (IQR)	After lockdown* Median (IQR)
Number of deaths	A	13 (11-17)	30 (22-38)	13 (11-16)
	B	44 (39-51)	67 (61-78)	40 (29-47)
	C	0 (0-0)	0 (0-1)	0 (0-0)
	D	25 (22-28)	42 (28-50)	7 (4-18)
	E	2 (0-3)	3 (0-6)	0 (0-1)
	F	12 (9-14)	18 (12-21)	13 (10-16)
	G	7 (6-9)	10 (7-12)	5 (4-7)
	H	3 (2-4)	4 (3-6)	3 (2-3)
	I	95 (93-100) ^	130 (112-146) ^	85 (80-88) ^
	J	10 (7-13)	18 (14-26)	11 (8-12)
Inpatient caseload	A	768 (762-774)	556 (544-567)	581 (568-588)
	B	454.5 (445-461)	363 (357-368)	375 (374-379)
	C	495 (489-503)	427.5 (422-443.5)	451 (443-454)
	D	701 (696-707)	625 (617-657)	623 (613-640)
	E	481 (471-491)	412 (400-418)	453 (437-463)
	F	207 (205-211)	140 (135-146)	147 (146-148)
	H	408 (399-412)	351 (338-360)	367 (358-381)
	J	400 (394-407)	361 (354-371)	375 (365-385)
AMH (community) caseload	A	8691 (8666-8714)	8504 (8438-8576)	8398 (8389-8409)
	B	8700 (8652-8739)	8039 (7823-8297)	7555 (7498-7587)
	D	11039.5 (10956-11091)	10803 (10688-10923)	10526 (10520-10559)
	F	2228 (2221-2242)	2178 (2167-2195)	2158 (2156-2162)
	H	724.5 (719-726)	695 (686-700)	684 (683-686)
	J	10687 (10506-10981)	10160 (9866-10519)	9599 (9529-9612)
CAMHS caseload	A	6915.5 (6792-7021)	6962 (6875-6997)	6789 (6752-6801)
	D	10240.5 (10187-10292)	10060 (9935-10189)	9807 (9795-9813)
	E	38 (37-38)	35 (35-35)	35 (35-35)
	F	2232 (2176-2247)	2106 (2061-2144)	1992 (1988-1999)
	H	4375.5 (4347-4445)	4383 (4234-4414)	4106 (4078-4137)
	J	15946.5 (15610-16046)	14821 (14342-15394)	13835 (13802-13942)
EIP caseload	A	1193 (1176-1208)	1190 (1185-1198)	1206 (1199-1208)
	B	354.5 (347-358)	333 (329-343)	336 (335-341)
	D	809 (805-812)	794 (790-806)	806 (804-810)
	E	862 (857-867)	852 (849-853)	860 (859-862)
	F	93 (92-96)	97 (96-98)	95 (93-95)
	H	768 (765-771)	767 (762-771)	765 (763-768)
	J	421 (415-424)	421 (419-423)	421 (415-424)
HTT caseload	A	214.5 (207-222)	153 (148-158)	191 (187-197)
	B	258.5 (241-276)	151 (139-161)	198 (188-202)
	D	263 (247-289)	162 (151-177)	206 (197-214)
	E	219 (202-234)	95 (87-103)	127 (121-133)
	F	73 (64-77)	54 (50-62)	56 (50-59)
	H	977.5 (968-996)	780 (715-850)	732 (725-740)
	J	354.5 (293-418)	100 (60-165)	50 (48-51)
Liaison caseload	A	1060 (1044-1074)	971 (956-980)	1005 (999-1015)
	B	80.5 (69-91)	27 (21-29)	47 (39-51)
	D	1851.5 (1786-1879)	1523 (1494-1573)	1505 (1493-1522)
	E	103 (90-110)	48 (42-65)	81 (75-86)
	F	499 (468-516)	407 (403-411)	448 (436-455)
	H	59.5 (57-62)	64 (63-68)	71 (68-74)
	J	221 (216-227)	203 (193-211)	200 (197-202)
OA caseload	A	1297 (1264-1308)	1065 (1018-1126)	1001 (991-1007)
	B	6243 (6193-6257)	5518 (5336-5746)	5149 (5132-5191)
	D	5731.5 (5677-5791)	5497 (5398-5596)	5346 (5338-5352)
	E	567 (561-582)	503 (497-508)	517 (511-518)
	F	502 (487-507)	350 (340-386)	344 (342-345)
	H	1638 (1621-1654)	1328 (1288-1383)	1261 (1259-1263)
	J	3844 (3821-3857)	3426 (3337-3557)	3284 (3280-3286)

*Before lockdown: 01.01.2020-22.03.2020; During lockdown: 23.03.2020-09.05.2020; After lockdown: 10.05.2020-31.05.2020
AMH: adult mental health service; CAMHS: child and adolescent mental health service; EIP: early intervention for psychosis service; HTT: home treatment team; OA: older adult service. ^For Site I we ascertained median and IQR for weekly mortality and have divided each estimate here by 7 for comparability.

For peer review only

Table 2. Estimated effects of lockdown and lift-of-lockdown announcements on service measures meta-analysed across sites A-H and J from 1st Jan to 31st May 2020, estimating transitions related to the lockdown announcement (23rd March) and lift-of-lockdown announcement (10th May). Incidence Rate Ratios (IRRs) and 95% confidence intervals and percentage heterogeneity across sites (I^2) were estimated.

	Lockdown announcement		Lift of Lockdown announcement		Lift of Lockdown vs. Before Lockdown	
**Measures**	**IRR (95%CI)**	**I^2**	**IRR (95%CI)**	**I^2**	**IRR (95%CI)**	**I^2**
Number of deaths	2.31*(1.86-2.87)	85.3	0.42*(0.27-0.66)	91.8	0.97 (0.68-1.38)	88.5
Trust-wide activity						
Number of new referrals accepted	0.62*(0.55-0.70)	0.0	1.36*(1.15-1.60)	0	0.85*(0.76-0.95)	0.0
Number of discharges	0.95 (0.85-1.06)	0.0	0.95 (0.81-1.11)	0	0.89 (0.80-1.00)	0.0
Inpatient care						
New admissions	0.75*(0.67-0.83)	0.0	1.21*(1.04-1.42)	0	0.90 (0.79-1.03)	0.0
Discharges	1.13 (0.97-1.31)	0.0	0.66*(0.53-0.84)	5.2	0.75*(0.62-0.90)	0.0
Inpatient caseload	0.85*(0.79-0.91)	99.6	1.06 (1.00-1.12)	99.0	0.90*(0.83-0.97)	99.7
Inpatient caseload on a MHA section	0.93^(0.87-0.99)	99.4	1.01 (0.93-1.10)	99.6	0.94 (0.86-1.02)	99.8
AMH (community)						
F2f contacts	0.55*(0.44-0.68)	49.4	0.99 (0.80-1.23)	0	0.57*(0.44-0.75)	40.2
Non-f2f contacts	3.74*(2.58-5.43)	83.2	1.27*(1.06-1.52)	0	3.95*(2.73-5.71)	70.3
F2f and Non-f2f contacts	1.07 (0.94-1.22)	0.0	1.12 (0.94-1.34)	0	1.19*(1.04-1.36)	0.0
Cancelled appointments or DNAs	0.85 (0.61-1.18)	73.1	1.02 (0.80-1.30)	10.6	0.79(0.56-1.11)	61.7
Caseload	1.03^(1.01-1.05)	98.2	1.01 (1.00-1.02)	94.6	1.04*(1.02-1.04)	0.0
CAMHS						
F2f contacts	0.35*(0.17-0.74)	93.2	1.12 (0.79-1.61)	14.5	0.36*(0.15-0.88)	88.8
Non-f2f contacts	3.58*(2.54-5.04)	70.2	1.23 (1.00-1.50)	0	3.76*(2.69-5.25)	50.1
F2f and Non-f2f contacts	1.08 (0.93-1.26)	0.0	1.20 (0.96-1.49)	0	1.23*(1.04-1.47)	0.0
Cancelled appointments or DNAs	0.87(0.67-1.14)	48.8	0.94 (0.65-1.36)	41.2	0.89 (0.76-1.04)	0.0
Caseload	0.93^(0.89-0.97)	99.2	1.03 (0.98-1.08)	99.3	0.94 (0.88-1.01)	99.5
EIP						
F2f contacts	0.27*(0.18-0.42)	86.0	1.64*(1.19-2.27)	48.4	0.45*(0.29-0.70)	82.5
Non-f2f contacts	4.04*(2.63-6.20)	83.3	1.48*(1.03-2.12)	52.9	5.51*(3.08-9.84)	87.5
F2f and Non-f2f contacts	1.09 (0.88-1.34)	51.6	1.45*(1.22-1.72)	0	1.51*(1.22-1.88)	49.7
Cancelled appointments or DNAs	0.76 (0.54-1.08)	74.4	0.99 (0.72-1.36)	44.3	0.70 (0.44-1.12)	85.2
Caseload	1.04^(1.02-1.06)	97.9	1.04 (1.00-1.08)	99.5	1.08*(1.03-1.13)	99.8
HTT						
F2f contacts	0.43*(0.32-0.57)	97.0	1.49*(1.36-1.62)	37.5	1.91*(1.49-2.43)	84.3
Non-f2f contacts	1.78*(1.56-2.03)	60.3	1.07 (0.87-1.32)	69.6	0.99 (0.87-1.14)	85.9
F2f and Non-f2f contacts	0.78*(0.68-0.89)	90.3	1.27*(1.16-1.39)	52.6	1.08 (0.87-1.34)	58.3
Cancelled appointments or DNAs	0.68*(0.51-0.89)	85.6	1.47*(1.11-1.96)	67.9	0.62*(0.43-0.88)	99.8
Caseload	0.62*(0.49-0.78)	99.7	1.01 (0.78-1.29)	99.4	0.64*(0.51-0.82)	94.4
Liaison						
F2f contacts	0.43*(0.36-0.50)	78.2	1.67*(1.39-2.00)	59.0	0.72*(0.61-0.86)	66.3
Non-f2f contacts	1.50*(1.15-1.96)	68.8	1.11 (0.81-1.53)	52.5	1.69*(1.16-2.47)	74.2
F2f and Non-f2f contacts	0.60*(0.54-0.66)	44.5	1.46*(1.29-1.65)	28.3	0.85^(0.74-0.98)	56.7
Cancelled appointments or DNAs	0.62(0.35-1.10)	84.5	0.75 (0.46-1.24)	45.9	0.65*(0.43-0.97)	41.6
Caseload	0.75*(0.69-0.81)	99.1	1.14*(1.07-1.21)	97.7	0.88*(0.83-0.93)	97.9
OA						
F2f contacts	0.32*(0.20-0.49)	83.8	1.45*(1.13-1.85)	0.35	0.39*(0.23-0.67)	82.3
Non-f2f contacts	3.66*(2.54-5.27)	78.7	1.28^(1.02-1.59)	0	4.27*(2.78-6.56)	73.3
F2f and Non-f2f contacts	1.06 (0.81-1.37)	62.5	1.31*(1.06-1.63)	0	1.28 (0.94-1.73)	50.8
Cancelled appointments or DNAs	1.08 (0.73-1.58)	77.9	0.70 (0.38-1.31)	75.8	0.80 (0.51-1.26)	63.1
Caseload	0.95 (0.92-0.99)	99.3	0.99 (0.96-1.01)	98.7	0.93 (0.84-1.03)	99.8
**Site I data†**						
Number of deaths	1.47*(1.35-1.60)		0.72*(0.66-0.80)		1.07 (0.99-1.15)	
New inpatient admissions	0.64*(0.58-0.71)		1.67*(1.48-1.88)		1.07 (0.93-1.22)	
Inpatient discharges	0.83^(0.73-0.94)		1.19*(1.08-1.32)		0.99 (0.88-1.10)	

^p<0.05; *p<0.001

†Derived from weekly data from 1st Jan to 5th Jul 2020.

AMH: adult mental health service; CAMHS: child and adolescent mental health service; EIP: early intervention for psychosis service; HTT: home treatment team; OA: older adult service.

For peer review only

For peer review only - http://bmjopen.bmj.com/site/about/guidelines.xhtml

Table 3. Sensitivity analyses of lift-of-lockdown vs. pre-lockdown comparisons for the extended period of follow-up (to 31st July 2020) available for four sites. Incidence Rate Ratios (IRRs) and 95% confidence intervals were estimated.

	Site A	Site F	Site G	Site J
	Lift of the Lockdown vs. Pre-lockdown	Lift of the Lockdown vs. Pre-lockdown	Lift of the Lockdown vs. Pre-lockdown	Lift of the Lockdown vs. Pre-lockdown
**Measures**	**IRR (95%CI)**	**IRR (95%CI)**	**IRR (95%CI)**	**IRR (95%CI)**
Number of deaths	1.20 (0.95-1.51)	2.06*(1.09-3.90)	0.89 (0.65-1.20)	1.08 (0.84-1.40)
Trust-wide activity				
Number of new referrals accepted	0.79*(0.68-0.93)	0.55^(0.30-0.98)	N/A	0.73 (0.50-1.07)
Number of discharges	0.83*(0.72-0.96)	0.60 (0.31-1.16)	N/A	0.74 (0.51-1.06)
Inpatient care				
New admissions	0.94 (0.75-1.18)	0.34 (0.06-1.83)	N/A	0.94 (0.62-1.42)
Discharges	0.64*(0.48-0.86)	0.24 (0.03-2.22)	N/A	0.81 (0.51-1.28)
Inpatient caseload	0.76*(0.74-0.77)	0.62*(0.59-0.66)	N/A	0.96^(0.95-0.97)
Inpatient caseload on a MHA section	0.87*(0.85-0.88)	1.02 (0.96-1.09)	N/A	1.01 (0.99-1.02)
Adult mental health (community)				
F2f contacts	0.50*(0.38-0.67)	0.44 (0.05-4.32)	0.31*(0.18-0.54)	0.47*(0.32-0.69)
Non-f2f contacts	2.99*(2.45-3.64)	12.11*(1.64-89.29)	13.66*(7.77-24.03)	8.69*(6.22-12.15)
F2f and Non-f2f contacts	1.28*(1.08-1.53)	0.97 (0.11-8.55)	0.83 (0.47-1.46)	1.68*(1.16-2.42)
Cancelled appointments or DNAs	0.70*(0.58-0.85)	0.39 (0.04-3.53)	0.73 (0.43-1.23)	1.06 (0.67-1.68)
Caseload	0.94^(0.91-0.99)	1.04^(1.02-1.04)	N/A	0.92*(0.90-0.93)
CAMHS				
F2f contacts	0.14*(0.08-0.26)	1.70 (0.17-16.59)	0.65 (0.40-1.03)	0.08*(0.05-0.14)
Non-f2f contacts	3.60*(2.96-4.37)	4.78 (0.77-29.85)	16.12*(9.28-27.99)	3.32*(2.19-5.04)
F2f and Non-f2f contacts	1.27*(1.03-1.56)	1.98 (0.22-17.86)	1.15 (0.75-1.77)	1.35 (0.89-2.05)
Cancelled appointments or DNAs	0.90 (0.74-1.10)	0.59 (0.06-5.61)	0.46*(0.25-0.83)	0.59*(0.38-0.93)
Caseload	0.95^(0.91-0.97)	0.96*(0.95-0.97)	N/A	0.84*(0.82-0.85)
EIP				
F2f contacts	0.20*(0.16-0.26)	0.79 (0.10-6.43)	N/A	0.22*(0.16-0.31)
Non-f2f contacts	4.22*(3.39-5.25)	35.59*(3.38-74.33)	N/A	13.90*(9.05-21.37)
F2f and Non-f2f contacts	1.39*(1.16-1.67)	1.32 (0.18-9.73)	N/A	1.95*(1.59-2.39)
Cancelled appointments or DNAs	0.55*(0.43-0.71)	0.86 (0.09-7.78)	N/A	1.15 (0.77-1.73)
Caseload	0.95^(0.92-0.98)	1.07*(1.05-1.08)	N/A	0.98^(0.96-0.99)
HTT				
F2f contacts	0.68*(0.61-0.74)	0.99 (0.51-1.92)	N/A	0.09*(0.06-0.13)
Non-f2f contacts	2.47*(2.05-2.96)	2.72*(1.04-7.14)	N/A	2.32*(1.51-3.56)
F2f and Non-f2f contacts	0.99 (0.91-1.07)	1.37 (0.73-2.56)	N/A	0.38*(0.28-0.51)
Cancelled appointments or DNAs	1.23 (0.97-1.56)	1.33 (0.34-5.29)	N/A	0.48*(0.27-0.86)
Caseload	0.81*(0.78-0.84)	1.16 (0.91-1.49)	N/A	0.13*(0.12-0.14)
Liaison				
F2f contacts	0.95 (0.84-1.07)	0.72 (0.19-2.76)	0.46*(0.39-0.54)	0.60*(0.47-0.78)
Non-f2f contacts	1.94*(1.48-2.54)	17.87*(10.08-78.09)	5.26*(3.42-8.07)	1.34 (0.91-1.98)
F2f and Non-f2f contacts	1.03 (0.92-1.16)	2.31 (0.55-9.71)	0.88 (0.74-1.04)	0.77*(0.62-0.95)
Cancelled appointments or DNAs	0.69 (0.43-1.10)	0.11 (0.02-0.70)	1.48 (0.91-2.41)	1.12 (0.61-2.05)
Caseload	0.95*(0.94-0.96)	0.82*(0.76-0.89)	N/A	0.75*(0.73-0.76)
Older adults				
F2f contacts	0.51*(0.41-0.64)	0.04*(0.00-0.32)	0.27*(0.16-0.44)	0.21*(0.13-0.33)
Non-f2f contacts	2.04*(1.62-2.56)	6.60 (0.69-63.35)	5.37*(2.80-10.29)	5.70*(3.91-8.29)
F2f and Non-f2f contacts	0.95 (0.78-1.16)	0.42 (0.04-4.20)	0.95 (0.58-1.55)	1.78*(1.20-2.64)
Cancelled appointments or DNAs	1.05 (0.60-1.82)	0.23 (0.02-2.79)	0.37*(0.19-0.74)	0.97 (0.68-1.37)
Caseload	0.76*(0.73-0.79)	0.95*(0.93-0.97)	N/A	0.81*(0.79-0.81)

^p<0.05; *p<0.001
AMH: adult mental health service; CAMHS: child and adolescent mental health service; EIP: early intervention for psychosis service; HTT: home treatment team; OA: older adult service. N/A: not available data for these type of services

For peer review only

For peer review only

For peer review only - http://bmjopen.bmj.com/site/about/guidelines.xhtml

Figure 1. Forest plots of changes in daily deaths by site associated with lockdown and lift-of-lockdown announcements, with pooled meta-analysis estimates. Incidence Rate Ratios (IRRs) and 95% confidence intervals, percentage heterogeneity (I^2) and p-values from chi-square tests of heterogeneity are displayed.

(A) Initiation of lockdown announcement

(B) Lift of lockdown announcement

(C) Lift of lockdown vs. pre-lockdown

For peer review only

For peer review only - http://bmjopen.bmj.com/site/about/guidelines.xhtml

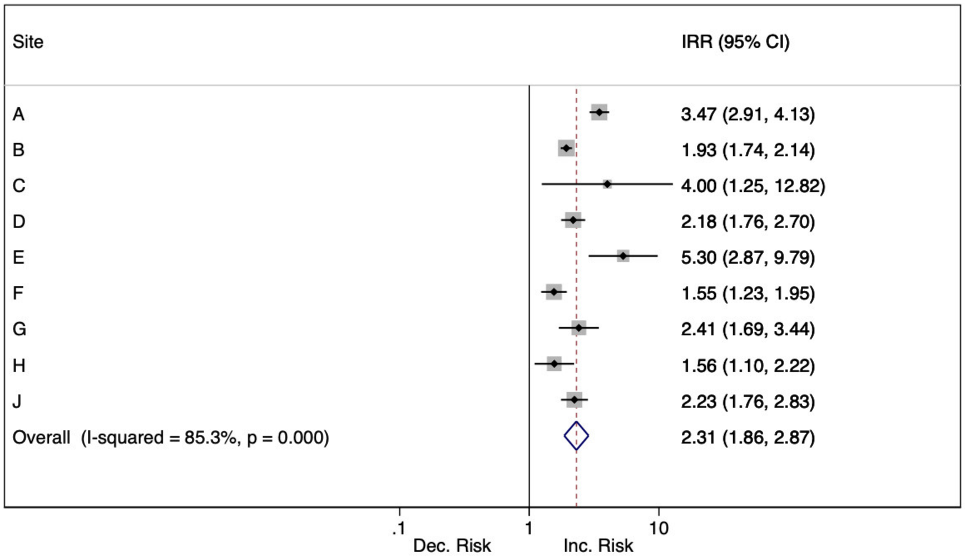

Figure 1A

101x61mm (300 x 300 DPI)

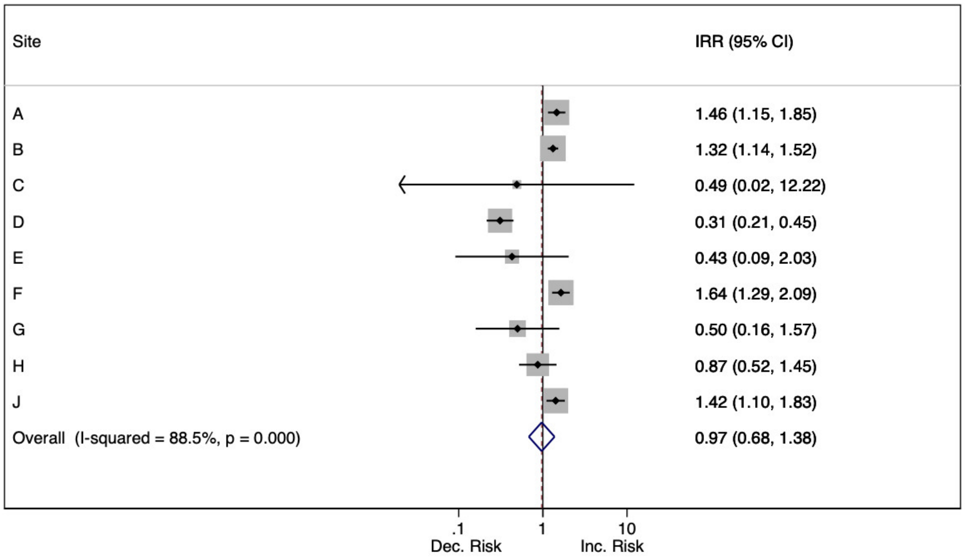

Figure 1B

101x61mm (300 x 300 DPI)

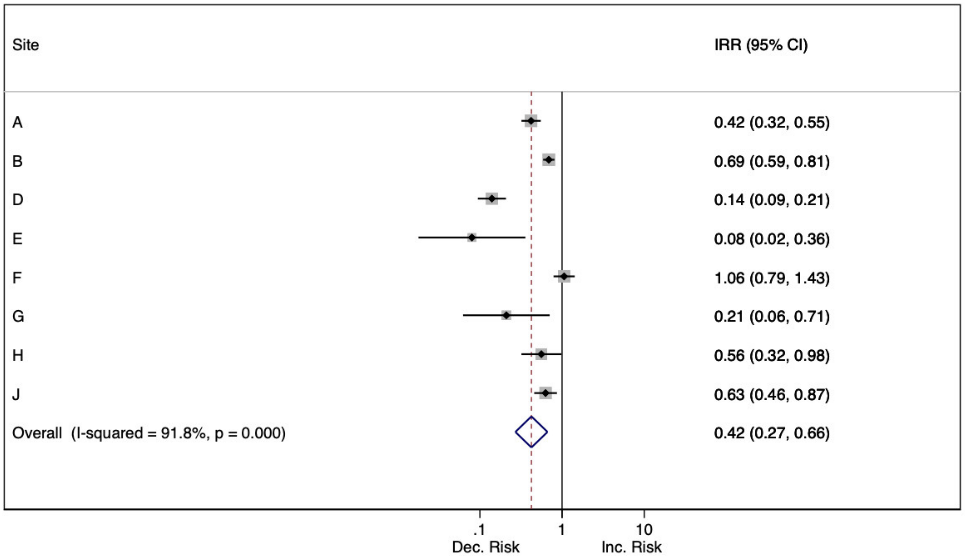

Figure 1C

101x61mm (300 x 300 DPI)

TECHNICAL APPENDIX

Rationale for statistical analysis

Regression discontinuity is a quasi-experimental design that has been applied previously for the evaluation of nationwide policies,[1] though this approach has found little application in epidemiology to date.[2,3] Regression discontinuity in time (RDiT),[4] applied here is being employed for the analysis of time series data. In our RDiT framework, we know the date c of a policy change (here 23rd March 2020 and 10th May 2020). Thus, we assume that for all dates $t > c$, the unit is 'treated', and for all dates $t < c$, the unit is not. This RDiT set-up uses time-series data: in our case, daily counts across 10 UK sites. Two comparable time intervals (cohorts) are chosen: in our case, a period in 2019 and one in 2020. In one interval, but not the other, an event (discontinuity) occurs: in this study, the start or end of 'lockdown'. Changes at the discontinuity are measured, with the other interval acting as the control. The key feature of RDiT is the focus on comparing outcomes in a 'short' time interval before the policy implementation with a 'short' time interval after the policy implementation relative to changes observed in the previous year. We therefore assume that no 2020-specific unobserved factors confound the relationship between the exposure and the outcome by using these short time windows (and in this analysis, considered changes in national infection rates as a particular confounder).

The conditions for a valid RDiT analysis[3] were met in our study: i) the decision rule (exposed or not exposed to the intervention) and cut-off value (23rd March 2020 and 10th May 2020) were known; ii) the assignment variable (distance from the lockdown announcement, measured in days) was continuous near the cut-off value and was not affected by the policy (see Supplementary Figures 1-4); iii) there was visual confirmation of an intervention effect, in that a graphical analysis (Supplementary Figures 1-4) confirmed the discontinuity, i.e. a visible jump at the cut-off value, indicating a policy effect. We also assumed parallel trends in the time window around the lockdown and lift of lockdown announcement (a form of common/parallel-trend assumption). We further fitted different models to our time series data, with our final model being visualised in Supplementary Figure 5.

For peer review only - http://bmjopen.bmj.com/site/about/guidelines.xhtml

Further details of statistical analyses

We adopted a parametric specification for the conditional expectation functions which account for heterogeneity of temporal effects (by weekday and month). We included functions to adjust for seasonal trends before and after the cut-off points

$$Y_{i,c} = \beta_0 + \beta_1 T_{i,c} + \beta_2 Year_{i,c} + \beta_3 T_{i,c} * Year_{i,c} + \beta_4 f(D_{i,c}) + \beta_5 f(D_{i,c}) * T_{i,c} + \beta_6 f(D_{i,c}) * Year_{i,c} + \beta_7 f(D_{i,c}) * T_{i,c} * Year_{i,c} + \beta_8 W_{i,c} + \beta_9 M_{i,c} + logE_c + \varepsilon_{i,c}$$

Within each day i and site c we employed negative binomial regression to model daily counts of mortality and mental health service use activity levels defined as $Y_{i,c}$. We allowed for overdispersion and accounted for a proportional relationship between activity counts and the total daily caseload of the site by mean of an offset $\log E_c$. $T_{i,c}$ is a categorical variable whose levels were: before lockdown, during lockdown, or after lockdown (or equivalent periods in 2019). The year of the lockdown is binary variable $Year_{i,c}$ and corresponds to 2020. $D_{i,c}$ is defined as the absolute distance in days from the lockdown order announcement -$f(D_{i,c})$ is a linear function of the distance in days from the lockdown announcement interacted with the lockdown variable $T_{i,c}$ and the $Year_{i,c}$ to allow for different effects on either side of the cut-off. The model also includes $W_{i,c}$ and $M_{i,c}$ defined as set of dummy variables of weekdays and months respectively. The parameter of interest, which identifies the effect of the lockdown, is the vector β_3 which is the coefficients associated with the interaction term between our pre-post policy dummy variable $T_{i,c}$and cohort variable $Year_{i,c}$. β_1,β_2,β_3, ...,β_9 are fixed effects coefficients. Differences in daily measures before versus after the cut-off dates are reported as relative risks (RR) with their corresponding 95% confidence intervals (CI). Negative binomial regression results were pooled across sites using random effects meta-analysis and heterogeneity was summarised using the I^2 statistic.

We performed the same negative binomial modelling with minor modifications for Site I weekly data. The time resolution was changed to week and only $M_{i,c}$ was included as an additional categorical covariate. Variables T_i and D_i were defined with respect to the week numbers of the announcement (week 13) and lift (week 20) of lockdown.

For peer review only

For peer review only - http://bmjopen.bmj.com/site/about/guidelines.xhtml

References

1. Bakolis I, Kelly R, Fecht D, et al. Protective effects of smoke-free legislation on birth outcomes in England: a regression discontinuity design. *Epidemiology* 2016; **27**: 810-8.

2. O'Keeffe AG, Geneletti S, Baio G, Sharples LD, Nazareth I, Petersen I. Regression discontinuity designs: an approach to the evaluation of treatment efficacy in primary care using observational data. *BMJ* 2014; **349**: g5293.

3. Moscoe E, Bor J, Bärnighausen T. Regression discontinuity designs are underutilized in medicine, epidemiology, and public health: a review of current and best practice. *Journal of Clinical Epidemiology* 2015; **68**: 132-43.

4. Imbens G, Lemieux T. Regression Discontinuity Designs: A Guide to Practice. *Journal of Econometrics* 2008; **142**: 615-35.

5. DerSimonian R, Laird N. Meta-analysis in clinical trials. *Controlled Clinical Trials* 1986; **7**: 177-88.

For peer review only

Supplementary Table 1: Site-specific data for lockdown initiation changes in daily mortality and service activity

	IRR (95%CI) for lockdown vs. pre-lockdown comparisons								
**Measures**	**Site A**	**Site B**	**Site C**	**Site D**	**Site E**	**Site F**	**Site G**	**Site H**	**Site J**
Number of deaths	3.47(2.92-4.14)	1.93(1.74-2.13)	4.00(1.25-12.85)	2.18(1.76-2.70)	5.30(2.87-9.79)	1.55(1.23-1.94)	2.41(1.69-3.44)	1.56(1.10-2.22)	2.23(1.76-2.84)
Trust-wide activity									
Number of new referrals accepted	0.64(0.46-0.90)	0.65(0.45-0.93)	N/A	0.62(0.46-0.83)	N/A	0.62(0.52-0.75)	N/A	0.62(0.43-0.89)	0.60(0.42-0.87)
Number of discharges	0.96(0.78-1.19)	1.08(0.75-1.56)	N/A	1.00(0.71-1.41)	N/A	0.89(0.73-1.09)	N/A	0.74(0.46-1.18)	1.05(0.78-1.41)
Inpatient care									
New admissions	0.81(0.67-0.99)	0.77(0.57-1.03)	0.59(0.45-0.77)	0.79(0.60-1.04)	0.72(0.47-1.11)	0.76(0.53-1.09)	N/A	0.73(0.53-0.99)	0.77(0.55-1.06)
Discharges	1.25(0.92-1.69)	1.06(0.68-1.63)	1.02(0.66-1.59)	1.24(0.75-2.06)	0.99(0.57-1.72)	1.10(0.72-1.69)	N/A	1.48(0.91-2.40)	0.92(0.62-1.39)
Inpatient caseload	0.77(0.75-0.79)	0.82(0.81-0.84)	0.91(0.90-0.92)	0.97(0.96-0.98)	0.79(0.77-0.80)	0.67(0.65-0.69)	N/A	0.89(0.87-0.90)	1.00(0.99-1.01)
Inpatient caseload (MHA)	0.84(0.82-0.86)	0.93(0.92-0.94)	N/A	1.00(0.99-1.02)	N/A	0.78(0.76-0.80)	N/A	0.99(0.97-1.00)	1.05(1.04-1.06)
AMH (community)									
F2f contacts	0.54(0.41-0.70)	0.44(0.27-0.71)	N/A	1.20(0.70-2.06)	0.72(0.42-1.23)	0.44(0.27-0.73)	0.37(0.14-0.97)	0.57(0.40-0.82)	0.41(0.29-0.59)
Non-f2f contacts	2.13(1.71-2.66)	3.16(2.11-4.74)	N/A	3.14(1.80-5.47)	3.04(1.75-5.31)	8.38(4.97-14.13)	8.22(3.65-18.56)	2.24(1.41-3.54)	5.32(3.74-7.55)
F2f and Non-f2f contacts	1.03(0.85-1.24)	1.21(0.77-1.89)	N/A	1.38(0.81-2.36)	1.11(0.65-1.89)	0.86(0.52-1.43)	0.51(0.20-1.30)	1.19(0.80-1.77)	1.15(0.83-1.61)
Cancelled/DNA	0.52(0.41-0.66)	1.24(0.76-2.02)	N/A	1.28(0.75-2.19)	0.45(0.25-0.81)	1.17(0.63-2.18)	0.54(0.22-1.38)	1.13(0.69-1.85)	0.93(0.62-1.41)
Caseload	1.02(1.00-1.04)	1.03(1.03-1.04)	N/A	1.02(1.00-1.04)	1.01(1.00-1.02)	1.02(1.01-1.03)	N/A	1.02(1.02-1.03)	1.06(1.05-1.07)
CAMHS									
F2f contacts	0.12(0.08-0.17)	N/A	N/A	1.01(0.62-1.63)	0.12(0.04-0.36)	0.99(0.59-1.65)	0.82(0.39-1.71)	0.32(0.20-0.51)	0.15(0.10-0.23)
Non-f2f contacts	2.53(2.03-3.15)	N/A	N/A	5.14(3.12-8.48)	30.64(3.9-67.51)	2.73(1.65-4.52)	8.87(4.10-19.15)	2.42(1.46-4.00)	3.02(2.05-4.47)
F2f and Non-f2f contacts	0.89(0.71-1.12)	N/A	N/A	1.41(0.87-2.29)	1.43(0.71-2.91)	1.15(0.69-1.93)	1.14(0.58-2.24)	1.25(0.77-2.04)	1.28(0.87-1.86)
Cancelled/DNA	0.83(0.66-1.04)	N/A	N/A	1.36(0.84-2.21)	0.62(0.13-2.98)	0.56(0.35-0.89)	2.91(0.95-8.96)	0.78(0.46-1.31)	0.80(0.54-1.20)
Caseload	1.02(1.00-1.04)	N/A	N/A	1.02(1.00-1.04)	0.83(0.82-0.85)	0.97(0.96-0.99)	N/A	0.94(0.93-0.95)	0.99(0.98-0.99)
EIP									
F2f contacts	0.19(0.15-0.26)	0.20(0.12-0.31)	N/A	1.06(0.61-1.87)	0.38(0.21-0.68)	0.16(0.09-0.30)	N/A	0.34(0.22-0.53)	0.16(0.12-0.23)
Non-f2f contacts	2.35(1.78-3.10)	3.71(2.49-5.51)	N/A	2.61(1.46-4.68)	7.95(4.33-14.60)	18.15(4.96-66.42)	N/A	2.17(1.50-3.14)	6.48(4.23-9.94)
F2f and Non-f2f contacts	0.97(0.76-1.23)	1.11(0.74-1.64)	N/A	1.34(0.77-2.34)	1.60(0.92-2.79)	0.47(0.26-0.84)	N/A	1.17(0.81-1.71)	1.24(0.97-1.58)
Cancelled/DNA	0.52(0.40-0.67)	1.30(0.80-2.13)	N/A	1.16(0.66-2.03)	0.38(0.19-0.78)	0.49(0.27-0.89)	N/A	1.10(0.72-1.70)	0.84(0.54-1.32)
Caseload	1.04(1.02-1.05)	1.01(1.00-1.02)	N/A	1.02(1.01-1.03)	1.01(1.00-1.02)	1.06(1.05-1.07)	N/A	1.04(1.03-1.05)	1.09(1.08-1.10)
HTT									
F2f contacts	0.47(0.44-0.51)	0.30(0.26-0.35)	N/A	0.75(0.69-0.83)	0.67(0.58-0.78)	0.48(0.41-0.57)	N/A	0.46(0.39-0.54)	0.15(0.11-0.20)
Non-f2f contacts	1.95(1.63-2.34)	2.06(1.80-2.35)	N/A	1.42(1.07-1.89)	2.79(1.73-4.50)	1.43(1.06-1.94)	N/A	1.64(1.45-1.86)	1.67(1.16-2.42)
F2f and Non-f2f contacts	0.76(0.71-0.81)	0.94(0.86-1.04)	N/A	0.83(0.77-0.90)	0.83(0.73-0.95)	0.65(0.56-0.76)	N/A	1.08(0.94-1.23)	0.38(0.28-0.52)
Cancelled/DNA	1.00(0.81-1.24)	0.97(0.78-1.22)	N/A	0.77(0.59-1.00)	0.48(0.33-0.70)	0.48(0.33-0.70)	N/A	0.94(0.76-1.16)	0.29(0.18-0.46)
Caseload	0.74(0.72-0.76)	0.56(0.53-0.59)	N/A	0.58(0.55-0.61)	0.42(0.39-0.45)	0.70(0.65-0.75)	N/A	0.98(0.97-0.99)	0.49(0.45-0.53)
Liaison									
F2f contacts	0.51(0.46-0.57)	0.34(0.24-0.49)	N/A	0.60(0.49-0.74)	0.36(0.27-0.47)	0.49(0.34-0.70)	0.38(0.28-0.51)	0.48(0.42-0.55)	0.29(0.23-0.37)
Non-f2f contacts	1.47(1.13-1.93)	1.69(0.95-2.99)	N/A	0.85(0.65-1.11)	1.22(0.51-2.89)	1.83(0.91-3.69)	3.37(1.77-6.42)	1.47(1.08-1.99)	1.63(1.16-2.29)

For peer review only

F2f and Non-f2f contacts	0.61(0.54-0.68)	0.53(0.37-0.75)	N/A	0.65(0.53-0.80)	0.39(0.29-0.51)	0.66(0.46-0.94)	0.74(0.56-0.98)	0.61(0.54-0.69)	0.59(0.49-0.70)
Cancelled/DNA	0.31(0.21-0.47)	0.41(0.13-1.33)	N/A	1.23(0.76-1.99)	0.15(0.02-1.35)	0.85(0.49-1.50)	2.05(0.80-5.24)	0.21(0.11-0.39)	1.04(0.67-1.63)
Caseload	0.91(0.90-0.92)	0.35(0.31-0.39)	N/A	0.92(0.91-0.93)	0.45(0.42-0.50)	0.82(0.80-0.84)	N/A	1.03(1.00-1.06)	0.92(0.90-0.93)
OA									
F2f contacts	0.28(0.20-0.38)	0.18(0.10-0.31)	N/A	1.21(0.70-2.11)	0.58(0.33-1.03)	0.19(0.11-0.33)	0.22(0.11-0.45)	0.40(0.24-0.65)	0.18(0.12-0.28)
Non-f2f contacts	1.84(1.44-2.36)	4.45(2.54-7.79)	N/A	3.22(1.86-5.57)	9.68(4.70-19.93)	3.78(2.27-6.31)	7.98(3.03-20.99)	2.40(1.41-4.09)	3.43(2.51-4.69)
F2f and Non-f2f contacts	0.74(0.59-0.94)	1.34(0.79-2.29)	N/A	1.77(1.02-3.08)	1.51(0.85-2.66)	0.63(0.38-1.03)	0.80(0.41-1.55)	1.17(0.71-1.92)	1.20(0.85-1.70)
Cancelled/DNA	0.60(0.37-0.99)	1.56(0.90-2.71)	N/A	1.52(0.87-2.65)	0.65(0.24-1.77)	1.85(1.17-2.93)	0.41(0.15-1.16)	0.73(0.47-1.14)	1.77(1.38-2.27)
Caseload	0.91(0.91-0.92)	1.00(1.00-1.00)	N/A	1.00(0.99-1.02)	0.92(0.91-0.93)	0.96(0.94-0.98)	N/A	0.94(0.93-0.95)	0.98(0.97-0.98)

AMH: adult mental health service; CAMHS: child and adolescent mental health service; EIP: early intervention for psychosis service; HTT: home treatment team; OA: older adult service. N/A: data not available for these type of services

For peer review only

Supplementary Table 2: Site-specific data for lift-of-lockdown changes in daily mortality and service activity

	IRR (95%CI) for lift of lockdown vs. lockdown comparisons								
**Measures**	**Site A**	**Site B**	**Site C**	**Site D**	**Site E**	**Site F**	**Site G**	**Site H**	**Site J**
Number of deaths	0.42(0.32-0.55)	0.69(0.59-0.81)	0.12(0.05-3.56)	0.14(0.10-0.22)	0.08(0.02-0.40)	1.06(0.79-1.43)	0.21(0.06-0.68)	0.56(0.32-0.98)	0.63(0.46-0.87)
Trust-wide activity									
Number of new referrals accepted	1.32(0.92-1.89)	1.55(0.90-2.65)	N/A	1.39(0.88-2.18)	N/A	1.33(1.02-1.74)	N/A	1.49(0.85-2.61)	1.21(0.70-2.08)
Number of discharges	0.90(0.71-1.15)	1.10(0.62-1.95)	N/A	0.97(0.58-1.60)	N/A	0.97(0.71-1.32)	N/A	1.40(0.66-2.96)	0.83(0.50-1.40)
Inpatient care									
New admissions	1.08(0.79-1.47)	1.47(0.95-2.27)	1.32(0.87-2.00)	1.27(0.84-1.91)	1.17(0.63-2.15)	0.87(0.48-1.58)	N/A	1.34(0.88-2.04)	1.18(0.66-2.12)
Discharges	0.51(0.34-0.76)	1.04(0.55-1.97)	0.90(0.48-1.72)	0.69(0.32-1.51)	0.44(0.16-1.20)	0.44(0.20-0.99)	N/A	0.60(0.29-1.23)	0.96(0.50-1.85)
Inpatient caseload	1.01(0.99-1.04)	1.14(1.13-1.16)	1.04(1.03-1.06)	0.94(0.93-0.95)	1.11(1.08-1.14)	1.04(1.00-1.07)	N/A	1.14(1.12-1.16)	1.08(1.07-1.09)
Inpatient caseload (MHA)	1.04(1.02-1.07)	1.08(1.07-1.10)	N/A	0.92(0.92-0.93)	N/A	1.07(1.03-1.11)	N/A	0.87(0.84-0.89)	1.09(1.08-1.10)
AMH (community)									
F2f contacts	0.82(0.59-1.15)	1.31(0.62-2.76)	N/A	1.23(0.52-2.91)	1.02(0.43-2.43)	0.91(0.35-2.40)	1.17(0.12-11.37)	1.16(0.67-2.00)	1.13(0.65-1.96)
Non-f2f contacts	1.35(1.07-1.70)	1.33(0.72-2.46)	N/A	1.30(0.53-3.22)	0.72(0.30-1.71)	0.62(0.25-1.49)	2.29(0.20-26.05)	1.15(0.56-2.38)	1.46(0.87-2.45)
F2f and Non-f2f contacts	1.10(0.88-1.38)	1.29(0.64-2.59)	N/A	1.23(0.52-2.91)	0.92(0.40-2.16)	0.78(0.31-1.97)	2.61(0.23-29.41)	1.14(0.61-2.14)	1.32(0.78-2.25)
Cancelled/DNA	1.24(0.95-1.62)	1.02(0.47-2.21)	N/A	0.96(0.41-2.26)	0.44(0.16-1.22)	0.49(0.21-1.16)	1.91(0.20-17.82)	1.17(0.56-2.46)	0.95(0.49-1.86)
Caseload	1.01(1.01-1.02)	1.01(1.00-1.01)	N/A	1.00(1.00-1.01)	1.01(1.01-1.01)	1.03(1.02-1.03)	N/A	1.01(1.00-1.01)	1.01(1.01-1.01)
CAMHS									
F2f contacts	1.29(0.60-2.80)	N/A	N/A	1.16(0.54-2.48)	2.25(0.56-9.08)	1.59(0.66-3.81)	0.30(0.04-2.12)	1.41(0.71-2.80)	0.58(0.28-1.19)
Non-f2f contacts	1.32(1.03-1.70)	N/A	N/A	1.04(0.46-2.36)	0.52(0.04-6.70)	0.80(0.40-1.60)	2.58(0.43-15.31)	1.38(0.63-3.03)	1.09(0.59-2.01)
F2f and Non-f2f contacts	1.25(0.93-1.67)	N/A	N/A	1.14(0.53-2.46)	1.33(0.48-3.68)	1.43(0.61-3.32)	0.47(0.07-2.98)	1.41(0.68-2.92)	0.93(0.53-1.66)
Cancelled/DNA	1.12(0.87-1.46)	N/A	N/A	0.73(0.33-1.63)	N/A	1.42(0.65-3.13)	0.07(0.01-0.64)	0.99(0.45-2.15)	0.85(0.44-1.65)
Caseload	0.99(0.99-0.99)	N/A	N/A	1.00(0.99-1.00)	1.11(1.09-1.12)	0.96(0.96-0.96)	N/A	0.98(0.97-0.98)	0.99(0.99-0.99)
EIP									
F2f contacts	1.16(0.85-1.60)	2.56(1.35-4.84)	N/A	1.11(0.47-2.65)	1.36(0.56-3.30)	3.92(1.77-8.72)	N/A	1.37(0.69-2.72)	1.70(1.05-2.76)
Non-f2f contacts	1.67(1.23-2.27)	1.30(0.68-2.48)	N/A	1.08(0.45-2.61)	0.54(0.22-1.34)	1.72(0.17-16.98)	N/A	1.41(0.80-2.49)	3.05(1.80-5.16)
F2f and Non-f2f contacts	1.37(1.04-1.81)	1.61(0.87-2.95)	N/A	1.09(0.46-2.57)	0.79(0.34-1.87)	2.02(0.92-4.41)	N/A	1.29(0.71-2.37)	1.64(1.22-2.22)
Cancelled/DNA	1.16(0.88-1.54)	0.74(0.36-1.55)	N/A	1.03(0.44-2.45)	0.24(0.09-0.65)	1.34(0.53-3.36)	N/A	1.27(0.67-2.39)	1.20(0.72-2.01)
Caseload	0.99(0.99-1.00)	1.06(1.04-1.07)	N/A	1.01(1.00-1.01)	1.00(1.00-1.00)	1.04(1.03-1.05)	N/A	1.10(1.10-1.11)	1.05(1.04-1.06)
HTT									
F2f contacts	1.55(1.37-1.75)	1.90(1.56-2.31)	N/A	1.40(1.21-1.61)	1.38(1.16-1.65)	1.38(1.13-1.67)	N/A	1.47(1.18-1.83)	1.15(0.73-1.81)
Non-f2f contacts	1.21(0.93-1.57)	1.11(0.93-1.34)	N/A	1.57(1.10-2.23)	0.61(0.33-1.13)	0.82(0.57-1.20)	N/A	0.86(0.72-1.02)	1.85(1.03-3.31)
F2f and Non-f2f contacts	1.37(1.24-1.52)	1.36(1.18-1.56)	N/A	1.39(1.22-1.59)	1.21(1.03-1.43)	1.19(0.97-1.46)	N/A	0.98(0.81-1.18)	1.42(0.92-2.17)
Cancelled/DNA	1.09(0.78-1.52)	1.34(0.97-1.85)	N/A	1.88(1.30-2.72)	1.49(0.76-2.92)	3.00(1.72-5.21)	N/A	0.93(0.67-1.27)	1.91(0.87-4.23)
Caseload	1.22(1.18-1.27)	1.26(1.17-1.36)	N/A	1.23(1.16-1.31)	1.35(1.24-1.46)	1.25(1.14-1.36)	N/A	0.93(0.91-0.94)	0.35(0.33-0.38)
Liaison									
F2f contacts	1.81(1.52-2.14)	2.11(1.22-3.64)	N/A	1.48(1.06-2.05)	1.58(1.04-2.42)	1.50(0.88-2.56)	0.98(0.53-1.82)	1.40(1.17-1.67)	2.67(1.93-3.67)
Non-f2f contacts	1.31(0.91-1.89)	0.59(0.25-1.36)	N/A	1.12(0.74-1.69)	2.62(0.79-8.69)	6.11(1.58-23.63)	1.09(0.35-3.40)	0.74(0.48-1.14)	0.93(0.57-1.52)
F2f and Non-f2f contacts	1.68(1.43-1.97)	1.51(0.88-2.60)	N/A	1.37(0.98-1.92)	1.57(1.02-2.43)	1.80(1.02-3.18)	0.87(0.45-1.67)	1.24(1.04-1.47)	1.55(1.21-1.99)

For peer review only

Cancelled/DNA	1.39(0.70-2.76)	1.04(0.10-10.5)	N/A	0.92(0.46-1.82)	6.97(0.41-117.19)	0.44(0.19-1.02)	0.13(0.01-1.21)	0.12(0.02-0.89)	0.83(0.51-1.35)
Caseload	1.10(1.08-1.12)	1.19(1.02-1.39)	N/A	0.99(0.98-1.01)	1.90(1.71-2.11)	1.16(1.12-1.20)	N/A	1.04(1.01-1.06)	1.00(0.98-1.01)
OA									
F2f contacts	1.98(1.37-2.86)	1.09(0.46-2.61)	N/A	1.65(0.69-3.96)	1.18(0.45-3.12)	0.76(0.34-1.72)	0.68(0.12-3.67)	1.20(0.53-2.71)	1.25(0.65-2.40)
Non-f2f contacts	1.20(0.87-1.66)	1.34(0.58-3.10)	N/A	0.91(0.38-2.21)	1.23(0.39-3.92)	1.34(0.65-2.78)	0.45(0.05-3.91)	1.37(0.61-3.08)	1.65(0.99-2.75)
F2f and Non-f2f contacts	1.46(1.07-1.99)	1.20(0.52-2.77)	N/A	1.28(0.53-3.06)	1.15(0.46-2.89)	0.88(0.41-1.87)	0.65(0.12-3.46)	1.16(0.53-2.56)	1.47(0.85-2.53)
Cancelled/DNA	2.15(1.10-4.17)	0.70(0.29-1.70)	N/A	0.82(0.34-2.01)	3.36(0.49-23.00)	0.15(0.07-0.34)	0.41(0.04-4.20)	0.64(0.29-1.43)	0.58(0.37-0.91)
Caseload	0.91(0.90-0.92)	0.99(0.98-0.99)	N/A	0.95(0.95-0.96)	1.11(1.09-1.13)	0.99(0.97-1.01)	N/A	0.99(0.98-1.00)	0.98(0.98-0.99)

AMH: adult mental health service; CAMHS: child and adolescent mental health service; EIP: early intervention for psychosis service; HTT: home treatment team; OA: older adult service. N/A: data not available for these type of services

For peer review only

Supplementary Table 3: Site-specific data for lift-of-lockdown vs. pre-lockdown changes in daily mortality and service activity

	IRR (95%CI) for lift of lockdown vs. pre-lockdown comparisons								
**Measures**	**Site A**	**Site B**	**Site C**	**Site D**	**Site E**	**Site F**	**Site G**	**Site H**	**Site J**
Number of deaths	1.46(1.15-1.85)	1.32(1.15-1.53)	0.49(0.02-12.44)	0.31(0.22-0.46)	0.43(0.09-2.00)	1.64(1.29-2.09)	0.50(0.16-1.58)	0.87(0.52-1.45)	1.42(1.10-1.82)
Trust-wide activity									
Number of new referrals accepted	0.85(0.72-0.99)	1.00(0.63-1.58)	N/A	0.85(0.58-1.26)	N/A	0.83(0.67-1.04)	N/A	0.92(0.57-1.49)	0.73(0.46-1.15)
Number of discharges	0.87(0.75-1.01)	1.19(0.73-1.96)	N/A	0.96(0.63-1.47)	N/A	0.86(0.67-1.11)	N/A	1.03(0.54-1.98)	0.88(0.54-1.41)
Inpatient care									
New admissions	0.88(0.66-1.15)	1.13(0.78-1.63)	0.76(0.53-1.09)	1.00(0.71-1.42)	0.84(0.52-1.36)	0.66(0.39-1.12)	N/A	0.97(0.68-1.40)	0.90(0.53-1.53)
Discharges	0.64(0.47-0.86)	1.10(0.63-1.89)	0.83(0.51-1.35)	0.86(0.43-1.69)	0.44(0.18-1.08)	0.49(0.23-1.03)	N/A	0.89(0.48-1.63)	0.89(0.50-1.56)
Inpatient caseload	0.78(0.77-0.79)	0.94(0.93-0.95)	0.95(0.94-0.96)	0.91(0.90-0.92)	0.87(0.85-0.89)	0.69(0.67-0.71)	N/A	1.01(1.00-1.02)	1.08(1.07-1.09)
Inpatient caseload (MHA)	0.88(0.87-0.89)	1.01(1.00-1.02)	N/A	0.93(0.92-0.93)	N/A	0.83(0.81-0.85)	N/A	0.86(0.84-0.88)	1.14(1.13-1.15)
AMH (community)									
F2f contacts	0.44(0.34-0.56)	0.57(0.30-1.09)	N/A	1.48(0.70-3.14)	0.73(0.34-1.55)	0.40(0.17-0.97)	0.43(0.05-3.58)	0.67(0.41-1.08)	0.46(0.29-0.76)
Non-f2f contacts	2.88(2.43-3.41)	4.20(2.46-7.17)	N/A	4.08(1.84-9.05)	2.18(1.02-4.64)	5.17(2.29-11.67)	18.87(1.87-90.10)	2.57(1.37-4.84)	7.79(5.04-12.04)
F2f and Non-f2f contacts	1.13(0.96-1.33)	1.56(0.85-2.85)	N/A	1.70(0.80-3.61)	1.02(0.49-2.14)	0.67(0.29-1.55)	1.33(0.14-12.90)	1.36(0.78-2.35)	1.53(0.95-2.44)
Cancelled/DNA	0.65(0.56-0.75)	1.26(0.65-2.46)	N/A	1.23(0.59-2.60)	0.20(0.08-0.50)	0.58(0.29-1.14)	1.04(0.13-8.21)	1.32(0.70-2.50)	0.89(0.50-1.59)
Caseload	1.04(1.03-1.04)	1.04(1.04-1.04)	N/A	1.02(1.02-1.02)	1.01(1.01-1.01)	1.04(1.04-1.05)	N/A	1.03(1.03-1.03)	1.07(1.07-1.07)
CAMHS									
F2f contacts	0.15(0.07-0.32)	N/A	N/A	1.16(0.60-2.26)	0.27(0.09-0.77)	1.57(0.72-3.40)	0.24(0.04-1.55)	0.45(0.25-0.82)	0.09(0.05-0.16)
Non-f2f contacts	3.35(2.76-4.06)	N/A	N/A	5.36(2.62-10.99)	15.89(2.94-85.84)	2.19(1.21-3.96)	22.85(4.40-81.80)	3.33(1.69-6.56)	3.30(1.93-5.65)
F2f and Non-f2f contacts	1.11(0.89-1.38)	N/A	N/A	1.61(0.82-3.15)	1.91(0.79-4.59)	1.64(0.79-3.43)	0.53(0.09-3.06)	1.77(0.95-3.27)	1.19(0.72-1.96)
Cancelled/DNA	0.93(0.78-1.11)	N/A	N/A	1.00(0.49-2.02)	N/A	0.79(0.39-1.60)	0.22(0.03-1.40)	0.77(0.40-1.48)	0.69(0.38-1.23)
Caseload	1.01(1.01-1.01)	N/A	N/A	1.02(1.01-1.02)	0.92(0.91-0.94)	0.93(0.92-0.94)	N/A	0.91(0.90-0.92)	0.97(0.97-0.97)
EIP									
F2f contacts	0.23(0.19-0.28)	0.50(0.30-0.84)	N/A	1.19(0.57-2.49)	0.52(0.24-1.09)	0.63(0.34-1.15)	N/A	0.47(0.26-0.84)	0.28(0.18-0.42)
Non-f2f contacts	3.91(3.18-4.82)	4.83(2.69-8.67)	N/A	2.82(1.29-6.16)	4.33(2.00-9.38)	31.28(3.75-60.97)	N/A	3.07(1.88-5.00)	19.75(12.4-31.2)

For peer review only

F2f and Non-f2f contacts	1.33(1.12-1.58)	1.78(1.04-3.02)	N/A	1.46(0.70-3.06)	1.27(0.61-2.64)	0.95(0.51-1.76)	N/A	1.52(0.90-2.58)	2.03(1.64-2.53)
Cancelled/DNA	0.60(0.51-0.71)	0.97(0.52-1.81)	N/A	1.20(0.57-2.51)	0.09(0.04-0.21)	0.66(0.30-1.41)	N/A	1.40(0.82-2.40)	1.02(0.71-1.44)
Caseload	1.04(1.03-1.04)	1.07(1.06-1.08)	N/A	1.03(1.02-1.03)	1.01(1.00-1.01)	1.10(1.09-1.11)	N/A	1.15(1.14-1.15)	1.15(1.14-1.15)
HTT									
F2f contacts	0.73(0.66-0.82)	0.57(0.49-0.66)	N/A	1.05(0.93-1.19)	0.93(0.82-1.06)	0.67(0.58-0.77)	N/A	0.68(0.56-0.82)	0.17(0.11-0.25)
Non-f2f contacts	2.36(1.87-2.97)	2.29(1.95-2.70)	N/A	2.23(1.64-3.02)	1.69(1.03-2.80)	1.18(0.88-1.59)	N/A	1.41(1.22-1.63)	3.09(1.81-5.28)
F2f and Non-f2f contacts	1.04(0.96-1.14)	1.28(1.14-1.44)	N/A	1.16(1.03-1.30)	1.01(0.88-1.15)	0.78(0.67-0.91)	N/A	1.06(0.91-1.23)	0.55(0.39-0.77)
Cancelled/DNA	1.09(0.81-1.46)	1.31(1.00-1.71)	N/A	1.44(1.07-1.94)	0.72(0.39-1.31)	1.44(0.90-2.32)	N/A	0.87(0.65-1.15)	0.55(0.28-1.10)
Caseload	0.91(0.88-0.94)	0.71(0.67-0.75)	N/A	0.71(0.68-0.75)	0.56(0.52-0.60)	0.87(0.80-0.94)	N/A	0.91(0.89-0.92)	0.17(0.16-0.18)
Liaison									
F2f contacts	0.93(0.80-1.08)	0.72(0.45-1.15)	N/A	0.89(0.67-1.18)	0.56(0.39-0.81)	0.73(0.47-1.14)	0.37(0.21-0.64)	0.67(0.59-0.77)	0.78(0.60-1.01)
Non-f2f contacts	1.93(1.41-2.65)	0.99(0.47-2.06)	N/A	0.95(0.67-1.35)	3.18(1.11-9.11)	11.20(3.05-41.19)	3.67(1.36-9.94)	1.09(0.76-1.56)	1.51(0.97-2.37)
F2f and Non-f2f contacts	1.02(0.89-1.16)	0.80(0.50-1.28)	N/A	0.89(0.67-1.19)	0.61(0.42-0.88)	1.18(0.72-1.93)	0.64(0.35-1.18)	0.75(0.65-0.86)	0.91(0.74-1.14)
Cancelled/DNA	0.44(0.24-0.81)	0.42(0.05-3.45)	N/A	1.13(0.65-1.98)	1.06(0.15-7.26)	0.38(0.19-0.76)	0.26(0.03-2.06)	0.02(0.00-0.19)	0.87(0.56-1.33)
Caseload	1.00(0.98-1.02)	0.41(0.36-0.47)	N/A	0.91(0.90-0.92)	0.86(0.79-0.94)	0.95(0.92-0.99)	N/A	1.06(1.03-1.10)	0.91(0.90-0.92)
OA									
F2f contacts	0.55(0.44-0.70)	0.20(0.09-0.42)	N/A	2.00(0.94-4.26)	0.69(0.29-1.63)	0.15(0.07-0.29)	0.15(0.03-0.72)	0.48(0.23-0.98)	0.23(0.13-0.40)
Non-f2f contacts	2.21(1.73-2.83)	5.98(2.9-12.2)	N/A	2.94(1.36-6.38)	11.92(4.31-32.98)	5.08(2.75-9.39)	3.56(0.49-26.01)	3.28(1.64-6.58)	5.66(3.58-8.95)
F2f and Non-f2f contacts	1.09(0.87-1.36)	1.61(0.78-3.33)	N/A	2.26(1.06-4.84)	1.73(0.77-3.90)	0.55(0.29-1.05)	0.52(0.11-2.47)	1.36(0.68-2.70)	1.76(1.10-2.82)
Cancelled/DNA	1.30(0.76-2.20)	1.09(0.50-2.37)	N/A	1.25(0.57-2.73)	2.19(0.39-12.32)	0.28(0.13-0.58)	0.17(0.02-1.41)	0.47(0.23-0.97)	1.03(0.67-1.57)
Caseload	0.83(0.83-0.84)	0.99(0.99-0.99)	N/A	0.95(0.95-0.95)	1.02(1.01-1.03)	0.95(0.94-0.96)	N/A	0.93(0.92-0.94)	0.96(0.96-0.96)

AMH: adult mental health service; CAMHS: child and adolescent mental health service; EIP: early intervention for psychosis service; HTT: home treatment team; OA: older adult service. N/A: data not available for these type of services

For peer review only

For peer review only - http://bmjopen.bmj.com/site/about/guidelines.xhtml

Supplementary Table 4. Meta-analysed effects of lockdown announcements on mortality and mental health service use activity omitting one week around the two transition dates (23rd March and 10th May).

	Lockdown announcement		Lift of Lockdown announcement		Lift of Lockdown vs. Pre-lockdown	
**Measures**	IRR (95%CI)	I^2	IRR (95%CI)	I^2	IRR (95%CI)	I^2
Number of deaths	2.40*(2.02-2.84)	67.5	0.33*(0.18-0.60)	72.7	0.79 (0.46-1.33)	63.8
Trust-wide activity						
Number of new referrals accepted	0.69*(0.60-0.79)	0.0	1.68*(1.21-2.35)	0.0	1.09 (0.81-1.47)	0.0
Number of discharges	0.86*(0.77-0.97)	0.0	1.34 (0.91-1.98)	0.0	1.23 (0.86-1.76)	0.0
Inpatient care						
New admissions	0.82^(0.68-0.98)	49.8	1.62*(1.21-2.17)	0.0	1.14 (0.80-1.63)	39.3
Discharges	0.96 (0.80-1.16)	0.0	1.23 (0.69-2.18)	26.5	1.25 (0.78-1.99)	13.4
Inpatient caseload	0.84*(0.76-0.92)	99.8	1.13*(1.06-1.21)	98.3	0.95 (0.89-1.01)	99.2
Inpatient caseload on MHA section	0.96 (0.89-1.03)	99.5	0.99 (0.90-1.10)	98.8	0.95 (0.85-1.07)	99.3
AMH (community)						
F2f contacts	0.55*(0.44-0.69)	14.8	1.97*(1.15-3.37)	0.0	1.19 (0.73-1.95)	0.0
Non-f2f contacts	4.54*(2.84-7.25)	85.7	1.52 (0.94-2.45)	0.0	5.35*(3.43-8.35)	0.0
F2f and Non-f2f contacts	1.23*(1.06-1.44)	0.0	1.90*(1.16-3.10)	0.0	2.37*(1.50-3.75)	0.0
Cancelled appointments or DNAs	0.74 (0.54-1.02)	59.3	1.85*(1.05-3.26)	0.0	1.57 (0.92-2.66)	0.0
Caseload	1.05*(1.03-1.07)	98.6	1.01 (1.00-1.02)	89.4	1.04^(1.02-1.06)	98.3
CAMHS						
F2f contacts	0.38*(0.16-0.91)	90.9	1.94 (0.95-3.97)	0.0	0.86 (0.45-1.64)	0.0
Non-f2f contacts	4.67*(3.34-6.53)	57.5	0.98 (0.48-1.99)	21.1	4.87*(1.99-11.89)	52.5
F2f and Non-f2f contacts	1.46*(1.22-1.75)	0.0	1.71 (0.95-3.07)	0.0	2.55*(1.48-4.41)	0.0
Cancelled appointments or DNAs	0.80 (0.64-1.01)	17.8	1.29 (0.67-2.46)	0.0	1.03 (0.57-1.88)	0.0
Caseload	0.92*(0.89-0.96)	98.8	0.98 (0.96-1.00)	98.3	0.94^(0.89-0.99)	99.8
EIP						
F2f contacts	0.31*(0.21-0.45)	71.9	1.49 (0.85-2.61)	3.5	0.47 (0.21-1.04)	57.5
Non-f2f contacts	5.61*(3.08-10.22)	88.7	7.44 (0.63-48.53)	95.8	4.03*(3.58-14.05)	96.5
F2f and Non-f2f contacts	1.35*(1.17-1.56)	0.0	1.24 (0.78-1.98)	0.0	1.69*(1.10-2.61)	0.0
Cancelled appointments or DNAs	0.83 (0.58-1.20)	66.0	1.44 (0.73-2.85)	21.0	1.18 (0.48-2.92)	60.1
Caseload	1.06^(1.01-1.11)	99.8	1.03 (1.00-1.06)	99.3	1.09*(1.04-1.13)	99.6
HTT						
F2f contacts	0.45*(0.31-0.65)	97.1	1.49*(1.24-1.80)	39.0	0.76 (0.57-1.01)	76.3
Non-f2f contacts	1.90*(1.62-2.23)	58.8	1.05 (0.74-1.49)	63.1	2.02*(1.52-2.68)	54.9
F2f and Non-f2f contacts	0.88*(0.80-0.96)	66.1	1.27*(1.11-1.46)	26.8	1.14 (0.96-1.34)	52.8
Cancelled appointments or DNAs	0.71*(0.52-0.97)	82.2	1.27 (0.91-1.79)	29.5	1.05 (0.67-1.63)	64.2
Caseload	0.53*(0.36-0.79)	99.9	1.18(0.94-1.49)	98.9	0.63*(0.50-0.79)	99.4
Liaison						
F2f contacts	0.46*(0.36-0.60)	84.9	1.70*(1.34-2.17)	20.5	0.80*(0.67-0.94)	0.0
Non-f2f contacts	1.67*(1.15-2.41)	75.7	1.32 (0.90-1.93)	2.7	1.90*(1.19-3.04)	37.2
F2f and Non-f2f contacts	0.63*(0.52-0.76)	75.6	1.49*(1.10-2.01)	46.4	0.88 (0.74-1.05)	0.0
Cancelled appointments or DNAs	0.74 (0.45-1.22)	67.6	1.26 (0.51-3.12)	0.0	1.11 (0.42-2.88)	13.6
Caseload	0.77*(0.72-0.82)	98.7	1.19*(1.09-1.30)	95.9	0.87 (0.78-0.97)	97.8
OA						
F2f contacts	0.33*(0.21-0.53)	77.4	2.03*(1.09-3.78)	0.0	0.93 (0.45-1.89)	31.0
Non-f2f contacts	4.14*(2.69-6.37)	75.5	1.50 (0.83-2.71)	0.0	6.29*(2.95-13.41)	41.4
F2f and Non-f2f contacts	1.19 (0.86-1.64)	58.6	1.64 (0.92-2.95)	0.0	2.38*(1.23-4.60)	28.5
Cancelled appointments or DNAs	0.99 (0.69-1.42)	58.7	1.58 (0.74-3.39)	0.0	1.72 (0.85-3.49)	0.0
Caseload	0.91^(0.85-0.97)	99.8	1.02 (0.99-1.06)	99.3	0.94 (0.89-1.00)	99.7

^p<0.05; *p<0.001
AMH: adult mental health service; CAMHS: child and adolescent mental health service; EIP: early intervention for psychosis service; HTT: home treatment team; OA: older adult service.

For peer review only

Supplementary Table 5. Meta-analysed effects of lockdown announcements on mortality and mental health service use activity further adjusted for national covid-19 related deaths.

	Lockdown announcement		Lift of Lockdown announcement		Lift of Lockdown vs. Pre-lockdown	
**Measures**	IRR (95%CI)	I^2	IRR (95%CI)	I^2	IRR (95%CI)	I^2
Number of deaths	1.74*(1.23-2.46)	88.1	0.44*(0.24-0.78)	92.1	0.71 (0.42-1.21)	90.9
Trust-wide activity						
Number of new referrals accepted	0.69*(0.59-0.81)	0.0	1.28*(1.07-1.53)	0.0	0.90 (0.78-1.04)	0.0
Number of discharges	1.10 (0.95-1.29)	0.0	0.88 (0.75-1.04)	0.0	0.96 (0.84-1.11)	0.0
Inpatient care						
New admissions	0.71*(0.60-0.85)	22.9	1.18 (1.00-1.41)	0.0	0.86 (0.73-1.01)	7.1
Discharges	1.60*(1.32-1.94)	1.3	0.52*(0.41-0.66)	0.0	0.86 (0.69-1.08)	0.0
Inpatient caseload	0.89*(0.84-0.95)	99.4	1.03 (0.97-1.09)	99.2	0.91^(0.84-0.99)	99.8
Inpatient caseload on MHA section	0.95 (0.89-1.01)	98.3	0.99 (0.90-1.08)	99.4	0.93 (0.82-1.05)	99.8
AMH (community)						
F2f contacts	0.59*(0.45-0.77)	41.4	0.94 (0.76-1.16)	0.0	0.61*(0.43-0.86)	50.0
Non-f2f contacts	4.09*(2.55-6.55)	81.1	1.27*(1.05-1.52)	0.0	4.07*(2.62-6.32)	69.6
F2f and Non-f2f contacts	1.04 (0.90-1.20)	0.0	1.12 (0.94-1.32)	0.0	1.18 (0.99-1.40)	0.0
Cancelled appointments or DNAs	0.99 (0.66-1.47)	66.4	0.87 (0.62-1.23)	35.9	0.86 (0.60-1.23)	50.1
Caseload	1.01 (1.00-1.02)	85.1	1.01 (1.00-1.02)	95.1	1.04^(1.02-1.07)	98.8
CAMHS						
F2f contacts	0.36*(0.16-0.84)	92.1	1.11 (0.77-1.60)	16.7	0.38*(0.16-0.91)	86.3
Non-f2f contacts	4.05*(2.63-6.24)	74.2	1.23^(1.02-1.49)	0.0	3.97*(2.78-5.66)	47.7
F2f and Non-f2f contacts	1.12 (0.88-1.41)	26.2	1.22 (0.99-1.51)	0.0	1.24 (1.00-1.52)	2.1
Cancelled appointments or DNAs	1.09 (0.81-1.47)	45.7	0.94 (0.73-1.22)	15.8	0.98 (0.81-1.18)	0.0
Caseload	0.95 (0.92-0.99)	99.5	1.03 (0.90-1.19)	99.7	0.95 (0.91-1.00)	99.6
EIP						
F2f contacts	0.33*(0.21-0.51)	74.6	1.40^(1.02-1.91)	38.3	0.48*(0.30-0.75)	76.9
Non-f2f contacts	4.56*(2.57-8.11)	85.4	1.45 (0.97-2.17)	57.5	6.07*(2.92-12.60)	88.6
F2f and Non-f2f contacts	1.15 (0.89-1.48)	50.3	1.41*(1.20-1.66)	0.0	1.53*(1.20-1.94)	40.4
Cancelled appointments or DNAs	0.77 (0.48-1.26)	75.8	1.01 (0.74-1.37)	36.4	0.70 (0.38-1.29)	87.0
Caseload	1.04^(1.02-1.05)	93.6	1.03(0.99-1.07)	99.3	1.08^(1.03-1.13)	99.7
HTT						
F2f contacts	0.44*(0.34-0.58)	92.5	1.50*(1.39-1.61)	0.0	0.65*(0.51-0.83)	92.3
Non-f2f contacts	1.70*(1.48-1.95)	20.7	1.08 (0.83-1.41)	70.6	1.80*(1.34-2.40)	80.6
F2f and Non-f2f contacts	0.67*(0.53-0.84)	92.5	1.30*(1.14-1.47)	67.5	0.89 (0.75-1.06)	86.6
Cancelled appointments or DNAs	0.56*(0.37-0.83)	83.5	1.63*(1.08-2.44)	77.9	0.97 (0.78-1.21)	37.5
Caseload	0.65*(0.52-0.83)	99.5	0.95 (0.67-1.35)	99.8	0.62*(0.45-0.86)	99.7
Liaison						
F2f contacts	0.48*(0.41-0.55)	57.6	1.63*(1.38-1.93)	51.1	0.77*(0.66-0.91)	57.3
Non-f2f contacts	1.52*(1.07-2.15)	72.0	1.20 (0.83-1.75)	62.8	1.73*(1.18-2.54)	68.8
F2f and Non-f2f contacts	0.62*(0.55-0.70)	46.8	1.43*(1.22-1.68)	51.4	0.88 (0.77-1.01)	42.4
Cancelled appointments or DNAs	0.81 (0.39-1.71)	88.7	0.68 (0.39-1.16)	53.0	0.72 (0.43-1.21)	60.9
Caseload	0.86*(0.81-0.91)	97.8	1.13*(1.06-1.21)	98.2	0.96 (0.92-1.00)	95.7
OA						
F2f contacts	0.39*(0.23-0.66)	81.9	1.31 (0.95-1.80)	24.6	0.46*(0.26-0.85)	80.4
Non-f2f contacts	3.84*(2.43-6.08)	82.3	1.24 (1.00-1.52)	0.0	4.20*(2.61-6.77)	71.1
F2f and Non-f2f contacts	1.09 (0.80-1.48)	61.2	1.30*(1.06-1.60)	0.0	1.31 (0.93-1.85)	48.1
Cancelled appointments or DNAs	1.34 (0.90-1.99)	63.8	0.63 (0.33-1.23)	74.9	0.88 (0.49-1.57)	70.0
Caseload	0.97 (0.95-1.00)	98.8	0.98 (0.92-1.04)	99.7	0.95 (0.89-1.02)	99.8

^p<0.05; *p<0.001

AMH: adult mental health service; CAMHS: child and adolescent mental health service; EIP: early intervention for psychosis service; HTT: home treatment team; OA: older adult service.

For peer review only

For peer review only - http://bmjopen.bmj.com/site/about/guidelines.xhtml

Supplementary Table 6. Meta-analysed effects of the lockdown announcement on mortality and mental health service use activity with the use of equally spaced windows around the cut-off date of 23rd March

	Lockdown announcement		Lift of the Lockdown vs. Pre-lockdown	
**Measures**	IRR (95%CI)	I^2	IRR (95%CI)	I^2
Number of deaths	2.13 (1.96-2.31)	4.1	0.82 (0.55-1.21)	86.0
Trust-wide activity				
Number of new referrals accepted	0.65 (0.58-0.74)	0.0	0.88 (0.74-1.03)	0.0
Number of discharges	0.86 (0.77-0.96)	0.0	0.88 (0.74-1.04)	3.4
Inpatient care				
New admissions	0.79 (0.69-0.90)	24.2	0.97 (0.83-1.12)	0.0
Discharges	1.04 (0.88-1.23)	0.0	0.82 (0.65-1.03)	0.0
Inpatient caseload	0.85 (0.80-0.91)	99.4	0.93 (0.86-0.99)	99.8
Inpatient caseload on MHA section	0.95 (0.90-1.01)	98.8	0.94 (0.84-1.06)	99.8
AMH (community)				
F2f contacts	0.56 (0.45-0.70)	40.3	0.68 (0.53-0.88)	0.0
Non-f2f contacts	3.70 (2.63-5.20)	77.6	3.32 (2.50-4.42)	23.7
F2f and Non-f2f contacts	1.16 (1.02-1.33)	0.0	1.34 (1.07-1.69)	0.0
Cancelled appointments or DNAs	0.77 (0.59-1.02)	59.3	0.87 (0.60-1.26)	39.6
Caseload	1.05 (1.03-1.06)	93.8	1.04 (1.03-1.06)	97.7
CAMHS				
F2f contacts	0.37 (0.18-0.77)	90.5	0.40 (0.19-0.83)	71.3
Non-f2f contacts	3.89 (2.83-5.34)	60.3	3.63 (2.23-5.91)	56.5
F2f and Non-f2f contacts	1.32 (1.11-1.55)	0.0	1.37 (1.03-1.81)	0.0
Cancelled appointments or DNAs	0.83 (0.66-1.04)	26.0	0.79 (0.59-1.06)	0.0
Caseload	0.96 (0.93-0.99)	99.0	0.96 (0.92-1.00)	99.4
EIP				
F2f contacts	0.30 (0.20-0.46)	81.5	0.45 (0.31-0.66)	52.8
Non-f2f contacts	3.65 (2.39-5.59)	80.8	4.30 (2.73-6.78)	64.7
F2f and Non-f2f contacts	1.22 (1.05-1.40)	11.9	1.48 (1.19-1.84)	0.0
Cancelled appointments or DNAs	0.69 (0.47-1.01)	75.2	0.58 (0.29-1.16)	85.6
Caseload	1.06 (1.04-1.09)	98.3	1.10 (1.04-1.16)	99.7
HTT				
F2f contacts	0.44 (0.33-0.60)	96.3	0.70 (0.57-0.87)	88.3
Non-f2f contacts	1.69 (1.47-1.94)	53.6	1.62 (1.19-2.22)	88.1
F2f and Non-f2f contacts	0.82 (0.75-0.91)	74.7	0.97 (0.84-1.13)	82.8
Cancelled appointments or DNAs	0.74 (0.61-0.90)	68.5	1.10 (0.87-1.38)	54.9
Caseload	0.59 (0.46-0.77)	99.5	0.62 (0.46-0.85)	99.7
Liaison				
F2f contacts	0.50 (0.42-0.59)	77.2	0.75 (0.64-0.88)	47.7
Non-f2f contacts	1.56 (1.20-2.03)	64.6	1.50 (1.10-2.06)	52.3
F2f and Non-f2f contacts	0.66 (0.57-0.76)	70.3	0.84 (0.75-0.94)	10.8
Cancelled appointments or DNAs	0.62 (0.39-0.99)	69.4	1.08 (0.76-1.53)	0.0
Caseload	0.77 (0.70-0.84)	99.2	0.90 (0.82-1.00)	99.1
OA				
F2f contacts	0.34 (0.21-0.53)	82.7	0.48 (0.29-0.80)	64.7
Non-f2f contacts	3.39 (2.38-4.82)	72.2	3.60 (2.38-5.43)	49.3
F2f and Non-f2f contacts	1.10 (0.87-1.40)	46.8	1.32 (0.92-1.87)	38.2
Cancelled appointments or DNAs	0.90 (0.65-1.26)	64.5	0.66 (0.43-1.03)	39.8
Caseload	0.93 (0.91-0.96)	98.7	0.93 (0.87-1.00)	99.8

AMH: adult mental health service; CAMHS: child and adolescent mental health service; EIP: early intervention for psychosis service; HTT: home treatment team; OA: older adult service.

For peer review only - http://bmjopen.bmj.com/site/about/guidelines.xhtml

For peer review only

Supplementary Figure 1. Number of deaths per day before and after lockdown announcement for sites A, F, G and J during the period of 1st January 2020 to 31st July 2020. The vertical axis shows the daily number of deaths in the days before (orange dots) and after (red dots) the lockdown order was announced in 23rd March 2020 and after the lift (purple dots) of the lockdown order announced in 10th May. The green dots represent the number of daily deaths during the period of 1st January 2019 to 31st July 2019.

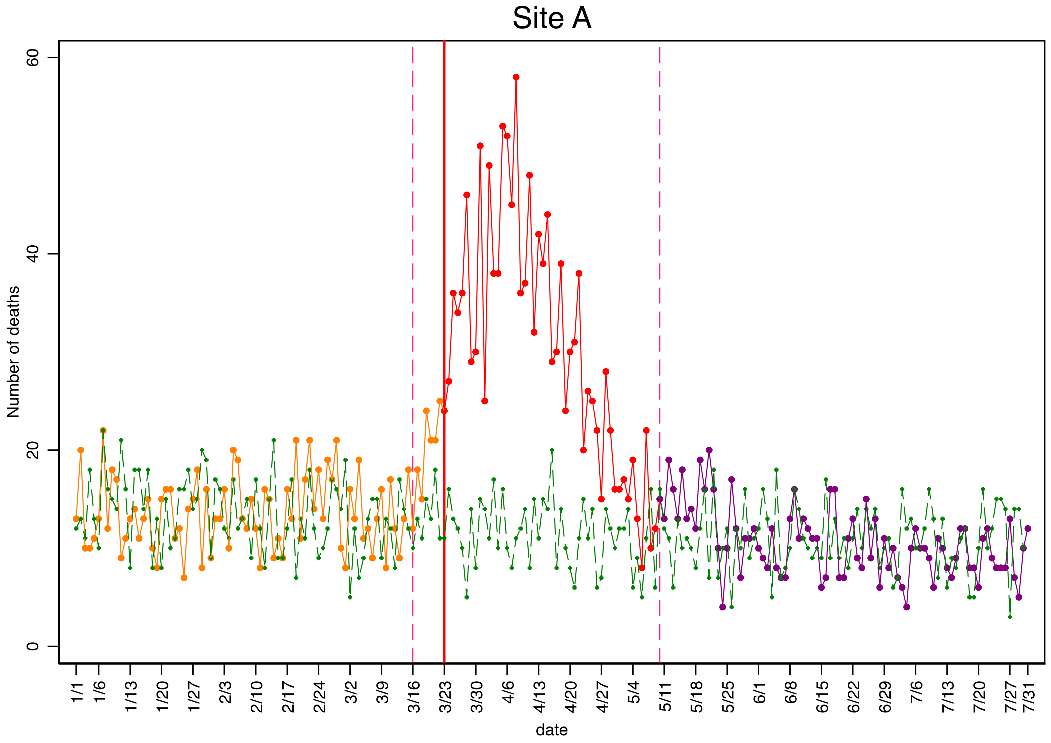

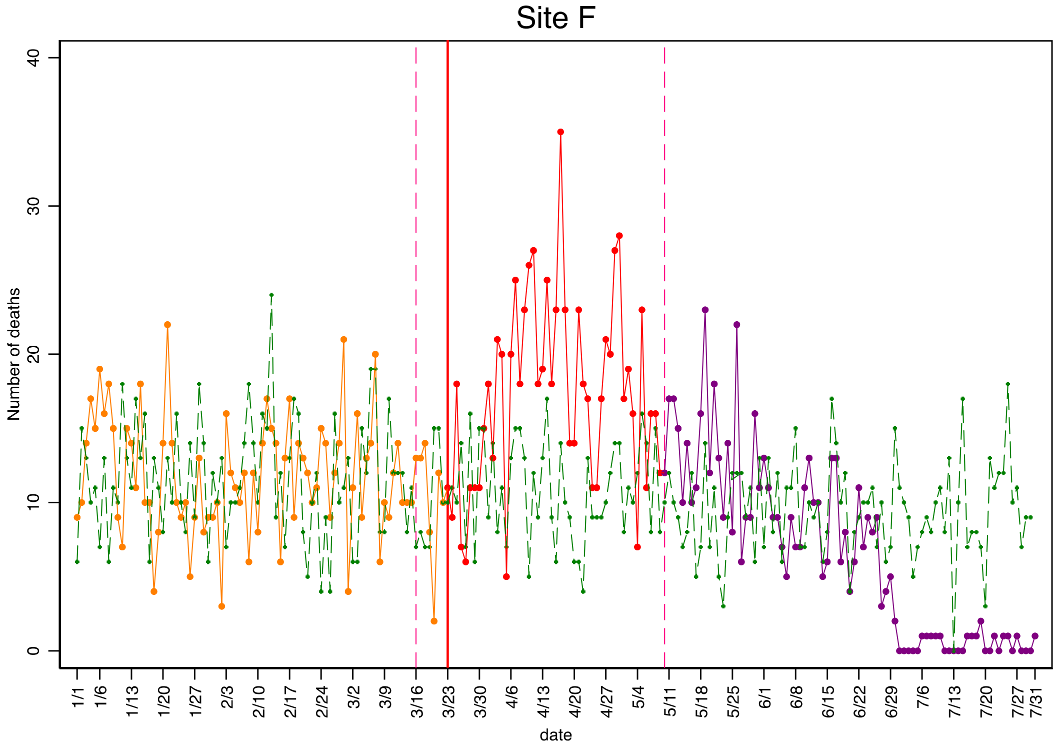

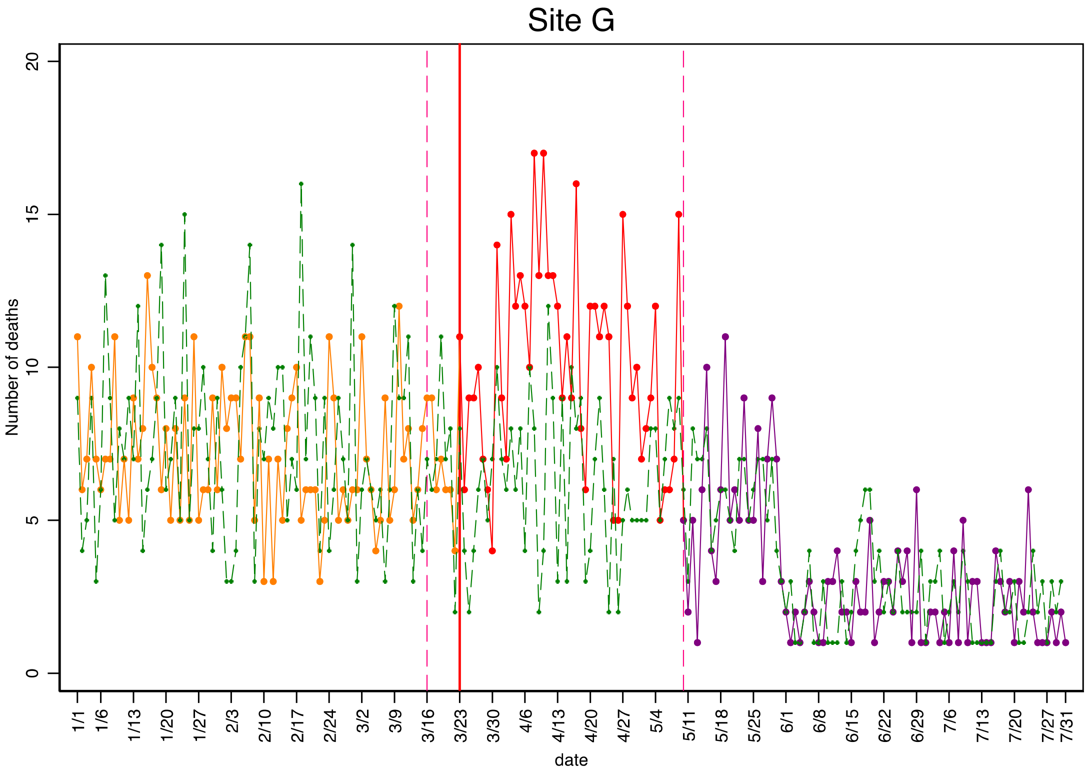
Site G
Number of deaths
0
5
10
15
20
1/1 1/6 1/13 1/20 1/27 2/3 2/10 2/17 2/24 3/2 3/9 3/16 3/23 3/30 4/6 4/13 4/20 4/27 5/4 5/11 5/18 5/25 6/1 6/8 6/15 6/22 6/29 7/6 7/13 7/20 7/27 7/31
date

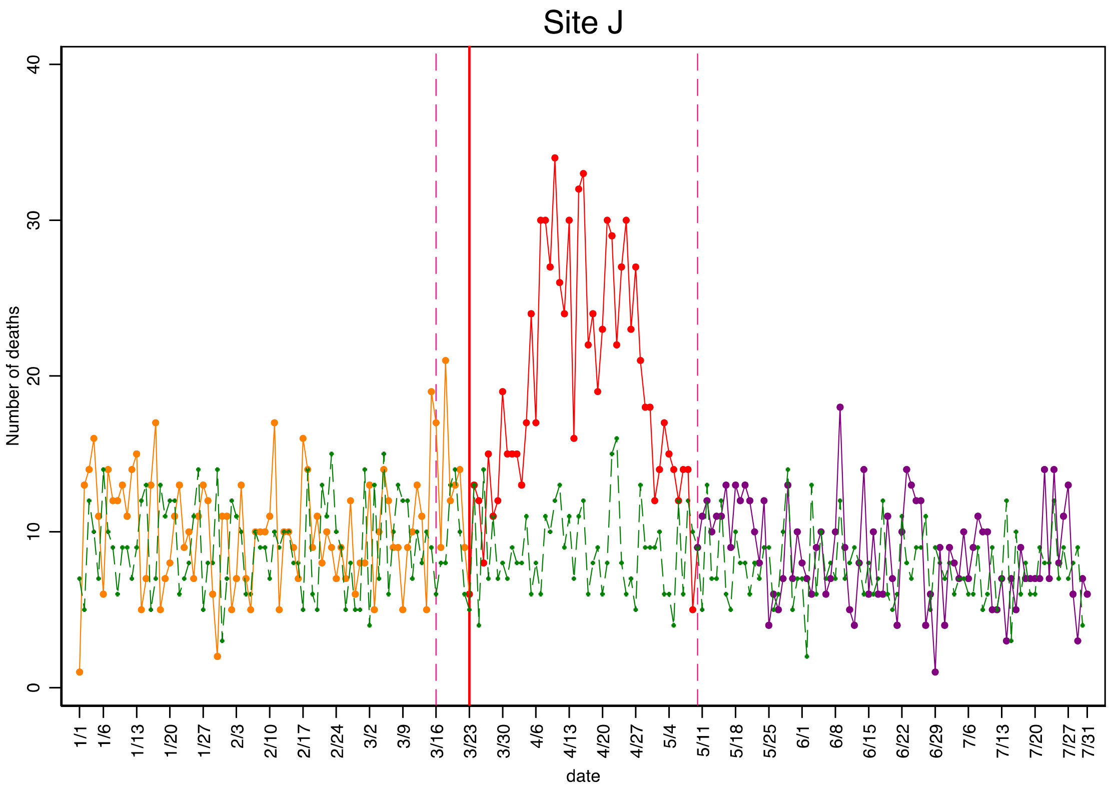
Site J
Number of deaths
0
10
20
30
40
1/1 1/6 1/13 1/20 1/27 2/3 2/10 2/17 2/24 3/2 3/9 3/16 3/23 3/30 4/6 4/13 4/20 4/27 5/4 5/11 5/18 5/25 6/1 6/8 6/15 6/22 6/29 7/6 7/13 7/20 7/27 7/31
date

Supplementary Figure 2. Number of deaths (A) and inpatient admissions (B) per week before and after lockdown announcement for site I during the period of 1st January 2020 to 5th July 2020. The vertical axis shows the daily number of deaths in the days before (orange dots) and after (red dots) the lockdown order was announced on 23rd March 2020 and after the lift (purple dots) of the lockdown order announced in 10th May. The green dots represent the number of daily deaths during the period of 1st January 2019 to 5th July 2019.

A

B

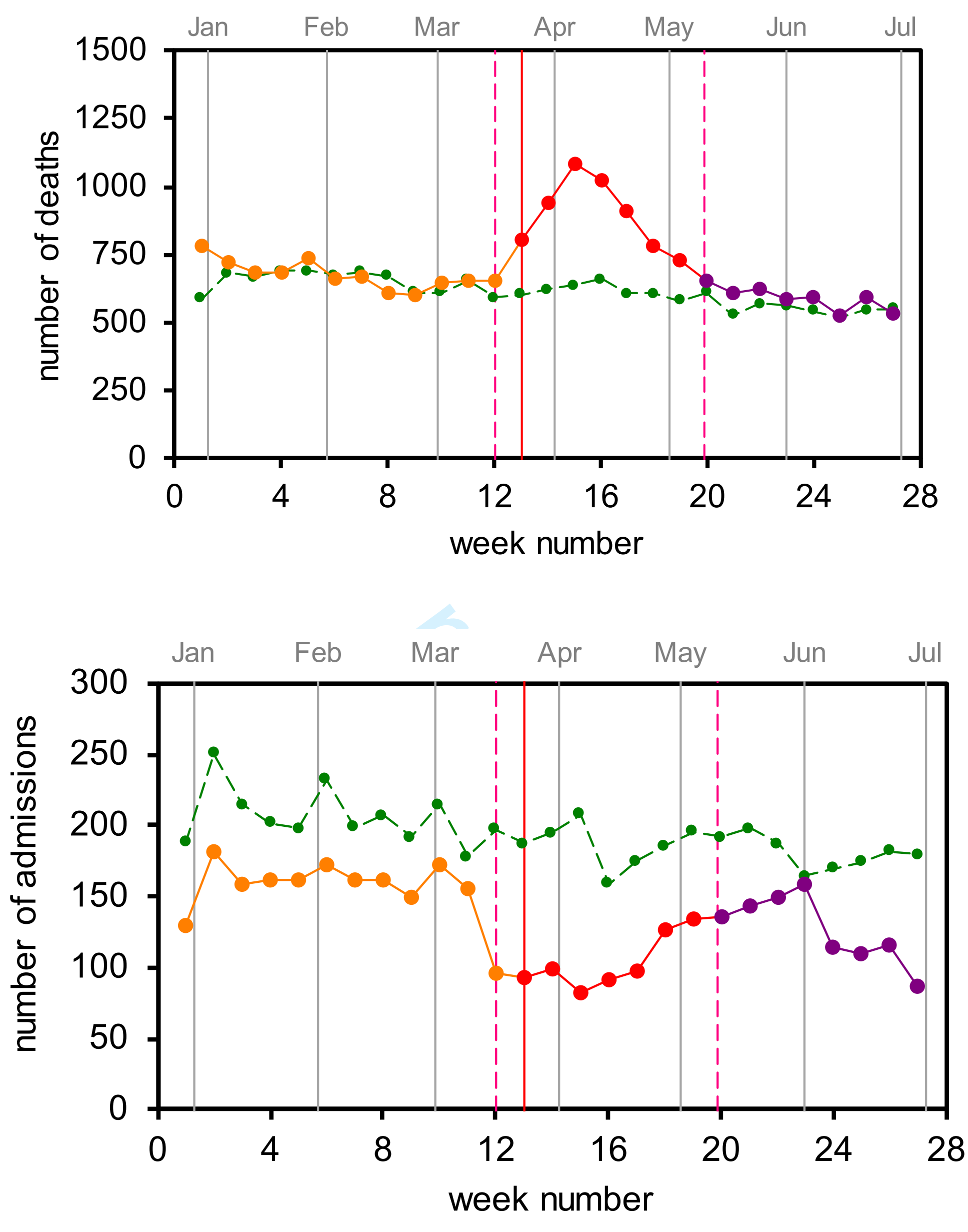

Supplementary Figure 3. Number of deaths per day before and after lockdown announcement for sites B, C, D, E, H and I during the period of 1st January 2020 to 31st May 2020. The vertical axis shows the daily number of deaths in the days before (orange dots) and after (red dots) the lockdown order was announced in 23rd March 2020 and after the lift (purple dots) of the lockdown order announced in 10th May. The green dots represent the number of daily deaths during the period of 1st January 2019 to 31st May 2019.

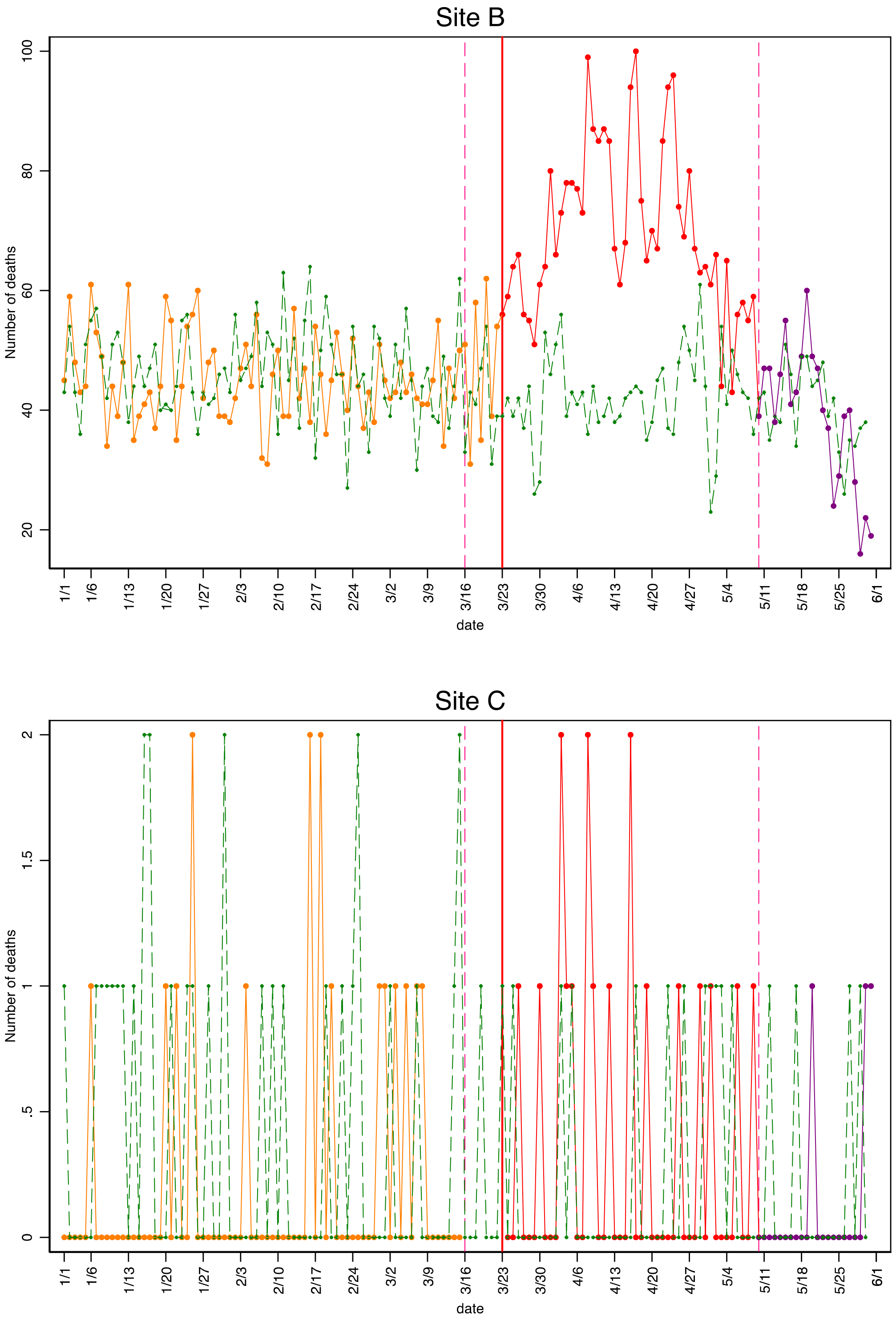

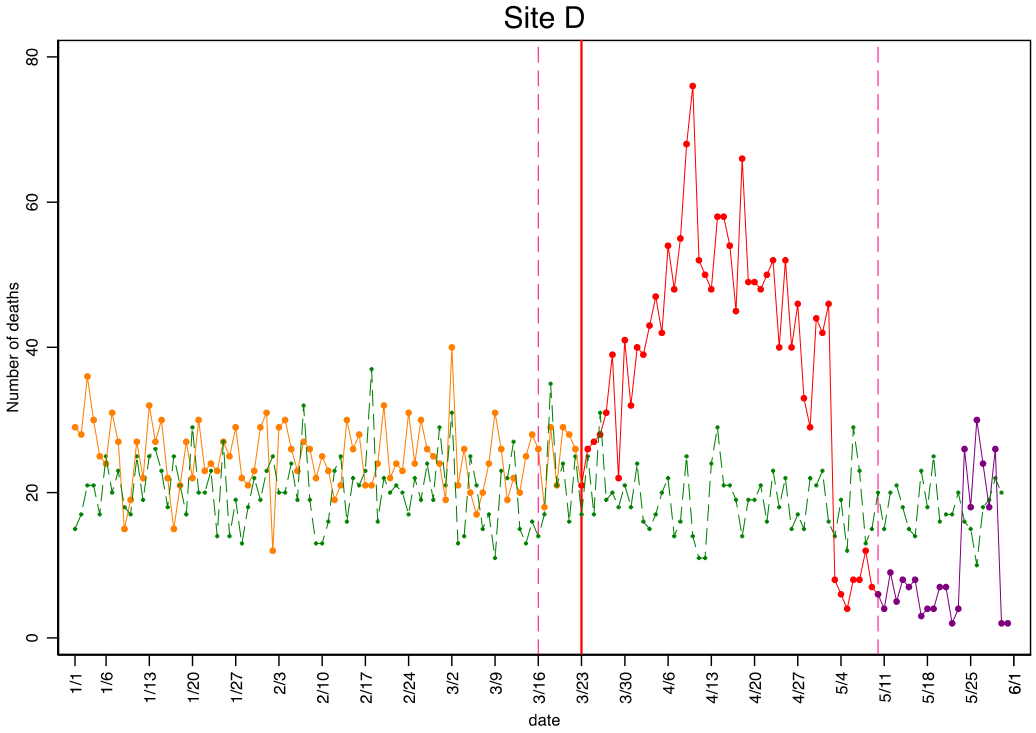
Site D
Number of deaths
0
20
40
60
80
1/1
1/6
1/13
1/20
1/27
2/3
2/10
2/17
2/24
3/2
3/9
3/16
3/23
3/30
4/6
4/13
4/20
4/27
5/4
5/11
5/18
5/25
6/1
date

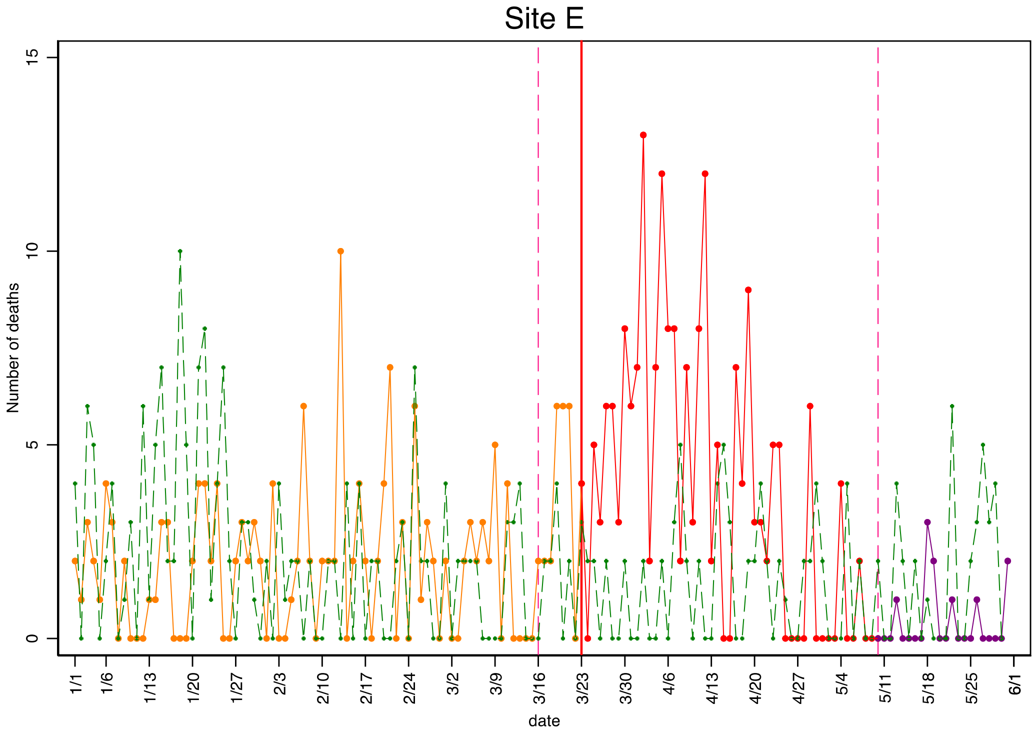
Site E
Number of deaths
0
5
10
15
1/1
1/6
1/13
1/20
1/27
2/3
2/10
2/17
2/24
3/2
3/9
3/16
3/23
3/30
4/6
4/13
4/20
4/27
5/4
5/11
5/18
5/25
6/1
date

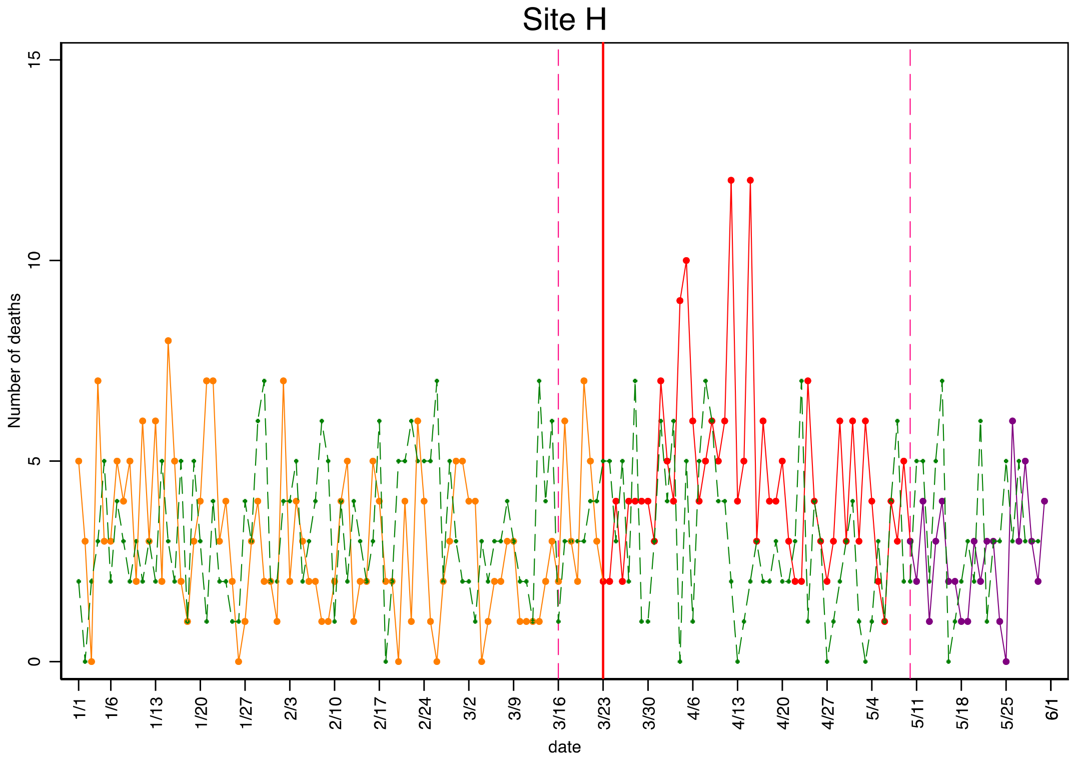

For peer review only

Supplementary Figure 4. Number of inpatient admissions per day before and after lockdown announcement for sites A, F and J during the period of 1st January 2020 to 31st July 2020. The vertical axis shows the daily number of deaths in the days before (orange dots) and after (red dots) the lockdown order was announced on 23rd March and after the lift (purple dots) of the lockdown order announced in 10th May. The green dots represent the number of daily deaths during the period of 1st January 2019 to 31st July 2019.

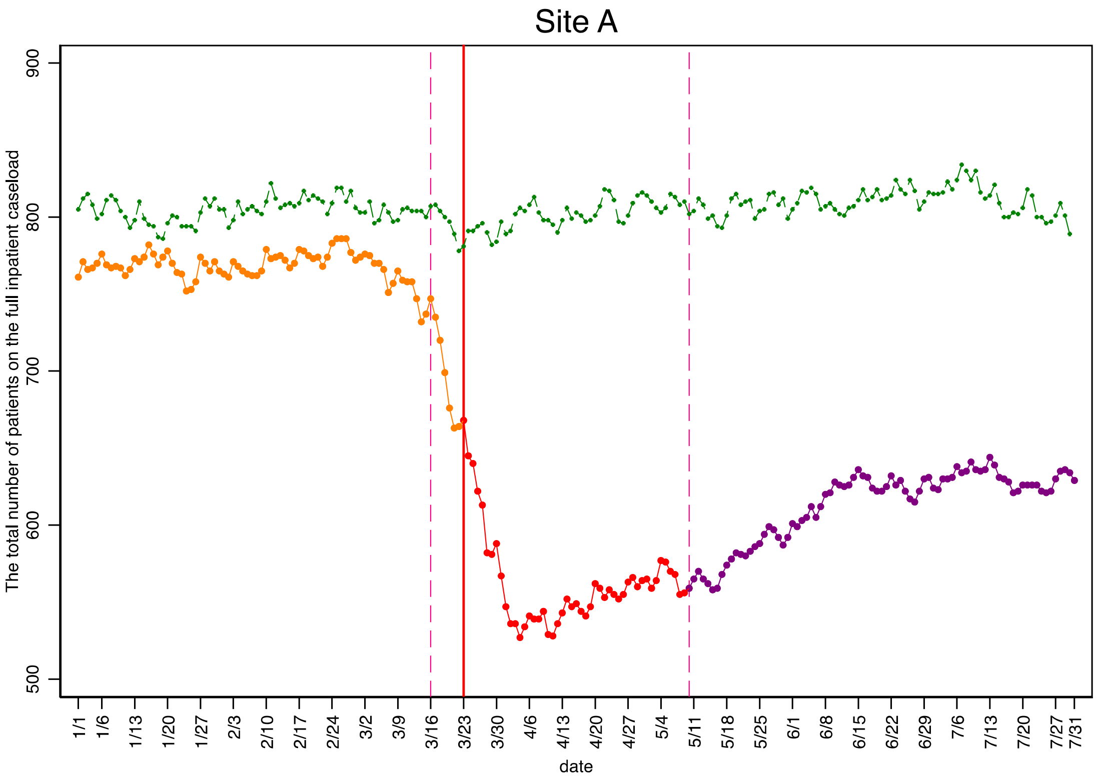

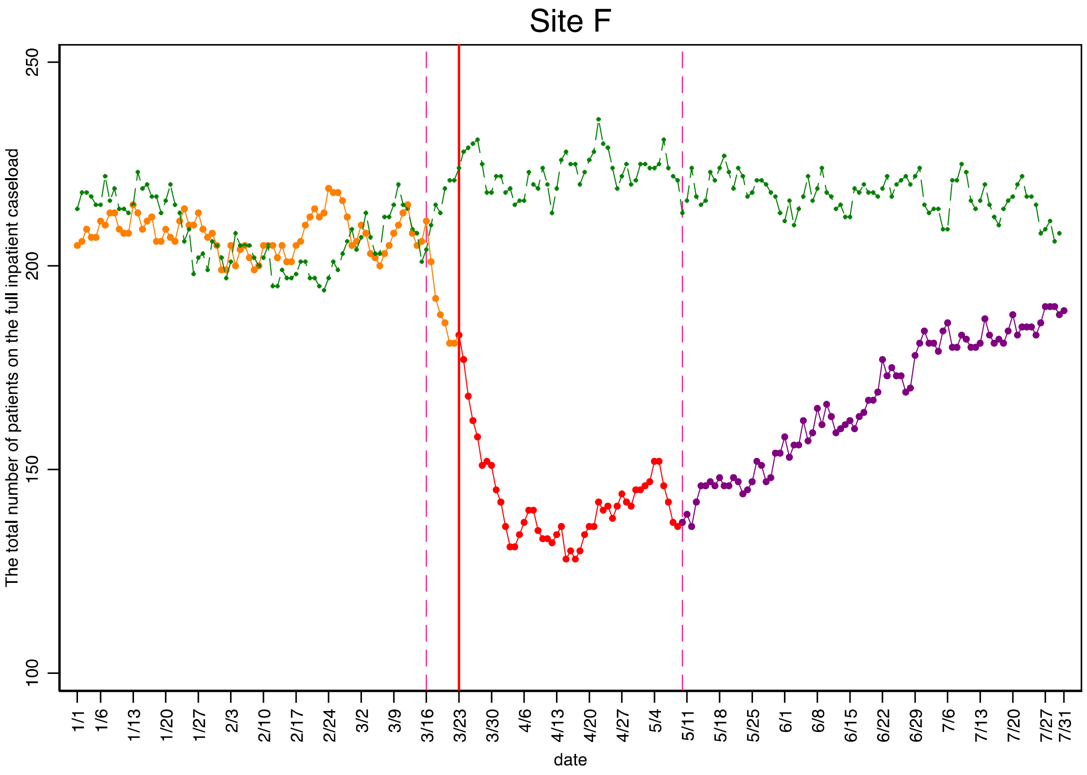

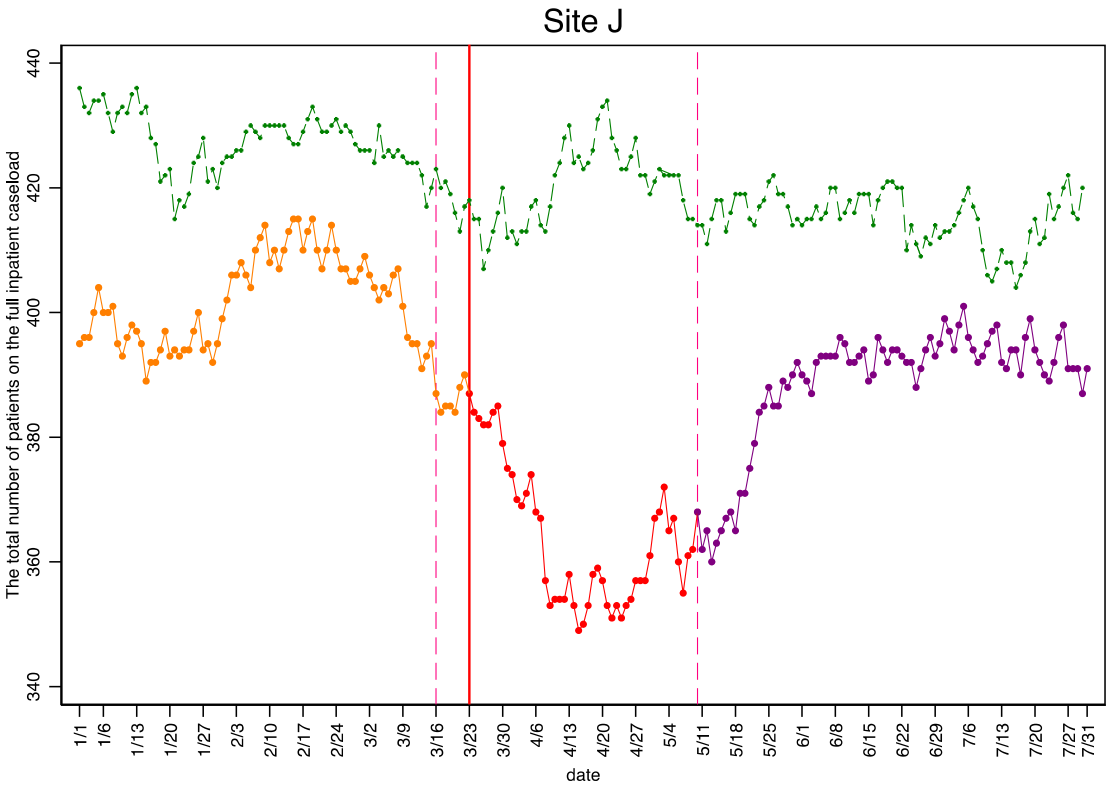
Site J
The total number of patients on the full inpatient caseload
440
420
400
380
360
340
1/1
1/6
1/13
1/20
1/27
2/3
2/10
2/17
2/24
3/2
3/9
3/16
3/23
3/30
4/6
4/13
4/20
4/27
5/4
5/11
5/18
5/25
6/1
6/8
6/15
6/22
6/29
7/6
7/13
7/20
7/27
7/31
date

review only

Supplementary Figure 5. Number of deaths per day before and after lockdown announcement for sites A, B, D, F, and J during the period of 1st January 2020 to 31st May 2020. The vertical axis shows the daily number of deaths in the days before (orange dots) and after (red dots) the lockdown order was announced on 23rd March and after the lift (purple dots) of the lockdown order announced on 10th May. The green dots represent the number of daily deaths during the period of 1st January 2019 to 31st May 2019. The lines represent the predicted values of our fitted model.

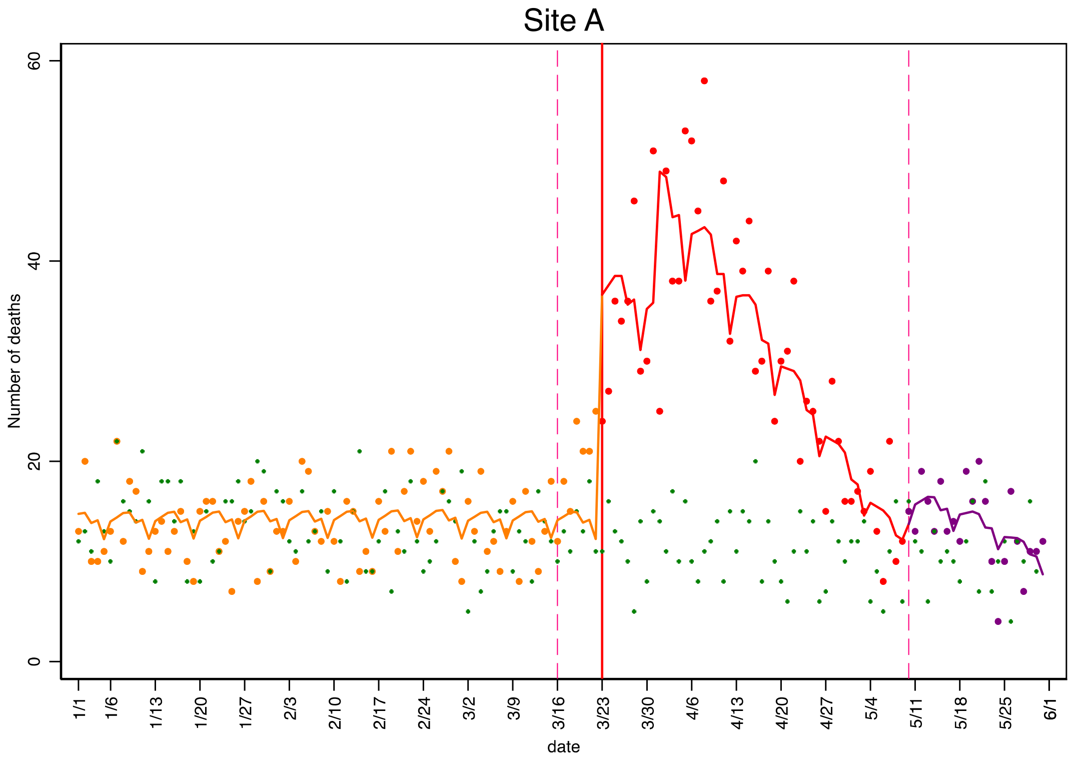

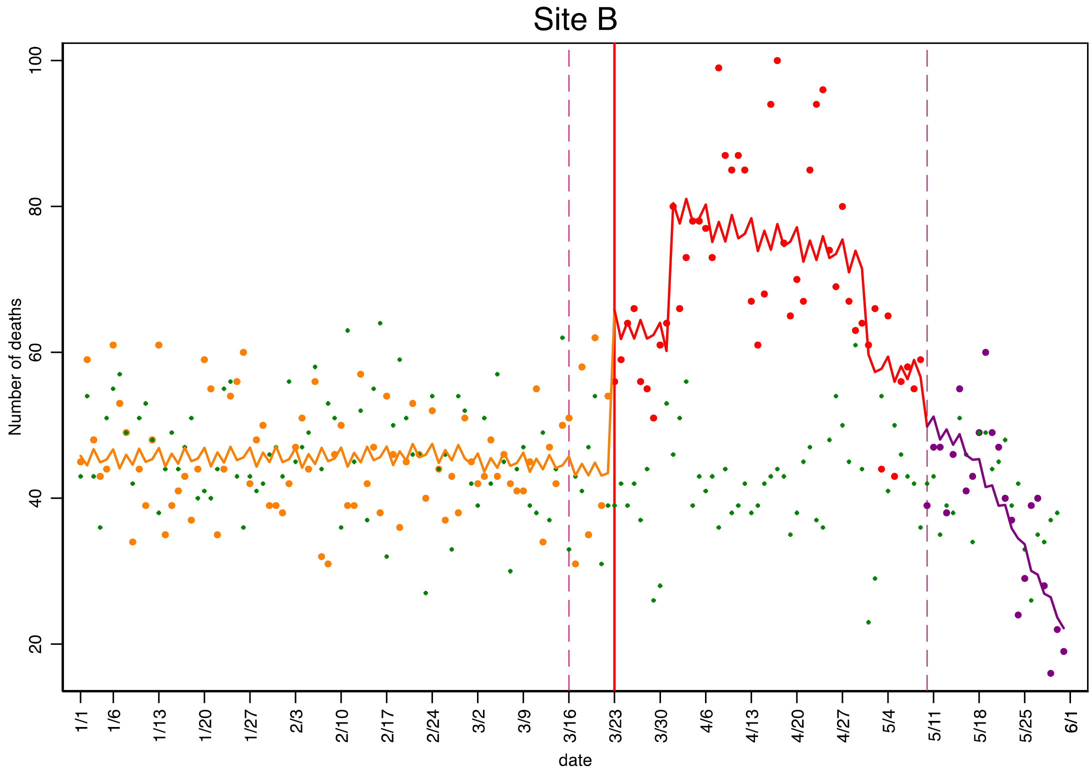

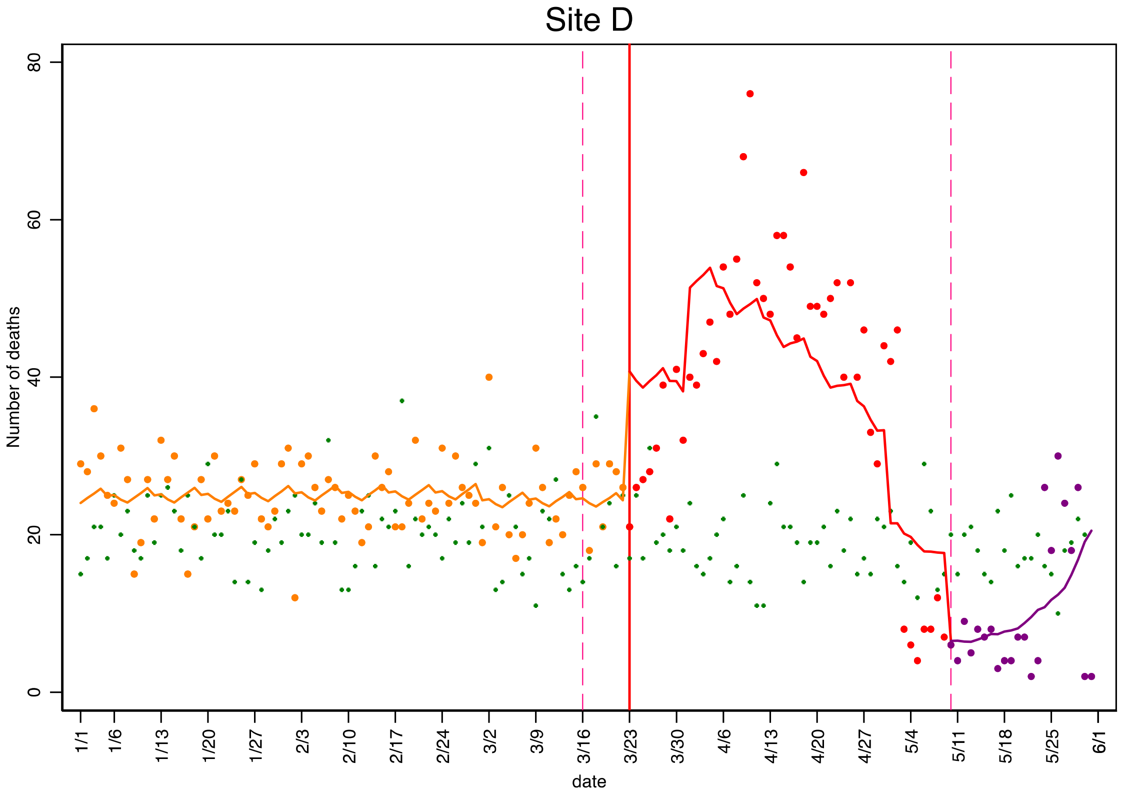
Site D
Number of deaths
date
0
20
40
60
80
1/1
1/6
1/13
1/20
1/27
2/3
2/10
2/17
2/24
3/2
3/9
3/16
3/23
3/30
4/6
4/13
4/20
4/27
5/4
5/11
5/18
5/25
6/1

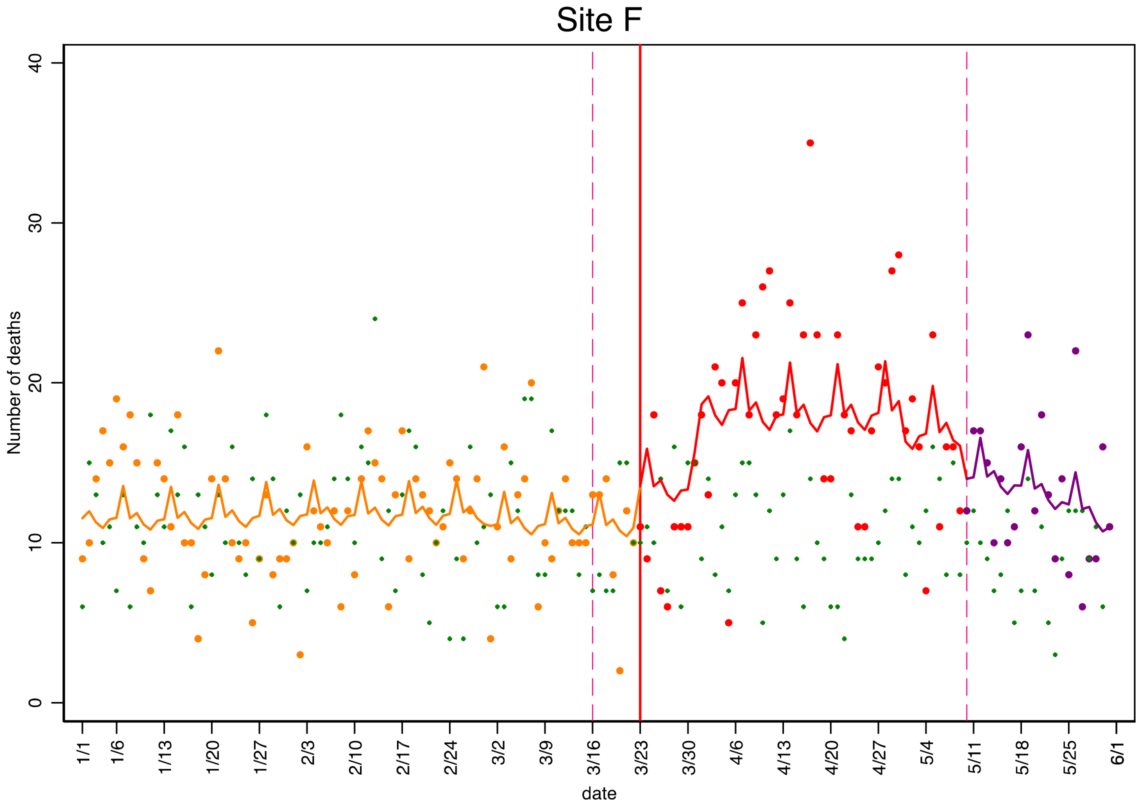
Site F
Number of deaths
date
0
10
20
30
40
1/1
1/6
1/13
1/20
1/27
2/3
2/10
2/17
2/24
3/2
3/9
3/16
3/23
3/30
4/6
4/13
4/20
4/27
5/4
5/11
5/18
5/25
6/1

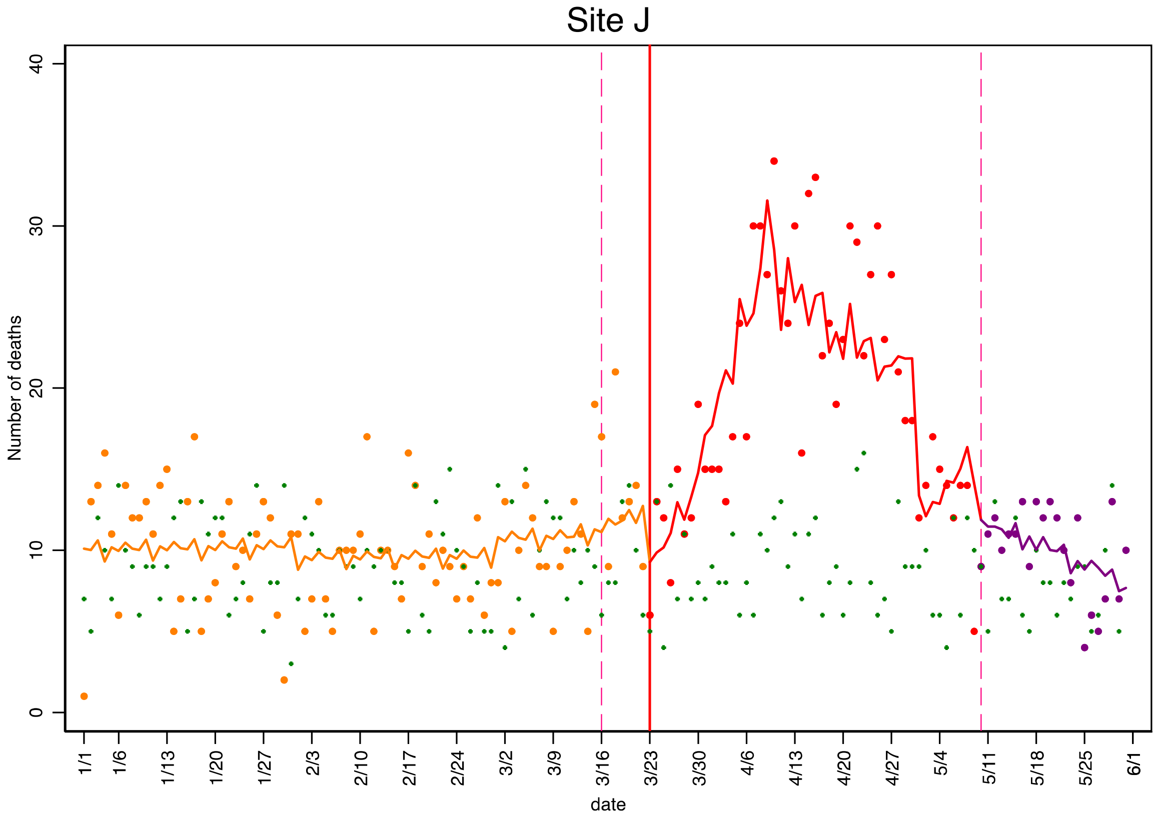

For peer review only

STROBE Statement—Checklist of items that should be included in reports of ***cross-sectional studies***

	Item No	Recommendation	Page No
**Title and abstract**	1	(*a*) Indicate the study's design with a commonly used term in the title or the abstract	1
		(*b*) Provide in the abstract an informative and balanced summary of what was done and what was found	5
**Introduction**			
Background/rationale	2	Explain the scientific background and rationale for the investigation being reported	7
Objectives	3	State specific objectives, including any prespecified hypotheses	7
**Methods**			
Study design	4	Present key elements of study design early in the paper	8-9
Setting	5	Describe the setting, locations, and relevant dates, including periods of recruitment, exposure, follow-up, and data collection	8-9, supplement
Participants	6	(*a*) Give the eligibility criteria, and the sources and methods of selection of participants	8-9
Variables	7	Clearly define all outcomes, exposures, predictors, potential confounders, and effect modifiers. Give diagnostic criteria, if applicable	9-10
Data sources/ measurement	8*	For each variable of interest, give sources of data and details of methods of assessment (measurement). Describe comparability of assessment methods if there is more than one group	8-10
Bias	9	Describe any efforts to address potential sources of bias	8-10
Study size	10	Explain how the study size was arrived at	N/A
Quantitative variables	11	Explain how quantitative variables were handled in the analyses. If applicable, describe which groupings were chosen and why	9-10
Statistical methods	12	(*a*) Describe all statistical methods, including those used to control for confounding	10-11, supplement
		(*b*) Describe any methods used to examine subgroups and interactions	10-11, supplement
		(*c*) Explain how missing data were addressed	N/A
		(*d*) If applicable, describe analytical methods taking account of sampling strategy	10-11, supplement
		(*e*) Describe any sensitivity analyses	10-11, supplement
**Results**			
Participants	13*	(a) Report numbers of individuals at each stage of study—eg numbers potentially eligible, examined for eligibility, confirmed eligible, included in the study, completing follow-up, and analysed	Table 1
		(b) Give reasons for non-participation at each stage	N/A
		(c) Consider use of a flow diagram	N/A
Descriptive data	14*	(a) Give characteristics of study participants (e.g. demographic, clinical, social) and information on exposures and potential confounders	Table 1
		(b) Indicate number of participants with missing data for each variable of interest	N/A

For peer review only - http://bmjopen.bmj.com/site/about/guidelines.xhtml

Outcome data	15*	Report numbers of outcome events or summary measures	Table 1
Main results	16	(*a*) Give unadjusted estimates and, if applicable, confounder-adjusted estimates and their precision (eg, 95% confidence interval). Make clear which confounders were adjusted for and why they were included	12-13, Table 2, supplement
		(*b*) Report category boundaries when continuous variables were categorized	N/A
		(*c*) If relevant, consider translating estimates of relative risk into absolute risk for a meaningful time period	N/A
Other analyses	17	Report other analyses done—eg analyses of subgroups and interactions, and sensitivity analyses	12-14, Table 3, supplement
**Discussion**			
Key results	18	Summarise key results with reference to study objectives	15
Limitations	19	Discuss limitations of the study, taking into account sources of potential bias or imprecision. Discuss both direction and magnitude of any potential bias	17
Interpretation	20	Give a cautious overall interpretation of results considering objectives, limitations, multiplicity of analyses, results from similar studies, and other relevant evidence	15-18
Generalisability	21	Discuss the generalisability (external validity) of the study results	17
**Other information**			
Funding	22	Give the source of funding and the role of the funders for the present study and, if applicable, for the original study on which the present article is based	2-3

*Give information separately for exposed and unexposed groups.

Note: An Explanation and Elaboration article discusses each checklist item and gives methodological background and published examples of transparent reporting. The STROBE checklist is best used in conjunction with this article (freely available on the Web sites of PLoS Medicine at http://www.plosmedicine.org/, Annals of Internal Medicine at http://www.annals.org/, and Epidemiology at http://www.epidem.com/). Information on the STROBE Initiative is available at www.strobe-statement.org.

For peer review only

BMJ Open

Changes in daily mental health service use and mortality at the commencement and lifting of COVID-19 'lockdown' policy in ten UK sites: A regression discontinuity in time design

Journal:	*BMJ Open*
Manuscript ID	bmjopen-2021-049721.R1
Article Type:	Original research
Date Submitted by the Author:	11-May-2021

Complete List of Authors:	Bakolis, Ioannis; King's College London Institute of Psychiatry Psychology and Neuroscience<br>Stewart, Robert; King's College London Institute of Psychiatry Psychology and Neuroscience; South London and Maudsley NHS Foundation Trust<br>Baldwin, David ; Southern Health NHS Foundation Trust; University of Southampton Faculty of Medicine<br>Beenstock, Jane; Lancashire and South Cumbria NHS Foundation Trust<br>Bibby, Paul; Lancashire and South Cumbria NHS Foundation Trust<br>Broadbent, Matthew; South London and Maudsley NHS Foundation Trust<br>Cardinal, Rudolf; University of Cambridge School of Clinical Medicine, Department of Psychiatry; Cambridgeshire and Peterborough NHS Foundation Trust, Liaison Psychiatry Service<br>Chen, Shanquan; University of Cambridge School of Clinical Medicine, Department of Psychiatry<br>Chinnasamy, Karthik; Camden and Islington NHS Foundation Trust<br>Cipriani, Andrea; University of Oxford Medical Sciences Division, Department of Psychiatry; Oxford Health NHS Foundation Trust<br>Douglas, Simon; Cumbria Northumberland Tyne and Wear NHS Foundation Trust<br>Horner, Philip; Lancashire and South Cumbria NHS Foundation Trust<br>Jackson, Caroline A.; University of Edinburgh Division of Medical and Radiological Sciences, Usher Institute of Population Health Sciences & Informatics<br>John, Ann; Swansea University Medical School, Population Data Science<br>Joyce, Dan W.; University of Oxford, Department of Psychiatry<br>Lee, Sze; Swansea University Medical School, Population Data Science<br>Lewis, Jonathan; Cambridgeshire and Peterborough NHS Foundation Trust<br>McIntosh, Andrew; University of Edinburgh Division of Medical and Radiological Sciences, Division of Psychiatry<br>Nixon, Neil; University of Nottingham Faculty of Medicine and Health Sciences, Division of Psychiatry and Applied Psychology; Nottinghamshire Healthcare NHS Foundation Trust<br>Osborn, David; University College London Faculty of Medical Sciences, Division of Psychiatry<br>Phiri, Peter; Southern Health NHS Foundation Trust<br>Rathod, Shanaya; Southern Health NHS Foundation Trust

	Smith, Tanya; Oxford Health NHS Foundation Trust<br>Sokal, Rachel; Nottinghamshire Healthcare NHS Foundation Trust<br>Waller, Rob; Lothian Primary Care NHS Trust<br>Landau, Sabine; King's College London Institute of Psychiatry Psychology and Neuroscience

**Primary Subject Heading**:	Mental health
Secondary Subject Heading:	Health services research, Epidemiology
Keywords:	COVID-19, MENTAL HEALTH, Organisation of health services < HEALTH SERVICES ADMINISTRATION & MANAGEMENT, Adult psychiatry < PSYCHIATRY, Old age psychiatry < PSYCHIATRY

SCHOLARONE™ Manuscripts

I, the Submitting Author has the right to grant and does grant on behalf of all authors of the Work (as defined in the below author licence), an exclusive licence and/or a non-exclusive licence for contributions from authors who are: i) UK Crown employees; ii) where BMJ has agreed a CC-BY licence shall apply, and/or iii) in accordance with the terms applicable for US Federal Government officers or employees acting as part of their official duties; on a worldwide, perpetual, irrevocable, royalty-free basis to BMJ Publishing Group Ltd ("BMJ") its licensees and where the relevant Journal is co-owned by BMJ to the co-owners of the Journal, to publish the Work in this journal and any other BMJ products and to exploit all rights, as set out in our licence.

The Submitting Author accepts and understands that any supply made under these terms is made by BMJ to the Submitting Author unless you are acting as an employee on behalf of your employer or a postgraduate student of an affiliated institution which is paying any applicable article publishing charge ("APC") for Open Access articles. Where the Submitting Author wishes to make the Work available on an Open Access basis (and intends to pay the relevant APC), the terms of reuse of such Open Access shall be governed by a Creative Commons licence – details of these licences and which Creative Commons licence will apply to this Work are set out in our licence referred to above.

Other than as permitted in any relevant BMJ Author's Self Archiving Policies, I confirm this Work has not been accepted for publication elsewhere, is not being considered for publication elsewhere and does not duplicate material already published. I confirm all authors consent to publication of this Work and authorise the granting of this licence.

For peer review only

Changes in daily mental health service use and mortality at the commencement and lifting of COVID-19 'lockdown' policy in ten UK sites: A regression discontinuity in time design

Ioannis Bakolis[1]*, Robert Stewart[2,3]*, David S. Baldwin[4], Jane Beenstock[5], Paul Bibby[5], Matthew Broadbent[3], Rudolf Cardinal[6,7], Shanquan Chen[6], Karthik Chinnasamy[8], Andrea Cipriani[9,10], Simon Douglas[11], Philip Horner[5], Caroline Jackson[12], Ann John[13], Dan W. Joyce[9], Sze Chim Lee[13], Jonathan Lewis[7], Andrew McIntosh[14], Neil Nixon[15,16], David P.J. Osborn[17], Peter Phiri[18], Shanaya Rathod[18], Tanya Smith[10], Rachel Sokal[16], Rob Waller[19], Sabine Landau[20].

*Equal contributions to authorship

[1]Departments of Biostatistics and Health Informatics & Health Services and Population Research, Institute of Psychiatry, Psychology and Neuroscience, King's College London, London, UK.

[2]Department of Psychological Medicine, Institute of Psychiatry, Psychology and Neuroscience, King's College London, London, UK.

[3]South London and Maudsley NHS Foundation Trust, London, UK.

[4]Clinical and Experimental Sciences (CNS and Psychiatry), Faculty of Medicine, University of Southampton, Southampton, UK.

[5]Lancashire & South Cumbria NHS Foundation Trust, Preston, UK.

[6]Department of Psychiatry, University of Cambridge, Cambridge, UK.

[7]Cambridgeshire & Peterborough NHS Foundation Trust, Cambridge, UK.

[8]Camden and Islington NHS Foundation Trust, London, UK.

[9]Department of Psychiatry, University of Oxford, Oxford, UK.

[10]Oxford Health NHS Foundation Trust, Oxford, UK.

[11]Cumbria, Northumberland, Tyne & Wear NHS Foundation Trust, Newcastle upon Tyne, UK.

[12]Usher Institute of Population Health Sciences & Informatics, University of Edinburgh, Edinburgh, UK.

[13]Population Data Science, Swansea University Medical School, Swansea, UK.

[14]University of Edinburgh, Centre for Clinical Brain Sciences, Edinburgh, UK.

For peer review only - http://bmjopen.bmj.com/site/about/guidelines.xhtml

[15]Division of Psychiatry and Applied Psychology, School of Medicine, University of Nottingham, Nottingham, UK.

[16]Nottinghamshire Healthcare NHS Foundation Trust, Nottingham, UK.

[17]Division of Psychiatry, University College London, London, UK.

[18]Southern Health NHS Foundation Trust, Southampton, UK.

[19]Lothian Primary Care NHS Trust, Edinburgh, UK.

[20]Department of Biostatistics and Health Informatics, Institute of Psychiatry, Psychology and Neuroscience, King's College London, London, UK.

Corresponding author: Prof. Robert Stewart, Department of Psychological Medicine, Institute of Psychiatry, Psychology and Neuroscience, King's College London, De Crespigny Park, London SE5 8AF, UK. Email: r.stewart@iop.kcl.ac.uk

Funding statement

No specific funding was received for this study. Regarding relevant background infrastructure funding, RS is part-funded by: i) the NIHR Biomedical Research Centre at the South London and Maudsley NHS Foundation Trust and King's College London; ii) a Medical Research Council (MRC) Mental Health Data Pathfinder Award to King's College London; iii) an NIHR Senior Investigator Award; iv) the NIHR Applied Research Collaboration South London (NIHR ARC South London) at King's College Hospital NHS Foundation Trust. IB and SL are supported by the NIHR Biomedical Research Centre at South London and Maudsley NHS Foundation Trust and King's College London, and by the NIHR Applied Research Collaboration South London (NIHR ARC South London) at King's College Hospital NHS Foundation Trust. RNC's research is funded by the MRC (grant MC_PC_17213), and the NIHR Cambridge Biomedical Research Centre. AC is supported by the NIHR Oxford Cognitive Health Clinical Research Facility, by an NIHR Research Professorship (grant RP-2017-08-ST2-006), by the NIHR Oxford and Thames Valley Applied Research Collaboration, and by the NIHR Oxford Health Biomedical Research Centre (grant BRC-1215-20005). AJ is part funded by MQ ADP, and a Medical Research Council (MRC) Mental Health Data Pathfinder Award to Swansea University. AJ and SCL are part funded by Health and Care Research Wales National Centre for Mental Health. DO is supported by the NIHR Biomedical Research Centre (BRC) at University College London Hospitals (UCLH), and by the National Institute for Health Research ARC North Thames. Additional infrastructure funding was provided by the Medical Research Council (MRC)

For peer review only

Mental Health Data Pathfinder Award to University of Edinburgh (MC_PC_17209). The collaboration providing Wales data was led by the Swansea University Health Data Research UK team under the direction of the Welsh Government Technical Advisory Cell (TAC) and includes the following groups and organisations: the Secure Anonymised Information Linkage (SAIL) Databank, Administrative Data Research (ADR) Wales, NHS Wales Informatics Service (NWIS), Public Health Wales, NHS Shared Services and the Welsh Ambulance Service Trust (WAST), MRC grant MR/V028367. This study was additionally supported by the NIHR Mental Health Translational Research Collaboration (MH-TRC). The views expressed are those of the authors and not necessarily those of the UK National Health Service, the NIHR, or the UK Department of Health.

Declaration of interests of interests statement

RNC consults for Campden Instruments Ltd. and receives royalties from Cambridge University Press, Cambridge Enterprise, and Routledge. DSB reports royalties from Wiley publishers. AC reports research funding from Angelini Pharma and personal fees from INCiPiT, CARIPLO Foundation and Angelini Pharma. AM reports research funding from The Sackler Trust and personal fees from Janssen and Illumina. PP reports research funding from NovoNordisk, and royalties from John Wiley & Sons. SR reports education support from Janssen, Otsuka and Lundbeck. RS reports research support from Janssen, GSK and Takeda, and royalties from Oxford University Press.

Acknowledgements

This work uses data provided by patients and collected by the NHS as part of their care and support. We would also like to acknowledge all data providers who make anonymised data available for research. We wish to acknowledge the collaborative partnership that enabled acquisition and access to the de-identified data, which led to this output, and which was facilitated by MQ and the National Institute for Health Research (NIHR) Mental Health Translational Research Collaborative (MH-TRC).

Author contributions

The study was initially conceived by RSt, IB and SL, and further planned and designed by RSt, IB, DSB, JB, PB, MB, RC, SC, KC, AC, SD, PH, CJ, AJ, DWJ, SCL, JL, AM, NN, DPJO, PP, SR, TS, RSo, RW and SL. The study was carried out by RSt, IB, DSB, JB, PB, MB, RC, SC, KC, AC, SD, PH, CJ, AJ, DWJ, SCL, JL, AM, NN, DPJO, PP, SR, TS, RSo

For peer review only - http://bmjopen.bmj.com/site/about/guidelines.xhtml

and RW. Data analyses were carried out by IB, SL, AJ and SCL. The manuscript was initially drafted by IB and RSt with input on successive drafts from DSB, JB, PB, MB, RC, SC, KC, AC, SD, PH, CJ, AJ, DWJ, SCL, JL, AM, NN, DPJO, PP, SR, TS, RSo, RW and SL. The final version of the manuscript has been seen and approved by all authors.

Patient and Public Involvement statement

The research data resources and infrastructures supporting this study receive extensive patient and public input. The specific analyses reported here did not include any specific involvement in the design, conduct or reporting.

Data availability statement

All data relevant to the study are available from the corresponding author on request.

Manuscript word count: 4517

For peer review only

ABSTRACT

Objectives: To investigate changes in daily mental health service use and mortality in response to the introduction and the lifting of the COVID-19 'lockdown' policy in Spring 2020.

Design: A regression discontinuity in time analysis of daily service-level activity.

Setting and participants: Mental healthcare data were extracted from ten UK providers.

Outcome measures: Daily (weekly for one site) deaths from all causes, referrals and discharges, inpatient care (admissions, discharges, caseloads), and community services (face-to-face/non-face-to-face contacts, caseloads): Adult, Older Adult, and Child/Adolescent Mental Health; Early Intervention in Psychosis; Home Treatment Teams; Liaison/A&E. Data were extracted for 2019 and 2020 to 31st May for all sites, supplemented to 31st July for four sites. Changes around the commencement and lifting of COVID-19 'lockdown' policy (March 23rd and May 10th respectively) were estimated using a regression discontinuity in time design with a difference-in-difference approach generating incidence rate ratios (IRRs), meta-analysed across sites.

Results: Pooled estimates for the lockdown transition showed increased daily deaths (IRR 2.31, 95% CI 1.86-2.87), reduced referrals (0.62, 0.55-0.70), and reduced inpatient admissions (0.75, 0.67-0.83) and caseloads (0.85, 0.79-0.91) compared to the pre-lockdown period. All community services saw shifts from face-to-face to non-face-to-face contacts, but varied in caseload changes. Lift-of-lockdown was associated with reduced deaths (0.42, 0.27-0.66), increased referrals (1.36, 1.15-1.60), and increased inpatient admissions (1.21, 1.04-1.42) and caseloads (1.06, 1.00-1.12) compared to the lockdown period. Site-wide activity, inpatient care and community services did not return to pre-lockdown levels after lift-of-lockdown, while number of deaths did. Between-site heterogeneity most often indicated variation in size rather than direction of effect.

Conclusions: Mental health service delivery underwent sizeable changes during the first national lockdown, with as-yet unknown and unevaluated consequences.

Keywords: COVID-19; mental healthcare; electronic health records; inpatient mental health; home treatment team; community mental health team; child and adolescent mental health; older adult mental health; liaison psychiatry.

For peer review only

For peer review only

Strengths and limitations of this study

- The data were obtained from a large number of mental health providers covering an extensive and varied geography within the UK: we believe, the most extensive multi-site evaluation to date
- Despite this, data are catchment-specific and would require further replication to clarify national and international generalisability
- Data are combined across a number of potentially diverse services for each site, with no attempt to investigate within-site heterogeneity.
- The changes described here evaluated the first wave of the COVID-19 'lockdown' policy in the UK and cannot necessarily be extrapolated to further lockdown periods.

For peer review only

For peer review only - http://bmjopen.bmj.com/site/about/guidelines.xhtml

INTRODUCTION

The ‘first wave’ of the COVID-19 pandemic affected healthcare sectors, not only through the virus’ direct effects on communities and healthcare staff, but also from the national public health policies enacted to reduce spread (1). Mental health (MH) care faced a range of challenges in many countries, including the heightened vulnerability of its patient populations (e.g. through cardiovascular and respiratory disorders), already-reduced life expectancy (2), and problems in accessing healthcare (3, 4). Services had to be radically reconfigured to manage suspected or confirmed COVID-19 infections in inpatient and outpatient settings, staff sickness or self-isolation, the need to minimise face-to-face contacts, and the imperative to accommodate increasing pressures on acute medical care from cases of viral infection (5, 6). In turn, these changes were accompanied by the (as yet unknown) impacts of ‘social distancing’ on already isolated or otherwise vulnerable populations (although with potential positive effects of increased support, sense of community cohesion, and reduced social strain for some individuals), and of economic constraints on already impoverished and disadvantaged communities (7, 8). This indicates a pressing need for research assessing the impact of the pandemic and lockdown policies on mental health service demand and uptake (9).

Published data are emerging on MH outcomes in community samples (10) and specific groups such as healthcare workers (11), as well as on infection rates in MH inpatients (12), and staff/expert concerns about MH provision (6, 13). However, there have been few studies to date quantifying changes in mental healthcare activity (5). Single-site reports from UK services have highlighted falls in activity following the March 23rd national lockdown, followed by increased demand for some services, decreased activity for others, shifts from face-to-face to virtual consultations, and a rise in mortality (14-19). Drawing on a network of providers, we sought to determine the level and heterogeneity of such mental healthcare changes across multiple national sites using a quasi-experimental, regression discontinuity design in time.

For peer review only - http://bmjopen.bmj.com/site/about/guidelines.xhtml

METHODS

Data sources

Early in the first UK lockdown, we enquired of several MH care providers as to the feasibility of a relatively short-notice extraction of service-level data according to a standardised protocol. Sites were sourced initially from the NIHR Mental Health Translational Research Collaboration, a network of MH services with NIHR or equivalent funding. MH Trusts in England provide all NHS specialist MH care to defined geographic catchment areas. Information from these was supplemented by further extractions of available mental healthcare data from Scotland and Wales service providers.

The following English Trusts were able to participate: i) Cambridgeshire and Peterborough Foundation Trust, serving the Combined Authority of Cambridgeshire and Peterborough, around 860,000 people; ii) Camden & Islington Foundation Trust, serving the Camden and Islington boroughs of north London, around 500,000 people; iii) Cumbria, Northumberland, Tyne & Wear Foundation Trust, based in Newcastle and covering more than 70 sites across North Cumbria, Northumberland and Tyne and Wear, a population of around 2 million; iv) Lancashire & South Cumbria Foundation Trust, serving Lancashire and South Cumbria, population 1.8 million; v) Oxford Health Foundation Trust, serving Oxfordshire, Buckinghamshire, Wiltshire, Milton Keynes, Swindon, Bath and North East Somerset, a population of around 3 million; vi) Nottinghamshire Healthcare Trust, serving City and County of Nottinghamshire, around 1.2 million people; vii) South London and Maudsley Foundation Trust, serving Croydon, Lambeth, Lewisham, and Southwark boroughs of south London, a population of around 1.3 million; viii) Southern Health Foundation Trust, serving Hampshire (excluding Portsmouth City), a population of around 1.3 million. Additional data were made available from NHS Lothian, a provider of all health services to a population of 850,000 in and around Edinburgh, including all physical and MH services; and from the Secure Anonymised Information Linkage (SAIL) databank which contains anonymised data on all health services provided to the population of Wales, around 3 million.

All data were extracted at service level, with no individual patient-level extractions. Sites were de-identified prior to analysis and are referred to subsequently as Sites A-J.

For peer review only - http://bmjopen.bmj.com/site/about/guidelines.xhtml

Nine of the ten sites extracted daily activity data from 1st January 2020 to 31st May 2020 and 2019; extractions took place in June-July 2020. Four of the ten sites were subsequently able to provide extensions of daily data up to 31st July 2020, extracted in August-September 2020. One site extracted (in September 2020) all activity data on a weekly level from 1st January 2020 to 5th July 2020 in addition to 2019.

Mental health service activity and mortality variables

Where possible, sites extracted the following data for each day:

1. Site activity: number of new referrals accepted; number of discharges from services.
2. MH inpatient services: number of new admissions; number of discharges; daily inpatient caseload (total and caseload detained under the Mental Health Act [MHA]) section for all age groups).
3. Community services. For each service, the number of face-to-face (f2f) contacts, non-face-to-face (non-f2f) contacts, total contacts (sum of f2f and non-f2f), cancelled appointments or non-attendance (DNA), and daily caseload (patients with an active referral to that service). These were extracted for each of the following community service types:
    a. Adult Mental Health (AMH; community MH teams)
    b. Child and Adolescent Mental Health Services (CAMHS)
    c. Early Intervention in Psychosis (EIP)
    d. Home Treatment Team (HTT) – also known as crisis resolution teams which provide the option for at least daily reviews as an alternative to inpatient care.
    e. Liaison psychiatry services, including services provided to emergency departments and settings created as alternatives to emergency departments during the pandemic.
    f. Older Adults (OA) MH services.

Daily mortality was extracted as recorded within clinical systems from routine data supplied to sites from national sources. For sites A, B, E, F, H and J, deaths recorded on the clinical system represent those for all past service contacts regardless of whether the individual is a current patient at the time of death (for 90% of the catchment served by site H). Sites D and G ascertain deaths for all current patients at the time of death along with selected discharged patients (up to 6 months post-discharge for Site G). For site C, deaths were restricted to those on MH inpatient wards at the time of death. For Site I, weekly deaths and weekly inpatient

For peer review only - http://bmjopen.bmj.com/site/about/guidelines.xhtml

admissions/discharges were extracted where mental health was recorded as the primary diagnosis.

Statistical analysis

To describe changes in the above measures we used two time points coinciding with the commencement and lifting of the COVID-19 'lockdown' policy: 23rd March 2020 and 10th May 2020 respectively. To estimate these, we deployed a regression discontinuity in time (RDiT) design (20) combined with a difference-in difference (DiD) approach to estimation, comparing measures before and after the lockdown announcement in 2020 to those before and after the same date in 2019, thus adjusting for potential seasonal changes. As similar analysis was conducted for the lift-of-lockdown announcement.

For analytical purposes, we divided the sample into two cohort periods (2019, 2020). The parameters of interest were the effects of (a) lockdown and (b) lift of the lockdown policy announcements upon extracted measures in different time windows before and after 23rd March and 10th May 2020, relative to that observed before and after 23rd March and 10th May 2019. This approach was strengthened by borrowing elements from a DiD approach and is similar to a difference in discontinuities design because it rests on the intuition of combining an RDiT with a DiD strategy (RDiT-DiD) (21). Further rationale and details of the analytic approach are provided in the Technical Appendix.

Differences in measures before versus after the cut-off dates were reported as incidence rate ratios (IRRs) and their corresponding 95% confidence intervals (CI). Negative binomial regression models were used to assess the effect of March 23rd COVID-19 'lockdown' policy on mortality and mental health service activity adjusted for temporal trends (e.g weekday, month, year) and taking into account overdispersion (please technical appendix and Supplementary Figures 1-5). Results from each site extracting daily data were pooled across sites using random effects meta-analysis and heterogeneity was summarised using the I^2 statistic (22).

A series of sensitivity analyses were also performed.

1. We re-ran a subgroup analysis of our main RDiT-DiD analysis for four sites with daily data that could update their data extractions to 31 July 2020. We increased the

For peer review only

time window after the lift-of the lockdown announcement in order to capture any potential effects of the lift-of the lockdown announcement on mortality and mental health service activity with greater statistical certainty due to larger numbers.

2. To account for anticipatory effects of the lockdown and lift-of-lockdown announcements, we omitted one week either side of the cut-off date of 23rd March 2020 (i.e. 16th March 2020 to 30th March 2020) and one week either side of 10th May 2020 (i.e. 3rd May 2020 to 17th May 2020). Anticipatory effects, such as the announcement of social distancing on 16th March 2020, could have resulted in altered MH service activity in the study population just before the implementation of lockdown. Similarly, non-adherence to the lockdown rules was documented during the month of April 2020 and before lockdown was lifted (23).
3. To account for changes in the infection rate, we further adjusted our models for national COVID-19 related deaths occurring one week prior to the date of our observed daily counts.
4. To check robustness of our findings, that these were not an artefact of the pre-lockdown time period from 1st January 2020, we reduced the time window before the lockdown announcement from 4th February 2020. Thus, we used the same number of daily observations before and after the lockdown announcement (23rd March to 10th May 2020 vs. 4th February to 23rd March 2020) and the lift-of-lockdown announcement (10th to 31st May 2020 vs. 19th February to 23rd March 2020).
5. To account for changes due to the media coverage of the pandemic we further adjusted our models for trends in the UK occurring on the day of our observed daily counts. Google Trends data provides an unfiltered sample of search requests made to Google. It supplies an index for search intensity by topic over the time period requested in the UK area. This is the number of daily searches for the specified topic divided by the maximum number of daily searches for this topic over the time period in question in the UK. This is scaled from zero to 100. A value of 100 is the peak popularity for the term. A value of 50 means that the term is half as popular. A score of 0 means there was not enough data for this term (24).

Analyses were performed using STATA 15.1 (Stata Corporation, College Station, TX USA).

For peer review only

For peer review only - http://bmjopen.bmj.com/site/about/guidelines.xhtml

333

For peer review only

For peer review only - http://bmjopen.bmj.com/site/about/guidelines.xhtml

RESULTS

The ten participating sites are described and compared in Table 1 and pooled IRRs from the random-effect meta-analysis are presented in Table 2 for the three period comparisons of interest in the sites extracting daily data.

Transition to lockdown

For this transition, pooled estimates showed a significant and greater than two-fold increase in daily deaths (IRR 2.31 (95% CI: 1.86-2.87); Figure 1). New accepted referrals decreased (IRR 0.62 (0.55-0.70)). Pooled estimates from inpatient care showed a decrease in new admissions (IRR 0.75 (0.67-0.83)), total daily caseload (IRR 0.85 (0.79-0.91)) and numbers of patients detained under an MHA section (IRR 0.93 (0.87-0.99)). Of community services evaluated, all showed sizeable decreases in daily numbers of f2f contacts and increases in non-f2f contacts (both strongest for EIP). Total contacts reduced in Liaison and HTT services and increased (but not significantly so) in AMH, CAMHS, EIP, and OA services. Daily caseloads significantly reduced in CAMHS, HTT and Liaison, and significantly increased in AMH. No significant changes were observed in the numbers of cancelled or non-attended appointments, apart from a reduction within HTT.

Transition from lockdown

Numbers of deaths reduced considerably (IRR 0.42 (0.27-0.66)), and IRR estimates for pre- vs. post-lockdown differences were not significantly different (0.97 (0.68-1.38)). Numbers of new site referrals increased after the lifting of lockdown (IRR 1.36 (1.15-1.60)) compared to the lockdown period but remained lower than pre-lockdown (IRR 0.85 (0.76-0.95)). Inpatient services saw increased admissions (IRR 1.21 (1.04-1.42)) and reduced discharges (IRR 0.66 (0.53-0.84)) after the lifting of lockdown. Discharges remained lower than before lockdown, as did caseloads. F2f contacts increased after lockdown in all services apart from AMH and CAMHS, increases in non-f2f contacts were seen in AMH, EIP and OA services, and total contacts increased in EIP, HTT, Liaison and OA services. Compared to pre-lockdown, pooled daily f2f contacts reduced and non-f2f contacts increased in all services after lifting of lockdown apart from HTT in which f2f contacts increased and non-f2f contacts were unchanged; total contacts increased in AMH, CAMHS, and EIP services and decreased in Liaison. Daily caseloads decreased in HTT and Liaison services, and cancelled or non-attended appointments reduced in Liaison services.

For peer review only - http://bmjopen.bmj.com/site/about/guidelines.xhtml

There was moderate to substantial heterogeneity in the majority of our IRRs estimates across the three periods (i.e., before, during, and after lockdown) in number of deaths, inpatient caseloads, f2f and non-f2f contacts and daily caseloads for all community services. For example, IRRs for number of deaths on transition into lockdown ranged from 1.55 (1.23-1.94) to 5.30 (2.87-9.79) (Figure 1). All IRRs are provided individually by site in Supplementary Tables 1-3. Inpatient caseload reductions on the transition into lockdown varied in IRRs from 0.67 (0.65-0.69) to 1.00 (0.99-1.01), reductions in HTT caseloads from 0.42 (0.39-0.45) to 0.98 (0.97-0.99), Liaison caseload reductions from 0.35 (0.31-0.39) to 1.03 (1.00-1.06). IRRs for the transition from before lockdown to lifting of lockdown (Supplementary Table 3) also varied, with daily deaths ranging from 0.31 (0.22-0.46) to 1.64 (1.29-2.09), those for inpatient caseloads from 0.87 (0.85-0.89) to 1.08 (1.07-1.09), HTT caseloads from 0.17 (0.16-0.18) to 0.91 (0.89-0.92), and Liaison caseloads from 0.41 (0.36-0.47) to 1.06 (1.03-1.10).

For Site I, where extractions of numbers of deaths and service activity data were weekly rather than daily and could not therefore be combined with other sites, there was a significant increase in weekly deaths (IRR 1.47 (1.35-1.60)) at lockdown, and a decrease (IRR 0.72 (0.66-0.80)) after the lifting of lockdown. Slightly, but non-significantly, increased numbers of deaths were observed after the lifting of lockdown compared to before lockdown. There was a reduction in admissions (IRR 0.64 (0.58-0.71)) and discharges (IRR 0.83 (0.73-0.94)) at lockdown, but no differences after the lifting of lockdown compared to before lockdown.

Sensitivity analyses

1. Further data for the post- vs. pre-lockdown comparisons are presented in Table 3 for the four sites able to extract daily data to 31st July. Considering the findings that were most consistently significant, all showed reduced total inpatient caseload, reduced OA f2f contacts, increased non-f2f AMH and EIP contacts, and reduced CAMHS, Liaison and OA caseloads.

2. When excluding data for one week around the lockdown and lift-of-lockdown announcements, minimal changes were observed, and IRRs remained largely similar (Supplementary Table 4).

For peer review only - http://bmjopen.bmj.com/site/about/guidelines.xhtml

3. Further adjustment for levels of national COVID-19 mortality levels (Supplementary Table 5) resulted in some dilution in the strength of IRRs for deaths, new admissions and daily caseloads at lockdown initiation, but minimal changes to those IRRs that were statistically significant. A few IRRs for the lift-of-lockdown transition fell below statistical significance (increases in inpatient admissions, in OA f2f contacts), as did some 'after vs. before' lockdown differences (reductions in Trust new referrals and inpatient discharges; increases in CAMHS total contacts).

4. Additionally, no substantial changes were observed in our IRRs when we employed different equally spaced exposure time window intervals around the lockdown and lift-of-lockdown announcements (Supplementary Table 6).

5. Further adjustment for Google trends index (Supplementary Table 7) resulted in some dilution in the strength of IRRs for non-f2f contacts across services at lockdown initiation and lift-of-lockdown transition, but findings remained statistically significant. No substantial changes were observed in the other IRRs.

For peer review only - http://bmjopen.bmj.com/site/about/guidelines.xhtml

DISCUSSION

Summary of findings

To our knowledge, this is the first multi-site study to present data and evaluate the impact of the COVID-19 'lockdown' policy in the UK on mortality and MH service activity provision. In summary, the initiation of lockdown was associated with increases in the death rate, decreases in new accepted referrals, and decreases in inpatient admissions and daily caseload. In community services, sizeable increases were observed in non-f2f contacts, with decreases in f2f contacts. Caseloads decreased in CAMHS, HTT, Liaison and OA services, but increased slightly in AMH and EIP services. The lift-of-lockdown transition was associated with decreased deaths, increased referrals and inpatient admissions and decreased inpatient discharges. In community settings, most services saw increases in non-f2f contacts and total contacts. There was moderate to high heterogeneity of these estimates across sites; only site-wide new referrals/discharges, and inpatient admissions/discharges were consistent.

Impact of the COVID-19 first wave

The potential impact of the COVID-19 pandemic has been widely discussed in a general sense, focusing on the initial priorities of infection control, treatment options for severe complications, and the preparedness of critical care services (25). For mental healthcare, there is a need to understand the population-level impact of both the viral infection and the social distancing being imposed by many national governments (9, 26, 27), as well as concerns about higher levels of population distress coupled with reduced use/availability of psychological support (28). For people with pre-existing mental disorders, there is also a concern that vulnerability to COVID-19 infection may be higher than expected, because of infection susceptibility due to physical comorbidities and because of barriers to health service access (29, 30). A higher risk of relapse of mental disorders is a concern due to the stress of the pandemic itself, the stress of consequent quarantine (31), reduced access to routine outpatient visits for evaluations and prescriptions, and possibly also an avoidance of health services because of the perceived risk of infection or a wish to 'protect the NHS'. Finally, a higher risk of suicide might conceivably result from rapid social, economic, and health changes (32, 33). On the other hand, it is possible that the pandemic may have had positive effects on mental health and the reduced use of services might reflect reduced need – for example, arising from increased family contact at home during 'lockdown', and/or reduced exposure to social pressures outside the home, and/or a sense of a more supportive

For peer review only - http://bmjopen.bmj.com/site/about/guidelines.xhtml

community or shared adversity. Either way, the scale of mental healthcare changes has not yet been fully quantified beyond single site findings, although recommendations made in China for tighter admission criteria and reduced hospital outpatient visits, amongst others (34), may reflect similar changes in service provision to those we report.

While it was not our intention here to investigate factors underlying the observed service changes, many are likely to be unsurprising. Clearly, a reduction in f2f clinical contacts was a likely outcome of social distancing, plus rising concern about the infection risks to patients and staff of such contacts. These were balanced by an increase in non-f2f contacts, and the only reductions after 23rd March 2020 in total contacts were seen in Liaison and HTT services. These also saw strongest reductions in daily caseload, which for Liaison services may reflect high numbers of discharges from acute hospitals to prepare for a surge in pandemic-related demand, and possibly patient reluctance to attend emergency care. While HTT caseload reductions might reflect reduced demand, the levels and consequences of unmet need during the lockdown require further evaluation. Of interest, rates of cancelled or non-attended appointments did not change significantly for most services. On the face of it, this is reassuring, given the sizeable switch towards non-f2f contacts. However, the findings should be viewed with some caution, as it is not yet clear whether unsuccessful attempts at non-f2f contacts are recorded in the same way as a non-attended f2f appointment. Furthermore, longer-term trends beyond the rather unusual context of the 'first-wave' lockdown need additional evaluation.

Between-site variation

An important feature of the findings was their heterogeneity between sites. Mainly this reflected magnitude rather than direction of effect: for example, all sites saw increases in deaths around lockdown initiation (Figure 1, Supplementary Table 1), although IRRs varied in size. This heterogeneity may reflect site differences in patient demographics and pandemic timing across the country. IRRs for inpatient caseload changes at the same transition point may reflect differences in facilities – for example, strongest caseload reductions were seen at Site A which had highest pre-lockdown levels (Table 1). On the other hand, reductions in HTT caseloads were weakest at Site H where pre-lockdown caseloads were highest, and Liaison caseload reductions differed substantially between sites (B and H) with similar pre-lockdown caseloads; this might reflect local differences in acute hospital practice/demand and indicates scope for further investigation.

For peer review only - http://bmjopen.bmj.com/site/about/guidelines.xhtml

Causality considerations

It is challenging to disentangle the effect of the ongoing COVID-19 pandemic itself from those of social distancing/lockdown policies (or other psychosocial consequences) on MH service activity and mortality. Our estimates retrieved from RDiT are of a compound effect: the causal effect of the 23rd March 2020 lockdown announcement on mental health service activity and any unobserved sorting/anticipation/adaptation/avoidance effects that may exist but cannot be tested for. The extent to which the results should be interpreted solely as the causal treatment effect of interest depend on the likelihood of other influences. For example, observed increases in mortality are likely to represent effects of the ongoing pandemic rather than lockdown policy; whereas for the service activity measures, adjustment for national mortality (sensitivity analysis 3) is less of an issue and we consider those results to be robust. However, we did not attempt to capture the variation in the timing or magnitude of infection and mortality rates across the UK.

The estimation of mortality increase in our analysis is clearly reflecting a before-after comparison with no population control, and is also restricted to all-cause mortality as an outcome with no information available across sites on causes of death responsible. However, changes in all-cause mortality are as valid as those of specific COVID-19 mortality because the former encompasses a wider range of impacts beyond the direct effects of the infection, including adverse consequences of reduced access to medical care as well as suicide mortality as a consequence of reduced mental health support. A report from one of the constituent sites indicates that most of the excess mortality in mental health service users during March to June 2020 was from deaths attributed to COVID-19, as well as small excesses in deaths with dementia as an underlying cause (35). In that study, there was also an excess in unexplained deaths compared to previous years, many of which were likely to involve cases awaiting inquests, although these will not necessarily result in suicide verdicts, and estimates to date (for the UK as well as a number of other nations) have not indicated increased suicide rates, at least in the early stages of the pandemic (36), and non-fatal self-harm presentations have also been reported as relatively low during that early period (18, 37).

Strengths and limitations

Study strengths include the use of relatively ‘real-time’ mental healthcare data from ten large providers or routinely collected datasets, allowing investigation of changes in service activity

For peer review only - http://bmjopen.bmj.com/site/about/guidelines.xhtml

following dramatic and rapid transitions at a national scale. The size of the data permitted precise estimates and the relative consistency in the direction of findings supports generalisability. Here we report on what might be termed a 'natural experiment'; while individuals were not randomly assigned to experience lockdown or not, assignment is assumed to be quasi-random for observations close to the cut-off . Our regression discontinuity design approach allows valid causal effects to be identified (38) and has being previously employed with electronic health record data (39). Limitations include that data were drawn from specific services of interest and do not reflect the full activity of the ten sites; they were also combined by service type and we did not seek to investigate within-service (team-level) variation. Daily contact numbers were quantified from structured fields in electronic health records and might reflect recording behaviour rather than activity levels (e.g. if multiple contacts were subsumed within one entry); also, the dichotomy between f2f and non-f2f contact is a relatively crude one and does not reflect the quality or depth of assessments being recorded, nor the platform that was used (e.g. telephone vs. video). While we sought to harmonise data extractions as much as was feasible, there were inevitable differences in data availability between sites and likely variation in some measurements (e.g. in the completeness of mortality data in clinical systems), although we believe there should be robust within-site consistency across the time periods examined. Some IRR heterogeneity may reflect differences in service provision (e.g. acute hospital beds covered by a given Liaison service). Clearly the observation period was limited in duration and there will be a continuing need to collect data to evaluate potential longer-term consequences of both the first and subsequent pandemic waves. Finally, it is important to emphasise that findings here represent quantitative estimates of care provision, rather than care as experienced by patients; there is therefore an important need to evaluate patient satisfaction and perception of care, and changes in care delivery, before drawing conclusions regarding beneficial or adverse consequences.

Implications

The alterations in MH service activity observed here have been profound and reflect the very limited time period over which to implement responses to the COVID-19 'lockdown' policy. The sharp downturn in inpatient admissions and f2f community contacts are potentially concerning, as is the apparently much longer-term shift in balance from f2f to virtual contacts which remains a marked but unevaluated change in care. On the other hand, as argued above, reduced inpatient admissions and crisis service contact might potentially reflect a reduced

For peer review only - http://bmjopen.bmj.com/site/about/guidelines.xhtml

demand due to better mental health, and virtual contacts might represent an option of care with potential advantages in the future, extending opportunities for patient choice and reducing travel and time costs for both patients and clinical services. Predicting the continuing impact of COVID-19 and related infection-control policies on MH service provision and demand remains challenging. For example, while national 'shared' emergencies may be experienced as less isolating for people with mental disorders, and some consequences may be less problematic than feared (32), treatment pathways are likely to have been disrupted significantly, particularly since social distancing/lockdown policies were imposed (continuously or intermittently) over many months. It is therefore imperative that national and international collaborations are established to monitor MH service needs and experiences of care as closely as possible, to identify adverse outcomes early enough for rapid intervention – in particular, to understand how people with new and existing severe mental health conditions can best be supported in a pandemic, given the need for socially distanced care input.

Ethics statement

The authors assert that all procedures contributing to this work comply with the ethical standards of the relevant national and institutional committees on human experimentation and with the Helsinki Declaration of 1975, as revised in 2008. The extractions were carried out as service evaluations (http://www.hra-decisiontools.org.uk/research/) with appropriate local information governance approval. Those for Cambridge and Peterborough, and South London and Maudsley sites used CRATE and CRIS data platforms respectively which have been pre-approved for both service evaluation and research purposes (REC references 17/EE/0442 and 18/SC/0372 respectively). Those for Wales sites received Information Governance Research Panel approval (SAIL 0911).

For peer review only

For peer review only - http://bmjopen.bmj.com/site/about/guidelines.xhtml

REFERENCES

1. Kontis V, Bennett JE, Rashid T, Parks RM, Pearson-Stuttard J, Guillot M, et al. Magnitude, demographics and dynamics of the effect of the first wave of the COVID-19 pandemic on all-cause mortality in 21 industrialized countries. Nature Medicine. 2020; https://doi.org/10.1038/s41591-020-1112-0.

2. Chang CK, Hayes RD, Perera G, Broadbent MTM, Fernandes AC, Lee WE, et al. Life expectancy at birth for people with serious mental illness, substance use disorders, and depressive disorders from a secondary mental health care case register in London, UK. PLoS One. 2011; 6: e19590.

3. Woodhead C, Ashworth M, Broadbent M, Callard F, Hotopf M, Schofield P, et al. Cardiovascular disease treatment among severe mental illness patients: a data linkage study between primary and secondary care. British Journal of General Practice. 2016; 66: e374-e81.

4. Woodhead C, Cunningham R, Ashworth M, Barley E, Stewart R, Henderson M. Cervical and breast cancer screening uptake among women with serious mental illness: a data linkage study. BMC Cancer. 2016; 16: 819.

5. WHO. The impact of COVID-19 on mental, neurological and substance use services: results of a rapid assessment. World Health Organization. 2020; https://www.who.int/publications/i/item/978924012455.

6. Thome J, J. D, Coogan AN, Bailey-Rodriguez D, da Cruz E Silva OAB, Faltraco F, et al. The impact of the early phase of the COVID-19 pandemic on mental-health services in Europe. . World Journal of Biological Psychiatry. 2020; Dec 23: 1-10. doi: .1080/15622975.2020.1844290.

7. Leigh-Hunt N, Bagguley D, Bash K, Turner V, Turnbull S, Valtorta N, et al. An overview of systematic reviews on the public health consequences of social isolation and loneliness. Public Health. 2017; 152: 157-71.

8. Gayer-Anderson C, Latham R, El Zerbi C, Strang L, Moxham Hall V, Knowles G, et al. Impacts of social isolation among disadvantaged and vulnerable groups during public health crises. ESRC. 2020; https://esrc.ukri.org/files/news-events-and-publications/evidence-

For peer review only

briefings/impacts-of-social-isolation-among-disadvantaged-and-vulnerable-groups-during-public-health-crises/.

9. Holmes EA, O'Connor RC, Perry VH, Tracey I, Wessely S, Arseneault L, et al. Multidisciplinary research priorities for the COVID-19 pandemic: a call for action for mental health science. Lancet Psychiatry. 2020; Apr 15: https://doi.org/10.1016/S2215-0366(20)30168-1.

10. Torales J, O'Higgins M, Castaldelli-Maia JM, Ventriglio A. The outbreak of COVID-19 coronavirus and its impact on global mental health. International Journal of Social Psychiatry. 2020; 66(4): 317-20.

11. Lai J, Ma S, Wang Y, Cai Z, Hu J, Wei N, et al. Factors Associated With Mental Health Outcomes Among Health Care Workers Exposed to Coronavirus Disease 2019. JAMA Network Open. 2020; 3(3): e203976.

12. Livingston G, Rostamipour H, Gallagher P, Kalafatis C, Shastri A, Huzzey L, et al. Prevalence, management, and outcomes of SARS-CoV-2 infections in older people and those with dementia in mental health wards in London, UK: a retrospective observational study. Lancet Psychiatry. 2020; doi: https://doi.org/10.1016/S2215-0366(20)30434-X.

13. Johnson S, Dalton-Locke C, Vera San Juan N, Foye U, Oram S, Papamichail A, et al. Impact on mental health care and on mental health service users of the COVID-19 pandemic: a mixed methods survey of UK mental health care staff. Social Psychiatry and Psychiatric Epidemiology. 2020.

14. Chen S, Jones PB, Underwood BR, Moore A, Bullmore ET, Banerjee S, et al. The early impact of COVID-19 on mental health and community physical health services and their patients' mortality in Cambridgeshire and Peterborough, UK. Journal of Psychiatric Research. 2020; 22 Sep 2020: https://doi.org/10.1016/j.jpsychires.2020.09.020.

15. Stewart R, Martin E, Bakolis I, Broadbent M, Byrne N, Landau S. Comparison of mental health service activity before and shortly after UK social distancing responses to the COVID-19 pandemic: February-March 2020. medRxiv. 2020; 28 Sep 2020: https://www.medrxiv.org/content/10.1101/2020.09.26.20202150v1.

For peer review only

16. Stewart R, Broadbent M, Das-Munshi J. Excess mortality in mental health service users during the COVID-19 pandemic described by ethnic group: South London and Maudsley data. medRxiv. 2020; 14 Jul 2020: https://medrxiv.org/cgi/content/short/2020.07.13.20152710v1.

17. Stewart R, Martin E, Broadbent M. Mental health service activity during COVID-19 lockdown: South London and Maudsley data on working age community and home treatment team services and mortality from February to mid-May 2020. medRxiv. 2020; 16 Jun 2020: https://www.medrxiv.org/content/10.1101/2020.06.13.20130419v1

18. Chen S, She R, Qin P, Kershenbaum A, Fernandez-Egea E, Nelder JR, et al. The medium-term impact of COVID-19 lockdown on referrals to secondary care mental health services: a controlled interrupted time series study. Frontiers in Psychiatry. 2020; 26 Nov: doi: 10.3389/fpsyt.2020.585915.

19. Tromans S, Chester V, Harrison H, Pankhania P, Booth H, Chakraborty N. Patterns of use of secondary mental health services before and during COVID-19 lockdown: observational study. BJPsych Open. 2020; 6: e117.

20. Hausman C, Rapson DS. Regression discontinuity in time: considerations for empirical applications. Davis Energy Economics Program. 2018; April: http://deep.ucdavis.edu/uploads/5/6/8/7/56877229/deep_wp019.pdf.

21. Grembi V, Nannicini T, Troiano U. Policy Responses to Fiscal Restraints: A Difference-inDiscontinuities Design. 2012: https://www.princeton.edu/rppe/speaker-series/speakerseries-2012-13/Troiano.pdf.

22. DerSimonian R, Laird N. Meta-analysis in clinical trials. Controlled Clinical Trials. 1986; 7: 177-88.

23. Jeffrey B, Walters CE, Ainslie KEC, Eales O, Ciavarella C, Bhatia S, et al. Anonymised and aggregated crowd level mobility data from mobile phones suggests that initial compliance with COVID-19 social distancing interventions was high and geographically consistent across the UK. Imperial College London. 2020; 29 May 2020: https://doi.org/10.25561/79387.

For peer review only - http://bmjopen.bmj.com/site/about/guidelines.xhtml

24. Brodeur A, Clark AE, Flèche S, Powdthavee N. COVID-19, lockdowns and well-being: evidence from Google Trends. . Centre for Economic Performance. 2020; Discussion Paper No 1693: https://cep.lse.ac.uk/pubs/download/dp1693.pdf.

25. Adalja AA, Toner E, Inglesby TV. Priorities for the US health community responding to COVID-19. Journal of the American Medical Association. 2020; 323: 1343-4.

26. WHO. Mental health and psychosocial considerations during the COVID-19 outbreak. World Health Organization. 2020; https://www.who.int/docs/default-source/coronaviruse/mental-health-considerations.pdf?sfvrsn=6d3578af_2.

27. Auerbach J, Miller BF. COVID-19 exposes the cracks in our already fragile mental health system. American Journal of Public Health. 2020; Apr 9: doi:10.2105/AJPH.020.305699.

28. Davies J. What impact has Covid-19 had on mental health services? Nuffield Trust QualityWatch. 2020; Nov 30: https://www.nuffieldtrust.org.uk/news-item/what-impact-has-covid-19-had-on-mental-health-services.

29. Yao H, Chen JH, Xu YF. Patients with mental health disorders in the COVID-19 epidemic. Lancet Psychiatry. 2020; 7: e21.

30. Wang Q, Xu R, Volkow ND. Increased risk of COVID-19 infection and mortality in people with mental disorders: analysis from electronic health records in the United States. . World Psychiatry. 2020; 7 October: DOI: 10.1002/wps.20806.

31. Brooks SK, Webster RK, Smith LE, Woodland L, Wessely S, Greenberg N, et al. The psychological impact of quarantine and how to reduce it: rapid review of the evidence. Lancet. 2020; 395: 912-20.

32. Reger MA, Stanley IH, Joiner TE. Suicide mortality and coronavirus disease 2019 - a perfect storm? JAMA Psychiatry. 2020; April 10: doi:10.1001/jamapsychiatry.2020.1060.

33. Gunnell D, Appleby L, Arensman E, Hawton K, John A, Kapur N, et al. Suicide risk and prevention during the COVID-19 pandemic. Lancet Psychiatry. 2020; 7: 468-71.

For peer review only

34. Xiang YT, Zhao YJ, Liu ZH, Li XH, Zhao N, Cheung T, et al. The COVID-19 outbreak and psychiatric hospitals in China: managing challenges through mental health service reform. International Journal of Biological Sciences. 2020; 16: 1741-4.

35. Stewart R, Jewell A, Broadbent M, Bakolis I, Das-Munshi J. Causes of death in mental health service users during the first wave of the COVID-19 pandemic: South London and Maudsley data from March to June 2020, compared with 2015-2019. medRxiv. 2020: doi: 10.1101/2020.10.25.20219071.

36. Appleby L. What has been the effect of covid-19 on suicide rates? BMJ. 2021; 372: n834.

37. Nuzum E, Martin E, Morgan G, Dutta R, Mueller C, Polling C, et al. Self-harm presentations to Emergency Departments and Place of Safety during the first wave of the UK COVID-19 pandemic: South London and Maudsley data on service use from February to June 2020. . medRxiv. 2020: doi: 10.1101/2020.12.10.20247155.

38. Bor J, Moscoe E, Mutevedzi P, Newell ML, Barnighausen T. Regression discontinuity designs in epidemiology: causal inference without randomized trials. Epidemiology. 2014; 25(5): 729-37.

39. Bakolis I, Kelly R, Fecht D, Best N, Millett C, Garwood K, et al. Protective effects of smoke-free legislation on birth outcomes in England: a regression discontinuity design. Epidemiology. 2016; 27: 810-8.

For peer review only - http://bmjopen.bmj.com/site/about/guidelines.xhtml

Table 1. Descriptive characteristics of daily mortality and mental health service caseloads by site

Measures	Site	Before lockdown* Median (IQR)	During lockdown* Median (IQR)	After lockdown* Median (IQR)
Number of deaths	A	13 (11-17)	30 (22-38)	13 (11-16)
	B	44 (39-51)	67 (61-78)	40 (29-47)
	C	0 (0-0)	0 (0-1)	0 (0-0)
	D	25 (22-28)	42 (28-50)	7 (4-18)
	E	2 (0-3)	3 (0-6)	0 (0-1)
	F	12 (9-14)	18 (12-21)	13 (10-16)
	G	7 (6-9)	10 (7-12)	5 (4-7)
	H	3 (2-4)	4 (3-6)	3 (2-3)
	I	95 (93-100) ^	130 (112-146) ^	85 (80-88) ^
	J	10 (7-13)	18 (14-26)	11 (8-12)
Inpatient caseload	A	768 (762-774)	556 (544-567)	581 (568-588)
	B	454.5 (445-461)	363 (357-368)	375 (374-379)
	C	495 (489-503)	427.5 (422-443.5)	451 (443-454)
	D	701 (696-707)	625 (617-657)	623 (613-640)
	E	481 (471-491)	412 (400-418)	453 (437-463)
	F	207 (205-211)	140 (135-146)	147 (146-148)
	H	408 (399-412)	351 (338-360)	367 (358-381)
	J	400 (394-407)	361 (354-371)	375 (365-385)
AMH (community) caseload	A	8691 (8666-8714)	8504 (8438-8576)	8398 (8389-8409)
	B	8700 (8652-8739)	8039 (7823-8297)	7555 (7498-7587)
	D	11039.5 (10956-11091)	10803 (10688-10923)	10526 (10520-10559)
	F	2228 (2221-2242)	2178 (2167-2195)	2158 (2156-2162)
	H	724.5 (719-726)	695 (686-700)	684 (683-686)
	J	10687 (10506-10981)	10160 (9866-10519)	9599 (9529-9612)
CAMHS caseload	A	6915.5 (6792-7021)	6962 (6875-6997)	6789 (6752-6801)
	D	10240.5 (10187-10292)	10060 (9935-10189)	9807 (9795-9813)
	E	38 (37-38)	35 (35-35)	35 (35-35)
	F	2232 (2176-2247)	2106 (2061-2144)	1992 (1988-1999)
	H	4375.5 (4347-4445)	4383 (4234-4414)	4106 (4078-4137)
	J	15946.5 (15610-16046)	14821 (14342-15394)	13835 (13802-13942)
EIP caseload	A	1193 (1176-1208)	1190 (1185-1198)	1206 (1199-1208)
	B	354.5 (347-358)	333 (329-343)	336 (335-341)
	D	809 (805-812)	794 (790-806)	806 (804-810)
	E	862 (857-867)	852 (849-853)	860 (859-862)
	F	93 (92-96)	97 (96-98)	95 (93-95)
	H	768 (765-771)	767 (762-771)	765 (763-768)
	J	421 (415-424)	421 (419-423)	421 (415-424)
HTT caseload	A	214.5 (207-222)	153 (148-158)	191 (187-197)
	B	258.5 (241-276)	151 (139-161)	198 (188-202)
	D	263 (247-289)	162 (151-177)	206 (197-214)
	E	219 (202-234)	95 (87-103)	127 (121-133)
	F	73 (64-77)	54 (50-62)	56 (50-59)
	H	977.5 (968-996)	780 (715-850)	732 (725-740)
	J	354.5 (293-418)	100 (60-165)	50 (48-51)
Liaison caseload	A	1060 (1044-1074)	971 (956-980)	1005 (999-1015)
	B	80.5 (69-91)	27 (21-29)	47 (39-51)
	D	1851.5 (1786-1879)	1523 (1494-1573)	1505 (1493-1522)
	E	103 (90-110)	48 (42-65)	81 (75-86)
	F	499 (468-516)	407 (403-411)	448 (436-455)
	H	59.5 (57-62)	64 (63-68)	71 (68-74)
	J	221 (216-227)	203 (193-211)	200 (197-202)
OA caseload	A	1297 (1264-1308)	1065 (1018-1126)	1001 (991-1007)
	B	6243 (6193-6257)	5518 (5336-5746)	5149 (5132-5191)
	D	5731.5 (5677-5791)	5497 (5398-5596)	5346 (5338-5352)
	E	567 (561-582)	503 (497-508)	517 (511-518)
	F	502 (487-507)	350 (340-386)	344 (342-345)
	H	1638 (1621-1654)	1328 (1288-1383)	1261 (1259-1263)
	J	3844 (3821-3857)	3426 (3337-3557)	3284 (3280-3286)

*Before lockdown: 01.01.2020-22.03.2020; During lockdown: 23.03.2020-09.05.2020; After lockdown: 10.05.2020-31.05.2020
AMH: adult mental health service; CAMHS: child and adolescent mental health service; EIP: early intervention for psychosis service; HTT: home treatment team; OA: older adult service. ^For Site I we ascertained median and IQR for weekly mortality and have divided each estimate here by 7 for comparability.

For peer review only

Table 2. Estimated effects of lockdown and lift-of-lockdown announcements on service measures meta-analysed across sites A-H and J from 1st Jan to 31st May 2020, estimating transitions related to the lockdown announcement (23rd March) and lift-of-lockdown announcement (10th May). Incidence Rate Ratios (IRRs) and 95% confidence intervals and percentage heterogeneity across sites (I^2) were estimated.

	Lockdown Announcement vs Pre-lockdown		Lift of Lockdown announcement vs Lockdown		Lift of Lockdown vs. Before Lockdown	
**Measures**	**IRR (95%CI)**	**I^2**	**IRR (95%CI)**	**I^2**	**IRR (95%CI)**	**I^2**
Number of deaths	2.31*(1.86-2.87)	85.3	0.42*(0.27-0.66)	91.8	0.97 (0.68-1.38)	88.5
Trust-wide activity						
Number of new referrals accepted	0.62*(0.55-0.70)	0.0	1.36*(1.15-1.60)	0	0.85*(0.76-0.95)	0.0
Number of discharges	0.95 (0.85-1.06)	0.0	0.95 (0.81-1.11)	0	0.89 (0.80-1.00)	0.0
Inpatient care						
New admissions	0.75*(0.67-0.83)	0.0	1.21*(1.04-1.42)	0	0.90 (0.79-1.03)	0.0
Discharges	1.13 (0.97-1.31)	0.0	0.66*(0.53-0.84)	5.2	0.75*(0.62-0.90)	0.0
Inpatient caseload	0.85*(0.79-0.91)	99.6	1.06 (1.00-1.12)	99.0	0.90*(0.83-0.97)	99.7
Inpatient caseload on a MHA section	0.93^(0.87-0.99)	99.4	1.01 (0.93-1.10)	99.6	0.94 (0.86-1.02)	99.8
AMH (community)						
F2f contacts	0.55*(0.44-0.68)	49.4	0.99 (0.80-1.23)	0	0.57*(0.44-0.75)	40.2
Non-f2f contacts	3.74*(2.58-5.43)	83.2	1.27*(1.06-1.52)	0	3.95*(2.73-5.71)	70.3
F2f and Non-f2f contacts	1.07 (0.94-1.22)	0.0	1.12 (0.94-1.34)	0	1.19*(1.04-1.36)	0.0
Cancelled appointments or DNAs	0.85 (0.61-1.18)	73.1	1.02 (0.80-1.30)	10.6	0.79(0.56-1.11)	61.7
Caseload	1.03^(1.01-1.05)	98.2	1.01 (1.00-1.02)	94.6	1.04*(1.02-1.04)	0.0
CAMHS						
F2f contacts	0.35*(0.17-0.74)	93.2	1.12 (0.79-1.61)	14.5	0.36*(0.15-0.88)	88.8
Non-f2f contacts	3.58*(2.54-5.04)	70.2	1.23 (1.00-1.50)	0	3.76*(2.69-5.25)	50.1
F2f and Non-f2f contacts	1.08 (0.93-1.26)	0.0	1.20 (0.96-1.49)	0	1.23*(1.04-1.47)	0.0
Cancelled appointments or DNAs	0.87(0.67-1.14)	48.8	0.94 (0.65-1.36)	41.2	0.89 (0.76-1.04)	0.0
Caseload	0.93^(0.89-0.97)	99.2	1.03 (0.98-1.08)	99.3	0.94 (0.88-1.01)	99.5
EIP						
F2f contacts	0.27*(0.18-0.42)	86.0	1.64*(1.19-2.27)	48.4	0.45*(0.29-0.70)	82.5
Non-f2f contacts	4.04*(2.63-6.20)	83.3	1.48*(1.03-2.12)	52.9	5.51*(3.08-9.84)	87.5
F2f and Non-f2f contacts	1.09 (0.88-1.34)	51.6	1.45*(1.22-1.72)	0	1.51*(1.22-1.88)	49.7
Cancelled appointments or DNAs	0.76 (0.54-1.08)	74.4	0.99 (0.72-1.36)	44.3	0.70 (0.44-1.12)	85.2
Caseload	1.04^(1.02-1.06)	97.9	1.04 (1.00-1.08)	99.5	1.08*(1.03-1.13)	99.8
HTT						
F2f contacts	0.43*(0.32-0.57)	97.0	1.49*(1.36-1.62)	37.5	1.91*(1.49-2.43)	84.3
Non-f2f contacts	1.78*(1.56-2.03)	60.3	1.07 (0.87-1.32)	69.6	0.99 (0.87-1.14)	85.9
F2f and Non-f2f contacts	0.78*(0.68-0.89)	90.3	1.27*(1.16-1.39)	52.6	1.08 (0.87-1.34)	58.3
Cancelled appointments or DNAs	0.68*(0.51-0.89)	85.6	1.47*(1.11-1.96)	67.9	0.62*(0.43-0.88)	99.8
Caseload	0.62*(0.49-0.78)	99.7	1.01 (0.78-1.29)	99.4	0.64*(0.51-0.82)	94.4
Liaison						
F2f contacts	0.43*(0.36-0.50)	78.2	1.67*(1.39-2.00)	59.0	0.72*(0.61-0.86)	66.3
Non-f2f contacts	1.50*(1.15-1.96)	68.8	1.11 (0.81-1.53)	52.5	1.69*(1.16-2.47)	74.2
F2f and Non-f2f contacts	0.60*(0.54-0.66)	44.5	1.46*(1.29-1.65)	28.3	0.85^(0.74-0.98)	56.7
Cancelled appointments or DNAs	0.62(0.35-1.10)	84.5	0.75 (0.46-1.24)	45.9	0.65*(0.43-0.97)	41.6
Caseload	0.75*(0.69-0.81)	99.1	1.14*(1.07-1.21)	97.7	0.88*(0.83-0.93)	97.9
OA						
F2f contacts	0.32*(0.20-0.49)	83.8	1.45*(1.13-1.85)	0.35	0.39*(0.23-0.67)	82.3
Non-f2f contacts	3.66*(2.54-5.27)	78.7	1.28^(1.02-1.59)	0	4.27*(2.78-6.56)	73.3
F2f and Non-f2f contacts	1.06 (0.81-1.37)	62.5	1.31*(1.06-1.63)	0	1.28 (0.94-1.73)	50.8
Cancelled appointments or DNAs	1.08 (0.73-1.58)	77.9	0.70 (0.38-1.31)	75.8	0.80 (0.51-1.26)	63.1
Caseload	0.95 (0.92-0.99)	99.3	0.99 (0.96-1.01)	98.7	0.93 (0.84-1.03)	99.8
**Site I data†**						
Number of deaths	1.47*(1.35-1.60)		0.72*(0.66-0.80)		1.07 (0.99-1.15)	
New inpatient admissions	0.64*(0.58-0.71)		1.67*(1.48-1.88)		1.07 (0.93-1.22)	
Inpatient discharges	0.83^(0.73-0.94)		1.19*(1.08-1.32)		0.99 (0.88-1.10)	

^p<0.05; *p<0.001

†Derived from weekly data from 1st Jan to 5th Jul 2020.

AMH: adult mental health service; CAMHS: child and adolescent mental health service; EIP: early intervention for psychosis service; HTT: home treatment team; OA: older adult service.

For peer review only - http://bmjopen.bmj.com/site/about/guidelines.xhtml

724

Table 3. Sensitivity analyses of lift-of-lockdown vs. pre-lockdown comparisons for the extended period of follow-up (to 31st July 2020) available for four sites. Incidence Rate Ratios (IRRs) and 95% confidence intervals were estimated.

	Site A<br>Lift of the Lockdown vs. Pre-lockdown	Site F<br>Lift of the Lockdown vs. Pre-lockdown	Site G<br>Lift of the Lockdown vs. Pre-lockdown	Site J<br>Lift of the Lockdown vs. Pre-lockdown
**Measures**	**IRR (95%CI)**	**IRR (95%CI)**	**IRR (95%CI)**	**IRR (95%CI)**
Number of deaths	1.20 (0.95-1.51)	2.06*(1.09-3.90)	0.89 (0.65-1.20)	1.08 (0.84-1.40)
Trust-wide activity				
Number of new referrals accepted	0.79*(0.68-0.93)	0.55^(0.30-0.98)	N/A	0.73 (0.50-1.07)
Number of discharges	0.83*(0.72-0.96)	0.60 (0.31-1.16)	N/A	0.74 (0.51-1.06)
Inpatient care				
New admissions	0.94 (0.75-1.18)	0.34 (0.06-1.83)	N/A	0.94 (0.62-1.42)
Discharges	0.64*(0.48-0.86)	0.24 (0.03-2.22)	N/A	0.81 (0.51-1.28)
Inpatient caseload	0.76*(0.74-0.77)	0.62*(0.59-0.66)	N/A	0.96^(0.95-0.97)
Inpatient caseload on a MHA section	0.87*(0.85-0.88)	1.02 (0.96-1.09)	N/A	1.01 (0.99-1.02)
Adult mental health (community)				
F2f contacts	0.50*(0.38-0.67)	0.44 (0.05-4.32)	0.31*(0.18-0.54)	0.47*(0.32-0.69)
Non-f2f contacts	2.99*(2.45-3.64)	12.11*(1.64-89.29)	13.66*(7.77-24.03)	8.69*(6.22-12.15)
F2f and Non-f2f contacts	1.28*(1.08-1.53)	0.97 (0.11-8.55)	0.83 (0.47-1.46)	1.68*(1.16-2.42)
Cancelled appointments or DNAs	0.70*(0.58-0.85)	0.39 (0.04-3.53)	0.73 (0.43-1.23)	1.06 (0.67-1.68)
Caseload	0.94^(0.91-0.99)	1.04^(1.02-1.04)	N/A	0.92*(0.90-0.93)
CAMHS				
F2f contacts	0.14*(0.08-0.26)	1.70 (0.17-16.59)	0.65 (0.40-1.03)	0.08*(0.05-0.14)
Non-f2f contacts	3.60*(2.96-4.37)	4.78 (0.77-29.85)	16.12*(9.28-27.99)	3.32*(2.19-5.04)
F2f and Non-f2f contacts	1.27*(1.03-1.56)	1.98 (0.22-17.86)	1.15 (0.75-1.77)	1.35 (0.89-2.05)
Cancelled appointments or DNAs	0.90 (0.74-1.10)	0.59 (0.06-5.61)	0.46*(0.25-0.83)	0.59*(0.38-0.93)
Caseload	0.95^(0.91-0.97)	0.96*(0.95-0.97)	N/A	0.84*(0.82-0.85)
EIP				
F2f contacts	0.20*(0.16-0.26)	0.79 (0.10-6.43)	N/A	0.22*(0.16-0.31)
Non-f2f contacts	4.22*(3.39-5.25)	35.59*(3.38-74.33)	N/A	13.90*(9.05-21.37)
F2f and Non-f2f contacts	1.39*(1.16-1.67)	1.32 (0.18-9.73)	N/A	1.95*(1.59-2.39)
Cancelled appointments or DNAs	0.55*(0.43-0.71)	0.86 (0.09-7.78)	N/A	1.15 (0.77-1.73)
Caseload	0.95^(0.92-0.98)	1.07*(1.05-1.08)	N/A	0.98^(0.96-0.99)
HTT				
F2f contacts	0.68*(0.61-0.74)	0.99 (0.51-1.92)	N/A	0.09*(0.06-0.13)
Non-f2f contacts	2.47*(2.05-2.96)	2.72*(1.04-7.14)	N/A	2.32*(1.51-3.56)
F2f and Non-f2f contacts	0.99 (0.91-1.07)	1.37 (0.73-2.56)	N/A	0.38*(0.28-0.51)
Cancelled appointments or DNAs	1.23 (0.97-1.56)	1.33 (0.34-5.29)	N/A	0.48*(0.27-0.86)
Caseload	0.81*(0.78-0.84)	1.16 (0.91-1.49)	N/A	0.13*(0.12-0.14)
Liaison				
F2f contacts	0.95 (0.84-1.07)	0.72 (0.19-2.76)	0.46*(0.39-0.54)	0.60*(0.47-0.78)
Non-f2f contacts	1.94*(1.48-2.54)	17.87*(10.08-78.09)	5.26*(3.42-8.07)	1.34 (0.91-1.98)
F2f and Non-f2f contacts	1.03 (0.92-1.16)	2.31 (0.55-9.71)	0.88 (0.74-1.04)	0.77*(0.62-0.95)
Cancelled appointments or DNAs	0.69 (0.43-1.10)	0.11 (0.02-0.70)	1.48 (0.91-2.41)	1.12 (0.61-2.05)
Caseload	0.95*(0.94-0.96)	0.82*(0.76-0.89)	N/A	0.75*(0.73-0.76)
Older adults				
F2f contacts	0.51*(0.41-0.64)	0.04*(0.00-0.32)	0.27*(0.16-0.44)	0.21*(0.13-0.33)
Non-f2f contacts	2.04*(1.62-2.56)	6.60 (0.69-63.35)	5.37*(2.80-10.29)	5.70*(3.91-8.29)
F2f and Non-f2f contacts	0.95 (0.78-1.16)	0.42 (0.04-4.20)	0.95 (0.58-1.55)	1.78*(1.20-2.64)
Cancelled appointments or DNAs	1.05 (0.60-1.82)	0.23 (0.02-2.79)	0.37*(0.19-0.74)	0.97 (0.68-1.37)
Caseload	0.76*(0.73-0.79)	0.95*(0.93-0.97)	N/A	0.81*(0.79-0.81)

725 ^p<0.05; *p<0.001

726 AMH: adult mental health service; CAMHS: child and adolescent mental health service; EIP: early intervention for psychosis service; HTT:
727 home treatment team; OA: older adult service. N/A: not available data for these type of services

For peer review only

For peer review only

For peer review only - http://bmjopen.bmj.com/site/about/guidelines.xhtml

Figure legend

Figure 1. Forest plots of changes in daily deaths by site associated with lockdown and lift-of-lockdown announcements, with pooled meta-analysis estimates. Incidence Rate Ratios (IRRs) and 95% confidence intervals, percentage heterogeneity (I^2) and p-values from chi-square tests of heterogeneity are displayed. (A) Initiation of lockdown announcement vs. Before Lockdown; (B) Lift of lockdown announcement; (C) Lift of lockdown vs. pre-lockdown

For peer review only

For peer review only - http://bmjopen.bmj.com/site/about/guidelines.xhtml

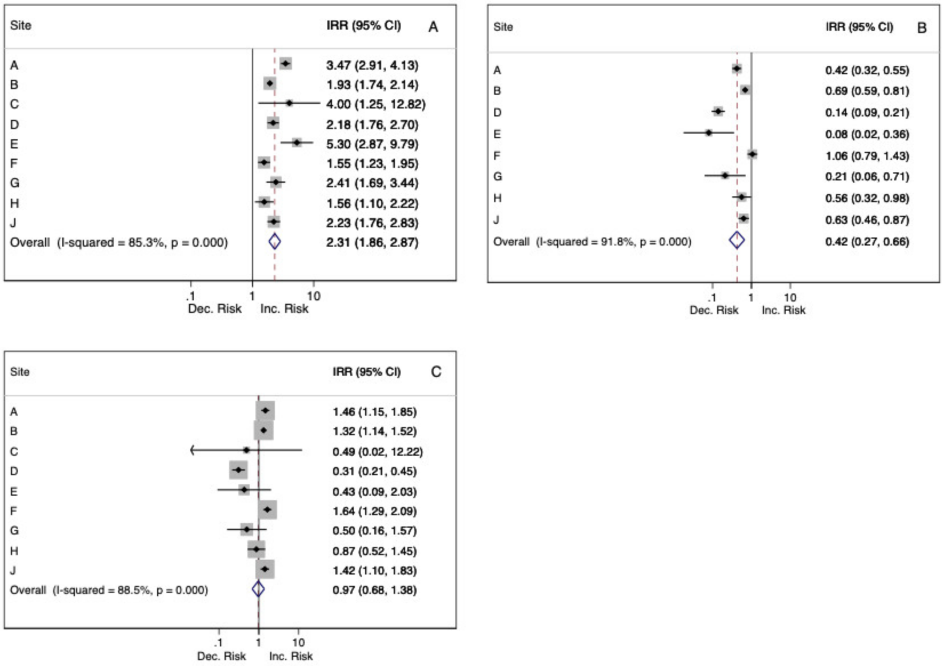

Figure 1. Forest plots of changes in daily deaths by site associated with lockdown and lift-of-lockdown announcements, with pooled meta-analysis estimates. Incidence Rate Ratios (IRRs) and 95% confidence intervals, percentage heterogeneity (I2) and p-values from chi-square tests of heterogeneity are displayed. (A) Initiation of lockdown announcement vs. Before Lockdown; (B) Lift of lockdown announcement; (C) Lift of lockdown vs. pre-lockdown

74x53mm (300 x 300 DPI)

TECHNICAL APPENDIX

Rationale for statistical analysis

Regression discontinuity is a quasi-experimental design that has been applied previously for the evaluation of nationwide policies,[1] though this approach has found little application in epidemiology to date.[2,3] Regression discontinuity in time (RDiT),[4] applied here is being employed for the analysis of time series data. In our RDiT framework, we know the date c of a policy change (here 23rd March 2020 and 10th May 2020). Thus, we assume that for all dates $t > c$, the unit is 'treated', and for all dates $t < c$, the unit is not. This RDiT set-up uses time-series data: in our case, daily counts across 10 UK sites. Two comparable time intervals (cohorts) are chosen: in our case, a period in 2019 and one in 2020. In one interval, but not the other, an event (discontinuity) occurs: in this study, the start or end of 'lockdown'. Changes at the discontinuity are measured, with the other interval acting as the control. The key feature of RDiT is the focus on comparing outcomes in a 'short' time interval before the policy implementation with a 'short' time interval after the policy implementation relative to changes observed in the previous year. We therefore assume that no 2020-specific unobserved factors confound the relationship between the exposure and the outcome by using these short time windows (and in this analysis, considered changes in national infection rates as a particular confounder).

The conditions for a valid RDiT analysis[3] were met in our study: i) the decision rule (exposed or not exposed to the intervention) and cut-off value (23rd March 2020 and 10th May 2020) were known; ii) the assignment variable (distance from the lockdown announcement, measured in days) was continuous near the cut-off value and was not affected by the policy (see Supplementary Figures 1-4); iii) there was visual confirmation of an intervention effect, in that a graphical analysis (Supplementary Figures 1-4) confirmed the discontinuity, i.e. a visible jump at the cut-off value, indicating a policy effect. We also assumed parallel trends in the time window around the lockdown and lift of lockdown announcement (a form of common/parallel-trend assumption). We further fitted different models to our time series data, with our final model being visualised in Supplementary Figure 5.

For peer review only - http://bmjopen.bmj.com/site/about/guidelines.xhtml

Further details of statistical analyses

We adopted a parametric specification for the conditional expectation functions which account for heterogeneity of temporal effects (by weekday and month). We included functions to adjust for seasonal trends before and after the cut-off points

$$Y_{i,c} = \beta_0 + \beta_1 T_{i,c} + \beta_2 Year_{i,c} + \beta_3 T_{i,c} * Year_{i,c} + \beta_4 f(D_{i,c}) + \beta_5 f(D_{i,c}) * T_{i,c} + \beta_6 f(D_{i,c}) * Year_{i,c} + \beta_7 f(D_{i,c}) * T_{i,c} * Year_{i,c} + \beta_8 W_{i,c} + \beta_9 M_{i,c} + logE_c + \varepsilon_{i,c}$$

Within each day i and site c we employed negative binomial regression to model daily counts of mortality and mental health service use activity levels defined as $Y_{i,c}$. We allowed for overdispersion and accounted for a proportional relationship between activity counts and the total daily caseload of the site by mean of an offset $\log E_c$. $T_{i,c}$ is a categorical variable whose levels were: before lockdown, during lockdown, or after lockdown (or equivalent periods in 2019). The year of the lockdown is binary variable $Year_{i,c}$ and corresponds to 2020. $D_{i,c}$ is defined as the absolute distance in days from the lockdown order announcement -$f(D_{i,c})$ is a linear function of the distance in days from the lockdown announcement interacted with the lockdown variable $T_{i,c}$ and the $Year_{i,c}$ to allow for different effects on either side of the cut-off. The model also includes $W_{i,c}$ and $M_{i,c}$ defined as set of dummy variables of weekdays and months respectively. The parameter of interest, which identifies the effect of the lockdown, is the vector β_3 which is the coefficients associated with the interaction term between our pre-post policy dummy variable $T_{i,c}$and cohort variable $Year_{i,c}$. $\beta_1, \beta_2, \beta_3, \ldots\ldots, \beta_9$ are fixed effects coefficients. Differences in daily measures before versus after the cut-off dates are reported as relative risks (RR) with their corresponding 95% confidence intervals (CI). Negative binomial regression results were pooled across sites using random effects meta-analysis and heterogeneity was summarised using the I^2 statistic.

We performed the same negative binomial modelling with minor modifications for Site I weekly data. The time resolution was changed to week and only $M_{i,c}$ was included as an additional categorical covariate. Variables T_i and D_i were defined with respect to the week numbers of the announcement (week 13) and lift (week 20) of lockdown.

For peer review only - http://bmjopen.bmj.com/site/about/guidelines.xhtml

References

1. Bakolis I, Kelly R, Fecht D, et al. Protective effects of smoke-free legislation on birth outcomes in England: a regression discontinuity design. *Epidemiology* 2016; **27**: 810-8.

2. O'Keeffe AG, Geneletti S, Baio G, Sharples LD, Nazareth I, Petersen I. Regression discontinuity designs: an approach to the evaluation of treatment efficacy in primary care using observational data. *BMJ* 2014; **349**: g5293.

3. Moscoe E, Bor J, Bärnighausen T. Regression discontinuity designs are underutilized in medicine, epidemiology, and public health: a review of current and best practice. *Journal of Clinical Epidemiology* 2015; **68**: 132-43.

4. Imbens G, Lemieux T. Regression Discontinuity Designs: A Guide to Practice. *Journal of Econometrics* 2008; **142**: 615-35.

5. DerSimonian R, Laird N. Meta-analysis in clinical trials. *Controlled Clinical Trials* 1986; **7**: 177-88.

For peer review only

Supplementary Table 1: Site-specific data for lockdown initiation changes in daily mortality and service activity

	IRR (95%CI) for lockdown vs. pre-lockdown comparisons								
**Measures**	**Site A**	**Site B**	**Site C**	**Site D**	**Site E**	**Site F**	**Site G**	**Site H**	**Site J**
Number of deaths	3.47(2.92-4.14)	1.93(1.74-2.13)	4.00(1.25-12.85)	2.18(1.76-2.70)	5.30(2.87-9.79)	1.55(1.23-1.94)	2.41(1.69-3.44)	1.56(1.10-2.22)	2.23(1.76-2.84)
Trust-wide activity									
Number of new referrals accepted	0.64(0.46-0.90)	0.65(0.45-0.93)	N/A	0.62(0.46-0.83)	N/A	0.62(0.52-0.75)	N/A	0.62(0.43-0.89)	0.60(0.42-0.87)
Number of discharges	0.96(0.78-1.19)	1.08(0.75-1.56)	N/A	1.00(0.71-1.41)	N/A	0.89(0.73-1.09)	N/A	0.74(0.46-1.18)	1.05(0.78-1.41)
Inpatient care									
New admissions	0.81(0.67-0.99)	0.77(0.57-1.03)	0.59(0.45-0.77)	0.79(0.60-1.04)	0.72(0.47-1.11)	0.76(0.53-1.09)	N/A	0.73(0.53-0.99)	0.77(0.55-1.06)
Discharges	1.25(0.92-1.69)	1.06(0.68-1.63)	1.02(0.66-1.59)	1.24(0.75-2.06)	0.99(0.57-1.72)	1.10(0.72-1.69)	N/A	1.48(0.91-2.40)	0.92(0.62-1.39)
Inpatient caseload	0.77(0.75-0.79)	0.82(0.81-0.84)	0.91(0.90-0.92)	0.97(0.96-0.98)	0.79(0.77-0.80)	0.67(0.65-0.69)	N/A	0.89(0.87-0.90)	1.00(0.99-1.01)
Inpatient caseload (MHA)	0.84(0.82-0.86)	0.93(0.92-0.94)	N/A	1.00(0.99-1.02)	N/A	0.78(0.76-0.80)	N/A	0.99(0.97-1.00)	1.05(1.04-1.06)
AMH (community)									
F2f contacts	0.54(0.41-0.70)	0.44(0.27-0.71)	N/A	1.20(0.70-2.06)	0.72(0.42-1.23)	0.44(0.27-0.73)	0.37(0.14-0.97)	0.57(0.40-0.82)	0.41(0.29-0.59)
Non-f2f contacts	2.13(1.71-2.66)	3.16(2.11-4.74)	N/A	3.14(1.80-5.47)	3.04(1.75-5.31)	8.38(4.97-14.13)	8.22(3.65-18.56)	2.24(1.41-3.54)	5.32(3.74-7.55)
F2f and Non-f2f contacts	1.03(0.85-1.24)	1.21(0.77-1.89)	N/A	1.38(0.81-2.36)	1.11(0.65-1.89)	0.86(0.52-1.43)	0.51(0.20-1.30)	1.19(0.80-1.77)	1.15(0.83-1.61)
Cancelled/DNA	0.52(0.41-0.66)	1.24(0.76-2.02)	N/A	1.28(0.75-2.19)	0.45(0.25-0.81)	1.17(0.63-2.18)	0.54(0.22-1.38)	1.13(0.69-1.85)	0.93(0.62-1.41)
Caseload	1.02(1.00-1.04)	1.03(1.03-1.04)	N/A	1.02(1.00-1.04)	1.01(1.00-1.02)	1.02(1.01-1.03)	N/A	1.02(1.02-1.03)	1.06(1.05-1.07)
CAMHS									
F2f contacts	0.12(0.08-0.17)	N/A	N/A	1.01(0.62-1.63)	0.12(0.04-0.36)	0.99(0.59-1.65)	0.82(0.39-1.71)	0.32(0.20-0.51)	0.15(0.10-0.23)
Non-f2f contacts	2.53(2.03-3.15)	N/A	N/A	5.14(3.12-8.48)	30.64(3.9-67.51)	2.73(1.65-4.52)	8.87(4.10-19.15)	2.42(1.46-4.00)	3.02(2.05-4.47)
F2f and Non-f2f contacts	0.89(0.71-1.12)	N/A	N/A	1.41(0.87-2.29)	1.43(0.71-2.91)	1.15(0.69-1.93)	1.14(0.58-2.24)	1.25(0.77-2.04)	1.28(0.87-1.86)
Cancelled/DNA	0.83(0.66-1.04)	N/A	N/A	1.36(0.84-2.21)	0.62(0.13-2.98)	0.56(0.35-0.89)	2.91(0.95-8.96)	0.78(0.46-1.31)	0.80(0.54-1.20)
Caseload	1.02(1.00-1.04)	N/A	N/A	1.02(1.00-1.04)	0.83(0.82-0.85)	0.97(0.96-0.99)	N/A	0.94(0.93-0.95)	0.99(0.98-0.99)
EIP									
F2f contacts	0.19(0.15-0.26)	0.20(0.12-0.31)	N/A	1.06(0.61-1.87)	0.38(0.21-0.68)	0.16(0.09-0.30)	N/A	0.34(0.22-0.53)	0.16(0.12-0.23)
Non-f2f contacts	2.35(1.78-3.10)	3.71(2.49-5.51)	N/A	2.61(1.46-4.68)	7.95(4.33-14.60)	18.15(4.96-66.42)	N/A	2.17(1.50-3.14)	6.48(4.23-9.94)
F2f and Non-f2f contacts	0.97(0.76-1.23)	1.11(0.74-1.64)	N/A	1.34(0.77-2.34)	1.60(0.92-2.79)	0.47(0.26-0.84)	N/A	1.17(0.81-1.71)	1.24(0.97-1.58)
Cancelled/DNA	0.52(0.40-0.67)	1.30(0.80-2.13)	N/A	1.16(0.66-2.03)	0.38(0.19-0.78)	0.49(0.27-0.89)	N/A	1.10(0.72-1.70)	0.84(0.54-1.32)
Caseload	1.04(1.02-1.05)	1.01(1.00-1.02)	N/A	1.02(1.01-1.03)	1.01(1.00-1.02)	1.06(1.05-1.07)	N/A	1.04(1.03-1.05)	1.09(1.08-1.10)
HTT									
F2f contacts	0.47(0.44-0.51)	0.30(0.26-0.35)	N/A	0.75(0.69-0.83)	0.67(0.58-0.78)	0.48(0.41-0.57)	N/A	0.46(0.39-0.54)	0.15(0.11-0.20)
Non-f2f contacts	1.95(1.63-2.34)	2.06(1.80-2.35)	N/A	1.42(1.07-1.89)	2.79(1.73-4.50)	1.43(1.06-1.94)	N/A	1.64(1.45-1.86)	1.67(1.16-2.42)
F2f and Non-f2f contacts	0.76(0.71-0.81)	0.94(0.86-1.04)	N/A	0.83(0.77-0.90)	0.83(0.73-0.95)	0.65(0.56-0.76)	N/A	1.08(0.94-1.23)	0.38(0.28-0.52)
Cancelled/DNA	1.00(0.81-1.24)	0.97(0.78-1.22)	N/A	0.77(0.59-1.00)	0.48(0.33-0.70)	0.48(0.33-0.70)	N/A	0.94(0.76-1.16)	0.29(0.18-0.46)
Caseload	0.74(0.72-0.76)	0.56(0.53-0.59)	N/A	0.58(0.55-0.61)	0.42(0.39-0.45)	0.70(0.65-0.75)	N/A	0.98(0.97-0.99)	0.49(0.45-0.53)
Liaison									
F2f contacts	0.51(0.46-0.57)	0.34(0.24-0.49)	N/A	0.60(0.49-0.74)	0.36(0.27-0.47)	0.49(0.34-0.70)	0.38(0.28-0.51)	0.48(0.42-0.55)	0.29(0.23-0.37)
Non-f2f contacts	1.47(1.13-1.93)	1.69(0.95-2.99)	N/A	0.85(0.65-1.11)	1.22(0.51-2.89)	1.83(0.91-3.69)	3.37(1.77-6.42)	1.47(1.08-1.99)	1.63(1.16-2.29)
F2f and Non-f2f contacts	0.61(0.54-0.68)	0.53(0.37-0.75)	N/A	0.65(0.53-0.80)	0.39(0.29-0.51)	0.66(0.46-0.94)	0.74(0.56-0.98)	0.61(0.54-0.69)	0.59(0.49-0.70)
Cancelled/DNA	0.31(0.21-0.47)	0.41(0.13-1.33)	N/A	1.23(0.76-1.99)	0.15(0.02-1.35)	0.85(0.49-1.50)	2.05(0.80-5.24)	0.21(0.11-0.39)	1.04(0.67-1.63)
Caseload	0.91(0.90-0.92)	0.35(0.31-0.39)	N/A	0.92(0.91-0.93)	0.45(0.42-0.50)	0.82(0.80-0.84)	N/A	1.03(1.00-1.06)	0.92(0.90-0.93)
OA									
F2f contacts	0.28(0.20-0.38)	0.18(0.10-0.31)	N/A	1.21(0.70-2.11)	0.58(0.33-1.03)	0.19(0.11-0.33)	0.22(0.11-0.45)	0.40(0.24-0.65)	0.18(0.12-0.28)
Non-f2f contacts	1.84(1.44-2.36)	4.45(2.54-7.79)	N/A	3.22(1.86-5.57)	9.68(4.70-19.93)	3.78(2.27-6.31)	7.98(3.03-20.99)	2.40(1.41-4.09)	3.43(2.51-4.69)

For peer review only

F2f and Non-f2f contacts	0.74(0.59-0.94)	1.34(0.79-2.29)	N/A	1.77(1.02-3.08)	1.51(0.85-2.66)	0.63(0.38-1.03)	0.80(0.41-1.55)	1.17(0.71-1.92)	1.20(0.85-1.70)
Cancelled/DNA	0.60(0.37-0.99)	1.56(0.90-2.71)	N/A	1.52(0.87-2.65)	0.65(0.24-1.77)	1.85(1.17-2.93)	0.41(0.15-1.16)	0.73(0.47-1.14)	1.77(1.38-2.27)
Caseload	0.91(0.91-0.92)	1.00(1.00-1.00)	N/A	1.00(0.99-1.02)	0.92(0.91-0.93)	0.96(0.94-0.98)	N/A	0.94(0.93-0.95)	0.98(0.97-0.98)

AMH: adult mental health service; CAMHS: child and adolescent mental health service; EIP: early intervention for psychosis service; HTT: home treatment team; OA: older adult service. N/A: data not available for these type of services

For peer review only

For peer review only - http://bmjopen.bmj.com/site/about/guidelines.xhtml

Supplementary Table 2: Site-specific data for lift-of-lockdown changes in daily mortality and service activity

	IRR (95%CI) for lift of lockdown vs. lockdown comparisons								
**Measures**	**Site A**	**Site B**	**Site C**	**Site D**	**Site E**	**Site F**	**Site G**	**Site H**	**Site J**
Number of deaths	0.42(0.32-0.55)	0.69(0.59-0.81)	0.12(0.05-3.56)	0.14(0.10-0.22)	0.08(0.02-0.40)	1.06(0.79-1.43)	0.21(0.06-0.68)	0.56(0.32-0.98)	0.63(0.46-0.87)
Trust-wide activity									
Number of new referrals accepted	1.32(0.92-1.89)	1.55(0.90-2.65)	N/A	1.39(0.88-2.18)	N/A	1.33(1.02-1.74)	N/A	1.49(0.85-2.61)	1.21(0.70-2.08)
Number of discharges	0.90(0.71-1.15)	1.10(0.62-1.95)	N/A	0.97(0.58-1.60)	N/A	0.97(0.71-1.32)	N/A	1.40(0.66-2.96)	0.83(0.50-1.40)
Inpatient care									
New admissions	1.08(0.79-1.47)	1.47(0.95-2.27)	1.32(0.87-2.00)	1.27(0.84-1.91)	1.17(0.63-2.15)	0.87(0.48-1.58)	N/A	1.34(0.88-2.04)	1.18(0.66-2.12)
Discharges	0.51(0.34-0.76)	1.04(0.55-1.97)	0.90(0.48-1.72)	0.69(0.32-1.51)	0.44(0.16-1.20)	0.44(0.20-0.99)	N/A	0.60(0.29-1.23)	0.96(0.50-1.85)
Inpatient caseload	1.01(0.99-1.04)	1.14(1.13-1.16)	1.04(1.03-1.06)	0.94(0.93-0.95)	1.11(1.08-1.14)	1.04(1.00-1.07)	N/A	1.14(1.12-1.16)	1.08(1.07-1.09)
Inpatient caseload (MHA)	1.04(1.02-1.07)	1.08(1.07-1.10)	N/A	0.92(0.92-0.93)	N/A	1.07(1.03-1.11)	N/A	0.87(0.84-0.89)	1.09(1.08-1.10)
AMH (community)									
F2f contacts	0.82(0.59-1.15)	1.31(0.62-2.76)	N/A	1.23(0.52-2.91)	1.02(0.43-2.43)	0.91(0.35-2.40)	1.17(0.12-11.37)	1.16(0.67-2.00)	1.13(0.65-1.96)
Non-f2f contacts	1.35(1.07-1.70)	1.33(0.72-2.46)	N/A	1.30(0.53-3.22)	0.72(0.30-1.71)	0.62(0.25-1.49)	2.29(0.20-26.05)	1.15(0.56-2.38)	1.46(0.87-2.45)
F2f and Non-f2f contacts	1.10(0.88-1.38)	1.29(0.64-2.59)	N/A	1.23(0.52-2.91)	0.92(0.40-2.16)	0.78(0.31-1.97)	2.61(0.23-29.41)	1.14(0.61-2.14)	1.32(0.78-2.25)
Cancelled/DNA	1.24(0.95-1.62)	1.02(0.47-2.21)	N/A	0.96(0.41-2.26)	0.44(0.16-1.22)	0.49(0.21-1.16)	1.91(0.20-17.82)	1.17(0.56-2.46)	0.95(0.49-1.86)
Caseload	1.01(1.01-1.02)	1.01(1.00-1.01)	N/A	1.00(1.00-1.01)	1.01(1.01-1.01)	1.03(1.02-1.03)	N/A	1.01(1.00-1.01)	1.01(1.01-1.01)
CAMHS									
F2f contacts	1.29(0.60-2.80)	N/A	N/A	1.16(0.54-2.48)	2.25(0.56-9.08)	1.59(0.66-3.81)	0.30(0.04-2.12)	1.41(0.71-2.80)	0.58(0.28-1.19)
Non-f2f contacts	1.32(1.03-1.70)	N/A	N/A	1.04(0.46-2.36)	0.52(0.04-6.70)	0.80(0.40-1.60)	2.58(0.43-15.31)	1.38(0.63-3.03)	1.09(0.59-2.01)
F2f and Non-f2f contacts	1.25(0.93-1.67)	N/A	N/A	1.14(0.53-2.46)	1.33(0.48-3.68)	1.43(0.61-3.32)	0.47(0.07-2.98)	1.41(0.68-2.92)	0.93(0.53-1.66)
Cancelled/DNA	1.12(0.87-1.46)	N/A	N/A	0.73(0.33-1.63)	N/A	1.42(0.65-3.13)	0.07(0.01-0.64)	0.99(0.45-2.15)	0.85(0.44-1.65)
Caseload	0.99(0.99-0.99)	N/A	N/A	1.00(0.99-1.00)	1.11(1.09-1.12)	0.96(0.96-0.96)	N/A	0.98(0.97-0.98)	0.99(0.99-0.99)
EIP									
F2f contacts	1.16(0.85-1.60)	2.56(1.35-4.84)	N/A	1.11(0.47-2.65)	1.36(0.56-3.30)	3.92(1.77-8.72)	N/A	1.37(0.69-2.72)	1.70(1.05-2.76)
Non-f2f contacts	1.67(1.23-2.27)	1.30(0.68-2.48)	N/A	1.08(0.45-2.61)	0.54(0.22-1.34)	1.72(0.17-16.98)	N/A	1.41(0.80-2.49)	3.05(1.80-5.16)
F2f and Non-f2f contacts	1.37(1.04-1.81)	1.61(0.87-2.95)	N/A	1.09(0.46-2.57)	0.79(0.34-1.87)	2.02(0.92-4.41)	N/A	1.29(0.71-2.37)	1.64(1.22-2.22)
Cancelled/DNA	1.16(0.88-1.54)	0.74(0.36-1.55)	N/A	1.03(0.44-2.45)	0.24(0.09-0.65)	1.34(0.53-3.36)	N/A	1.27(0.67-2.39)	1.20(0.72-2.01)
Caseload	0.99(0.99-1.00)	1.06(1.04-1.07)	N/A	1.01(1.00-1.01)	1.00(1.00-1.00)	1.04(1.03-1.05)	N/A	1.10(1.10-1.11)	1.05(1.04-1.06)
HTT									
F2f contacts	1.55(1.37-1.75)	1.90(1.56-2.31)	N/A	1.40(1.21-1.61)	1.38(1.16-1.65)	1.38(1.13-1.67)	N/A	1.47(1.18-1.83)	1.15(0.73-1.81)
Non-f2f contacts	1.21(0.93-1.57)	1.11(0.93-1.34)	N/A	1.57(1.10-2.23)	0.61(0.33-1.13)	0.82(0.57-1.20)	N/A	0.86(0.72-1.02)	1.85(1.03-3.31)
F2f and Non-f2f contacts	1.37(1.24-1.52)	1.36(1.18-1.56)	N/A	1.39(1.22-1.59)	1.21(1.03-1.43)	1.19(0.97-1.46)	N/A	0.98(0.81-1.18)	1.42(0.92-2.17)
Cancelled/DNA	1.09(0.78-1.52)	1.34(0.97-1.85)	N/A	1.88(1.30-2.72)	1.49(0.76-2.92)	3.00(1.72-5.21)	N/A	0.93(0.67-1.27)	1.91(0.87-4.23)
Caseload	1.22(1.18-1.27)	1.26(1.17-1.36)	N/A	1.23(1.16-1.31)	1.35(1.24-1.46)	1.25(1.14-1.36)	N/A	0.93(0.91-0.94)	0.35(0.33-0.38)
Liaison									
F2f contacts	1.81(1.52-2.14)	2.11(1.22-3.64)	N/A	1.48(1.06-2.05)	1.58(1.04-2.42)	1.50(0.88-2.56)	0.98(0.53-1.82)	1.40(1.17-1.67)	2.67(1.93-3.67)
Non-f2f contacts	1.31(0.91-1.89)	0.59(0.25-1.36)	N/A	1.12(0.74-1.69)	2.62(0.79-8.69)	6.11(1.58-23.63)	1.09(0.35-3.40)	0.74(0.48-1.14)	0.93(0.57-1.52)
F2f and Non-f2f contacts	1.68(1.43-1.97)	1.51(0.88-2.60)	N/A	1.37(0.98-1.92)	1.57(1.02-2.43)	1.80(1.02-3.18)	0.87(0.45-1.67)	1.24(1.04-1.47)	1.55(1.21-1.99)
Cancelled/DNA	1.39(0.70-2.76)	1.04(0.10-10.5)	N/A	0.92(0.46-1.82)	6.97(0.41-117.19)	0.44(0.19-1.02)	0.13(0.01-1.21)	0.12(0.02-0.89)	0.83(0.51-1.35)
Caseload	1.10(1.08-1.12)	1.19(1.02-1.39)	N/A	0.99(0.98-1.01)	1.90(1.71-2.11)	1.16(1.12-1.20)	N/A	1.04(1.01-1.06)	1.00(0.98-1.01)
OA									
F2f contacts	1.98(1.37-2.86)	1.09(0.46-2.61)	N/A	1.65(0.69-3.96)	1.18(0.45-3.12)	0.76(0.34-1.72)	0.68(0.12-3.67)	1.20(0.53-2.71)	1.25(0.65-2.40)
Non-f2f contacts	1.20(0.87-1.66)	1.34(0.58-3.10)	N/A	0.91(0.38-2.21)	1.23(0.39-3.92)	1.34(0.65-2.78)	0.45(0.05-3.91)	1.37(0.61-3.08)	1.65(0.99-2.75)
F2f and Non-f2f contacts	1.46(1.07-1.99)	1.20(0.52-2.77)	N/A	1.28(0.53-3.06)	1.15(0.46-2.89)	0.88(0.41-1.87)	0.65(0.12-3.46)	1.16(0.53-2.56)	1.47(0.85-2.53)

For peer review only - http://bmjopen.bmj.com/site/about/guidelines.xhtml

Cancelled/DNA	2.15(1.10-4.17)	0.70(0.29-1.70)	N/A	0.82(0.34-2.01)	3.36(0.49-23.00)	0.15(0.07-0.34)	0.41(0.04-4.20)	0.64(0.29-1.43)	0.58(0.37-0.91)
Caseload	0.91(0.90-0.92)	0.99(0.98-0.99)	N/A	0.95(0.95-0.96)	1.11(1.09-1.13)	0.99(0.97-1.01)	N/A	0.99(0.98-1.00)	0.98(0.98-0.99)

AMH: adult mental health service; CAMHS: child and adolescent mental health service; EIP: early intervention for psychosis service; HTT: home treatment team; OA: older adult service. N/A: data not available for these type of services

For peer review only

Supplementary Table 3: Site-specific data for lift-of-lockdown vs. pre-lockdown changes in daily mortality and service activity

	IRR (95%CI) for lift of lockdown vs. pre-lockdown comparisons								
**Measures**	**Site A**	**Site B**	**Site C**	**Site D**	**Site E**	**Site F**	**Site G**	**Site H**	**Site J**
Number of deaths	1.46(1.15-1.85)	1.32(1.15-1.53)	0.49(0.02-12.44)	0.31(0.22-0.46)	0.43(0.09-2.00)	1.64(1.29-2.09)	0.50(0.16-1.58)	0.87(0.52-1.45)	1.42(1.10-1.82)
Trust-wide activity									
Number of new referrals accepted	0.85(0.72-0.99)	1.00(0.63-1.58)	N/A	0.85(0.58-1.26)	N/A	0.83(0.67-1.04)	N/A	0.92(0.57-1.49)	0.73(0.46-1.15)
Number of discharges	0.87(0.75-1.01)	1.19(0.73-1.96)	N/A	0.96(0.63-1.47)	N/A	0.86(0.67-1.11)	N/A	1.03(0.54-1.98)	0.88(0.54-1.41)
Inpatient care									
New admissions	0.88(0.66-1.15)	1.13(0.78-1.63)	0.76(0.53-1.09)	1.00(0.71-1.42)	0.84(0.52-1.36)	0.66(0.39-1.12)	N/A	0.97(0.68-1.40)	0.90(0.53-1.53)
Discharges	0.64(0.47-0.86)	1.10(0.63-1.89)	0.83(0.51-1.35)	0.86(0.43-1.69)	0.44(0.18-1.08)	0.49(0.23-1.03)	N/A	0.89(0.48-1.63)	0.89(0.50-1.56)
Inpatient caseload	0.78(0.77-0.79)	0.94(0.93-0.95)	0.95(0.94-0.96)	0.91(0.90-0.92)	0.87(0.85-0.89)	0.69(0.67-0.71)	N/A	1.01(1.00-1.02)	1.08(1.07-1.09)
Inpatient caseload (MHA)	0.88(0.87-0.89)	1.01(1.00-1.02)	N/A	0.93(0.92-0.93)	N/A	0.83(0.81-0.85)	N/A	0.86(0.84-0.88)	1.14(1.13-1.15)
AMH (community)									
F2f contacts	0.44(0.34-0.56)	0.57(0.30-1.09)	N/A	1.48(0.70-3.14)	0.73(0.34-1.55)	0.40(0.17-0.97)	0.43(0.05-3.58)	0.67(0.41-1.08)	0.46(0.29-0.76)
Non-f2f contacts	2.88(2.43-3.41)	4.20(2.46-7.17)	N/A	4.08(1.84-9.05)	2.18(1.02-4.64)	5.17(2.29-11.67)	18.87(1.87-90.10)	2.57(1.37-4.84)	7.79(5.04-12.04)
F2f and Non-f2f contacts	1.13(0.96-1.33)	1.56(0.85-2.85)	N/A	1.70(0.80-3.61)	1.02(0.49-2.14)	0.67(0.29-1.55)	1.33(0.14-12.90)	1.36(0.78-2.35)	1.53(0.95-2.44)
Cancelled/DNA	0.65(0.56-0.75)	1.26(0.65-2.46)	N/A	1.23(0.59-2.60)	0.20(0.08-0.50)	0.58(0.29-1.14)	1.04(0.13-8.21)	1.32(0.70-2.50)	0.89(0.50-1.59)
Caseload	1.04(1.03-1.04)	1.04(1.04-1.04)	N/A	1.02(1.02-1.02)	1.01(1.01-1.01)	1.04(1.04-1.05)	N/A	1.03(1.03-1.03)	1.07(1.07-1.07)
CAMHS									
F2f contacts	0.15(0.07-0.32)	N/A	N/A	1.16(0.60-2.26)	0.27(0.09-0.77)	1.57(0.72-3.40)	0.24(0.04-1.55)	0.45(0.25-0.82)	0.09(0.05-0.16)
Non-f2f contacts	3.35(2.76-4.06)	N/A	N/A	5.36(2.62-10.99)	15.89(2.94-85.84)	2.19(1.21-3.96)	22.85(4.40-81.80)	3.33(1.69-6.56)	3.30(1.93-5.65)
F2f and Non-f2f contacts	1.11(0.89-1.38)	N/A	N/A	1.61(0.82-3.15)	1.91(0.79-4.59)	1.64(0.79-3.43)	0.53(0.09-3.06)	1.77(0.95-3.27)	1.19(0.72-1.96)
Cancelled/DNA	0.93(0.78-1.11)	N/A	N/A	1.00(0.49-2.02)	N/A	0.79(0.39-1.60)	0.22(0.03-1.40)	0.77(0.40-1.48)	0.69(0.38-1.23)
Caseload	1.01(1.01-1.01)	N/A	N/A	1.02(1.01-1.02)	0.92(0.91-0.94)	0.93(0.92-0.94)	N/A	0.91(0.90-0.92)	0.97(0.97-0.97)
EIP									
F2f contacts	0.23(0.19-0.28)	0.50(0.30-0.84)	N/A	1.19(0.57-2.49)	0.52(0.24-1.09)	0.63(0.34-1.15)	N/A	0.47(0.26-0.84)	0.28(0.18-0.42)
Non-f2f contacts	3.91(3.18-4.82)	4.83(2.69-8.67)	N/A	2.82(1.29-6.16)	4.33(2.00-9.38)	31.28(3.75-60.97)	N/A	3.07(1.88-5.00)	19.75(12.4-31.2)
F2f and Non-f2f contacts	1.33(1.12-1.58)	1.78(1.04-3.02)	N/A	1.46(0.70-3.06)	1.27(0.61-2.64)	0.95(0.51-1.76)	N/A	1.52(0.90-2.58)	2.03(1.64-2.53)
Cancelled/DNA	0.60(0.51-0.71)	0.97(0.52-1.81)	N/A	1.20(0.57-2.51)	0.09(0.04-0.21)	0.66(0.30-1.41)	N/A	1.40(0.82-2.40)	1.02(0.71-1.44)
Caseload	1.04(1.03-1.04)	1.07(1.06-1.08)	N/A	1.03(1.02-1.03)	1.01(1.00-1.01)	1.10(1.09-1.11)	N/A	1.15(1.14-1.15)	1.15(1.14-1.15)
HTT									
F2f contacts	0.73(0.66-0.82)	0.57(0.49-0.66)	N/A	1.05(0.93-1.19)	0.93(0.82-1.06)	0.67(0.58-0.77)	N/A	0.68(0.56-0.82)	0.17(0.11-0.25)
Non-f2f contacts	2.36(1.87-2.97)	2.29(1.95-2.70)	N/A	2.23(1.64-3.02)	1.69(1.03-2.80)	1.18(0.88-1.59)	N/A	1.41(1.22-1.63)	3.09(1.81-5.28)
F2f and Non-f2f contacts	1.04(0.96-1.14)	1.28(1.14-1.44)	N/A	1.16(1.03-1.30)	1.01(0.88-1.15)	0.78(0.67-0.91)	N/A	1.06(0.91-1.23)	0.55(0.39-0.77)
Cancelled/DNA	1.09(0.81-1.46)	1.31(1.00-1.71)	N/A	1.44(1.07-1.94)	0.72(0.39-1.31)	1.44(0.90-2.32)	N/A	0.87(0.65-1.15)	0.55(0.28-1.10)
Caseload	0.91(0.88-0.94)	0.71(0.67-0.75)	N/A	0.71(0.68-0.75)	0.56(0.52-0.60)	0.87(0.80-0.94)	N/A	0.91(0.89-0.92)	0.17(0.16-0.18)
Liaison									
F2f contacts	0.93(0.80-1.08)	0.72(0.45-1.15)	N/A	0.89(0.67-1.18)	0.56(0.39-0.81)	0.73(0.47-1.14)	0.37(0.21-0.64)	0.67(0.59-0.77)	0.78(0.60-1.01)
Non-f2f contacts	1.93(1.41-2.65)	0.99(0.47-2.06)	N/A	0.95(0.67-1.35)	3.18(1.11-9.11)	11.20(3.05-41.19)	3.67(1.36-9.94)	1.09(0.76-1.56)	1.51(0.97-2.37)
F2f and Non-f2f contacts	1.02(0.89-1.16)	0.80(0.50-1.28)	N/A	0.89(0.67-1.19)	0.61(0.42-0.88)	1.18(0.72-1.93)	0.64(0.35-1.18)	0.75(0.65-0.86)	0.91(0.74-1.14)
Cancelled/DNA	0.44(0.24-0.81)	0.42(0.05-3.45)	N/A	1.13(0.65-1.98)	1.06(0.15-7.26)	0.38(0.19-0.76)	0.26(0.03-2.06)	0.02(0.00-0.19)	0.87(0.56-1.33)
Caseload	1.00(0.98-1.02)	0.41(0.36-0.47)	N/A	0.91(0.90-0.92)	0.86(0.79-0.94)	0.95(0.92-0.99)	N/A	1.06(1.03-1.10)	0.91(0.90-0.92)
OA									
F2f contacts	0.55(0.44-0.70)	0.20(0.09-0.42)	N/A	2.00(0.94-4.26)	0.69(0.29-1.63)	0.15(0.07-0.29)	0.15(0.03-0.72)	0.48(0.23-0.98)	0.23(0.13-0.40)
Non-f2f contacts	2.21(1.73-2.83)	5.98(2.9-12.2)	N/A	2.94(1.36-6.38)	11.92(4.31-32.98)	5.08(2.75-9.39)	3.56(0.49-26.01)	3.28(1.64-6.58)	5.66(3.58-8.95)
F2f and Non-f2f contacts	1.09(0.87-1.36)	1.61(0.78-3.33)	N/A	2.26(1.06-4.84)	1.73(0.77-3.90)	0.55(0.29-1.05)	0.52(0.11-2.47)	1.36(0.68-2.70)	1.76(1.10-2.82)

Cancelled/DNA	1.30(0.76-2.20)	1.09(0.50-2.37)	N/A	1.25(0.57-2.73)	2.19(0.39-12.32)	0.28(0.13-0.58)	0.17(0.02-1.41)	0.47(0.23-0.97)	1.03(0.67-1.57)
Caseload	0.83(0.83-0.84)	0.99(0.99-0.99)	N/A	0.95(0.95-0.95)	1.02(1.01-1.03)	0.95(0.94-0.96)	N/A	0.93(0.92-0.94)	0.96(0.96-0.96)

AMH: adult mental health service; CAMHS: child and adolescent mental health service; EIP: early intervention for psychosis service; HTT: home treatment team; OA: older adult service. N/A: data not available for these type of services

For peer review only

For peer review only - http://bmjopen.bmj.com/site/about/guidelines.xhtml

Supplementary Table 4. Meta-analysed effects of lockdown announcements on mortality and mental health service use activity omitting one week around the two transition dates (23rd March and 10th May).

	Lockdown Announcement vs Pre-lockdown		Lift of Lockdown Announcement vs Lockdown		Lift of Lockdown vs. Pre-lockdown	
**Measures**	IRR (95%CI)	I^2	IRR (95%CI)	I^2	IRR (95%CI)	I^2
Number of deaths	2.40*(2.02-2.84)	67.5	0.33*(0.18-0.60)	72.7	0.79 (0.46-1.33)	63.8
Trust-wide activity						
Number of new referrals accepted	0.69*(0.60-0.79)	0.0	1.68*(1.21-2.35)	0.0	1.09 (0.81-1.47)	0.0
Number of discharges	0.86*(0.77-0.97)	0.0	1.34 (0.91-1.98)	0.0	1.23 (0.86-1.76)	0.0
Inpatient care						
New admissions	0.82^(0.68-0.98)	49.8	1.62*(1.21-2.17)	0.0	1.14 (0.80-1.63)	39.3
Discharges	0.96 (0.80-1.16)	0.0	1.23 (0.69-2.18)	26.5	1.25 (0.78-1.99)	13.4
Inpatient caseload	0.84*(0.76-0.92)	99.8	1.13*(1.06-1.21)	98.3	0.95 (0.89-1.01)	99.2
Inpatient caseload on MHA section	0.96 (0.89-1.03)	99.5	0.99 (0.90-1.10)	98.8	0.95 (0.85-1.07)	99.3
AMH (community)						
F2f contacts	0.55*(0.44-0.69)	14.8	1.97*(1.15-3.37)	0.0	1.19 (0.73-1.95)	0.0
Non-f2f contacts	4.54*(2.84-7.25)	85.7	1.52 (0.94-2.45)	0.0	5.35*(3.43-8.35)	0.0
F2f and Non-f2f contacts	1.23*(1.06-1.44)	0.0	1.90*(1.16-3.10)	0.0	2.37*(1.50-3.75)	0.0
Cancelled appointments or DNAs	0.74 (0.54-1.02)	59.3	1.85*(1.05-3.26)	0.0	1.57 (0.92-2.66)	0.0
Caseload	1.05*(1.03-1.07)	98.6	1.01 (1.00-1.02)	89.4	1.04^(1.02-1.06)	98.3
CAMHS						
F2f contacts	0.38*(0.16-0.91)	90.9	1.94 (0.95-3.97)	0.0	0.86 (0.45-1.64)	0.0
Non-f2f contacts	4.67*(3.34-6.53)	57.5	0.98 (0.48-1.99)	21.1	4.87*(1.99-11.89)	52.5
F2f and Non-f2f contacts	1.46*(1.22-1.75)	0.0	1.71 (0.95-3.07)	0.0	2.55*(1.48-4.41)	0.0
Cancelled appointments or DNAs	0.80 (0.64-1.01)	17.8	1.29 (0.67-2.46)	0.0	1.03 (0.57-1.88)	0.0
Caseload	0.92*(0.89-0.96)	98.8	0.98 (0.96-1.00)	98.3	0.94^(0.89-0.99)	99.8
EIP						
F2f contacts	0.31*(0.21-0.45)	71.9	1.49 (0.85-2.61)	3.5	0.47 (0.21-1.04)	57.5
Non-f2f contacts	5.61*(3.08-10.22)	88.7	7.44 (0.63-48.53)	95.8	4.03*(3.58-14.05)	96.5
F2f and Non-f2f contacts	1.35*(1.17-1.56)	0.0	1.24 (0.78-1.98)	0.0	1.69*(1.10-2.61)	0.0
Cancelled appointments or DNAs	0.83 (0.58-1.20)	66.0	1.44 (0.73-2.85)	21.0	1.18 (0.48-2.92)	60.1
Caseload	1.06^(1.01-1.11)	99.8	1.03 (1.00-1.06)	99.3	1.09*(1.04-1.13)	99.6
HTT						
F2f contacts	0.45*(0.31-0.65)	97.1	1.49*(1.24-1.80)	39.0	0.76 (0.57-1.01)	76.3
Non-f2f contacts	1.90*(1.62-2.23)	58.8	1.05 (0.74-1.49)	63.1	2.02*(1.52-2.68)	54.9
F2f and Non-f2f contacts	0.88*(0.80-0.96)	66.1	1.27*(1.11-1.46)	26.8	1.14 (0.96-1.34)	52.8
Cancelled appointments or DNAs	0.71*(0.52-0.97)	82.2	1.27 (0.91-1.79)	29.5	1.05 (0.67-1.63)	64.2
Caseload	0.53*(0.36-0.79)	99.9	1.18(0.94-1.49)	98.9	0.63*(0.50-0.79)	99.4
Liaison						
F2f contacts	0.46*(0.36-0.60)	84.9	1.70*(1.34-2.17)	20.5	0.80*(0.67-0.94)	0.0
Non-f2f contacts	1.67*(1.15-2.41)	75.7	1.32 (0.90-1.93)	2.7	1.90*(1.19-3.04)	37.2
F2f and Non-f2f contacts	0.63*(0.52-0.76)	75.6	1.49*(1.10-2.01)	46.4	0.88 (0.74-1.05)	0.0
Cancelled appointments or DNAs	0.74 (0.45-1.22)	67.6	1.26 (0.51-3.12)	0.0	1.11 (0.42-2.88)	13.6
Caseload	0.77*(0.72-0.82)	98.7	1.19*(1.09-1.30)	95.9	0.87 (0.78-0.97)	97.8
OA						
F2f contacts	0.33*(0.21-0.53)	77.4	2.03*(1.09-3.78)	0.0	0.93 (0.45-1.89)	31.0
Non-f2f contacts	4.14*(2.69-6.37)	75.5	1.50 (0.83-2.71)	0.0	6.29*(2.95-13.41)	41.4
F2f and Non-f2f contacts	1.19 (0.86-1.64)	58.6	1.64 (0.92-2.95)	0.0	2.38*(1.23-4.60)	28.5
Cancelled appointments or DNAs	0.99 (0.69-1.42)	58.7	1.58 (0.74-3.39)	0.0	1.72 (0.85-3.49)	0.0
Caseload	0.91^(0.85-0.97)	99.8	1.02 (0.99-1.06)	99.3	0.94 (0.89-1.00)	99.7

^p<0.05; *p<0.001

AMH: adult mental health service; CAMHS: child and adolescent mental health service; EIP: early intervention for psychosis service; HTT: home treatment team; OA: older adult service.

For peer review only

Supplementary Table 5. Meta-analysed effects of lockdown announcements on mortality and mental health service use activity further adjusted for national covid-19 related deaths.

	Lockdown Announcement vs Pre-lockdown		Lift of Lockdown Announcement Vs lockdown		Lift of Lockdown vs. Pre-lockdown	
**Measures**	IRR (95%CI)	I^2	IRR (95%CI)	I^2	IRR (95%CI)	I^2
Number of deaths	1.74*(1.23-2.46)	88.1	0.44*(0.24-0.78)	92.1	0.71 (0.42-1.21)	90.9
Trust-wide activity						
Number of new referrals accepted	0.69*(0.59-0.81)	0.0	1.28*(1.07-1.53)	0.0	0.90 (0.78-1.04)	0.0
Number of discharges	1.10 (0.95-1.29)	0.0	0.88 (0.75-1.04)	0.0	0.96 (0.84-1.11)	0.0
Inpatient care						
New admissions	0.71*(0.60-0.85)	22.9	1.18 (1.00-1.41)	0.0	0.86 (0.73-1.01)	7.1
Discharges	1.60*(1.32-1.94)	1.3	0.52*(0.41-0.66)	0.0	0.86 (0.69-1.08)	0.0
Inpatient caseload	0.89*(0.84-0.95)	99.4	1.03 (0.97-1.09)	99.2	0.91^(0.84-0.99)	99.8
Inpatient caseload on MHA section	0.95 (0.89-1.01)	98.3	0.99 (0.90-1.08)	99.4	0.93 (0.82-1.05)	99.8
AMH (community)						
F2f contacts	0.59*(0.45-0.77)	41.4	0.94 (0.76-1.16)	0.0	0.61*(0.43-0.86)	50.0
Non-f2f contacts	4.09*(2.55-6.55)	81.1	1.27*(1.05-1.52)	0.0	4.07*(2.62-6.32)	69.6
F2f and Non-f2f contacts	1.04 (0.90-1.20)	0.0	1.12 (0.94-1.32)	0.0	1.18 (0.99-1.40)	0.0
Cancelled appointments or DNAs	0.99 (0.66-1.47)	66.4	0.87 (0.62-1.23)	35.9	0.86 (0.60-1.23)	50.1
Caseload	1.01 (1.00-1.02)	85.1	1.01 (1.00-1.02)	95.1	1.04^(1.02-1.07)	98.8
CAMHS						
F2f contacts	0.36*(0.16-0.84)	92.1	1.11 (0.77-1.60)	16.7	0.38*(0.16-0.91)	86.3
Non-f2f contacts	4.05*(2.63-6.24)	74.2	1.23^(1.02-1.49)	0.0	3.97*(2.78-5.66)	47.7
F2f and Non-f2f contacts	1.12 (0.88-1.41)	26.2	1.22 (0.99-1.51)	0.0	1.24 (1.00-1.52)	2.1
Cancelled appointments or DNAs	1.09 (0.81-1.47)	45.7	0.94 (0.73-1.22)	15.8	0.98 (0.81-1.18)	0.0
Caseload	0.95 (0.92-0.99)	99.5	1.03 (0.90-1.19)	99.7	0.95 (0.91-1.00)	99.6
EIP						
F2f contacts	0.33*(0.21-0.51)	74.6	1.40^(1.02-1.91)	38.3	0.48*(0.30-0.75)	76.9
Non-f2f contacts	4.56*(2.57-8.11)	85.4	1.45 (0.97-2.17)	57.5	6.07*(2.92-12.60)	88.6
F2f and Non-f2f contacts	1.15 (0.89-1.48)	50.3	1.41*(1.20-1.66)	0.0	1.53*(1.20-1.94)	40.4
Cancelled appointments or DNAs	0.77 (0.48-1.26)	75.8	1.01 (0.74-1.37)	36.4	0.70 (0.38-1.29)	87.0
Caseload	1.04^(1.02-1.05)	93.6	1.03(0.99-1.07)	99.3	1.08^(1.03-1.13)	99.7
HTT						
F2f contacts	0.44*(0.34-0.58)	92.5	1.50*(1.39-1.61)	0.0	0.65*(0.51-0.83)	92.3
Non-f2f contacts	1.70*(1.48-1.95)	20.7	1.08 (0.83-1.41)	70.6	1.80*(1.34-2.40)	80.6
F2f and Non-f2f contacts	0.67*(0.53-0.84)	92.5	1.30*(1.14-1.47)	67.5	0.89 (0.75-1.06)	86.6
Cancelled appointments or DNAs	0.56*(0.37-0.83)	83.5	1.63*(1.08-2.44)	77.9	0.97 (0.78-1.21)	37.5
Caseload	0.65*(0.52-0.83)	99.5	0.95 (0.67-1.35)	99.8	0.62*(0.45-0.86)	99.7
Liaison						
F2f contacts	0.48*(0.41-0.55)	57.6	1.63*(1.38-1.93)	51.1	0.77*(0.66-0.91)	57.3
Non-f2f contacts	1.52*(1.07-2.15)	72.0	1.20 (0.83-1.75)	62.8	1.73*(1.18-2.54)	68.8
F2f and Non-f2f contacts	0.62*(0.55-0.70)	46.8	1.43*(1.22-1.68)	51.4	0.88 (0.77-1.01)	42.4
Cancelled appointments or DNAs	0.81 (0.39-1.71)	88.7	0.68 (0.39-1.16)	53.0	0.72 (0.43-1.21)	60.9
Caseload	0.86*(0.81-0.91)	97.8	1.13*(1.06-1.21)	98.2	0.96 (0.92-1.00)	95.7
OA						
F2f contacts	0.39*(0.23-0.66)	81.9	1.31 (0.95-1.80)	24.6	0.46*(0.26-0.85)	80.4
Non-f2f contacts	3.84*(2.43-6.08)	82.3	1.24 (1.00-1.52)	0.0	4.20*(2.61-6.77)	71.1
F2f and Non-f2f contacts	1.09 (0.80-1.48)	61.2	1.30*(1.06-1.60)	0.0	1.31 (0.93-1.85)	48.1
Cancelled appointments or DNAs	1.34 (0.90-1.99)	63.8	0.63 (0.33-1.23)	74.9	0.88 (0.49-1.57)	70.0
Caseload	0.97 (0.95-1.00)	98.8	0.98 (0.92-1.04)	99.7	0.95 (0.89-1.02)	99.8

^p<0.05; *p<0.001

AMH: adult mental health service; CAMHS: child and adolescent mental health service; EIP: early intervention for psychosis service; HTT: home treatment team; OA: older adult service.

For peer review only

Supplementary Table 6. Meta-analysed effects of the lockdown announcement on mortality and mental health service use activity with the use of equally spaced windows around the cut-off date of 23rd March

	Lockdown Announcement Vs Pre-lockdown		Lift of the Lockdown vs. Pre-lockdown	
**Measures**	IRR (95%CI)	I^2	IRR (95%CI)	I^2
Number of deaths	2.13 (1.96-2.31)	4.1	0.82 (0.55-1.21)	86.0
Trust-wide activity				
Number of new referrals accepted	0.65 (0.58-0.74)	0.0	0.88 (0.74-1.03)	0.0
Number of discharges	0.86 (0.77-0.96)	0.0	0.88 (0.74-1.04)	3.4
Inpatient care				
New admissions	0.79 (0.69-0.90)	24.2	0.97 (0.83-1.12)	0.0
Discharges	1.04 (0.88-1.23)	0.0	0.82 (0.65-1.03)	0.0
Inpatient caseload	0.85 (0.80-0.91)	99.4	0.93 (0.86-0.99)	99.8
Inpatient caseload on MHA section	0.95 (0.90-1.01)	98.8	0.94 (0.84-1.06)	99.8
AMH (community)				
F2f contacts	0.56 (0.45-0.70)	40.3	0.68 (0.53-0.88)	0.0
Non-f2f contacts	3.70 (2.63-5.20)	77.6	3.32 (2.50-4.42)	23.7
F2f and Non-f2f contacts	1.16 (1.02-1.33)	0.0	1.34 (1.07-1.69)	0.0
Cancelled appointments or DNAs	0.77 (0.59-1.02)	59.3	0.87 (0.60-1.26)	39.6
Caseload	1.05 (1.03-1.06)	93.8	1.04 (1.03-1.06)	97.7
CAMHS				
F2f contacts	0.37 (0.18-0.77)	90.5	0.40 (0.19-0.83)	71.3
Non-f2f contacts	3.89 (2.83-5.34)	60.3	3.63 (2.23-5.91)	56.5
F2f and Non-f2f contacts	1.32 (1.11-1.55)	0.0	1.37 (1.03-1.81)	0.0
Cancelled appointments or DNAs	0.83 (0.66-1.04)	26.0	0.79 (0.59-1.06)	0.0
Caseload	0.96 (0.93-0.99)	99.0	0.96 (0.92-1.00)	99.4
EIP				
F2f contacts	0.30 (0.20-0.46)	81.5	0.45 (0.31-0.66)	52.8
Non-f2f contacts	3.65 (2.39-5.59)	80.8	4.30 (2.73-6.78)	64.7
F2f and Non-f2f contacts	1.22 (1.05-1.40)	11.9	1.48 (1.19-1.84)	0.0
Cancelled appointments or DNAs	0.69 (0.47-1.01)	75.2	0.58 (0.29-1.16)	85.6
Caseload	1.06 (1.04-1.09)	98.3	1.10 (1.04-1.16)	99.7
HTT				
F2f contacts	0.44 (0.33-0.60)	96.3	0.70 (0.57-0.87)	88.3
Non-f2f contacts	1.69 (1.47-1.94)	53.6	1.62 (1.19-2.22)	88.1
F2f and Non-f2f contacts	0.82 (0.75-0.91)	74.7	0.97 (0.84-1.13)	82.8
Cancelled appointments or DNAs	0.74 (0.61-0.90)	68.5	1.10 (0.87-1.38)	54.9
Caseload	0.59 (0.46-0.77)	99.5	0.62 (0.46-0.85)	99.7
Liaison				
F2f contacts	0.50 (0.42-0.59)	77.2	0.75 (0.64-0.88)	47.7
Non-f2f contacts	1.56 (1.20-2.03)	64.6	1.50 (1.10-2.06)	52.3
F2f and Non-f2f contacts	0.66 (0.57-0.76)	70.3	0.84 (0.75-0.94)	10.8
Cancelled appointments or DNAs	0.62 (0.39-0.99)	69.4	1.08 (0.76-1.53)	0.0
Caseload	0.77 (0.70-0.84)	99.2	0.90 (0.82-1.00)	99.1
OA				
F2f contacts	0.34 (0.21-0.53)	82.7	0.48 (0.29-0.80)	64.7
Non-f2f contacts	3.39 (2.38-4.82)	72.2	3.60 (2.38-5.43)	49.3
F2f and Non-f2f contacts	1.10 (0.87-1.40)	46.8	1.32 (0.92-1.87)	38.2
Cancelled appointments or DNAs	0.90 (0.65-1.26)	64.5	0.66 (0.43-1.03)	39.8
Caseload	0.93 (0.91-0.96)	98.7	0.93 (0.87-1.00)	99.8

AMH: adult mental health service; CAMHS: child and adolescent mental health service; EIP: early intervention for psychosis service; HTT: home treatment team; OA: older adult service.

For peer review only

For peer review only - http://bmjopen.bmj.com/site/about/guidelines.xhtml

Supplementary Table 7. Meta-analysed effects of lockdown announcements on mortality and mental health service use activity further adjusted for covid-19 google trends in United Kingdom

	Lockdown Announcement Vs Pre-lockdown		Lift of Lockdown Announcement Vs Lockdown		Lift of Lockdown vs. Pre-lockdown	
**Measures**	IRR (95%CI)	I^2	IRR (95%CI)	I^2	IRR (95%CI)	I^2
Number of deaths	2.30(1.92-2.75)	55.1	0.39(0.23-0.67)	92.5	0.90(0.59-1.38)	87.7
Trust-wide activity						
Number of new referrals accepted	0.68(0.58-0.79)	0.0	1.32(1.10-1.57)	0.0	0.95(0.84-1.07)	0.0
Number of discharges	0.85(0.73-0.99)	0.0	1.01(0.85-1.19)	0.0	0.87(0.77-0.99)	0.0
Inpatient care						
New admissions	0.78(0.67-0.91)	15.1	1.21(1.02-1.43)	0.0	0.92(0.79-1.06)	0.0
Discharges	0.73(0.59-0.91)	0.0	0.78(0.60-1.02)	8.6	0.54(0.44-0.67)	0.0
Inpatient caseload	0.84(0.77-0.90)	99.1	1.05(0.98-1.13)	99.1	0.88(0.82-0.94)	99.4
Inpatient caseload on MHA section	0.91(0.84-0.99)	99.3	0.99(0.90-1.09)	99.6	0.90(0.85-0.96)	98.8
AMH (community)						
F2f contacts	0.64(0.51-0.80)	0.0	0.92(0.71-1.17)	0.0	0.59(0.47-0.72)	4.3
Non-f2f contacts	1.69(1.38-2.08)	0.0	1.49(1.21-1.83)	0.0	2.50(2.12-2.94)	0.0
F2f and Non-f2f contacts	1.03(0.85-1.25)	0.0	1.11(0.90-1.37)	0.0	1.19(1.03-1.38)	0.0
Cancelled appointments or DNAs	0.63(0.50-0.79)	0.0	1.08(0.82-1.42)	9.0	0.70(0.50-0.97)	42.1
Caseload	1.02(1.01-1.03)	92.8	1.01(1.00-1.02)	95.0	1.03(1.03-1.04)	87.1
CAMHS						
F2f contacts	0.50(0.28-0.90)	73.3	1.20(0.83-1.73)	0.0	0.56(0.28-1.11)	72.4
Non-f2f contacts	2.19(1.51-3.18)	40.0	1.50(1.19-1.90)	0.0	3.11(2.07-4.67)	47.8
F2f and Non-f2f contacts	1.08(0.87-1.35)	0.0	1.22(0.95-1.56)	0.0	1.29(1.06-1.57)	0.0
Cancelled appointments or DNAs	0.65(0.51-0.83)	0.0	1.15(0.82-1.61)	20.4	0.77(0.61-0.97)	8.5
Caseload	0.98(0.96-1.00)	96.6	0.99(0.96-1.01)	98.7	0.97(0.92-1.02)	99.1
EIP						
F2f contacts	0.37(0.23-0.58)	65.3	1.50(1.01-2.23)	50.4	0.55(0.33-0.92)	78.4
Non-f2f contacts	1.69(1.18-2.43)	45.8	1.88(1.47-2.41)	0.0	2.78(2.30-3.36)	0.0
F2f and Non-f2f contacts	0.90(0.73-1.12)	0.0	1.43(1.15-1.80)	0.0	1.28(1.09-1.50)	0.0
Cancelled appointments or DNAs	0.66(0.52-0.85)	0.0	0.97(0.65-1.46)	50.1	0.60(0.35-1.04)	80.4
Caseload	1.04(1.02-1.05)	96.8	1.04(0.99-1.08)	99.6	1.07(1.02-1.12)	99.8
HTT						
F2f contacts	0.60(0.48-0.75)	90.3	1.39(1.29-1.49)	8.2	0.84(0.71-0.99)	86.4
Non-f2f contacts	1.43(1.21-1.69)	51.0	1.14(0.95-1.36)	55.7	1.57(1.19-2.07)	85.8
F2f and Non-f2f contacts	0.88(0.81-0.96)	57.5	1.25(1.16-1.34)	31.4	1.08(0.95-1.21)	80.2
Cancelled appointments or DNAs	0.78(0.64-0.94)	49.4	1.44(1.08-1.93)	66.6	1.12(0.89-1.40)	57.1
Caseload	0.68(0.55-0.84)	99.0	1.16(1.01-1.35)	98.1	0.80(0.73-0.87)	96.5
Liaison						
F2f contacts	0.61(0.53-0.70)	39.0	1.40(1.24-1.57)	7.4	0.81(0.65-1.01)	76.3
Non-f2f contacts	1.06(0.89-1.28)	0.0	1.34(0.89-2.03)	62.1	1.46(0.97-2.19)	69.3
F2f and Non-f2f contacts	0.71(0.63-0.80)	23.7	1.33(1.17-1.51)	15.4	0.91(0.75-1.11)	68.5
Cancelled appointments or DNAs	0.56(0.37-0.87)	42.0	0.73(0.39-1.34)	41.6	0.57(0.35-0.95)	33.8
Caseload	0.88(0.82-0.94)	97.5	1.08(1.02-1.14)	95.4	0.94(0.86-1.03)	98.4
OA						
F2f contacts	0.46(0.29-0.71)	61.8	1.25(0.93-1.68)	4.1	0.48(0.26-0.87)	79.1
Non-f2f contacts	1.63(1.27-2.10)	0.0	1.51(1.16-1.97)	0.0	2.65(1.91-3.66)	30.1
F2f and Non-f2f contacts	0.91(0.72-1.15)	0.0	1.28(0.99-1.65)	0.0	1.14(0.89-1.46)	12.5
Cancelled appointments or DNAs	0.67(0.51-0.88)	0.0	0.88(0.41-1.90)	75.0	0.60(0.33-1.10)	67.1
Caseload	0.95(0.93-0.97)	97.8	0.99(0.93-1.04)	99.4	0.94(0.88-1.01)	99.8

^p<0.05; *p<0.001
AMH: adult mental health service; CAMHS: child and adolescent mental health service; EIP: early intervention for psychosis service; HTT: home treatment team; OA: older adult service.

For peer review only

Supplementary Figure 1. Number of deaths per day before and after lockdown announcement for sites A, F, G and J during the period of 1st January 2020 to 31st July 2020. The vertical axis shows the daily number of deaths in the days before (orange dots) and after (red dots) the lockdown order was announced in 23rd March 2020 and after the lift (purple dots) of the lockdown order announced in 10th May. The green dots represent the number of daily deaths during the period of 1st January 2019 to 31st July 2019.

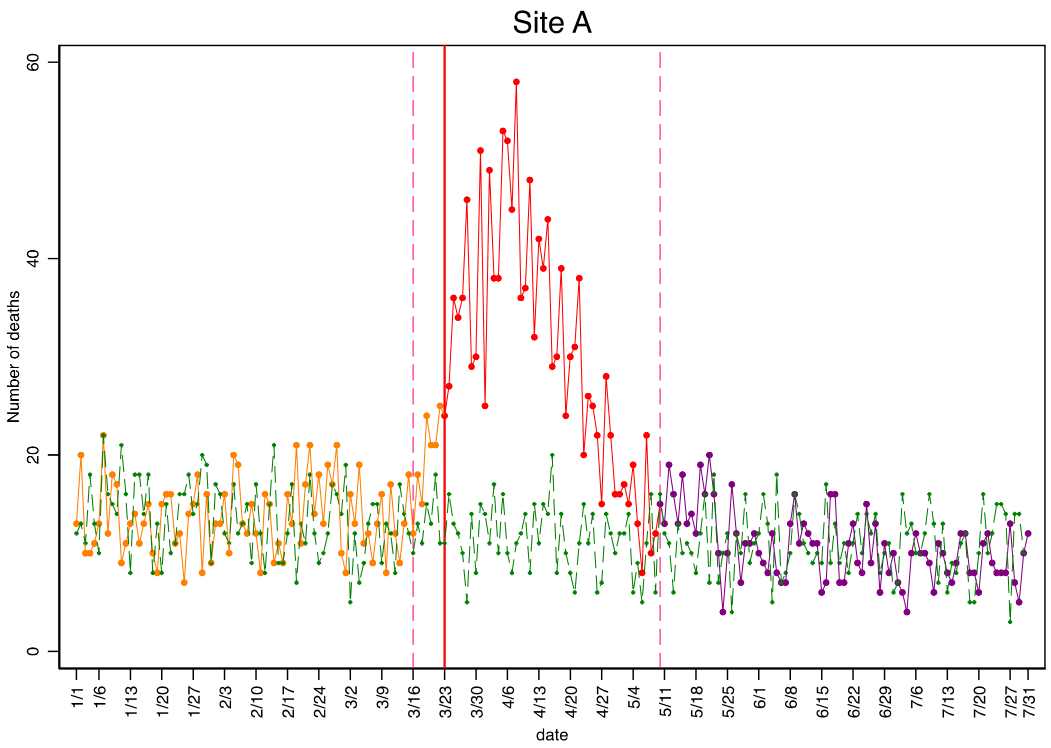

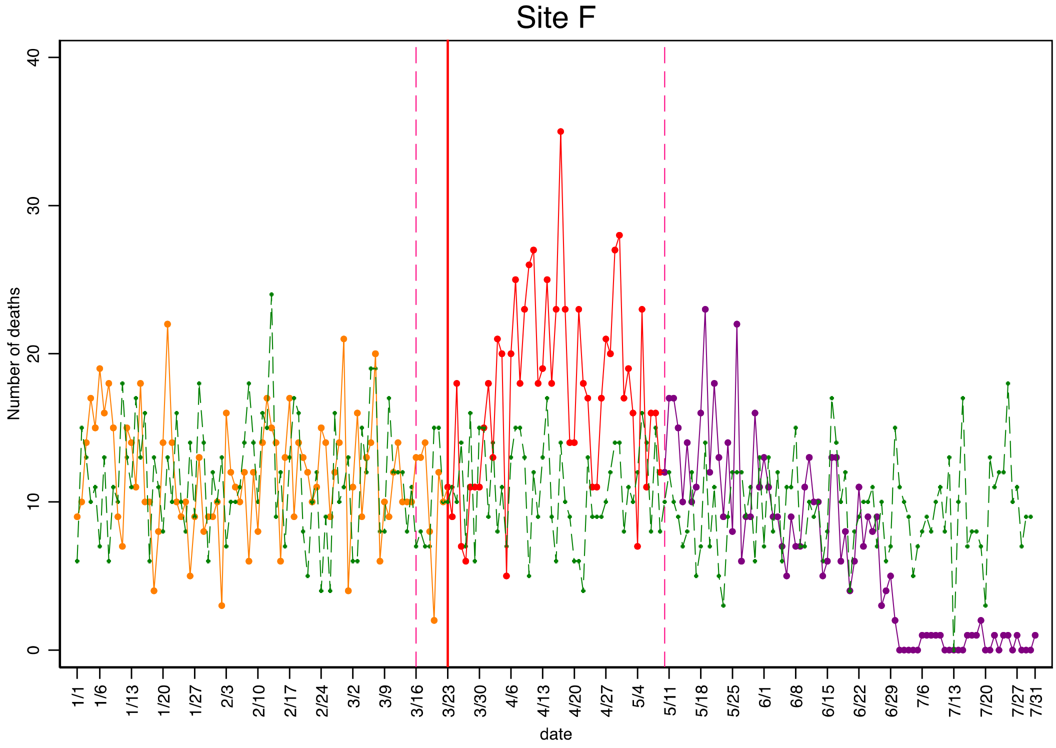

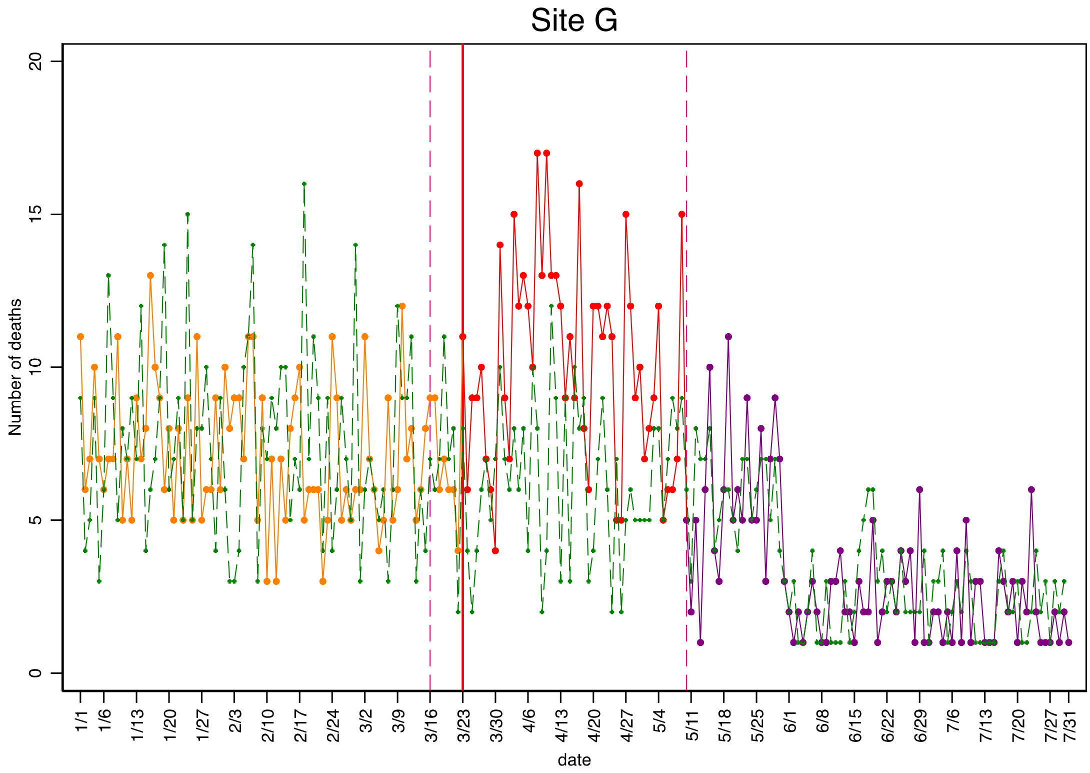
Site G
Number of deaths
0
5
10
15
20
1/1
1/6
1/13
1/20
1/27
2/3
2/10
2/17
2/24
3/2
3/9
3/16
3/23
3/30
4/6
4/13
4/20
4/27
5/4
5/11
5/18
5/25
6/1
6/8
6/15
6/22
6/29
7/6
7/13
7/20
7/27
7/31
date

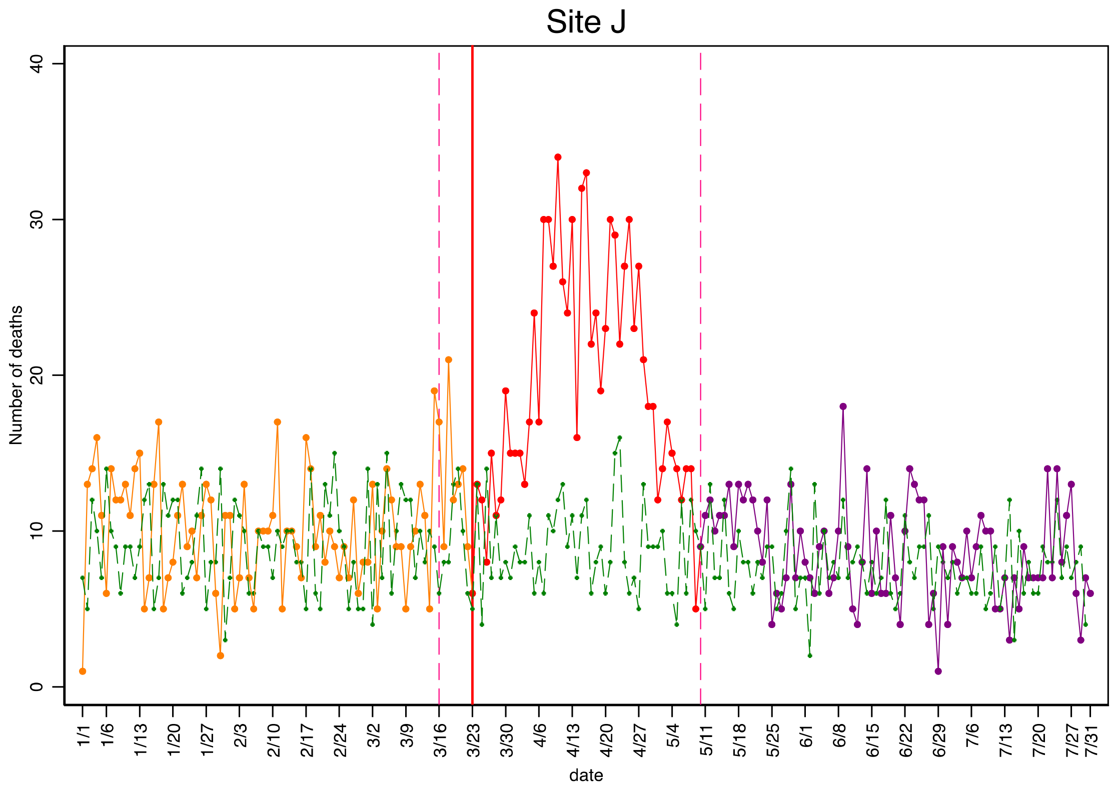
Site J
Number of deaths
0
10
20
30
40
1/1
1/6
1/13
1/20
1/27
2/3
2/10
2/17
2/24
3/2
3/9
3/16
3/23
3/30
4/6
4/13
4/20
4/27
5/4
5/11
5/18
5/25
6/1
6/8
6/15
6/22
6/29
7/6
7/13
7/20
7/27
7/31
date

Supplementary Figure 2. Number of deaths (A) and inpatient admissions (B) per week before and after lockdown announcement for site I during the period of 1st January 2020 to 5th July 2020. The vertical axis shows the daily number of deaths in the days before (orange dots) and after (red dots) the lockdown order was announced on 23rd March 2020 and after the lift (purple dots) of the lockdown order announced in 10th May. The green dots represent the number of daily deaths during the period of 1st January 2019 to 5th July 2019.

A

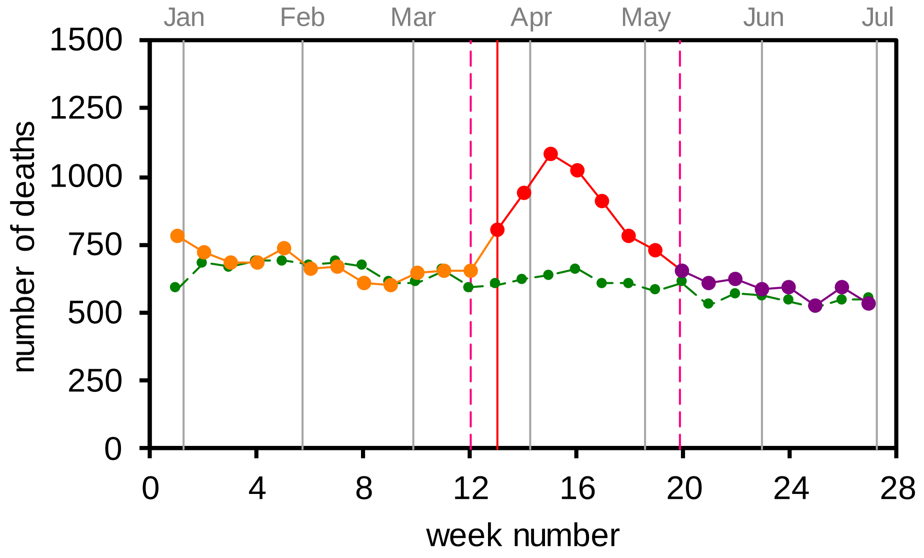

B

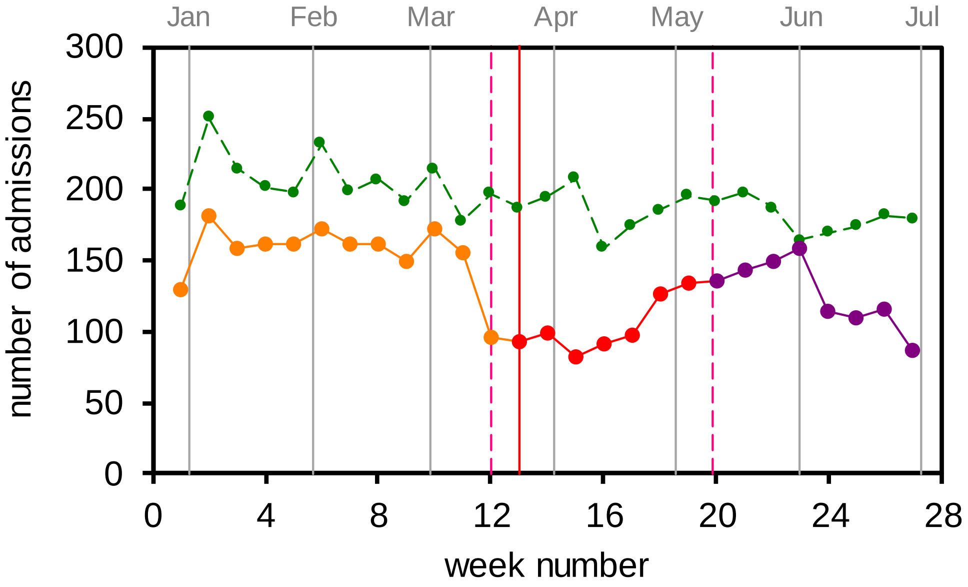

Supplementary Figure 3. Number of deaths per day before and after lockdown announcement for sites B, C, D, E, H and I during the period of 1st January 2020 to 31st May 2020. The vertical axis shows the daily number of deaths in the days before (orange dots) and after (red dots) the lockdown order was announced in 23rd March 2020 and after the lift (purple dots) of the lockdown order announced in 10th May. The green dots represent the number of daily deaths during the period of 1st January 2019 to 31st May 2019.

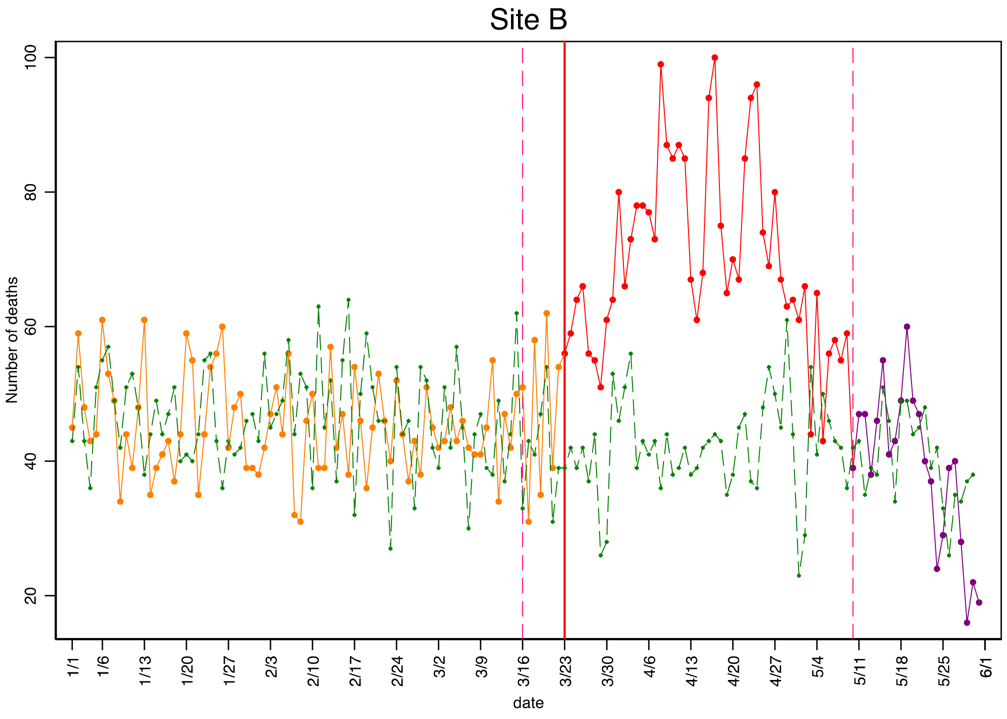

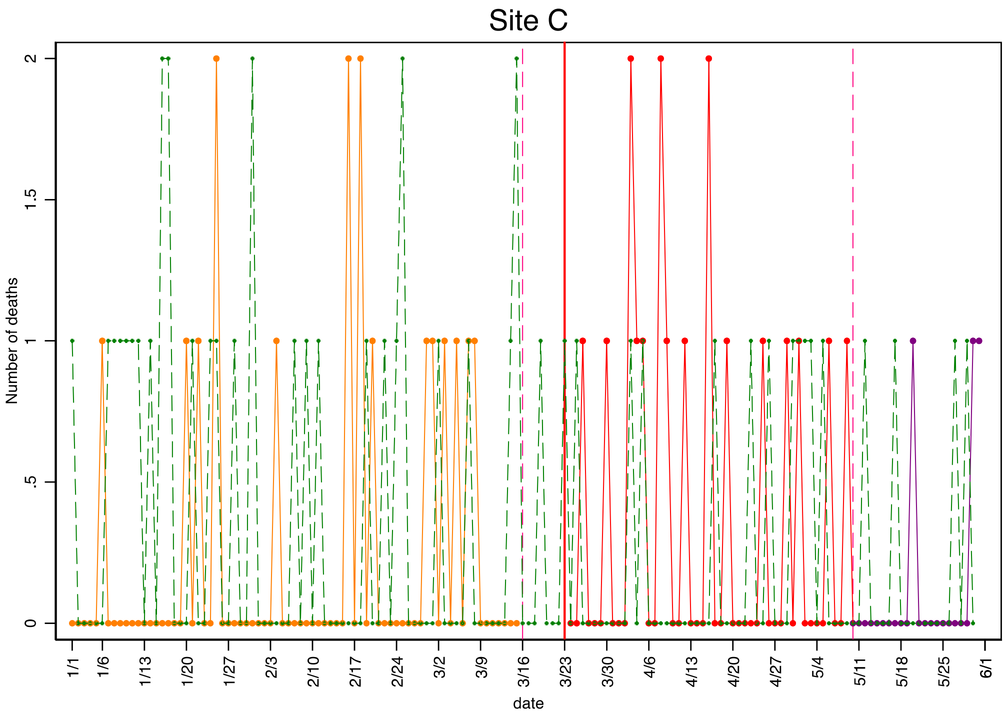

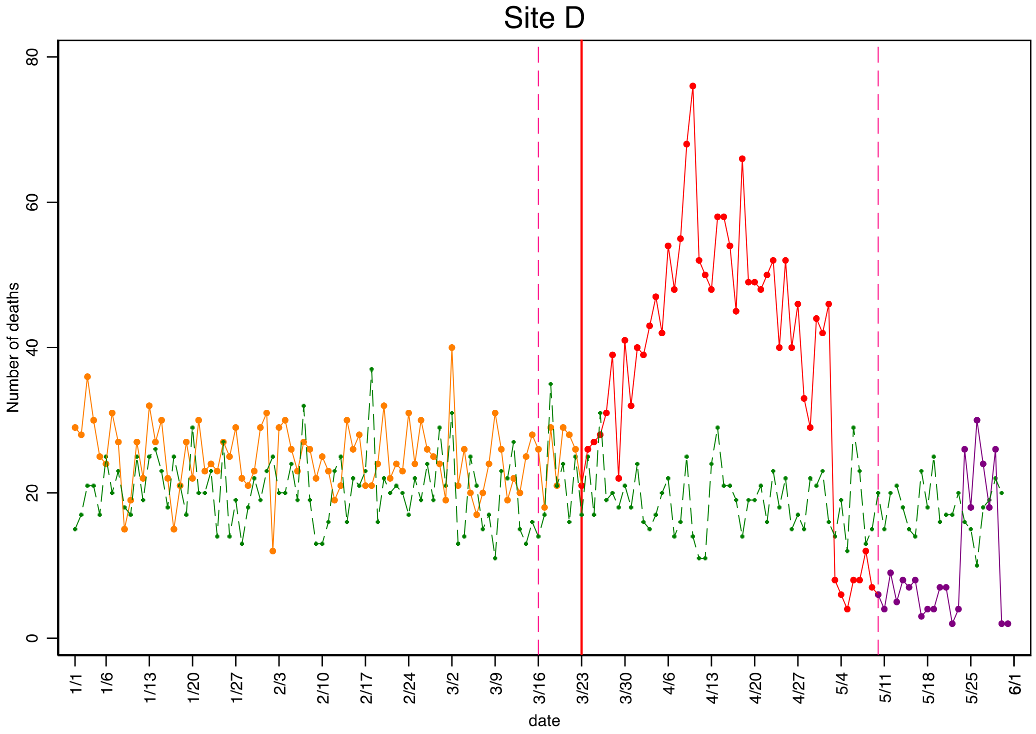
Site D
Number of deaths
0
20
40
60
80
date
1/1
1/6
1/13
1/20
1/27
2/3
2/10
2/17
2/24
3/2
3/9
3/16
3/23
3/30
4/6
4/13
4/20
4/27
5/4
5/11
5/18
5/25
6/1

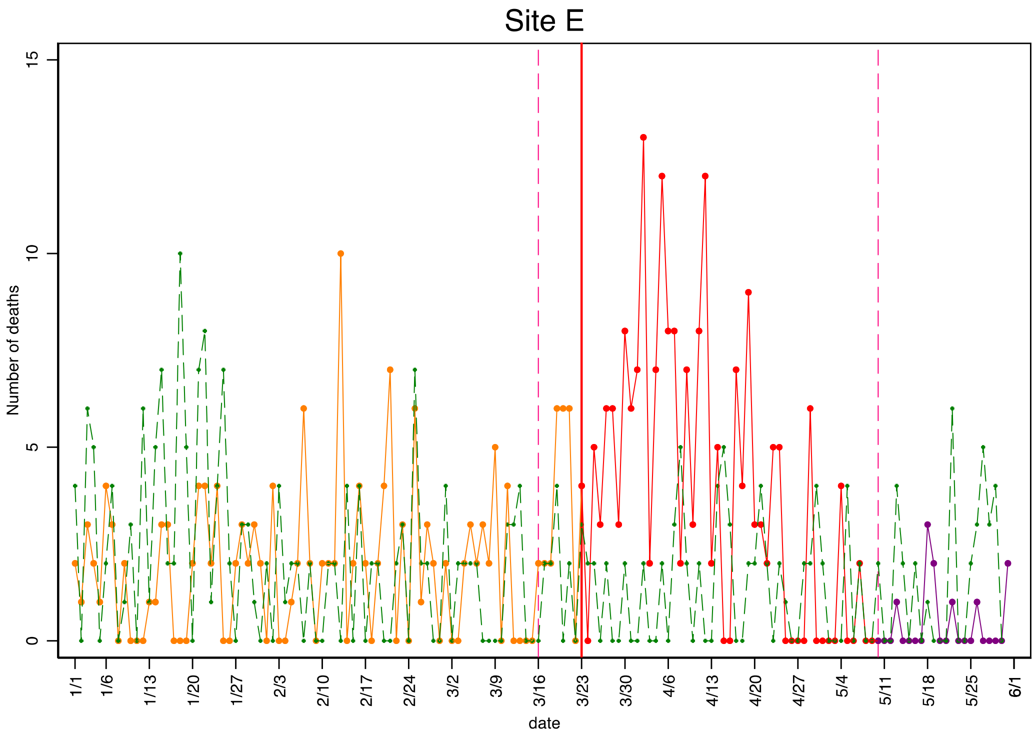
Site E
Number of deaths
0
5
10
15
date
1/1
1/6
1/13
1/20
1/27
2/3
2/10
2/17
2/24
3/2
3/9
3/16
3/23
3/30
4/6
4/13
4/20
4/27
5/4
5/11
5/18
5/25
6/1

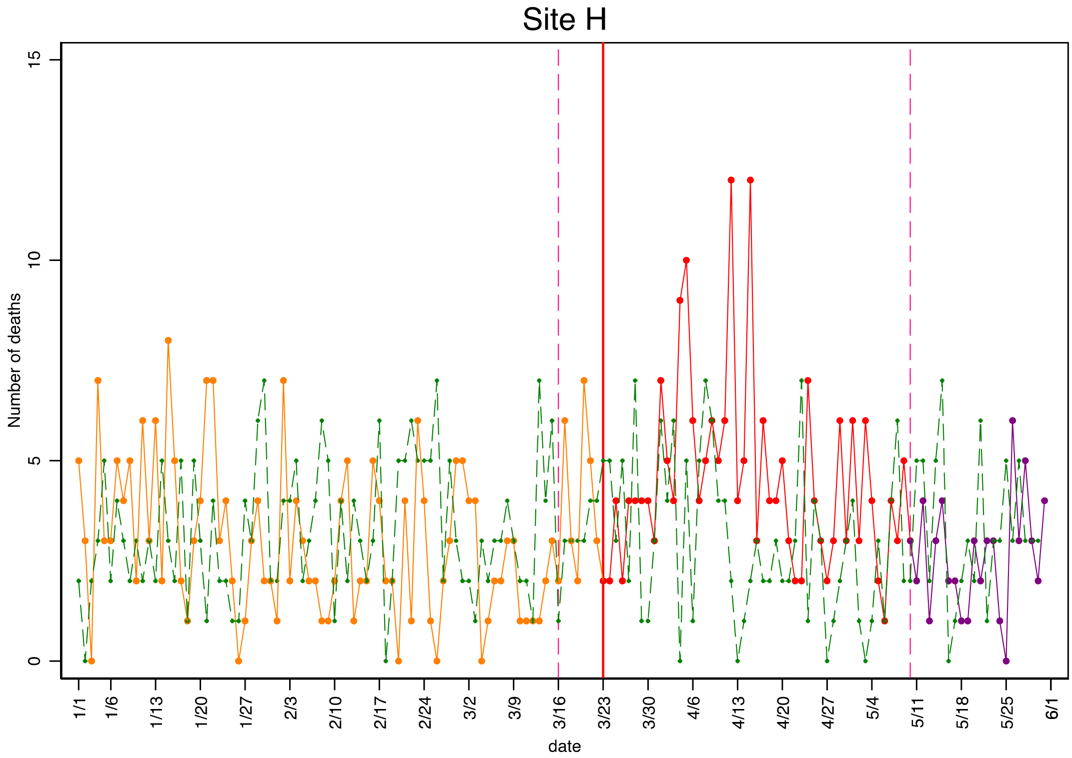

For peer review only

Supplementary Figure 4. Number of inpatient admissions per day before and after lockdown announcement for sites A, F and J during the period of 1st January 2020 to 31st July 2020. The vertical axis shows the daily number of deaths in the days before (orange dots) and after (red dots) the lockdown order was announced on 23rd March and after the lift (purple dots) of the lockdown order announced in 10th May. The green dots represent the number of daily deaths during the period of 1st January 2019 to 31st July 2019.

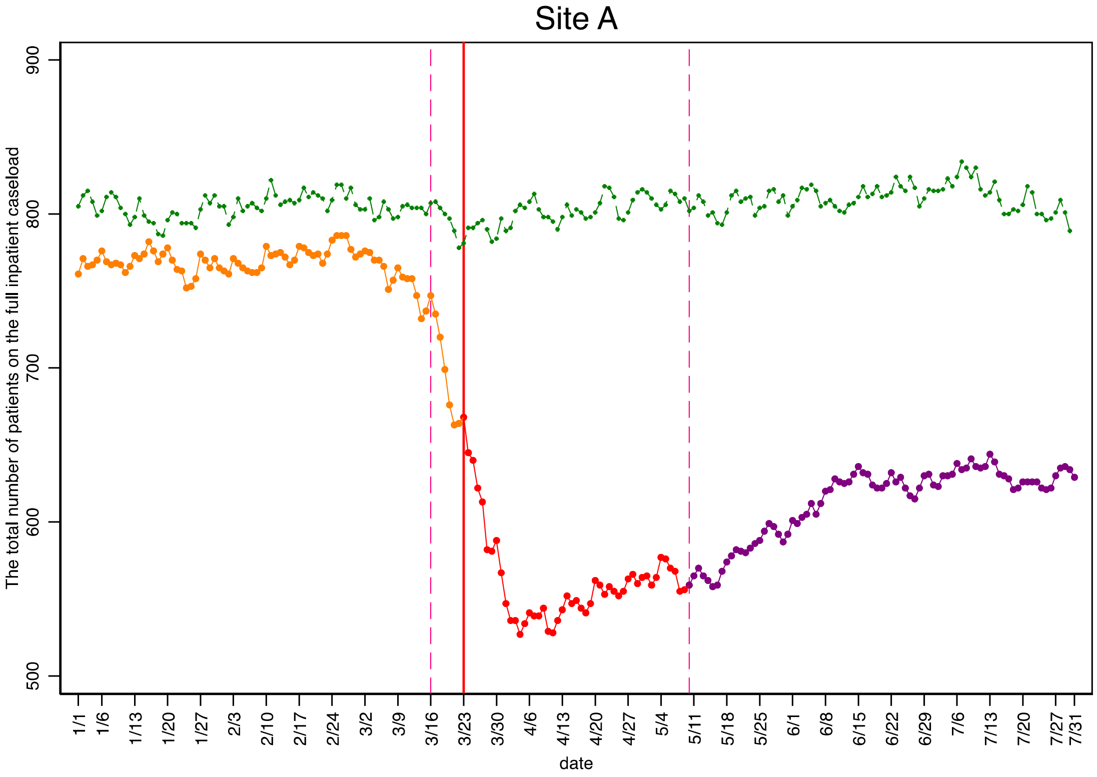

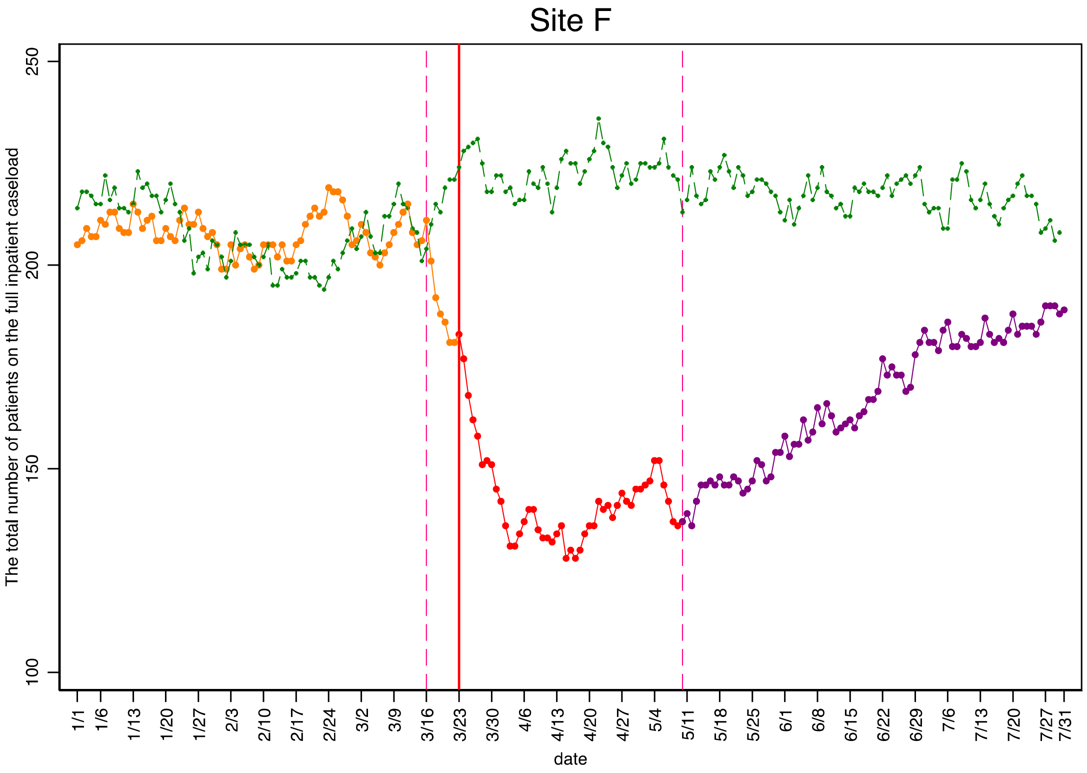

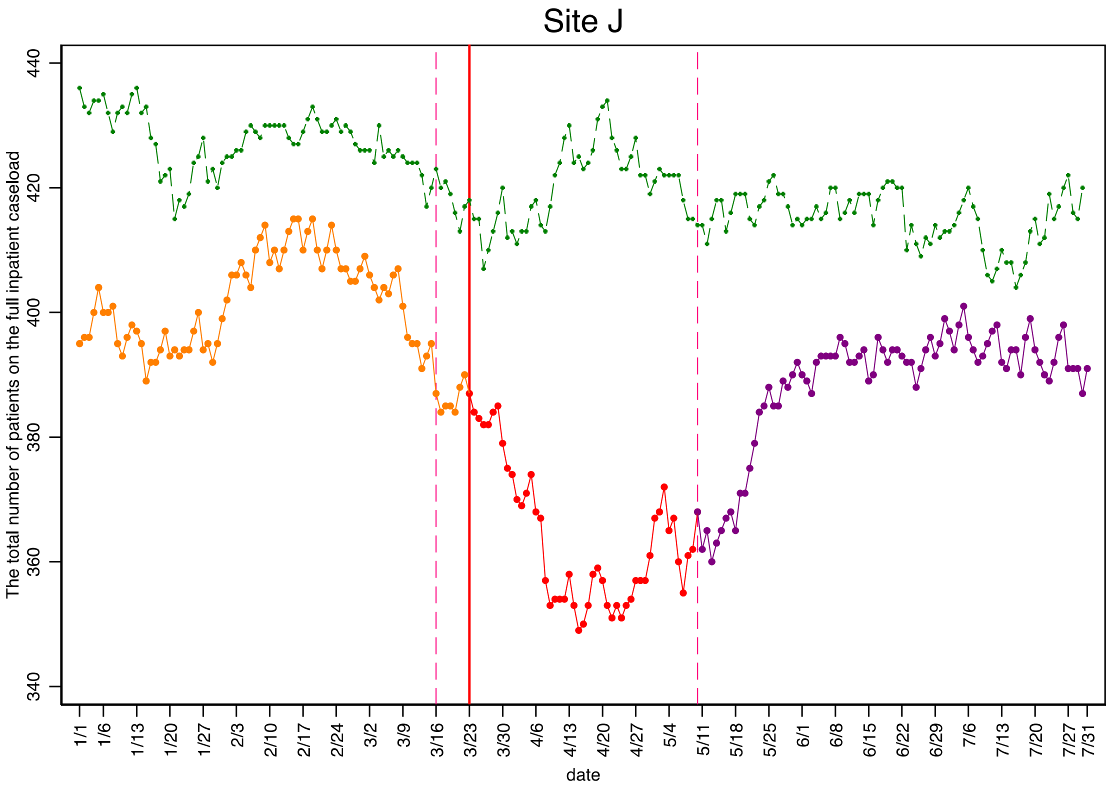
Site J
The total number of patients on the full inpatient caseload
440
420
400
380
360
340
1/1
1/6
1/13
1/20
1/27
2/3
2/10
2/17
2/24
3/2
3/9
3/16
3/23
3/30
4/6
4/13
4/20
4/27
5/4
5/11
5/18
5/25
6/1
6/8
6/15
6/22
6/29
7/6
7/13
7/20
7/27
7/31
date

review only

Supplementary Figure 5. Number of deaths per day before and after lockdown announcement for sites A, B, D, F, and J during the period of 1st January 2020 to 31st May 2020. The vertical axis shows the daily number of deaths in the days before (orange dots) and after (red dots) the lockdown order was announced on 23rd March and after the lift (purple dots) of the lockdown order announced on 10th May. The green dots represent the number of daily deaths during the period of 1st January 2019 to 31st May 2019. The lines represent the predicted values of our fitted model.

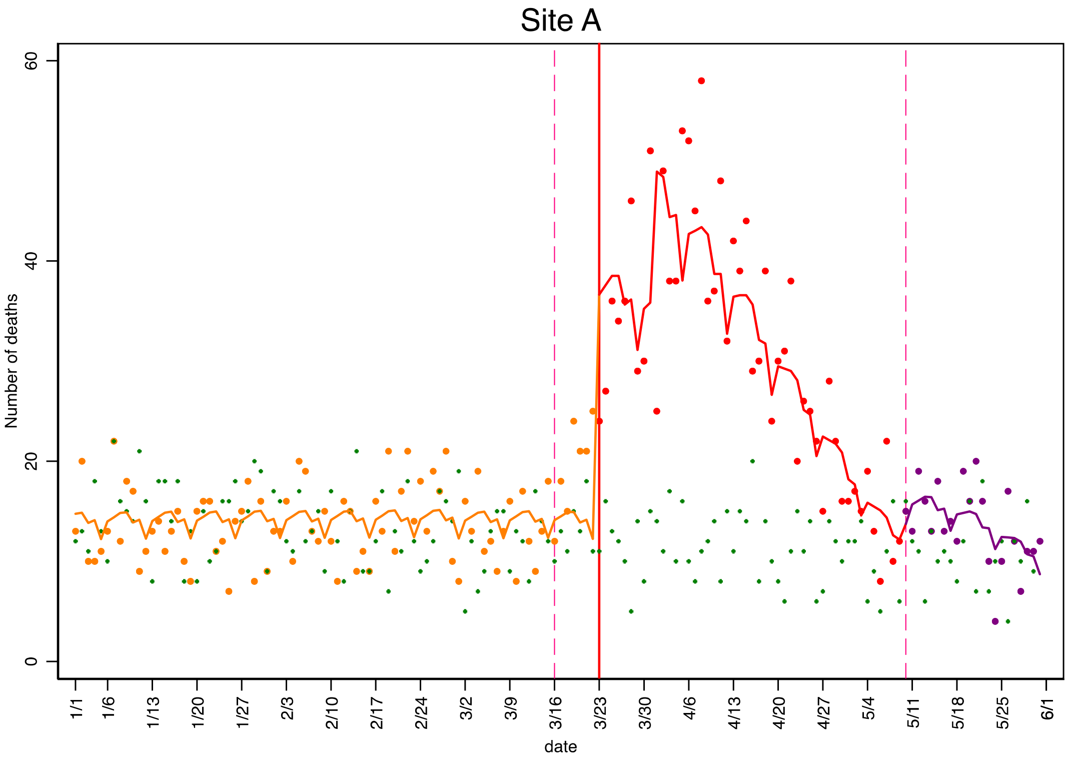

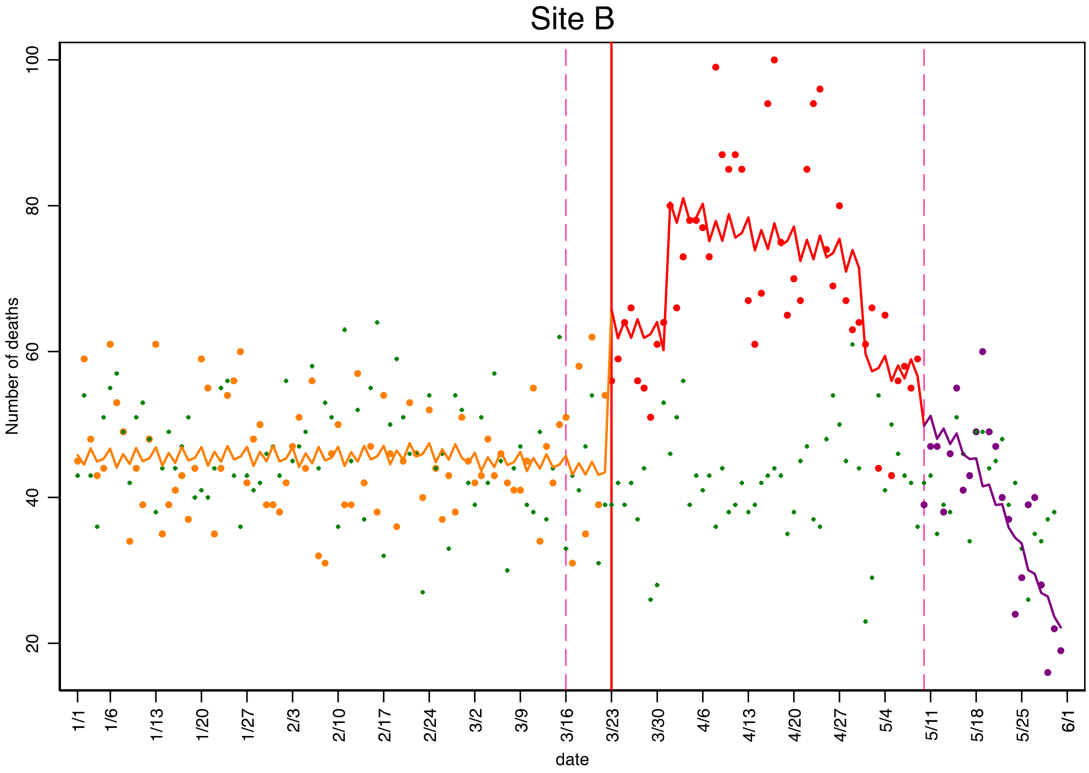

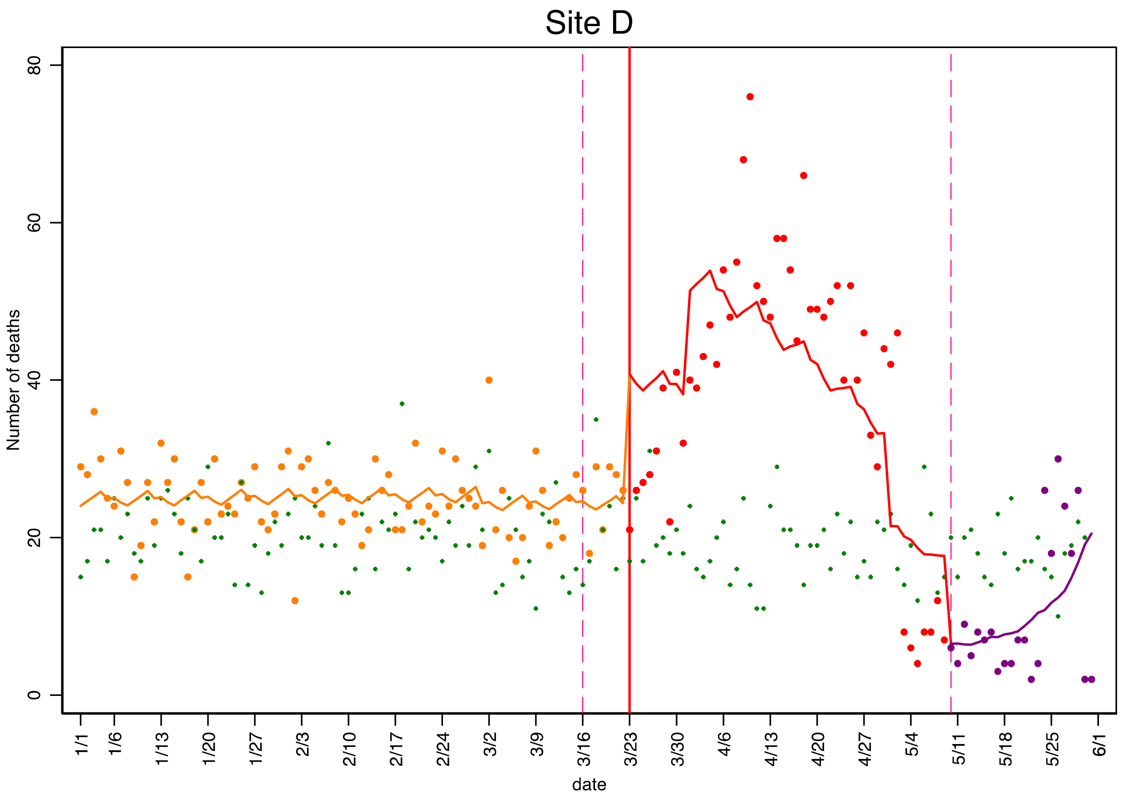
Site D
Number of deaths
0
20
40
60
80
1/1
1/6
1/13
1/20
1/27
2/3
2/10
2/17
2/24
3/2
3/9
3/16
3/23
3/30
4/6
4/13
4/20
4/27
5/4
5/11
5/18
5/25
6/1
date

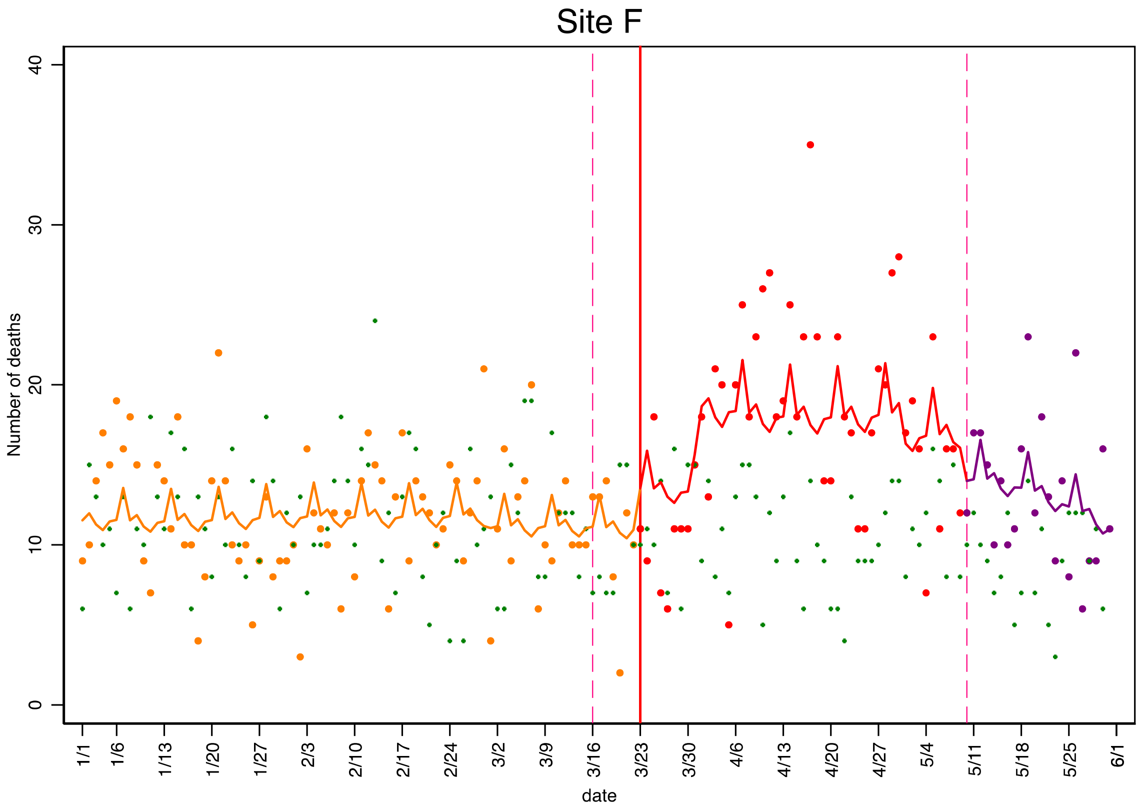
Site F
Number of deaths
0
10
20
30
40
1/1
1/6
1/13
1/20
1/27
2/3
2/10
2/17
2/24
3/2
3/9
3/16
3/23
3/30
4/6
4/13
4/20
4/27
5/4
5/11
5/18
5/25
6/1
date

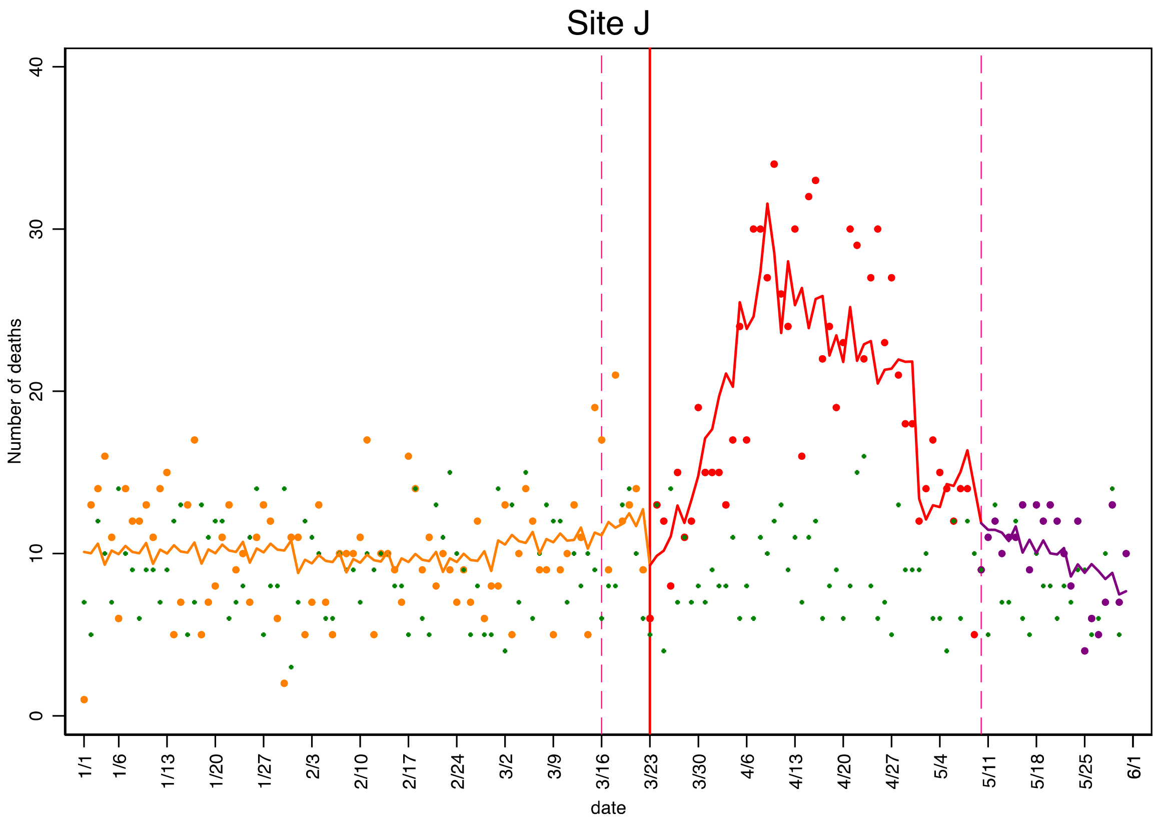

For peer review only - http://bmjopen.bmj.com/site/about/guidelines.xhtml

STROBE Statement—Checklist of items that should be included in reports of ***cross-sectional studies***

	Item No	Recommendation	Page No
**Title and abstract**	1	(*a*) Indicate the study's design with a commonly used term in the title or the abstract	1
		(*b*) Provide in the abstract an informative and balanced summary of what was done and what was found	5
**Introduction**			
Background/rationale	2	Explain the scientific background and rationale for the investigation being reported	7
Objectives	3	State specific objectives, including any prespecified hypotheses	7
**Methods**			
Study design	4	Present key elements of study design early in the paper	8-9
Setting	5	Describe the setting, locations, and relevant dates, including periods of recruitment, exposure, follow-up, and data collection	8-9, supplement
Participants	6	(*a*) Give the eligibility criteria, and the sources and methods of selection of participants	8-9
Variables	7	Clearly define all outcomes, exposures, predictors, potential confounders, and effect modifiers. Give diagnostic criteria, if applicable	9-10
Data sources/ measurement	8*	For each variable of interest, give sources of data and details of methods of assessment (measurement). Describe comparability of assessment methods if there is more than one group	8-10
Bias	9	Describe any efforts to address potential sources of bias	8-10
Study size	10	Explain how the study size was arrived at	N/A
Quantitative variables	11	Explain how quantitative variables were handled in the analyses. If applicable, describe which groupings were chosen and why	9-10
Statistical methods	12	(*a*) Describe all statistical methods, including those used to control for confounding	10-11, supplement
		(*b*) Describe any methods used to examine subgroups and interactions	10-11, supplement
		(*c*) Explain how missing data were addressed	N/A
		(*d*) If applicable, describe analytical methods taking account of sampling strategy	10-12, supplement
		(*e*) Describe any sensitivity analyses	10-12, supplement
**Results**			
Participants	13*	(a) Report numbers of individuals at each stage of study—eg numbers potentially eligible, examined for eligibility, confirmed eligible, included in the study, completing follow-up, and analysed	Table 1
		(b) Give reasons for non-participation at each stage	N/A
		(c) Consider use of a flow diagram	N/A
Descriptive data	14*	(a) Give characteristics of study participants (e.g. demographic, clinical, social) and information on exposures and potential confounders	Table 1
		(b) Indicate number of participants with missing data for each variable of interest	N/A

For peer review only

Outcome data	15*	Report numbers of outcome events or summary measures	Table 1
Main results	16	(*a*) Give unadjusted estimates and, if applicable, confounder-adjusted estimates and their precision (eg, 95% confidence interval). Make clear which confounders were adjusted for and why they were included	12-13, Table 2, supplement
		(*b*) Report category boundaries when continuous variables were categorized	N/A
		(*c*) If relevant, consider translating estimates of relative risk into absolute risk for a meaningful time period	N/A
Other analyses	17	Report other analyses done—eg analyses of subgroups and interactions, and sensitivity analyses	12-14, Table 3, supplement
**Discussion**			
Key results	18	Summarise key results with reference to study objectives	15
Limitations	19	Discuss limitations of the study, taking into account sources of potential bias or imprecision. Discuss both direction and magnitude of any potential bias	17-18
Interpretation	20	Give a cautious overall interpretation of results considering objectives, limitations, multiplicity of analyses, results from similar studies, and other relevant evidence	15-18
Generalisability	21	Discuss the generalisability (external validity) of the study results	17
**Other information**			
Funding	22	Give the source of funding and the role of the funders for the present study and, if applicable, for the original study on which the present article is based	2-3

*Give information separately for exposed and unexposed groups.

Note: An Explanation and Elaboration article discusses each checklist item and gives methodological background and published examples of transparent reporting. The STROBE checklist is best used in conjunction with this article (freely available on the Web sites of PLoS Medicine at http://www.plosmedicine.org/, Annals of Internal Medicine at http://www.annals.org/, and Epidemiology at http://www.epidem.com/). Information on the STROBE Initiative is available at www.strobe-statement.org.

For peer review only
